# A small molecule inhibitor of Notch1 modulates stemness and suppresses breast cancer cell growth

**DOI:** 10.3389/fphar.2023.1150774

**Published:** 2023-02-24

**Authors:** Uttara Saran, Balaji Chandrasekaran, Ashish Tyagi, Vaibhav Shukla, Amandeep Singh, Arun K. Sharma, Chendil Damodaran

**Affiliations:** ^1^ Texas A&M University, College Station, TX, United States; ^2^ Penn State Cancer Institute, College of Medicine, The Pennsylvania State University, Hershey, PA, United States

**Keywords:** breast cancer, breast cancer stem cell (BCSC), ASR490, autophagy, NOTCH1

## Abstract

Although breast cancer stem cells (BCSCs) are well characterized, molecularly targeting and eradicating this sub-population remains a challenge in the clinic. Recent studies have explored several signaling pathways that govern stem cell activation: We and others established that the Notch1 signaling plays a significant role in the proliferation, survival, and differentiation of BCSCs. Earlier, we reported that a newly developed small molecule, ASR490, binds to the negative regulatory region (NRR: The activation switch of the Notch receptor) of Notch1. *In vitro* results demonstrated that ASR490 significantly inhibited BCSCs (ALDH^+^ and CD44^+^/CD24^–^) and breast cancer (BC) growth at nM concentrations, and subsequently inhibited the colony- and mammosphere-forming abilities of BCSCs and BCs. ASR490 downregulated the expressions of Notch1 intracellular domain (NICD: The active form of Notch1) and its downstream effectors Hey1 and HES1. Inhibition of Notch1-NICD facilitated autophagy-mediated growth inhibition by triggering the fusion of autophagosome and autolysosome in BCSCs. ASR490 was found to be non-toxic to healthy cells as compared to existing Notch1 inhibitors. Moreover, oral administration of ASR490 abrogated BCSC and BC tumor growth in the *in vivo* xenograft models. Together our results indicate that ASR490 is a potential therapeutic agent that inhibits BC tumor growth by targeting and abolishing Notch1 signaling in BCSCs and BC cells.

## Introduction

Breast cancer stem cells (BCSCs) are a small population of cancer cells within breast tumors that are characterized by their self-renewal ability and relative resistance to current therapeutics (Al-Hajj et al., 2003). Furthermore, evidence suggests that BCSCs are not eliminated during cancer treatment (Shafee et al., 2008; Abravanel et al., 2015), consequently contributing to the development of therapeutic resistance and tumor recurrence in breast cancer (BC) patients (Dandawate et al., 2016; Bai et al., 2018). Hence, despite advances in early diagnosis and therapy (Harbeck et al., 2019), resistance and relapse of BC remain as significant challenges, resulting in this malignancy being the most prevalent cause of cancer-related deaths among women. Therefore, there is an urgent need to develop novel therapeutic strategies to target the signaling pathways responsible for BCSCs and eradicate this subpopulation in BC patients.

The aberrant activation of Notch signaling has been shown to play a significant role in BC progression by maintaining cancer stem cells (Majumder et al., 2021; Ranganathan et al., 2011; D'Angelo et al., 2015). Notch1 is predominantly active in BCSCs, and its aberrant activation has been reported to enhance BCSCs metastatic phenotype by promoting invasiveness and chemoresistance (Reedijk et al., 2005; Phillips et al., 2006; Dickson et al., 2007; Sajithlal et al., 2010; Zang et al., 2010; Krop et al., 2012; Bolos et al., 2013; Suman et al., 2013; Kim et al., 2015; Li et al., 2015). Overexpression of Notch1 has been correlated with a poor prognosis (Yuan et al., 2015) and decreased overall recurrence-free survival (Abravanel et al., 2015) in BC patients. Hence, Notch1 is an attractive target for eradicating BCSCs, and several preclinical studies have confirmed its potential role as a therapeutic target in cancer treatment.

Several strategies can target Notch1, including Notch1 monoclonal antibodies, siRNA, natural products, *γ*-secretase inhibitors, pan-Notch inhibitors, etc. (Sorrentino et al., 2019). We and others have shown that inhibition of Notch1 *in vitro* using either genetic or pharmacological approaches enhanced the antitumor efficacy of chemotherapy agents by suppressing BCSCs (Qiu et al., 2013; Suman et al., 2013; Cao et al., 2015; Zhou et al., 2017). These studies suggest that targeting Notch1 signaling of BCSCs could be an effective therapeutic strategy for eradicating and preventing metastatic disease. However, to date, Notch1-targeted therapies for BC have not advanced to clinical trials due to their toxicity and anticancer efficiency. Therefore, it is imperative to develop effective and safer Notch1-targeted treatments for BC that selectively inhibit in a way distinct from currently known Notch inhibitors to circumvent toxicity issues. Earlier, we reported the potency of a non-toxic small molecule Notch1 inhibitor, ASR490, a pyridine-2-carboxylic acid prodrug analog of Withaferin-A developed in our laboratories based on structure-activity relationship (SAR) studies on compounds generated by the modifications of Withaferin A core structure, in downregulating Notch1 expression and abrogating the growth of colon cancer cells (Tyagi et al., 2020).

Here, we explored whether ASR490 inhibited BCSC growth in the *in vitro* and *in vivo* models and dissected its mechanism of action. Our results suggested that ASR490 eradicated BCSCs and inhibited BC growth in preclinical models. Furthermore, molecular studies revealed that ASR490’s inhibition of Notch1 facilitated the induction of autophagy signaling resulting in the suppression of BCSCs.

## Materials and methods

### Cell culture and reagents

Human mammary immortalized cells (MCF10A), and the TNBC cell line MDA-MB-231 were purchased from American Type Culture Collection. MCF10A cells were grown in a 1:1 mixture of Dulbecco’s modified Eagle’s medium (DMEM) and Ham’s F12 medium with 20 ng mL^−1^ human epidermal growth factor, 100 ng mL^−1^ cholera toxin, 0.01 mg mL^−1^ bovine insulin, 500 ng mL^−1^ hydrocortisone, and 5% horse serum. MDA-MB-231 cells were grown in DMEM containing L-glutamine and sodium pyruvate, supplemented with 10% fetal bovine serum and 1% antibiotic and antimycotic solution in a humidified atmosphere of 5% CO2 at 37°C in an incubator. Human BCSC cells: ALDH^+^ and CD44^+^/CD24^−^, and BC cells: ALDH- were purchased from Celprogen (San Pedro, CA, United States) and maintained in human BCSC expansion and undifferentiation media. DAPT (γ-secretase), cycloheximide (CHX), chloroquine (CQ), and MG132 were purchased from Sigma (St. Louis, MO).

#### Synthesis of ASR490

ASR490 was synthesized in our laboratory as reported previously (Tyagi et al., 2020).

#### Cell viability assays

ALDH^−^, ALDH^+^, and CD44^+^/CD24^−^ cells were first treated with vehicle control (DMSO) or treatment (ASR490, DAPT, MG132, or CQ) for prescribed doses and time points. Cell viability assays: Alamar blue (Life Technologies Corporation Eugene, OR) and EdU Cell Proliferation (using the EdU-Click 488 kit, cat# BCK-EdU488-1, Sigma), were then performed per the manufacturer’s instructions.

#### Soft agar colony formation assay

Colony formation assays were performed to monitor anchorage-independent growth using the CytoSelect 96-well *in vitro* Tumor Sensitivity Assay kit (Cell Biolabs Inc., San Diego, CA, United States). The assay was performed as described previously (Suman et al., 2014).

#### Invasion assay

Invasion assays were performed and evaluated using Boyden chambers equipped with polyethylene terephthalate membranes with 8-mm pores (BD Biosciences, San Jose, CA, United States) as described previously (Das et al., 2014).

#### Wound healing migration assay

Control and ASR490-treated ALDH^−^, ALDH^+^, and CD44^+^/CD24^−^ cells were cultured in six-well plates and subjected to wound healing migration assays as described previously (Chandrasekaran et al., 2017).

#### Mammosphere formation assay

The mammosphere formation ability of ALDH^−^, ALDH^+^, and CD44^+^/CD24^−^ cells was determined by culturing the cells with MammoCult basal medium (StemCell Technologies) in ultra-low attachment plates (Corning, Acton, MA, United States). The cells were treated with either vehicle or ASR490 and allowed to form spheroids for 2–3 weeks. After the first-generation mammospheres were counted, the vehicle and ASR490 treated mammospheres were resuspended as single-cell suspensions, measured, and re-cultured without treatment to determine their second-generation mammosphere formation ability. The spheres formed after 3 weeks were counted, resuspended, and cultured for a third generation without treatment to determine their third generation mammosphere formation ability.

#### Immunofluorescence and microscopy

Immunofluorescence assays were performed to determine the Notch1 expression of the vehicle and ASR490-treated first, second, and third-generation spheres as per the protocol described earlier (Suman et al., 2014). The spheres were imaged using a KEYENCE fluorescence microscope (BZ-X800/BZ-X810).

#### Apoptosis assay

Quantification of apoptosis was performed using the Annexin V-FITC apoptosis kit (BD Pharmingen, San Diego, CA, United States). After treatment with vehicle or ASR490 for 24 and 48 h, the ALDH^−^, ALDH^+^, and CD44^+^/CD24^−^ cells were suspended in 500 µL of binding buffer and stained with 5 mL of FITC-Annexin-V and 5 mL of propidium iodide for 15 min in the dark at room-temperature before being analyzed using a flow cytometer (Pal et al., 2018).

#### Transfection

ALDH^+^ cells were transfected as per the protocol described elsewhere (Banks et al., 1985) with 20 nM of scrambled (SCR) or siRNA (siNOTCH1) obtained from OriGene Technologies Inc. (cat#: SR321124). Transfected cells were harvested after 48 h and used for cell proliferation, colony formation, and mammosphere assays following treatment with vehicle or ASR490. ALDH^−^ cells were transfected with 500 ng plasmid concentration of pCMV6-NOTCH1 and vector pCMV6-Entry [NOTCH1 (NM_017617) Human ORF Clone] obtained from OriGene Technologies Inc. The cells were then harvested following treatment with vehicle or ASR490. Whole-cell lysates of transfected ALDH^+^ and ALDH^−^ were also prepared for Western blot analysis following treatment with vehicle or ASR490.

#### Proteasome activity

The proteasome activity of vehicle and ASR490-treated ALDH^+^ cells was measured using a proteasome activity assay kit (BioVision) per the manufacturer’s protocol. MG132 was used as the positive control.

#### RNA isolation, cDNA library construction, and RNA sequencing

Total RNA was isolated from ALDH^+^ and ALDH^−^ cells treated for 6 and 12 h with either vehicle or ASR490 using TRIzol (Thermo Fisher Scientific, Waltham, MA, United States). The quantity and quality of RNA were assessed using a NanoDrop™ spectrophotometer (Fisher Scientific). The cDNA library was prepared according to the manufacturer’s instructions using Novogene Bioinformatics Technologies (Beijing, China) and a NEBNext Ultra TM RNA library kit for Illumina (New England Biolabs, Ipswich, MA, United States) by Novogene Bioinformatics Technologies Co. Ltd. The amplified cDNA library was then purified using the AMPure XP system (Beckman Coulter, Brea, CA, United States). The library quality was evaluated using the Agilent bioanalyzer 2,100 system. Finally, the enriched product was sequenced using the Illumina HiSeq 2,000/2,500 platform, and paired ends were generated. The raw data was analyzed and differential expression analysis was performed using the previously described method (Shukla et al., 2022).

#### Functional enrichment analysis

Gene set enrichment analysis (GSEA) was performed using GSEA software version 4.2.2 (16199517). Volcano plots were generated by SRplot (https://www.bioinformatics.com.cn/en), a free online platform for data analysis and visualization while ShinyGO was used to predict Kyoto Encyclopedia of Genes and Genomes (KEGG) pathways (Ge et al., 2020). Gene ontology (GO) analysis was performed by PANTHER (Mi et al., 2019).

#### Western blotting and immunoprecipitation (IP)

Cell lysates of ALDH^−^, ALDH^+^, and CD44^+^/CD24^−^ cells following treatment with vehicle and treatment (ASR490, DAPT, MG132, CHX, or CQ) for prescribed doses and time points, were prepared with RIPA buffer (Thermo Scientific, Rockford, IL, United States) per the manufacturer’s protocol. Western blotting was performed using specific antibodies against Notch1 (CST, #3608), HES1 (Sigma, #SAB2108472), Hey1 (Proteintech, #19929-1-AP), NFκB p65 (CST, #8242), Bcl-2 (CST, #15071), Bcl-xL (CST, #2764), Vimentin (CST, #46173), Slug (CST, #9585), E-Cadherin (CST, #3195), β-catenin (CST, #8480), Ubiquitin (CST, #3933), Cleaved-PARP (CST, #5625), Cleaved-caspase-9 (CST, #20750), BAX (CST, #41162), Notch2 (CST, #D76A6), Lamp1 (CST, #9091), and LC3B (Proteintech, #14600-1-AP). β-Actin (CST, #4970) was used as the loading control. Protein bands were visualized using the Bio-Rad ChemiDocTM imaging system. For IP experiments, protein samples were immunoprecipitated with Notch1 antibody as per the protocol described elsewhere (Chandrasekaran et al., 2020), and Western blots were performed with ubiquitin antibody.

#### Xenograft studies

The *in vivo* efficiency of ASR490 in abrogating ALDH^+^ and ALDH^−^ tumorigenesis was evaluated by subcutaneously injecting each cell type (ALDH^+^: 0.5 × 10^6^ cells and ALDH^−^: 0.8 × 10^6^ cells) into female athymic mice (The Jackson Laboratory). ASR490 was dissolved in DMSO and then diluted in PBS by sonication to create a 0.1% solution. Once the tumors were approximately ∼50 mm^3^, the mice bearing ALDH^+^ and ALDH^−^ tumors were randomized into the vehicle (0.1% DMSO in PBS) and treatment (25 mg/kg, ASR490) groups (*n* = 6). ASR490 was administered orally for 7 days over 4 weeks. The mice were monitored daily, and tumor volumes and body weight were measured weekly. After 4 weeks of treatment, all mice were euthanized by CO_2_ asphyxiation, and the xenograft tumors were collected for immunohistochemical analyses. All experimental animals were approved by the University of Louisville’s ethical committee and maintained by the Institutional Animal Care and Use Committee-approved protocols.

#### Immunohistochemistry (IHC) analysis

Tumor specimens from the vehicle and ASR490-treated ALDH^+^ and ALDH^−^ xenografts were fixed in 10% formalin and processed for IHC analysis per the protocol previously described (Chandrasekaran et al., 2020). IHC analyses were performed for Notch1, HES1, and Ki67 expression, and images (40x) were captured using an Olympus BX43 microscope (Olympus America, Center Valley, PA).

#### Statistical analysis

Statistical analyses were performed using GraphPad Prism 8.0 software (GraphPad Software Inc., La Jolla, CA, United States). Values were presented as mean ± SD. One-way or two-way ANOVA and unpaired two-tailed Student's *t*-tests were performed to determine the significance between groups. A *p*-value of < 0.05 was considered statistically significant.

## Results

### ASR490 inhibits the growth of BCSCs and BC

**FIGURE 1:**
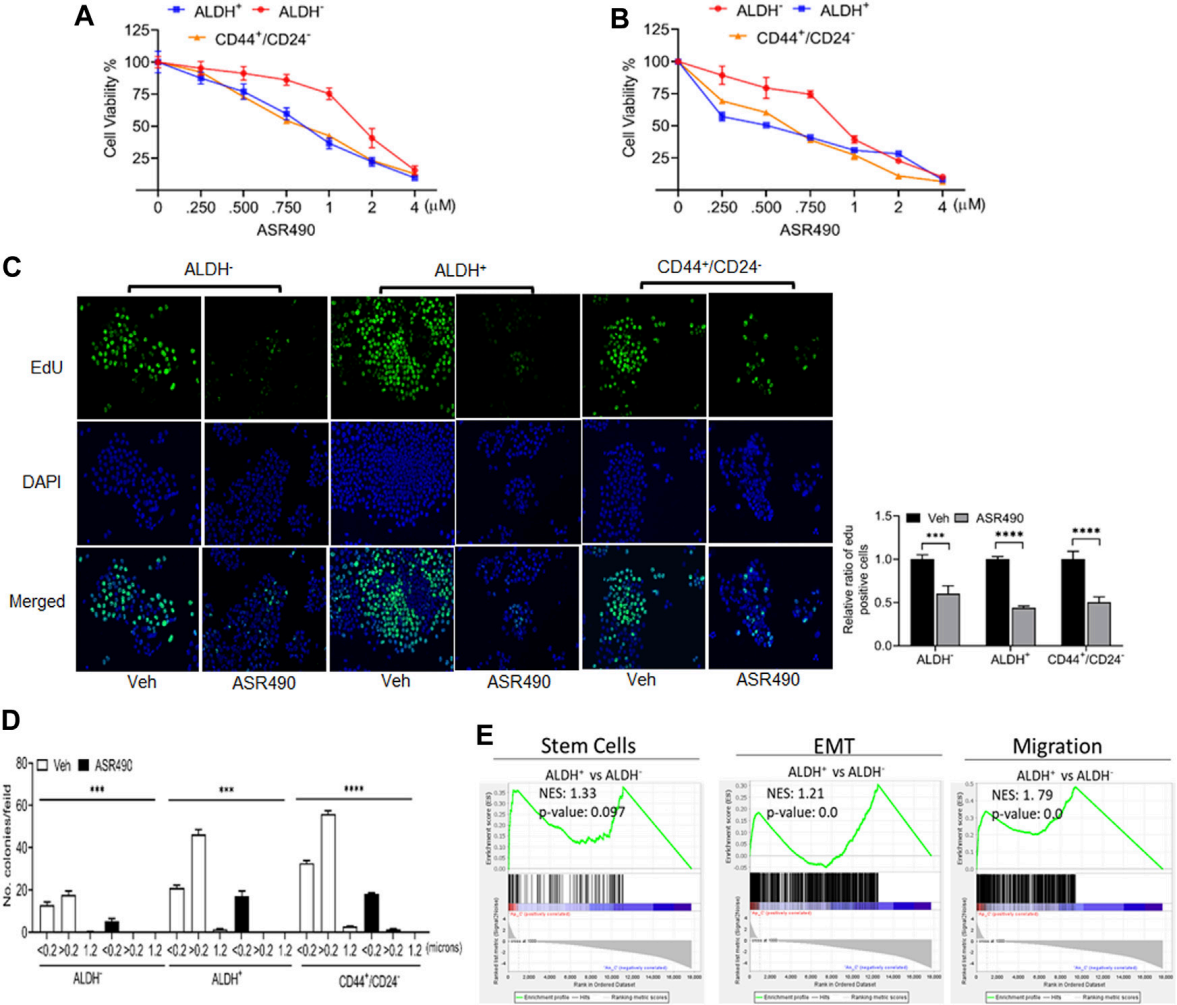
ASR490 suppresses the growth of ALDH^−^, ALDH^+^, and CD44^+^/CD24^−^ **(A, B)**. MTT cell viability assays performed on ALDH^−^, ALDH^+^, and CD44^+^/CD24^−^ cells following treatment with various concentrations of ASR490 (0, 0.250, 0.500, 0.750, 1, 2, and 4 µM) for 24 **(A)** and 48 h **(B)** (*n* = 6). **(C)** Representative images of EdU cell proliferation assay performed on vehicle and ASR490-treated ALDH^−^, ALDH^+^, and CD44^+^/CD24^−^ cells. Data were quantified by counting the cells demonstrating EdU (5-ethynyl-2′-deoxyuridine) expression per treatment group for each cell type using Image J software (*n* = 4, *** *p* < 0.001 and **** *p* < 0.0001). *p* values are based on two-way ANOVA with the *post hoc* Sidak test. **(D)** Colony-forming assay of the vehicle and ASR490-treated ALDH^−^, ALDH^+^, and CD44^+^/CD24^−^ cells (*n* = 6; *** *p* < 0.006 and **** *p* < 0.0001). *p* values are based on one-way ANOVA with a *post hoc* LSD test. **(E)** GSEA analyses of RNAseq data showing enrichment of genes in stem cell, EMT, metastasis signaling pathways in ALDH^+^ vs. ALDH^−^ cells (*n* = 4).

ALDH^+^ and CD44^+^/CD24^−^ are reported to be the top two markers enriched in BCSC populations (Douville et al., 2009; Charafe-Jauffret et al., 2010) which exhibit elevated Notch1 activity, and consequently, higher tumorigenic capability (D'Angelo et al., 2015; Ginestier et al., 2007). To determine ASR490’s inhibitory function, we performed two cell proliferation assays on the ALDH^−^, ALDH^+^, and CD44^+^/CD24^−^ cells. Cell viability assays demonstrated that ASR490 effectively inhibited the viability of all three cell types, though its effects on Notch1-positive BCSCs (ALDH^+^: IC50: 770 nM at 24 h, and 443 nM at 48 h; and CD44^+^/CD24^−^: IC50: 800 nM at 24 h, and 541 nM at 48 h) was more profound than its effect on ALDH^−^ BC cells (IC50: 1.6 μM at 24 h and 836 nM at 48 h) (Figures 1A, B). Similar results were confirmed by EdU-proliferation analyses on all three cell types (Figure 1C). Next, we determined if ASR490 could inhibit the anchorage-independent growth of ALDH^−^, ALDH^+^, and CD44^+^/CD24^−^ cells. As seen in Figure 1D, vehicle-treated ALDH^+^ and CD44^+^/CD24^−^ cells showed a higher number of <0.2, >0.2, and 1.2 micron-sized colonies compared to vehicle-treated ALDH^−^ cells; however, treatment with ASR490 significantly suppressed the colony-formation ability of ALDH^+^ and CD44^+^/CD24^−^ cells as compared to their respective vehicle-treated controls. Similar results were seen in ALDH^−^ BC cells.

Next, we performed transcriptomic analysis to determine the possible mechanistic action of ASR490 on the stem cells. GSEA of vehicle control treated ALDH^+^ and ALDH^−^ cells revealed a significant enrichment of genes involved in the mammary stem cells and their related signaling pathways (epithelial-mesenchymal transition (EMT) and migration) in the ALDH^+^ cells compared to the ALDH^−^ cells. (Figure 1E). In contrast, ASR490-treated ALDH^+^ cells showed significant downregulation of stem cell signaling compared to their ALDH^−^ counterparts (Figure 1E). We also performed a volcano plot-based filtering analysis to identify genes with significantly differential expression among these groups (Supplementary Figure S1). Further, KEGG analysis revealed that ASR490 inhibited Notch-mediated proliferation and EMT signaling in ALDH^+^ cells (Supplementary Figure S2).

### ASR490 specifically inhibits Notch1-NICD signaling in ALDH^+^ and CD44^+^/CD24^−^ cells

**FIGURE 2:**
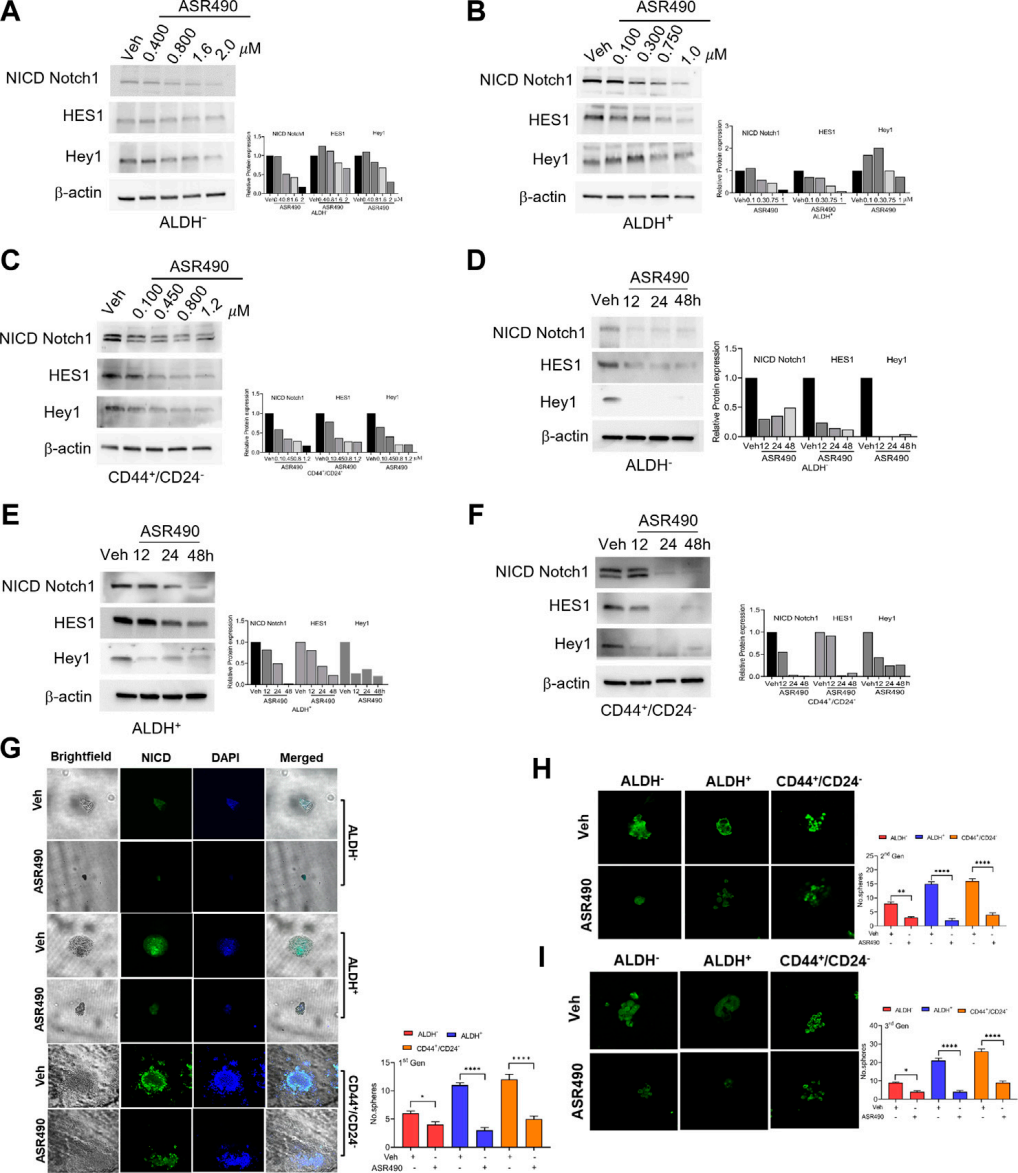
ASR490 mediates its effect by specifically inhibiting Notch1-NICD signaling and self-renewal capacity. **(A–F)** Western blots for Notch1-NICD (the active form of Notch1), HES1, and Hey1 expression to assess the dose- **(A–C)** and time-dependent **(D–F)** effects of ASR490 on ALDH^−^, ALDH^+^, and CD44^+^/CD24^−^ cells. **(G)** Representative brightfield and immunofluorescent images of Notch1-NICD (active form of Notch1) expression in vehicle and ASR490-treated first-generation mammospheres of ALDH^−^, ALDH^+^ and CD44^+^/CD24^−^ cells. The number of spheroids per group and cell type were quantified and presented graphically (*n* = 4, * *p* < 0.02 and **** *p* < 0.0001). **(H)** Immunofluorescent images of Notch1-NICD expression and number of spheroids quantified for second generation of serially passaged first-generation vehicle and ASR490-treated ALDH^−^, ALDH^+^, and CD44^+^/CD24^−^ mammospheres (*n* = 4, ** *p* < 0.007 and **** *p* < 0.0001), **(I)** Third generation of serially passaged second-generation spheroids. (*n* = 4, *, *p* < 0.02 and ****, *p* < 0.0001). Second and third-generation cells were not subjected to ASR490 treatment. *p* values are based on one-way ANOVA with *post hoc* Dunnett’s test.

Based on the GSEA analyses, we first validated the Notch1 targets in ASR490-treated ALDH^−^, ALDH^+^, and CD44^+^/CD24^−^ cells. Figures 2A–F show a dose and time-dependent downregulation of Notch1-NICD (the active form of Notch1) and its downstream effectors HES1 and Hey1 in all three cell types. Interestingly, the basal expression of Notch-1 in ALDH^+^ and CD44^+^/CD24^−^ BCSCs was significantly higher when compared to the ALDH^−^ BC cells. As one of stem cell characteristics is the formation of mammospheres, we next determined whether inhibition of Notch1 affected the self-renewal properties of the BCSCs. Both vehicle-treated ALDH^+^ and CD44^+^/CD24^−^ cells significantly developed more prominent and numerous spheroids than their ALDH^−^ counterparts (Figure 2G). As expected, ASR490 treatment resulted in a dramatic decrease in the number and size of spheroids in all three cell types, albeit its effect was significantly more profound in the ALDH^+^ and CD44^+^/CD24^−^ cells. We then performed immunofluorescence for Notch1 expression in vehicle and ASR490-treated spheres to determine whether ASR490’s inhibitory effects were mediated *via* its suppression of Notch1 expression in these cells. As expected, vehicle treated BCSCs (ALDH^+^ and CD44^+^/CD24^−^) were found to express significantly higher Notch1 expression as compared to ALDH^−^ BC cells, and the significantly decreased size and number of spheroids in the treatment group was found to be directly correlated with ASR490’s inhibition of Notch1 in all three cell types (Figure 2G).

To evaluate the potency of ASR490, these first-generation mammospheres were passaged for two more consecutive generations, though the second and third-generation spheroids were not exposed to ASR490 treatment. Analyses of the second and third-generation mammospheres revealed that ASR490 seemed to exert a prolonged effect on these cells, i.e., despite the two latter generations not being subjected to additional ASR490 treatment, the spheroids formed by the treatment groups continued to express significantly lower levels of Notch1, and consequently, formed smaller spheroids than their predecessors (Figures 2H, I; Supplementary Figure S3). This was in complete contrast to the vehicle-treated cells, which showed elevated Notch1 expression and larger spheroids.

**FIGURE 3:**
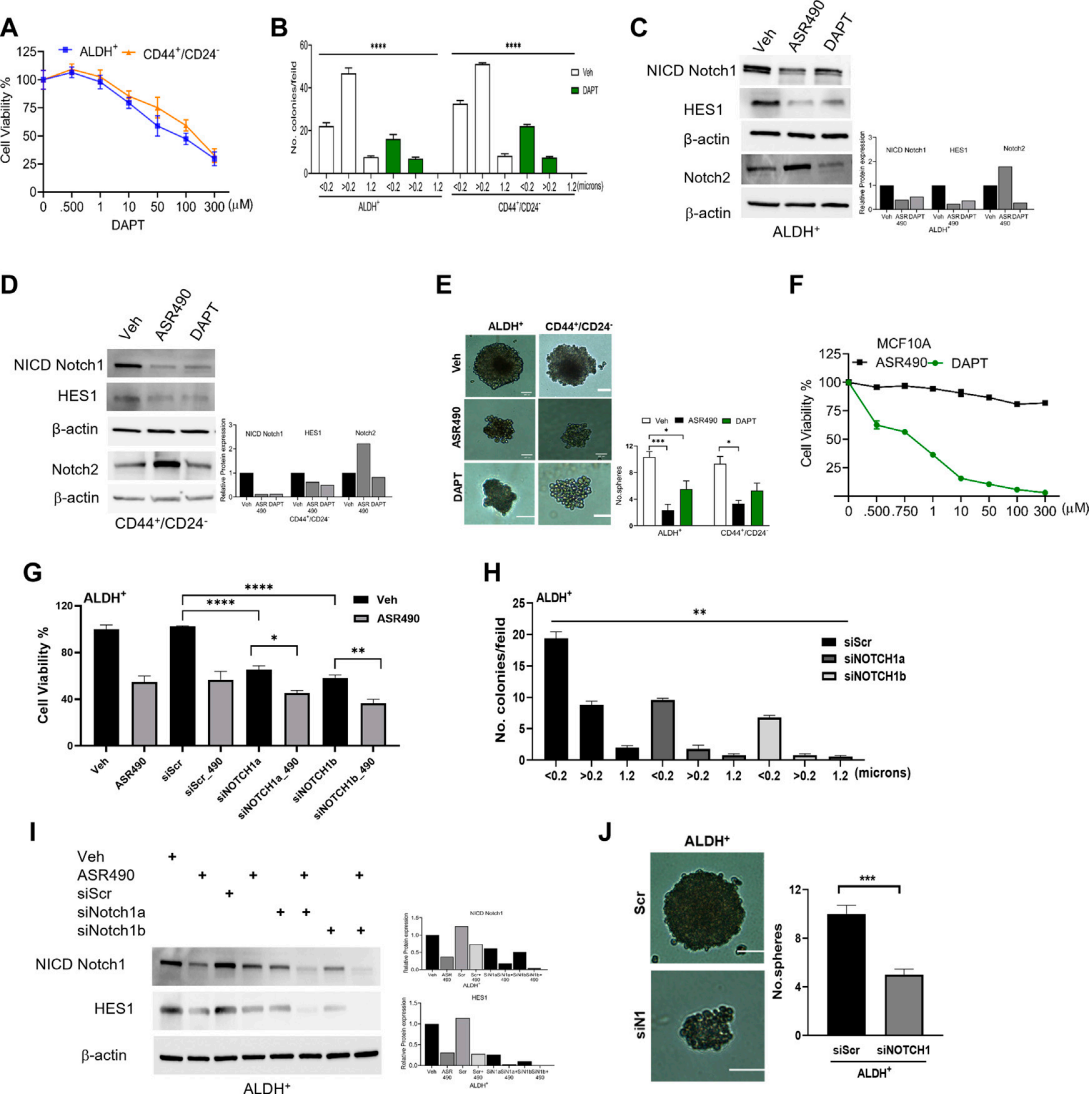
Pharmacological and genetic approaches to inhibit Notch1 signaling in BCSCs. **(A)** MTT cell viability assay performed on ALDH^+^ and CD44^+^/CD24^−^ cells following treatment with various concentrations of DAPT (0, 0.500, 1, 10, 100, and 300 µM) for 24 h (*n* = 6). **(B)** Colony-forming assay of vehicle and DAPT-treated ALDH^+^ and CD44^+^/CD24^−^ cells (*n* = 6; **** *p* < 0.0001). **(C, D)** Western blots for Notch1-NICD (the active form of Notch1), HES1 and Hey1 and Notch2 of vehicle, ASR490 and DAPT-treated ALDH^+^ **(C)** and CD44^+^/CD24^−^ **(D)** cells. **(E)** Mammosphere assay performed for vehicle, ASR490, and DAPT-treated ALDH^+^ and CD44^+^/CD24^−^ cells (*n* = 4, * *p* < 0.02 and *** *p* < 0.007). *p* values are based on one-way ANOVA with a *post hoc* LSD test. **(F)** MTT cell viability assay performed on MCF10A cells following treatment with various concentrations (0, 0.500, 0.750, 1, 10, 50, 100, and 300 µM) of ASR490 and DAPT for 24 h (*n* = 6). **(G)** Cell viability and **(H)** colony forming assays for vehicle and ASR490 treated non-transfected, SCR-siRNA, and Notch1-siRNA transfected ALDH^+^ cells for 24 h (*n* = 6; * *p* < 0.01, ** *p* < 0.008, and **** *p* < 0.0001). *p* values are based on one-way ANOVA with a *post hoc* LSD test. **(I)** Western blots for Notch-NICD and HES-1 expression were performed on vehicle or ASR490 treated SCR-siRNA, and Notch1-siRNA transfected ALDH^+^ cells. **(J)** Mammospheres assay performed for vehicle and ASR490 treated SCR-siRNA, and Notch1-siRNA transfected ALDH^+^ cells (*n* = 6; *** *p* < 0.001). *p* values are based on one-way ANOVA with a *post hoc* LSD test.

To confirm ASR490’s specificity in targeting Notch1, we compared its efficacy with a known pharmacological Notch inhibitor, DAPT (a γ-secretase), and by genetic inhibition (using siRNA for Notch1). Cell viability assays demonstrated that DAPT inhibited the growth of ALDH^+^ and CD44^+^/CD24^−^ in the μM range (IC_50_: ALDH^+^: 81 µM and CD44^+^/CD24^−^:135 µM), underscoring the higher comparative potency of ASR490 in nM concentrations against Notch1-dependent cell growth (Figure 3A). DAPT was also found to inhibit the colony-forming abilities of ALDH^+^ and CD44^+^/CD24^−^ cells (Figure 3B). Subsequent immunoblotting analyses revealed that in contrast to DAPT, ASR490 specifically only inhibited Notch1 and its downstream effector, HES-1, and did not affect the expression of Notch2 in ALDH^+^ and CD44^+^/CD24^−^ (Figures 3C, D). Further, head-to-head comparison of ASR490 and DAPT in mammospheres assays demonstrated that ASR490 was more effective than DAPT at curtailing the self-renewal capacity of the BCSCs (Figure 3E).

Finally, the IC_50_ concentration at which DAPT mediated its inhibitory effect on BCSCs was found to be toxic to healthy breast epithelial cells (MCF10A) as compared with ASR490 (Figure 3F).

Similar approaches were executed using SCR and Notch1 siRNA transfected ALDH^+^ to examine their cell viability, Notch1(NICD) and HES1 protein expressions, as well as colony- and mammosphere formation abilities following treatment with vehicle or ASR490. Results demonstrated that siNOTCH1-transfected cells showed significantly reduced cell viability and colony-forming abilities as compared to SCR-siRNA transfected ALDH^+^ cells. Interestingly, treatment with ASR490 further inhibited the growth of the siNOTCH1-transfected ALDH^+^ cells (Figures 3G, H). Similarly, while siNOTCH1-transfected ALDH^+^ cells demonstrated decreased Notch1-NICD and HES1 expression levels compared to SCR-siRNA-transfected cells (Figure 3I), ASR490 treatment further inhibited Notch1 signaling in these cells. Finally, mammosphere assays demonstrated that the spheroids formed by siNOTCH1-transfected cells were smaller than those of scrambled transfected cells (Figure 3J), suggesting that Notch1 is an essential target for inhibiting BCSC growth.

### Inhibition of Notch1 facilitates autophagy-mediated cell death in ASR490-treated BCSCs

**FIGURE 4:**
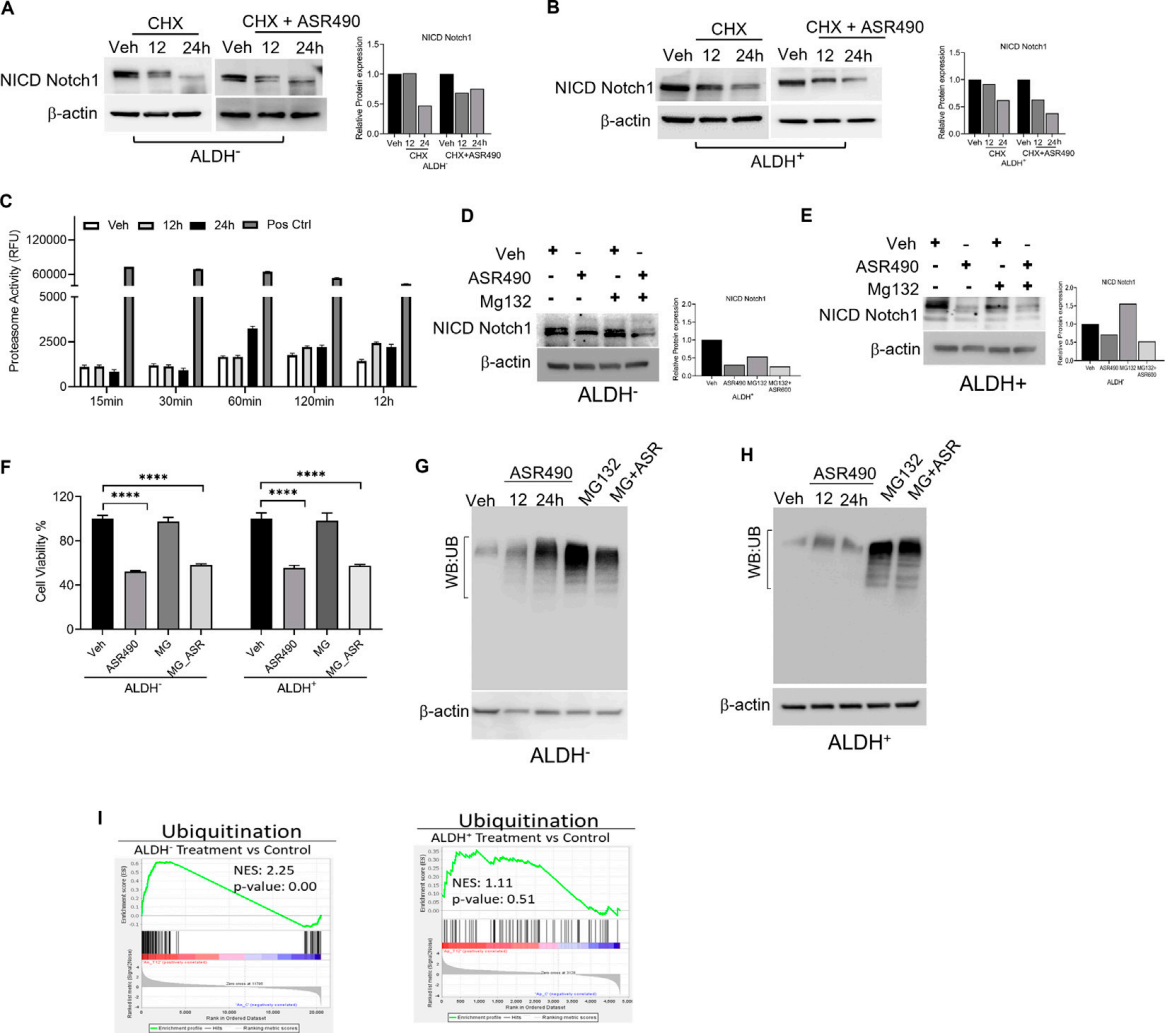
Molecular signaling responsible for NICD downregulation in ASR490 treated in ALDH^−^ and ALDH^+^ cells. **(A, B)** Western blots performed for Notch1-NICD expression in ALDH^−^ and ALDH^+^ cells treated with ASR490, CHX (50 µM), or combinations at the indicated time points. **(C)** Proteasome activity measured for ALDH^+^ cells treated with ASR490 at the indicated time points. **(D, E)** Western blots showing Notch1-NICD expression in ALDH^−^ and ALDH^+^ cells treated with ASR490, MG132 (10 µM), or combinations at the indicated time points. **(F)** MTT cell viability assays performed on ALDH^−^ and ALDH^+^ following treatment with ASR490, MG132, or combinations (*n* = 6; **** *p* < 0.0001). *p* values are based on one-way ANOVA with a *post hoc* LSD test. **(G, H)** Western blots for ubiquitin protein expression performed for ASR490-treated ALDH^−^ and ALDH^+^ cells. MG132 was used as a positive control. **(I)** GSEA of RNA-seq data demonstrating alterations in ubiquitin signaling in ASR490-treated ALDH^+^ and ALDH^−^ cells.

Interestingly, in this study, no significant changes in Notch1 expression was observed from our transcriptomic analysis of ASR490-treated BCSCs (data not shown). So, we postulated that ASR490 might post-translationally regulate Notch1 in BCSCs. To corroborate this, ALDH^+^ and ALDH^−^ cells were treated with protein synthesis inhibitor CHX in the presence and absence of ASR490. While reduced Notch-NICD expression was observed in both ALDH^+^ and ALDH^−^ cells at 24 h in the CHX-alone treated group, the combination of ASR490 + CHX resulted in significantly greater suppression of Notch-NICD levels in both cell types (Figures 4A, B), suggesting that ASR490 may mediate its inhibitory effects through Notch1-NICD degradation.

As several studies have reported that Notch1 degradation can occur either *via* the ubiquitin-proteasome or lysosome pathways (Jehn et al., 2002; McGill and McGlade, 2003; Ahn et al., 2016; Wu et al., 2016). We first analyzed the possibility of ASR490 being another proteasome inhibitor like MG132 by measuring proteasome activity using a chymotrypsin-like compound with a 7-amido-4-methyl coumarin (AMC)-tagged peptide substrate induction of proteasome activity in ALDH^+^ cells. Commercially available positive and negative controls were used for these experiments. Induction of proteasome activity was measured at different time points, and no significant changes were noted until 12 h in ASR490-treated ALDH^+^ cells (Figure 4C). This suggests that ASR490 was not a proteasomal inhibitor. To further verify whether ASR490 mediates its effects *via* the proteasomal pathway, we performed cell viability and immunoblot analyses of vehicle and ASR490 treated ALDH^+^ and ALDH^−^ in the presence and absence of MG132 (10 µM). Results demonstrated that MG132 could not rescue ASR490-mediated inhibition of Notch1-NICD expression in both cell types (Figures 4D–F). Further, we analyzed the ubiquitin expression in ASR490-treated cells and found no significant induction of ubiquitin in either cell type following 12 and 24 h of treatment with ASR490 (Figures 4G, H). GSEA analysis of ASR490-treated cells demonstrated that ASR490 downregulated ubiquitin signaling in ALDH^−^ cells but not in ALDH^+^ cells (Figure 4I; Supplementary Figure S4).

**FIGURE 5:**
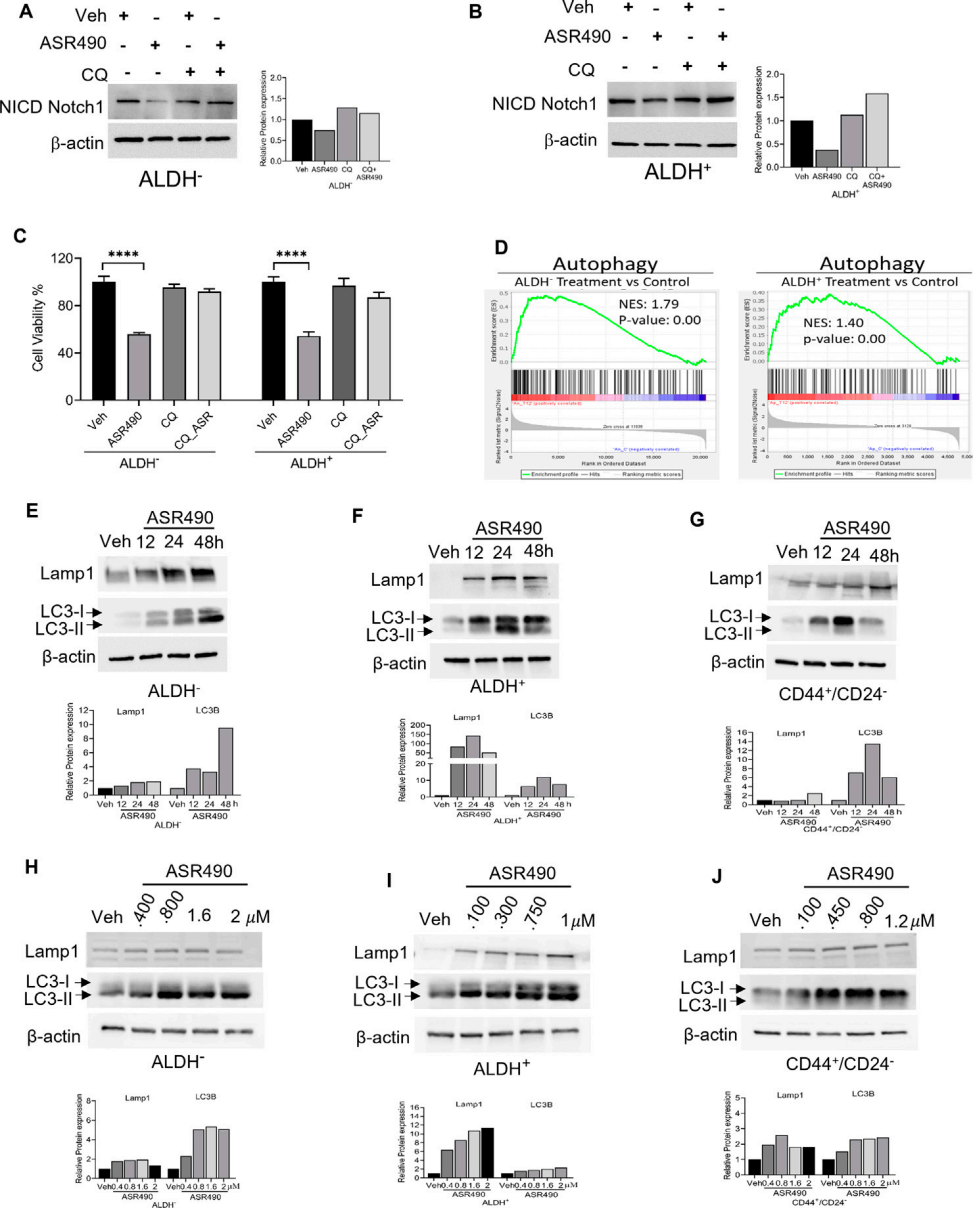
ASR490 abrogates both breast cancer and BCSC growth by activating autophagy pathways. **(A, B)** Western blots for Notch1-NICD expression in ALDH^−^ and ALDH^+^ cells treated with ASR490, CQ (50 µM), or combinations at the indicated time points. **(C)** MTT cell viability assays performed on ALDH^−^ and ALDH^+^ following treatment with ASR490, CQ, or combinations. (*n* = 6; **** *p* < 0.0001). *p* values are based on one-way ANOVA with the *post hoc* Tukey test. **(D)** GSEA of RNA-seq data demonstrating downregulation in autophagy-regulated genes in ASR490-treated ALDH^+^ and ALDH^−^ cells. **(E–J)**. Western blots showing time- **(E–G)** and dose-dependent **(H–J)** effects of ASR490 treatment on autophagy markers Lamp1 and LC3B in ALDH^−^, ALDH^+^, and CD44^+^/CD24^−^ cells.

**FIGURE 6:**
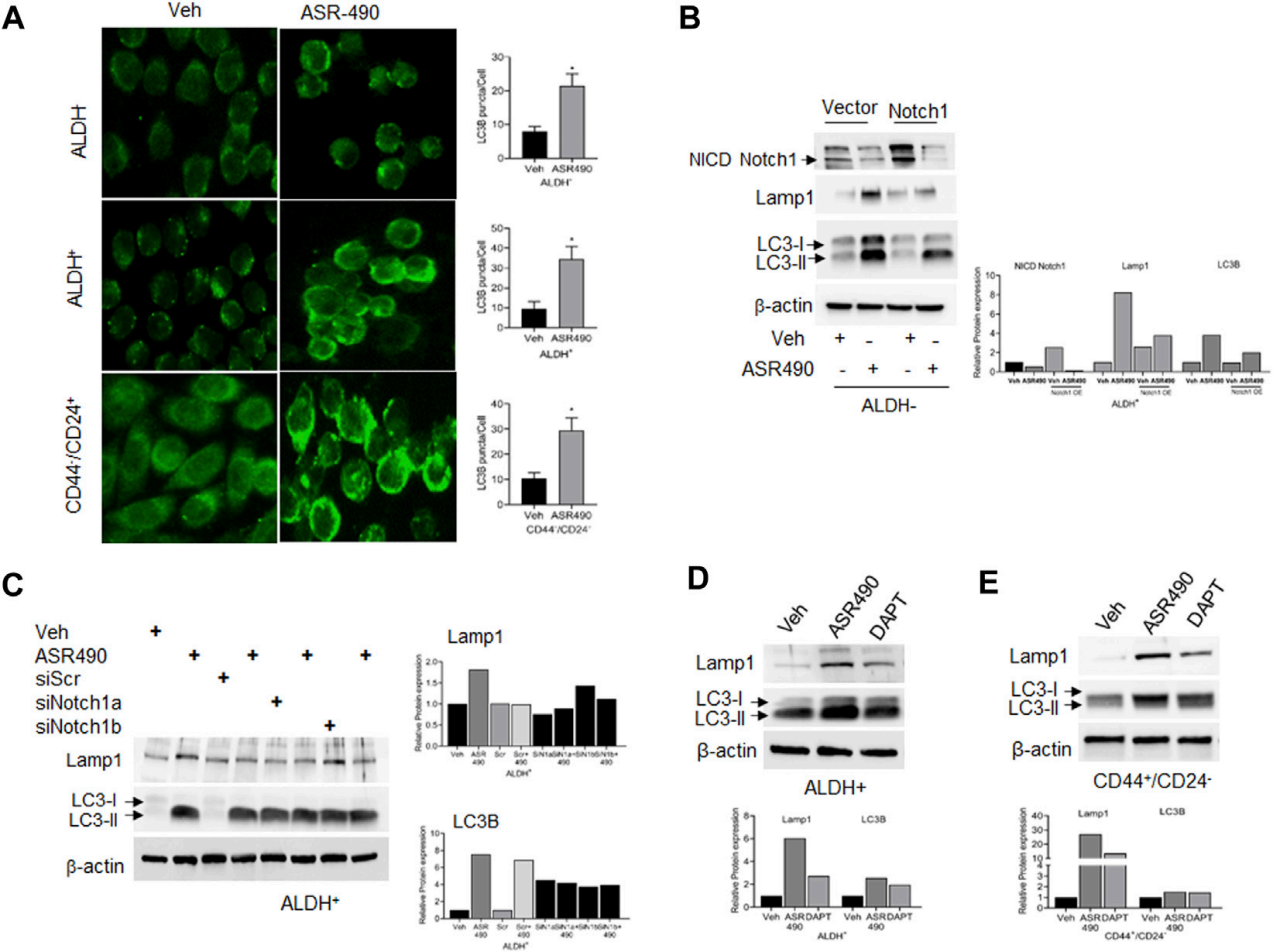
Notch-1 regulates autophagy in BCSC and BC cells. **(A)** Representative images of immunofluorescence analyses for LC3B punctae in vehicle and ASR490 treated ALDH^−^, ALDH^+^, and CD44^+^/CD24^−^ cells. **(B, C)** Western blots for Lamp1 and LC3B expression in vehicle and ASR490-treated pCMV- and Notch1-transfected ALDH^−^ cells **(B)** and SCR-siRNA, and Notch1-siRNA transfected ALDH^+^ cells **(C)**. **(D,E)** Western blots analysis of Notch1-NICD, Lamp-1, and LC3B for vehicle, ASR490, and DAPT-treated ALDH^+^ and CD44^+^/CD24^−^ cells.

In contrast, Western blots for Notch1-NICD and viability assays demonstrated that treatment with lysosome inhibitor (CQ) rescued NICD expression in ASR490 treated ALDH^+^ and ALDH^−^ cells, signifying the possible involvement of lysosome signaling in ASR490-mediated NICD degradation (Figures 5A–C). GSEA analysis suggested significant enrichment of autophagy regulatory genes in ASR490-treated ALDH^+^ and ALDH^−^ cells (Figure 5D). This was further corroborated by Western blots that demonstrated a significant time- and dose-dependent upregulation of autophagy markers Lamp1 and LC3B in ASR490-treated BCSCs and BC cells (Figures 5E–J). Immunofluorescence analysis showed a significant accumulation of LC3B puncta, indicative of autophagy involvement, in ASR490 treated BCSCs and BC cells (Figure 6A). To demonstrate that Notch1 mediates autophagy signaling, we first overexpressed Notch1 in ALDH^−^ cells, inhibiting endogenous and ASR490-induced LAMP1 and LC3B expressions (Figure 6B), suggesting that Notch1 activation impairs autophagy signaling in BC cells. Next, to confirm that Notch1 regulates autophagy signaling, we silenced Notch1 expression using siRNA Notch1 in ALDH^+^ cells and showed that inhibition of Notch1 reverts both LAMP1 and LC3B expression (Figure 6C). Inhibition of Notch1 using pharmacological Notch1 inhibitors also facilitated the induction of autophagy markers in BCSCs (Figures 6D, E), confirming the molecular interaction between Notch1 and autophagy signaling in BCSCs.

### ASR490 inhibits the proliferation, invasive and migratory abilities of BCSCs and BC

**FIGURE 7:**
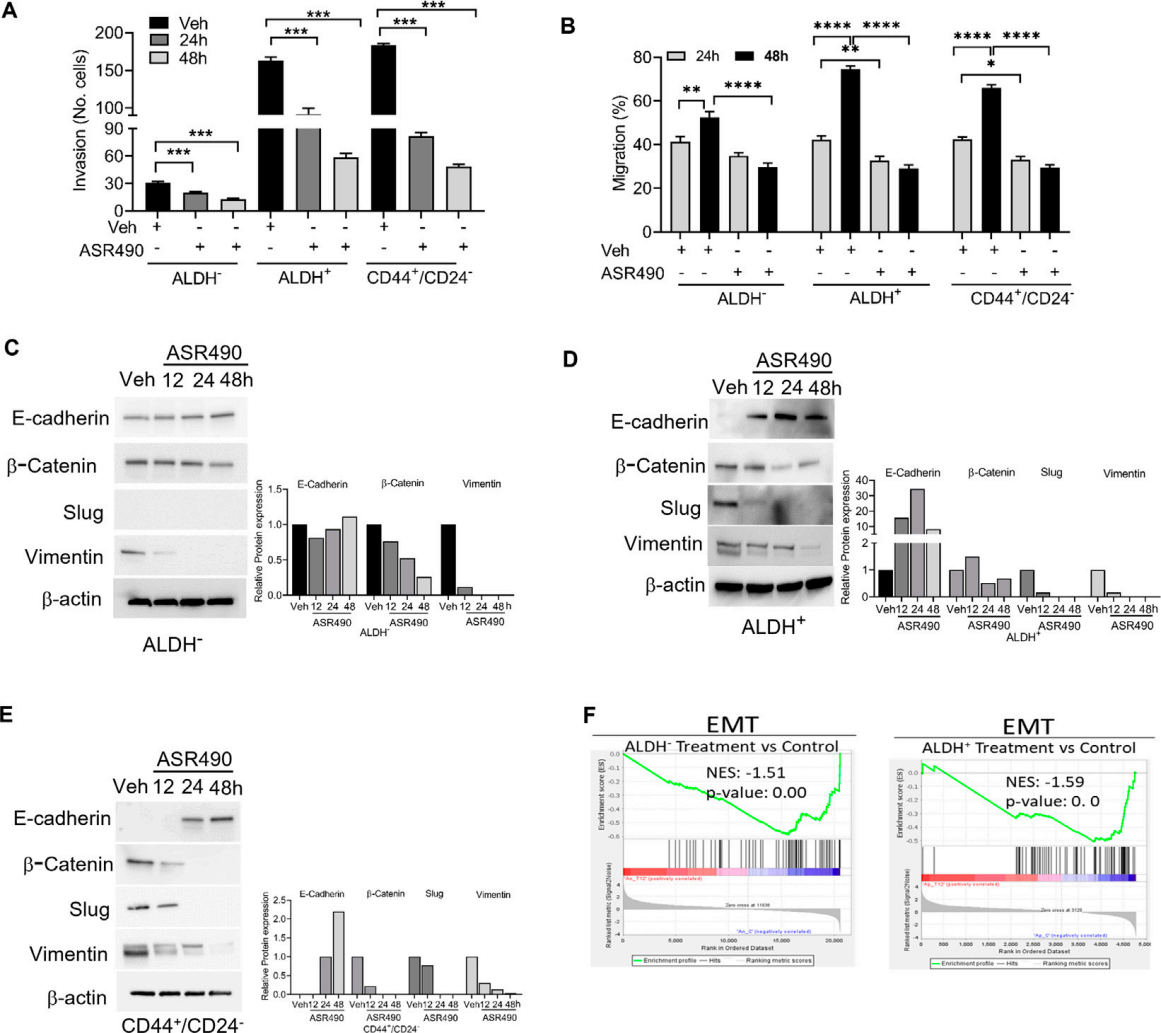
ASR490 inhibits the invasive and migratory abilities of BC and BCSCs. **(A)** Boyden chamber invasion assay was used to assess the time-dependent effects of ASR490 on the invasive ability of ALDH^−^, ALDH^+^, and CD44^+^/CD24^−^ cells (*n* = 3, **** *p* < 0.0001). **(B)** Time-dependent effects of ASR490 on the migratory abilities of ALDH^−^, ALDH^+^ and CD44^+^/CD24^−^ cells were assessed using a scratch wound assay (*n* = 3, * *p* < 0.03, ** *p* < 0.007 and **** *p* < 0.0001). *p* values are based on one-way ANOVA with *post hoc* Tukey’s test. **(C–E)** Western blots showing time-dependent effects of ASR490 treatment on EMT markers (E-Cadherin, β-Catenin, Slug, and Vimentin) in ALDH^−^, ALDH^+^, and CD44^+^/CD24^−^ cells. **(F)** GSEA of RNA-seq data demonstrating downregulation of EMT-regulated genes in ASR490-treated ALDH^−^ and ALDH^+^ cells.

Invasion and wound healing migration assays demonstrated that treatment with ASR490 significantly inhibited the invasive and migratory potential of both BCSCs and BC cells (Figures 7A, B), albeit ASR490’s effect was more profound on the BCSCs (ALDH^+^ and CD44^+^/CD22^−^ than the BC (ALDH^−^) cells. In addition, assessment of the expressions of essential EMT genes demonstrated a time-dependent increase of epithelial marker E-cadherin and decrease of mesenchymal markers β-catenin, Slug, and Vimentin in ASR490 treated BCSCs and BC cells (Figures 7C–E). This was further corroborated by GSEA results which demonstrated a significant decline in EMT-enriched genes in ASR490-treated cells compared to the enrichment of EMT and metastasis genes in control cells (Figure 7F).

**FIGURE 8:**
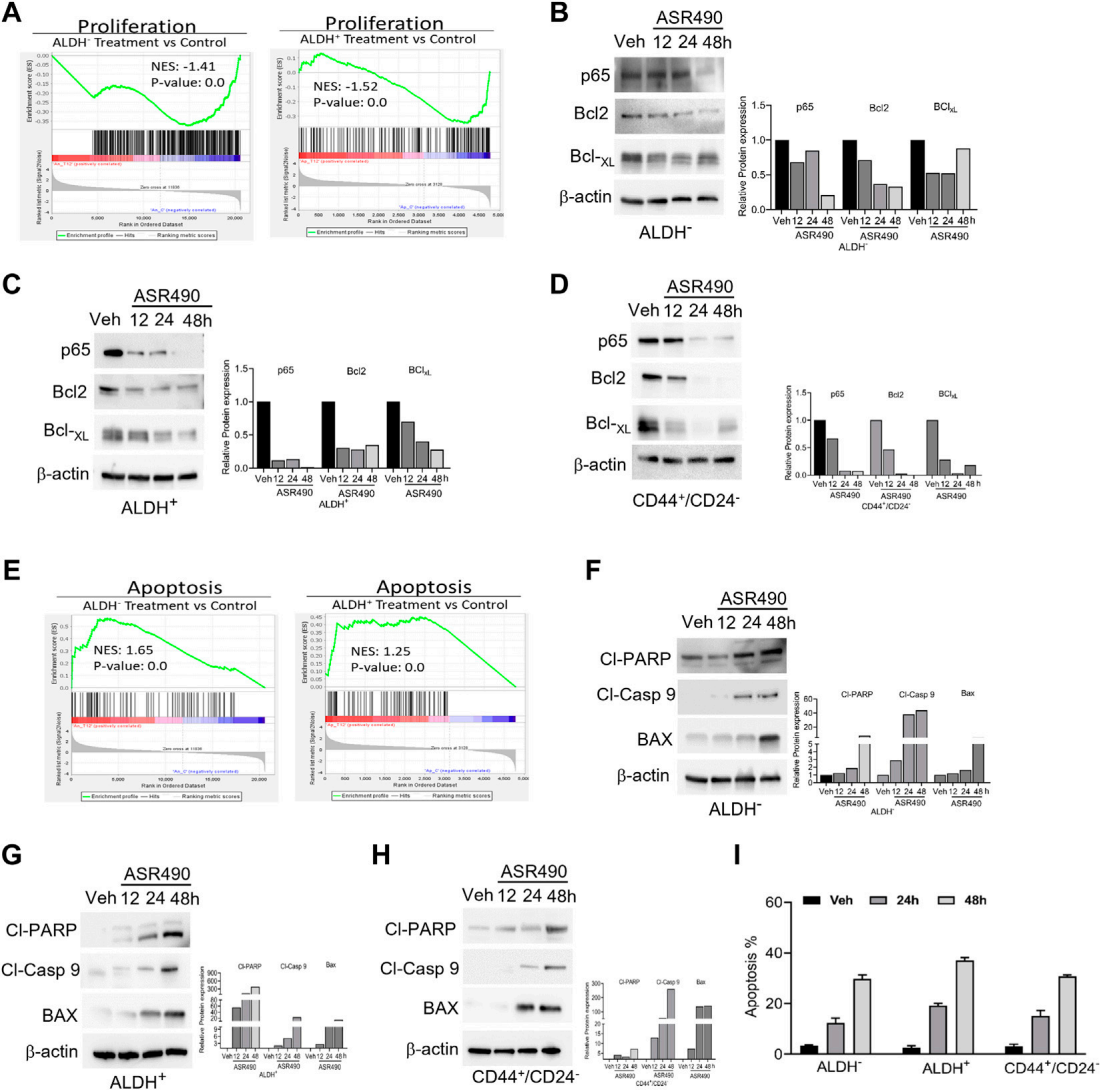
ASR490 inhibits the pro-survival signaling of BCSCs. **(A)** GSEA of RNA-seq data demonstrating alterations in proliferation signaling pathways in ASR490-treated ALDH^−^ and ALDH^+^ cells. **(B–D)** Western blots showing time-dependent effects of ASR490 treatment on pro-survival markers NFkB p65, Bcl2, and Bcl-_XL_ expression in ALDH^−^, ALDH^+^, and CD44^+^/CD24^−^ cells. **(E)** GSEA of RNA-seq data demonstrating alterations in proapoptotic signaling in ASR490-treated ALDH^−^ and ALDH^+^. **(F–H)**. Western blots showing time-dependent effects of ASR490 treatment on the proapoptotic markers Cleaved-PARP, Cleaved-Caspase 9, and BAX in ALDH^−^, ALDH^+^, and CD44^+^/CD24^−^ cells. **(I)** FACS analyses (Annexin V–FITC and PI staining) performed for vehicle and ASR490-treated ALDH^−^, ALDH^+^, and CD44^+^/CD24^−^ cells.

GSEA analyses also revealed that treatment with ASR490 also curbed the proliferative ability of both BCSCs and BC cells as evident by the decreased enrichment of proliferation genes in treated cells vs. control cells (Figure 8A). This was also confirmed by Western blots demonstrating a time-dependent downregulation of pro-survival genes (NFκB [p65], Bcl2, and BCL-xL) in ASR490-treated BCSCs and BC cells (Figures 8B–D). While both GSEA analysis (Figure 8E) and Western blots (Figures 8F–H) for apoptotic markers (Cleaved Caspase-9, cleaved-PARP, and BAX) demonstrated a time-dependent upregulation of apoptosis in ASR490 treated cells. Subsequent FACS analysis revealed that this induction was not significant (Figure 8I).

### ASR490 abrogates *in vivo* growth of both ALDH^+^ and ALDH^−^ tumors

**FIGURE 9:**
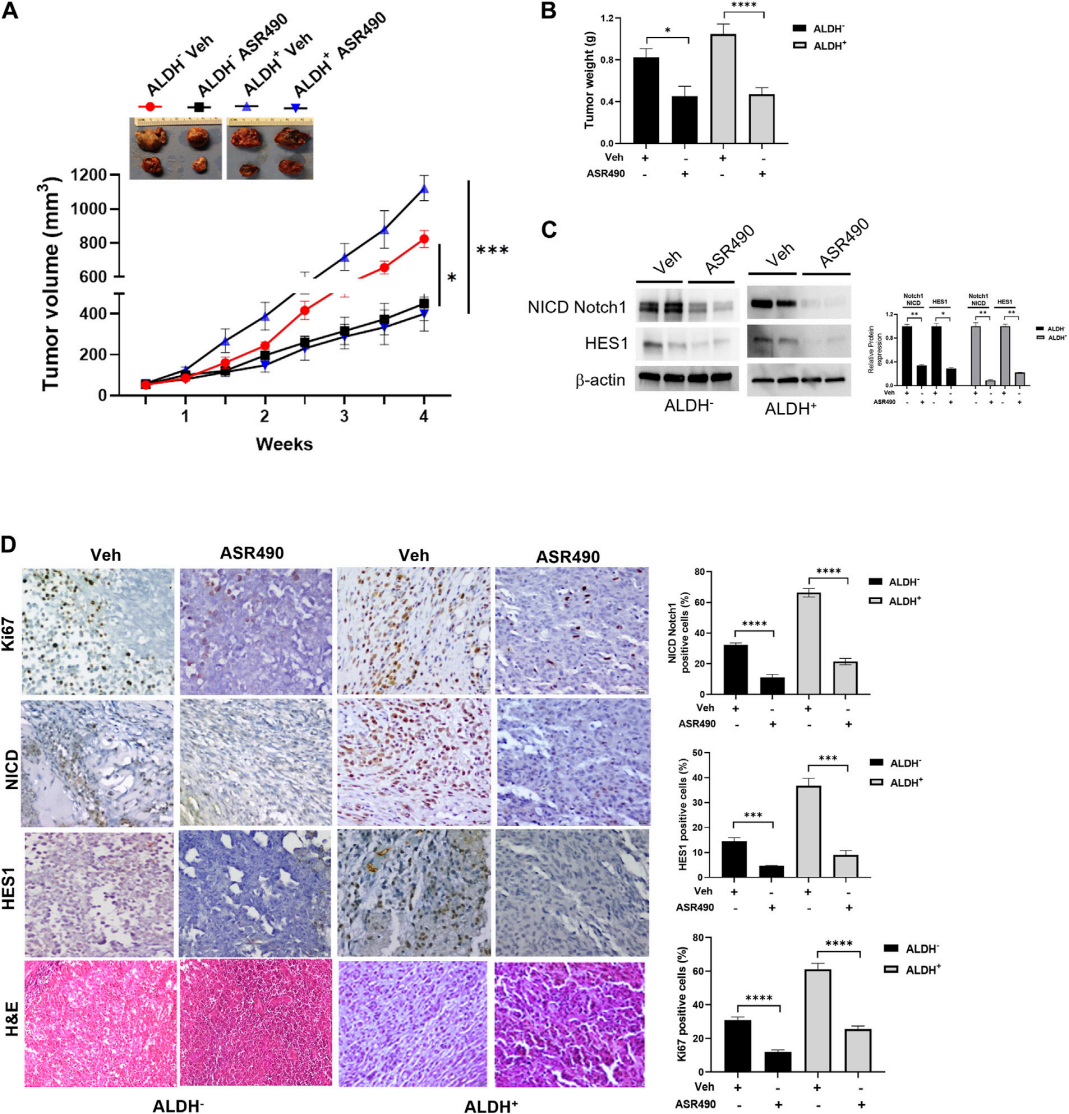
ASR490 reduces the tumor burden of xenotransplanted breast tumors. **(A)** Oral administration of ASR490 (25 mg/kg) significantly inhibited the growth of ALDH^−^ and ALDH^+^ xenotransplanted tumors (*n* = 6, *p < 0.01, ***p < 0.001). **(B)** Tumor weight of vehicle and ASR490 treated ALDH^−^ and ALDH^+^ tumors. **(C)** Western blots performed for Notch1-NICD and HES1 on vehicle and ASR490-treated ALDH^−^ and ALDH^+^ tumors. **(D)** IHC analyses was performed on vehicle and ASR490-treated ALDH^−^ and ALDH^+^ tumors to evaluate the expressions of Notch1-NICD, HES1, and Ki67 (proliferation marker). *p* values were calculated using a two-sided Student’s t-test.

Next, the anticancer effect of ASR490 was evaluated *in vivo* using ALDH^+^ and ALDH^−^ xenografted mice. Oral administration of ASR490 significantly reduced the tumor burden of both ALDH^+^ and ALDH^−^ xenografted mice (Figure 9), although its effect was more profound in the ALDH^+^ group (Figure 9A). The weight of ASR490-treated tumors was also lower than that of the vehicle-treated tumors (Figure 9B). Assessment of ASR490 treatment on Notch1 signaling in tumor tissue lysates showed significantly decreased Notch1-NICD and HES1 protein expression in the ASR490-treated groups compared to the vehicle-treated groups (Figure 9C). This finding was corroborated by IHC analyses, which showed significant downregulation of NICD, Hes1, and Ki67 (proliferation marker) expressions in the ASR490-treated tumors (Figure 9D).

## Discussion

This study provides evidence that the newly developed potent small molecule, ASR490, explicitly suppresses Notch1 expression in BCSCs and BC by inhibiting cell proliferation and tumor growth in both *in vitro* and *in vivo* models. For more than twodecades, there has been a growing interest in targeting Notch1 signaling, resulting in the development of several approaches to inhibit Notch1 signaling in preclinical models. In fact, to date, almost all Notch1 inhibitors have largely failed in their clinical management due to gastrointestinal toxicities, non-selective Notch inhibition, and effective therapeutic doses to curb tumor growth (Imbimbo, 2008; Wu et al., 2010).

Our newly developed molecule, ASR490, is a selective Notch1 inhibitor that attenuates tumor growth in BCSC and BC models. ASR490 differs from existing Notch1 inhibitors due to its specificity towards NRR, oral bioavailability, and non-toxicity to normal breast cells. Our dose and time-dependent analyses demonstrated that ASR490 inhibited Notch1 activity in both BCSCs (ALDH^+^ and CD44^+^/CD24^−^) and BC (ALDH^−^) cells. In addition, we observed that ASR490’s effects were more prominent in cells expressing higher levels of Notch1 (i.e., ALDH^+^ and CD44^+^CD24^−^) than in lower-Notch1-expressing ALDH^−^ cells. We also demonstrated that this inhibitor specifically inhibited Notch1 expression without affecting the expression of Notch2, in contrast to the pan-Notch inhibitor DAPT and minimal or no toxicity was observed following treatment with ASR490 in both *in vitro* and *in vivo* models. Remarkably, serial passaging of ASR490-treated BCSCs and BC cells demonstrated that ASR490 has long-term effects on these cells, as evidenced by the diminished spheroid forming ability that was carried over for at least two successive generations.

The presence of both ALDH^+^ and CD44^+^CD24^−^ populations have been previously correlated with a poor prognosis of BC patients (Ginestier et al., 2007; Ohi et al., 2011) and were showed to be able to induce lung metastasis (Sheridan et al., 2006). Moreover, others have shown that chemotherapy can enrich ALDH + populations within tumors, making them more resistant (Tanei et al., 2009). Interestingly, recent studies have suggested a role for Notch1 in drug-resistant BCSCs where exposure to chemo- and anti-hormonal therapies result in the enrichment of drug-resistant ALDH^+^ BCSCs (Osipo et al., 2008; Suman et al., 2013; Baker et al., 2018). These results along with the well-established oncogenic role of Notch1 in BC (Reedijk et al., 2005; Hu et al., 2006; Mohammadi-Yeganeh et al., 2015), where its increased expression has been shown to enhance metastatic phenotype (Zang et al., 2010; Li et al., 2015), suggests that inhibition of Notch1 could eliminate BCSCs and increase drug sensitivity. Our results indicate that ASR490 is a potent compound that can overcome Notch1 mediated BCSCs accumulation and resistance in BC.

In our studies, ASR490’s inhibition of Notch1 facilitated autophagy-mediated cell death in both BCSC and BC cells. The autophagy function is highly context-dependent (Chavez-Dominguez et al., 2020). For example, inhibition of Notch1 in glioblastoma cell lines induced the oncogenic function of autophagy; however, in combination with γ-secretase inhibitor RO4929097 (GSI) and a natural compound, Resveratrol, resulted in the accumulation of autophagosomes and subsequent growth inhibition by inducing apoptosis (Giordano et al., 2021). Whereas in lung cancer models, the induction of autophagy resulted in the downregulation of NICD expression and the eventual inhibition of EMT signaling (Zada et al., 2022). Our results suggest that Notch1 regulates the pro-apoptotic function of autophagy. Silencing of Notch1 in ALDH^+^ cells induced autophagy signaling, while in contrast overexpression of Notch1 blocked ASR490-mediated autophagy, suggesting that Notch1 regulates autophagy function in BCSC cells.

Moreover, autophagy has been implicated to play a critical role specifically in BCSCs populations. For example, the impairment of autophagy has been shown to affect the maintenance of BCSCs by limiting EMT and the CD44^+^/CD24^−^ phenotype (Cufi et al., 2011). Analysis of the mechanism of action revealed that ASR490 mediated its effects by suppressing Notch1-induced EMT signaling in BCSCs and BC cells. The loss of E-cadherin and the upregulation of β-catenin expression is a classical molecular switch for EMT that initiates the loss of apical polarity by altering cytoskeleton organization and leads to spindle-shaped morphologic features (Leong et al., 2007; Gangopadhyay et al., 2013). We found that induction of E-cadherin and inhibition of β-catenin expression in ASR490-treated BCSCs and BC cells suggest the abrogation of this EMT phenomenon by ASR490. Activation of the pro-survival transcription factor NF*κ*B (p65) is involved in EMT induction (Thiery et al., 2009) and is a crucial activator of BCSCs (Liu et al., 2010). Our results showed that ASR490 inhibited p65 expression as well as the expression its downstream targets Bcl-2 and BCL-xL in both BCSCs and BC cells.

ASR490 is an orally available potent inhibitor of Notch1-mediated activation that significantly abrogates the growth of BCSC and BC tumors. Moreover, the maximum tolerated dose of ASR490 was found to be 500 mg/kg (data not shown), which is approximately 20 times more than the dose used in our current study, indicative of ASR490’s high therapeutic index. Therefore, with its potent activity and lack of apparent toxicity, ASR490 provides the necessary selectivity and therapeutic window for cancer therapeutics targeting the Notch1 pathway and selectively inhibiting BCSC populations within tumors.

In conclusion, our results demonstrate a novel therapeutic strategy for BCSCs specifically for TNBC, by inhibiting Notch1 signaling, which curtails self-renewal and tumorigenicity. Also, the inhibition of Notch1 facilitates autophagy signaling, which resulted in the inhibition of EMT, and survival signaling to eradicate tumor growth. Hence, our small molecule ASR490 is a promising therapeutic agent, and the inhibition of Notch1 is an ideal strategy for BCSC and BC.

## Data Availability

The original contributions presented in the study are included in the article/Supplementary Material, further inquiries can be directed to the corresponding author.

## Supplementary material

SUPPLEMENTARY FIGURE S1 Volcano plot analyses of differentially expressed genes between ALDH^−^ treatment vs control, ALDH^+^ treatment vs. control, and ALDH^+^ vs. ALDH^−^ is plotted on the X axis, and the False Discovery Rate (FDR) significance is plotted on the Y axis (−log10 scale). The grey dots represent no significant change, red dots represent logFC of >1.5 and FDR < 0.05 and blue represent logFC < −1.5 and FDR <0.05.

SUPPLEMENTARY FIGURE S2 KEGG pathway for differential expressed genes based on RNA-seq data for ALDH^−^ treatment vs. control, ALDH^+^ treatment vs. control and ALDH^+^ against ALDH^−^. Each bubble’s color and size correspond to the amount of differentially expressed mRNAs that are enriched in a certain GO keyword or KEGG pathway, respectively. The cutoff utilized to choose KEGG keywords was p<0.05.

SUPPLEMENTARY FIGURE S3 Representative brightfield and immunofluorescent images of Notch1-NICD (active form of Notch1) expression in vehicle and ASR490-treated second and third generation mammospheres of ALDH^−^, ALDH^+^ and CD44^+^/CD24^−^ cells.

SUPPLEMENTARY FIGURE S4 Gene Ontology analysis for differential expressed genes based on RNA-seq data for ALDH^−^ treatment vs. control, and ALDH^+^ treatment vs. control.
