# Corrigendum: A small molecule inhibitor of Notch1 modulates stemness and suppresses breast cancer cell growth

**DOI:** 10.3389/fphar.2023.1207589

**Published:** 2023-05-03

**Authors:** Uttara Saran, Balaji Chandrasekaran, Ashish Tyagi, Vaibhav Shukla, Amandeep Singh, Arun K. Sharma, Chendil Damodaran

**Affiliations:** ^1^ Texas A&M University, College Station, TX, United States; ^2^ Penn State Cancer Institute, College of Medicine, The Pennsylvania State University, Hershey, PA, United States

**Keywords:** breast cancer, breast cancer stem cell (BCSC), ASR490, autophagy, Notch1

In the published article, there was an error in **Supplementary Data Sheet S1**. The western blot image for Figure 5A did not match the corresponding full western blot image in Data Sheet 1. The correct Western blot image for Figure 5A has been updated.

The authors apologize for this error and state that this does not change the scientific conclusions of the article in any way. The original article has been updated.

