# Corrigendum: A small molecule inhibitor of Notch1 modulates stemness and suppresses breast cancer cell growth

**DOI:** 10.3389/fphar.2024.1420266

**Published:** 2024-05-27

**Authors:** Uttara Saran, Balaji Chandrasekaran, Ashish Tyagi, Vaibhav Shukla, Amandeep Singh, Arun K. Sharma, Chendil Damodaran

**Affiliations:** ^1^ Texas A&M University, College Station, United States; ^2^ Penn State Cancer Institute, College of Medicine, The Pennsylvania State University, Hershey, PA, United States

**Keywords:** breast cancer, breast cancer stem cell (BCSC), ASR490, autophagy, NOTCH1

In the published article, there was an error in Figure 9 as published. Specifically, in Figure 9D, the same image was repeated for both ALDH- HES1 (Veh) and ALDH + Ki67 (ASR490). The correct IHC image for ALDH- HES1 (Veh) has been updated. The corrected Figure 9 and its caption appear below.

**FIGURE 9:**
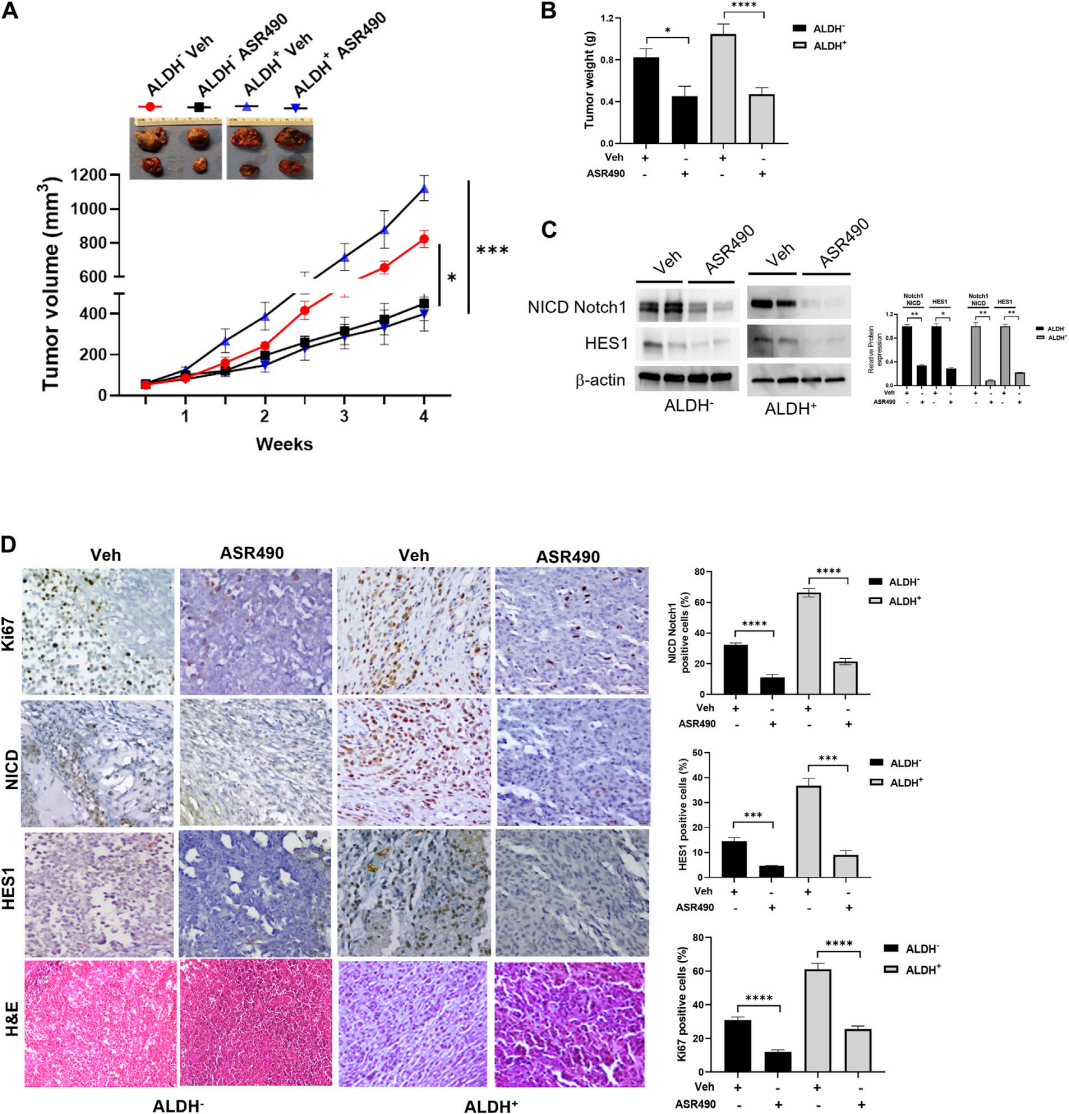
ASR490 reduces the tumor burden of xenotransplanted breast tumors. **(A)** Oral administration of ASR490 (25 mg/kg) significantly inhibited the growth of ALDH^−^ and ALDH^+^ xenotransplanted tumors (*n* = 6, *p < 0.01, ***p < 0.001). **(B)** Tumor weight of vehicle and ASR490 treated ALDH^−^ and ALDH^+^ tumors. **(C)** Western blots performed for Notch1-NICD and HES1 on vehicle and ASR490-treated ALDH^−^ and ALDH^+^ tumors. **(D)** IHC analyses was performed on vehicle and ASR490-treated ALDH^−^ and ALDH^+^ tumors to evaluate the expressions of Notch1-NICD, HES1, and Ki67 (proliferation marker). *p* values were calculated using a two-sided Student’s t-test.

The authors apologize for this error and state that this does not change the scientific conclusions of the article in any way. The original article has been updated.

