# Supplementary figures and images for: A small molecule inhibitor of Notch1 modulates stemness and suppresses breast cancer cell growth
Source: Front Pharmacol. 2023 Feb 24;14:1150774. doi: 10.3389/fphar.2023.1150774 (PMC9998682; doi:10.3389/fphar.2023.1150774)

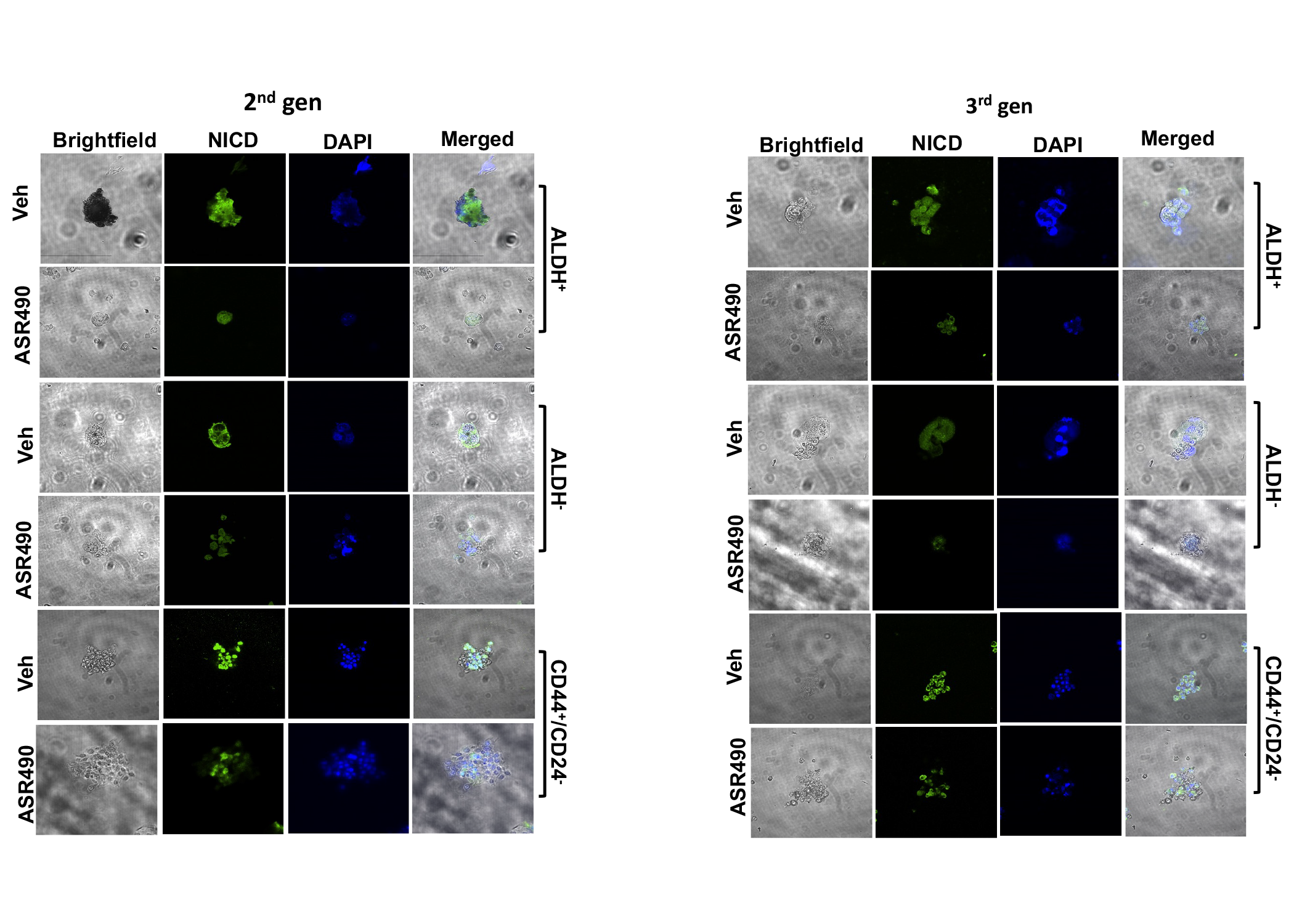

Supplement: Supplementary file 1 [file Image3.tif]

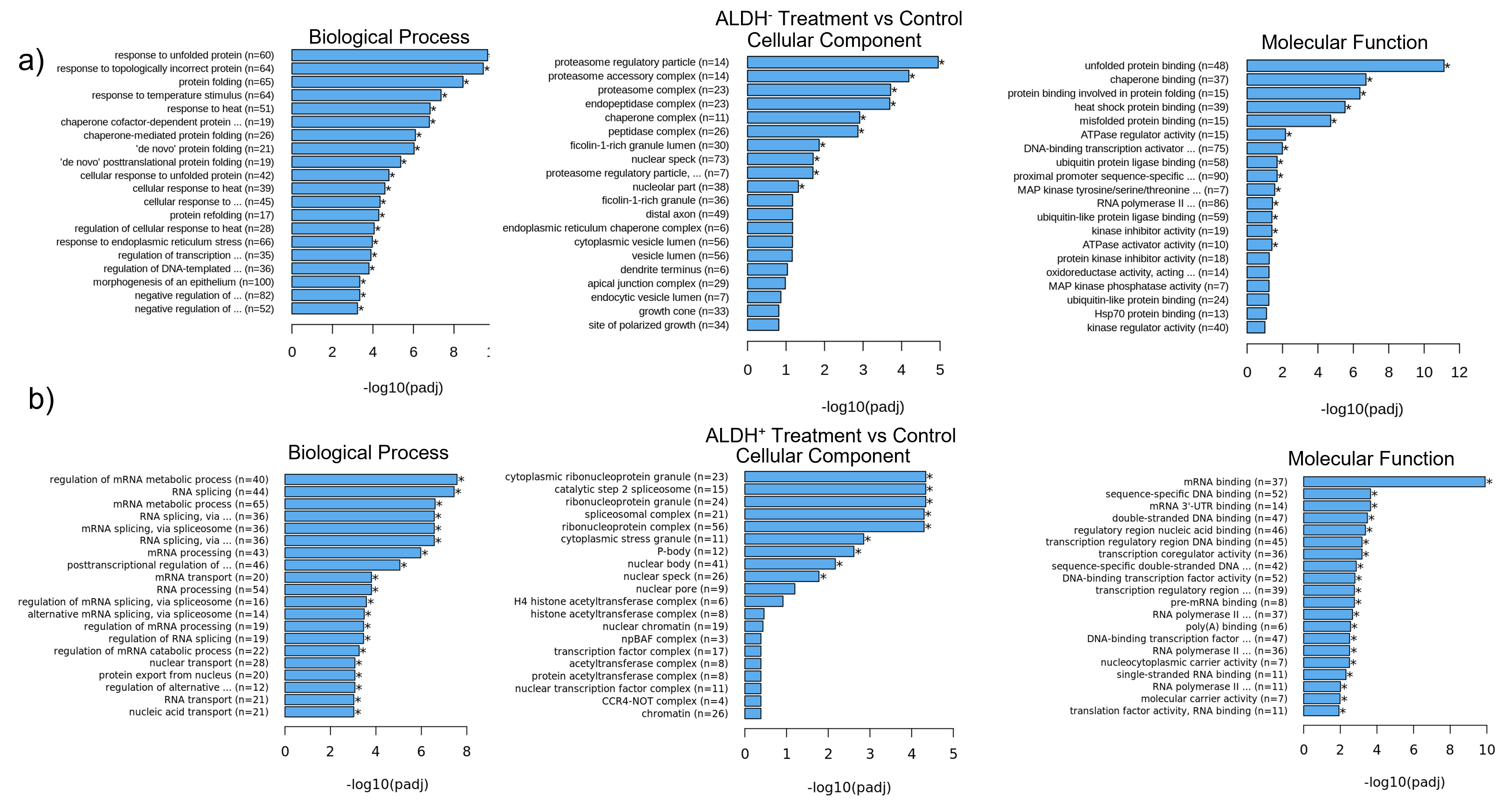

Supplement: Supplementary file 2 [file Image4.TIF]

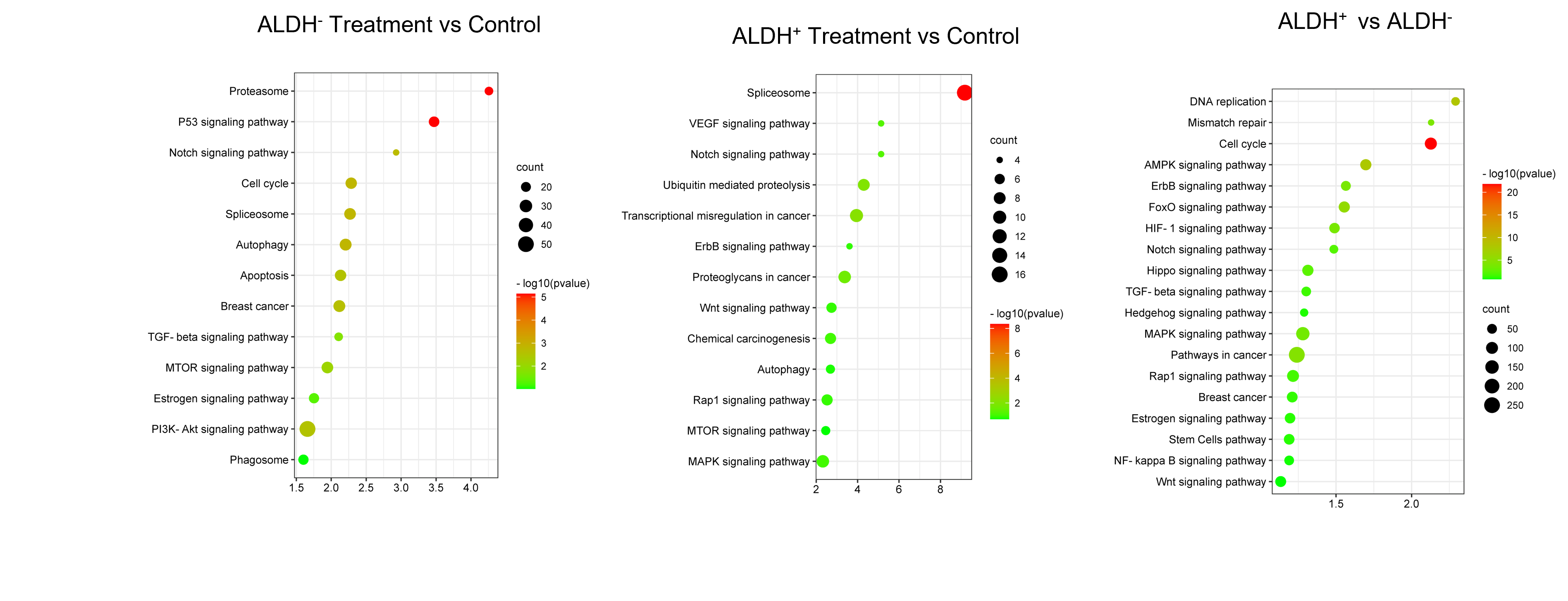

Supplement: Supplementary file 3 [file Image2.tif]

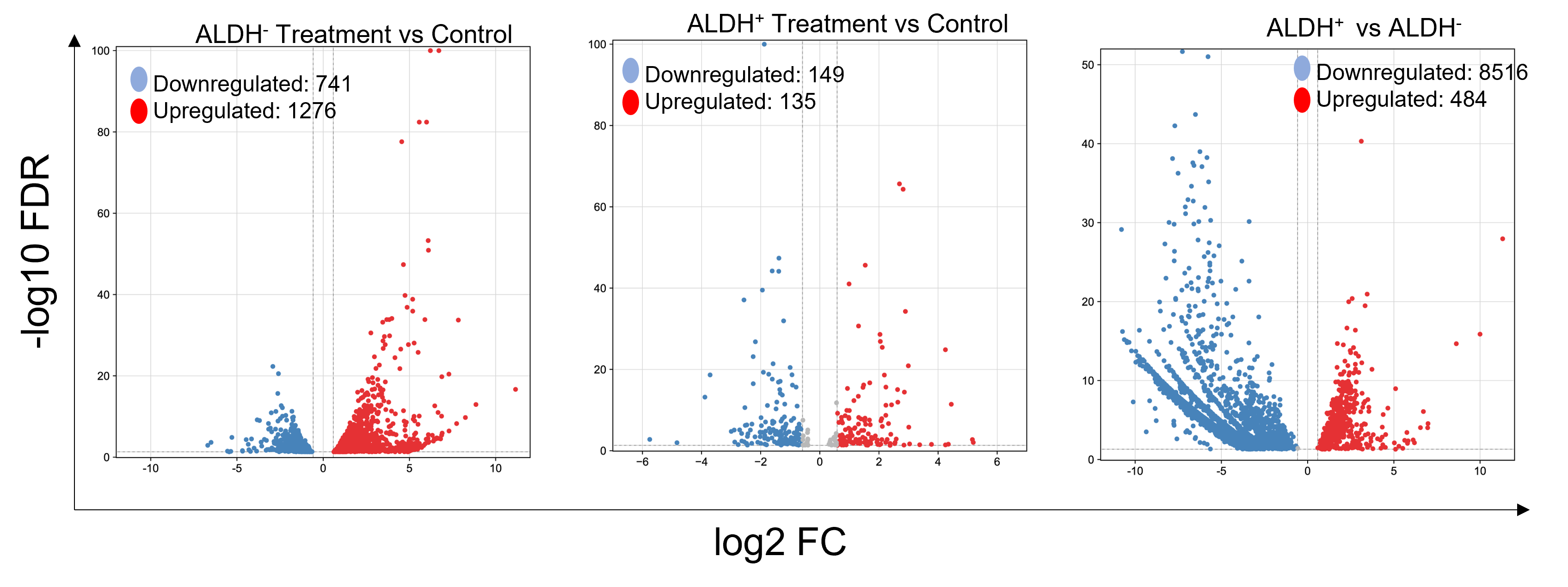

Supplement: Supplementary file 4 [file Image1.TIF]
