# Supplementary material for: A small molecule inhibitor of Notch1 modulates stemness and suppresses breast cancer cell growth
Source: Front Pharmacol. 2023 Feb 24;14:1150774. doi: 10.3389/fphar.2023.1150774 (PMC9998682; doi:10.3389/fphar.2023.1150774)

Figure 2A

NICD Notch1

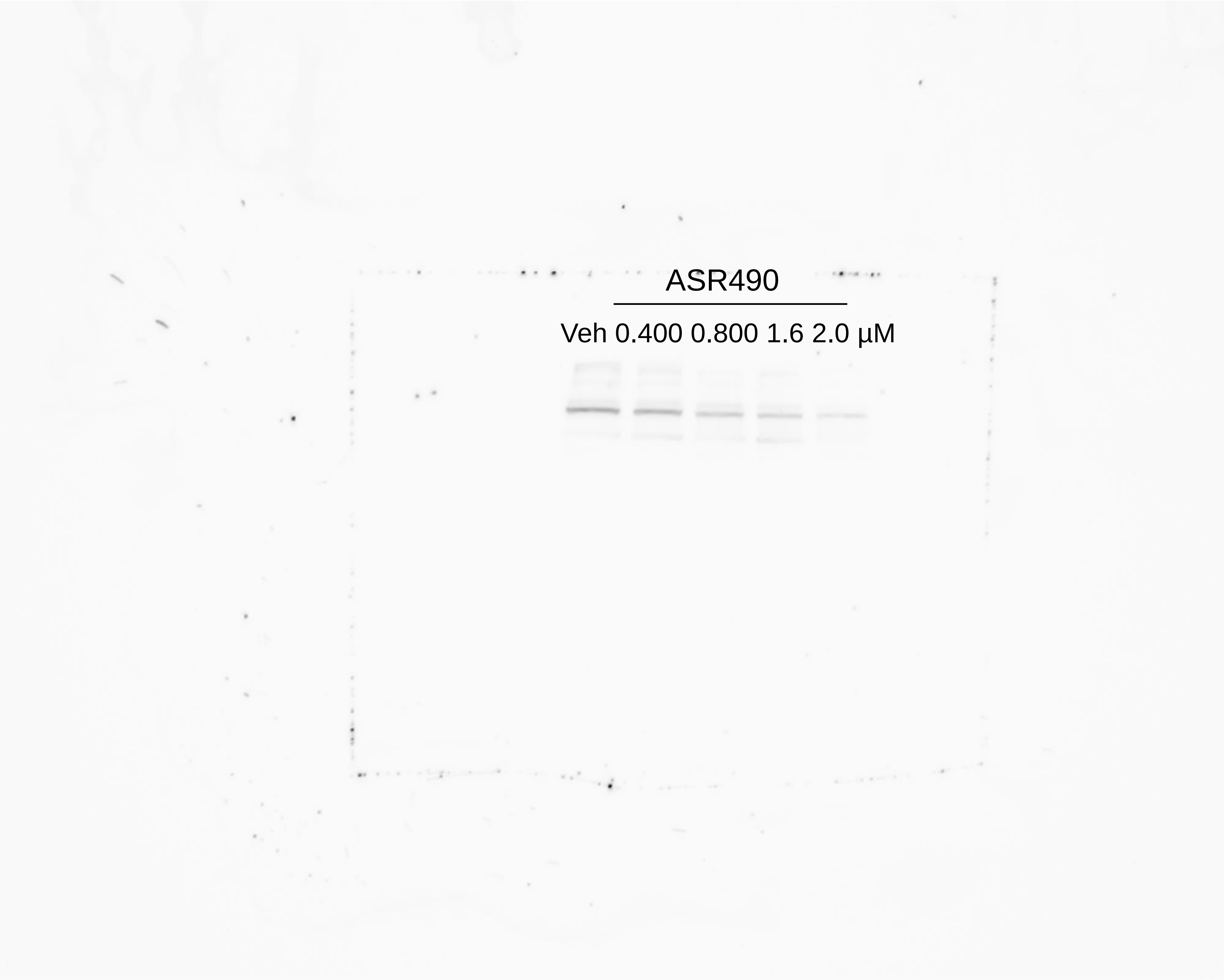

HES1

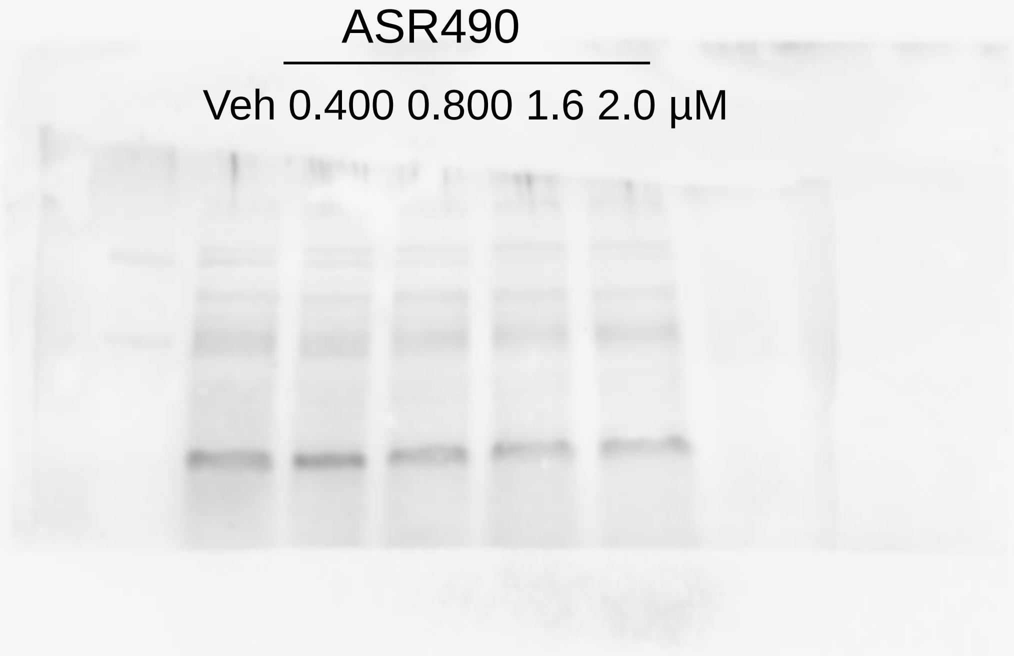

Hey1

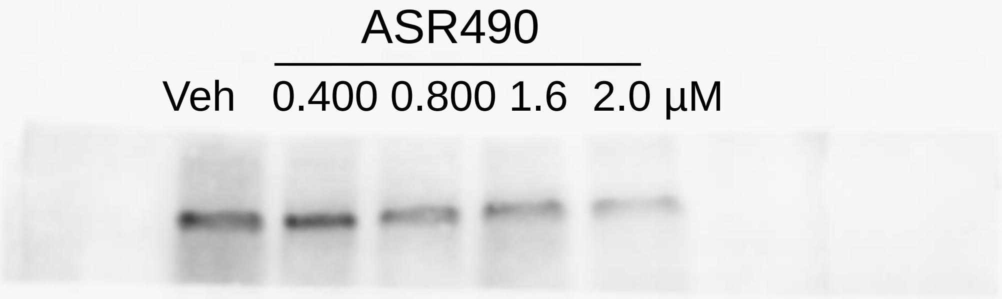

Actin

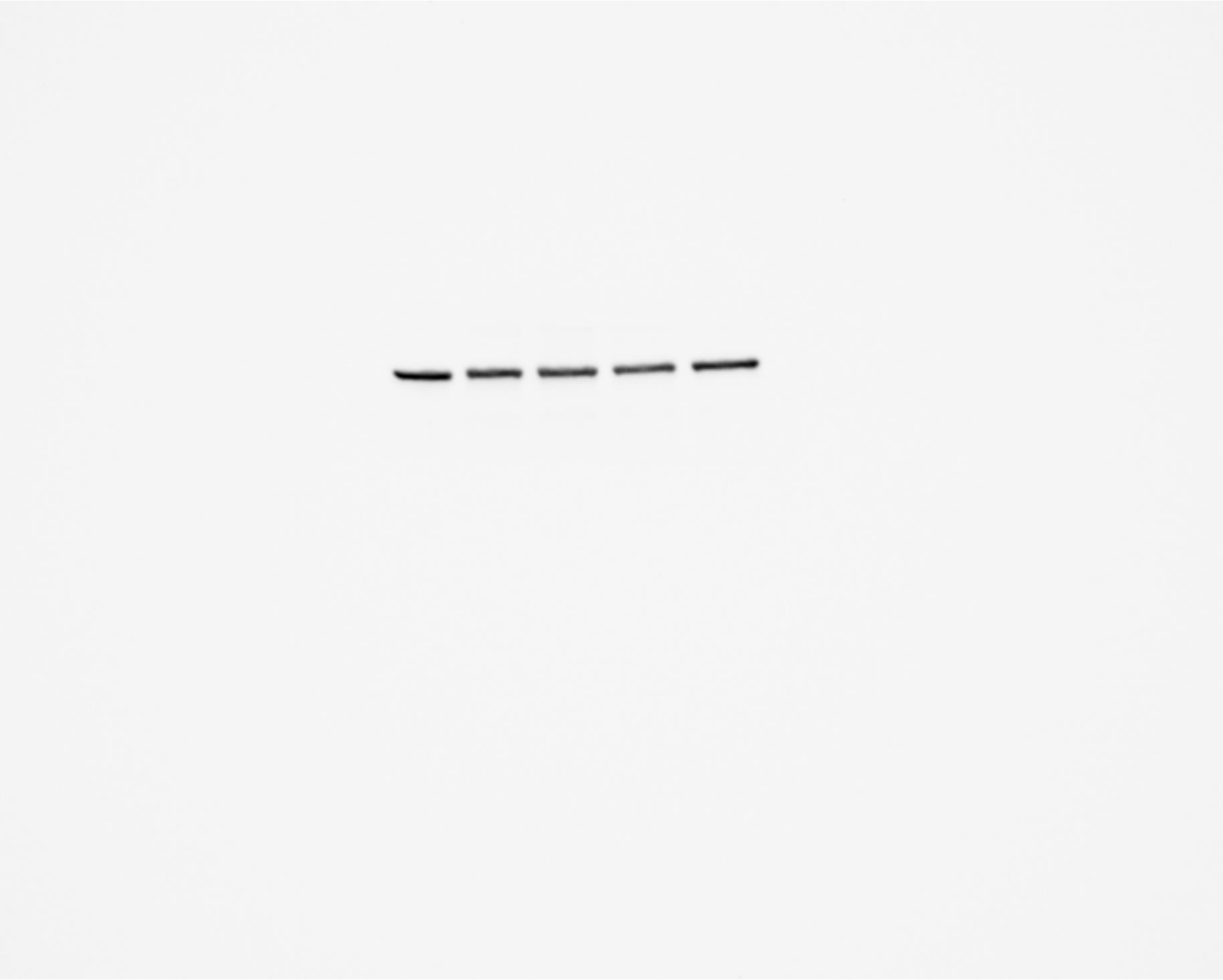

Figure 2B

NICD Notch1

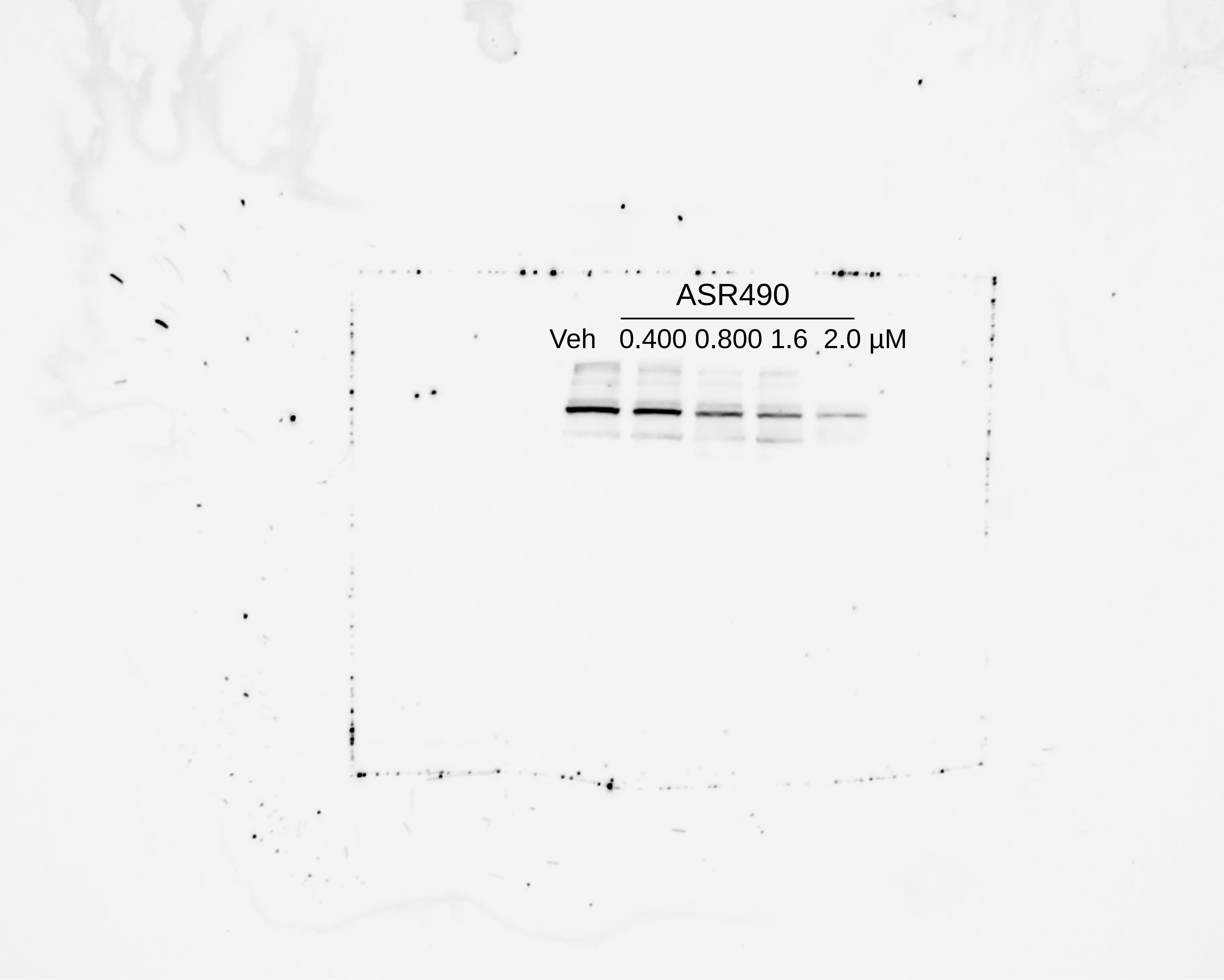

HES1

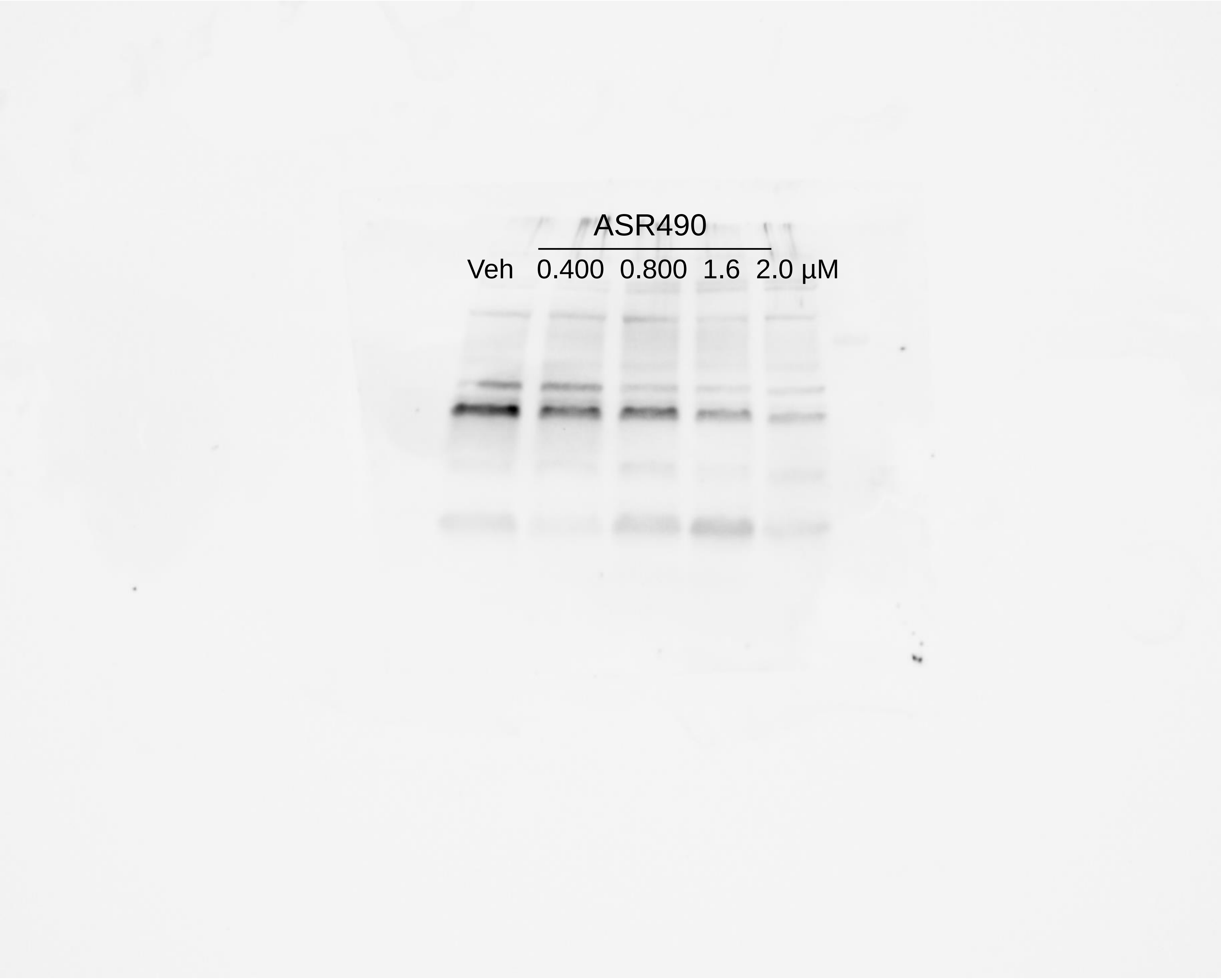

Hey1

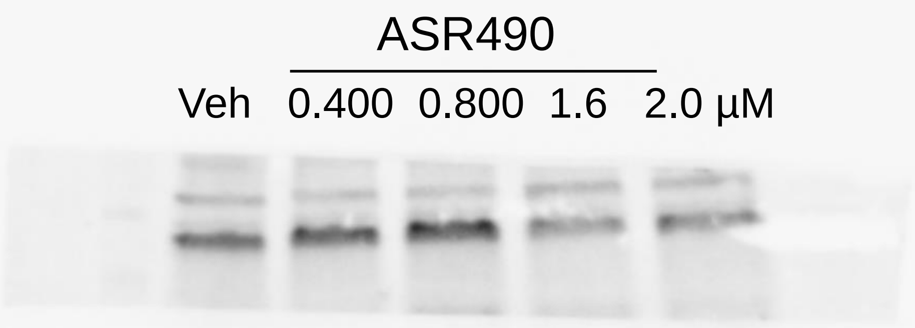

β-Actin

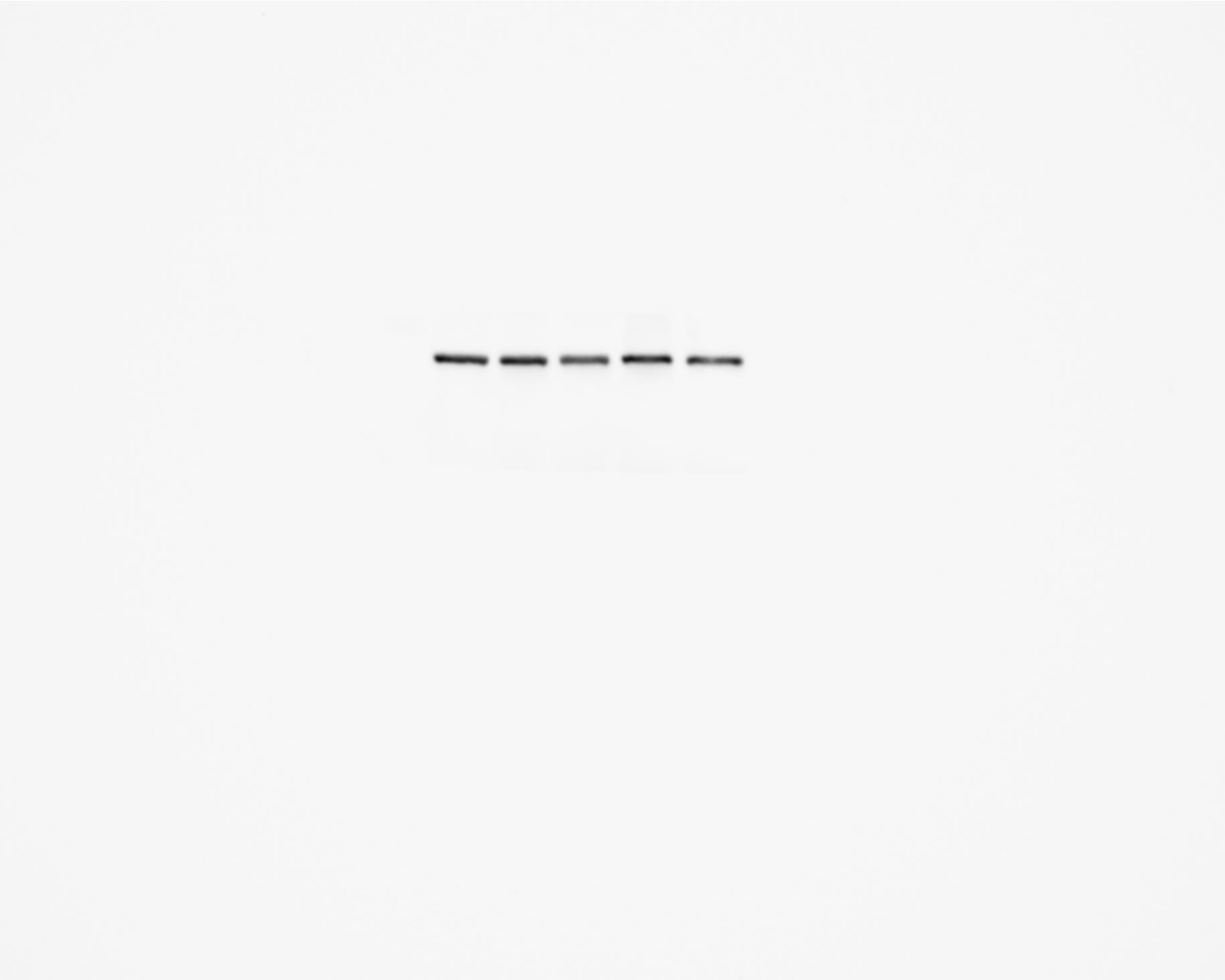

Figure 2C

NICD Notch1

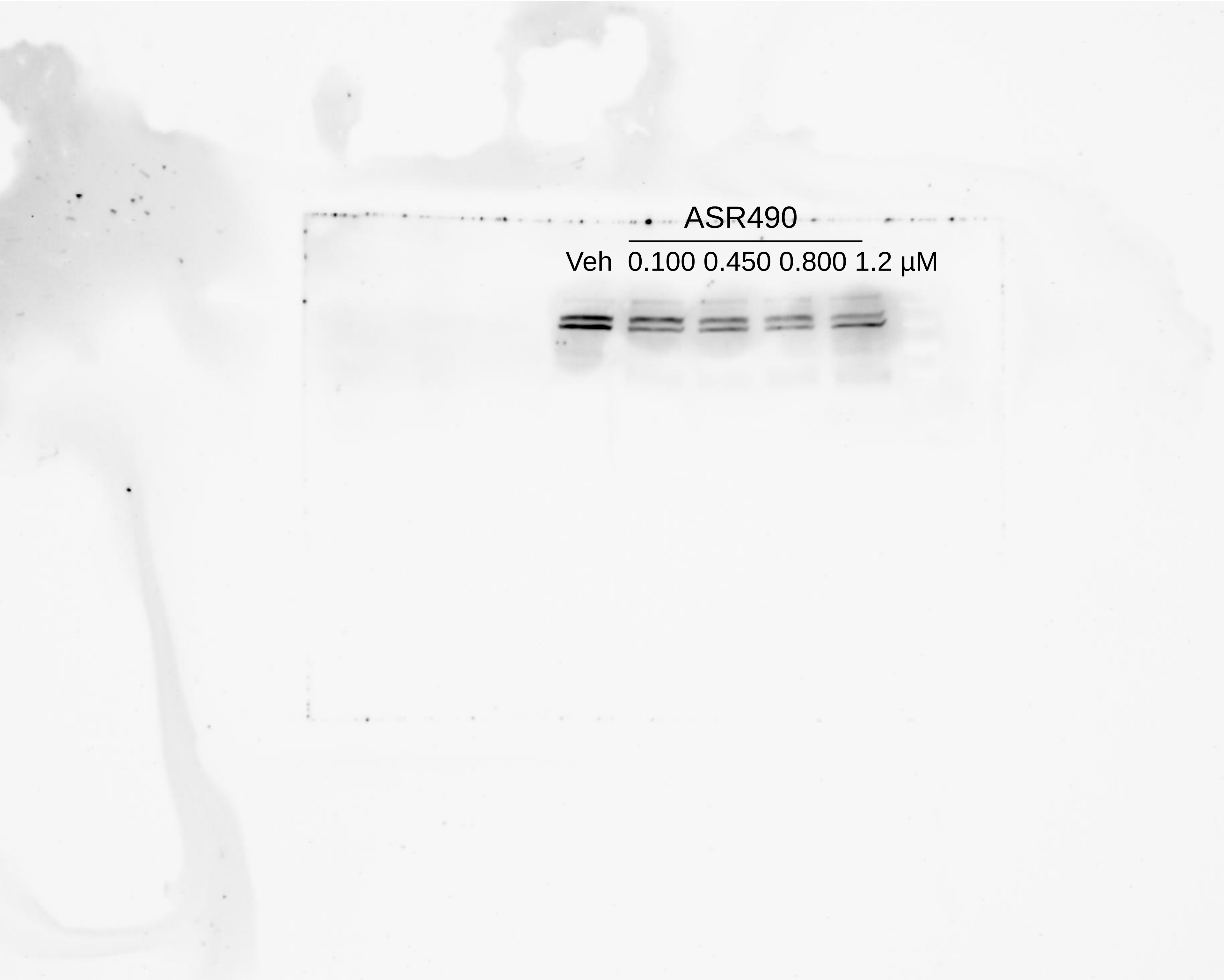

HES1

ASR490

Veh 0.100 0.450 0.800 1.2 µM

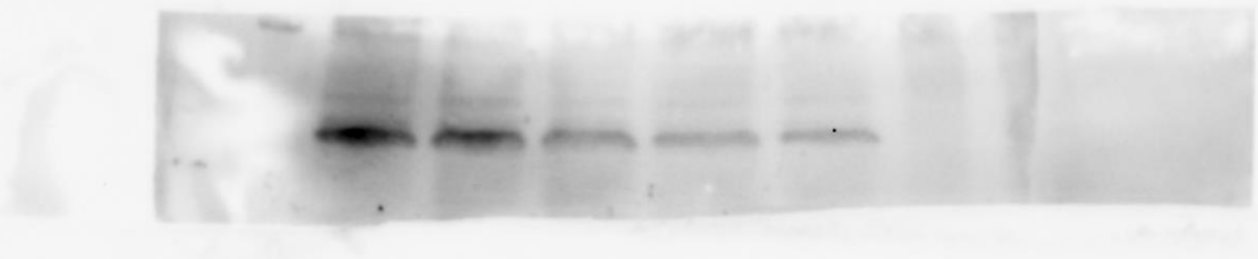

Hey1

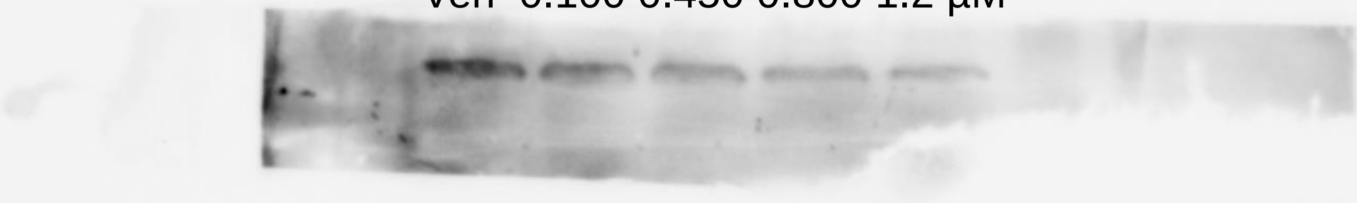

β-Actin

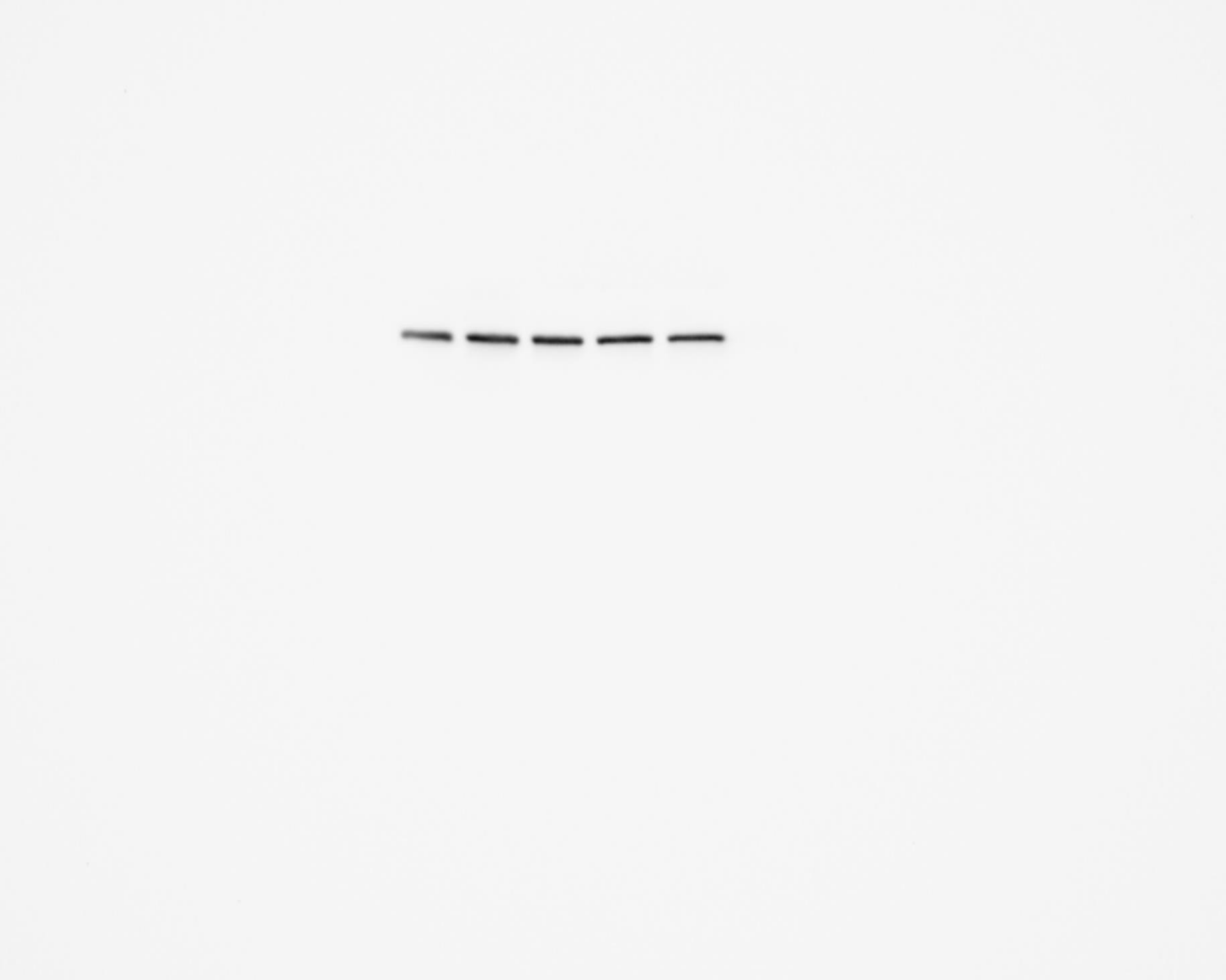

Figure 2D

NICD Notch1

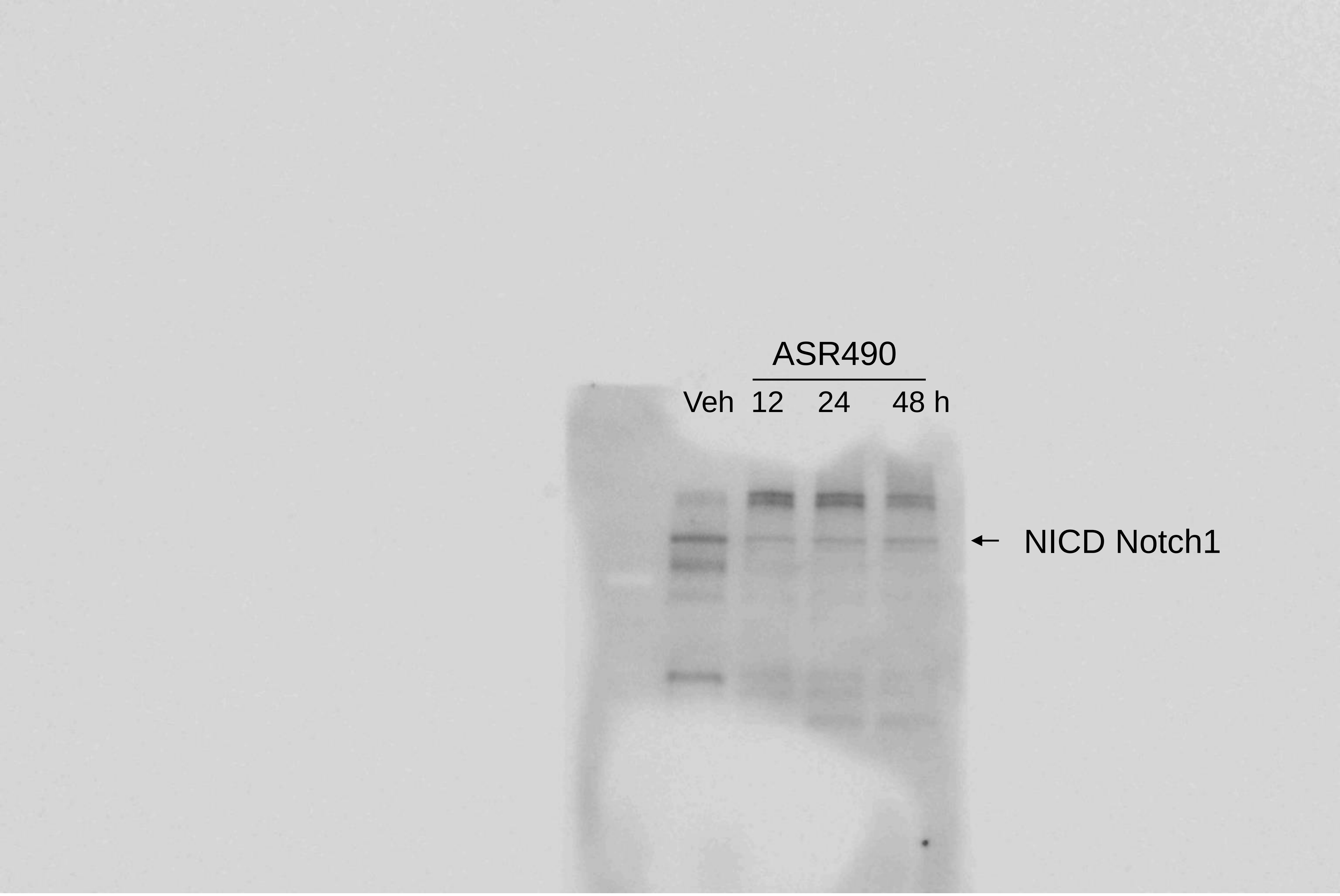

HES1

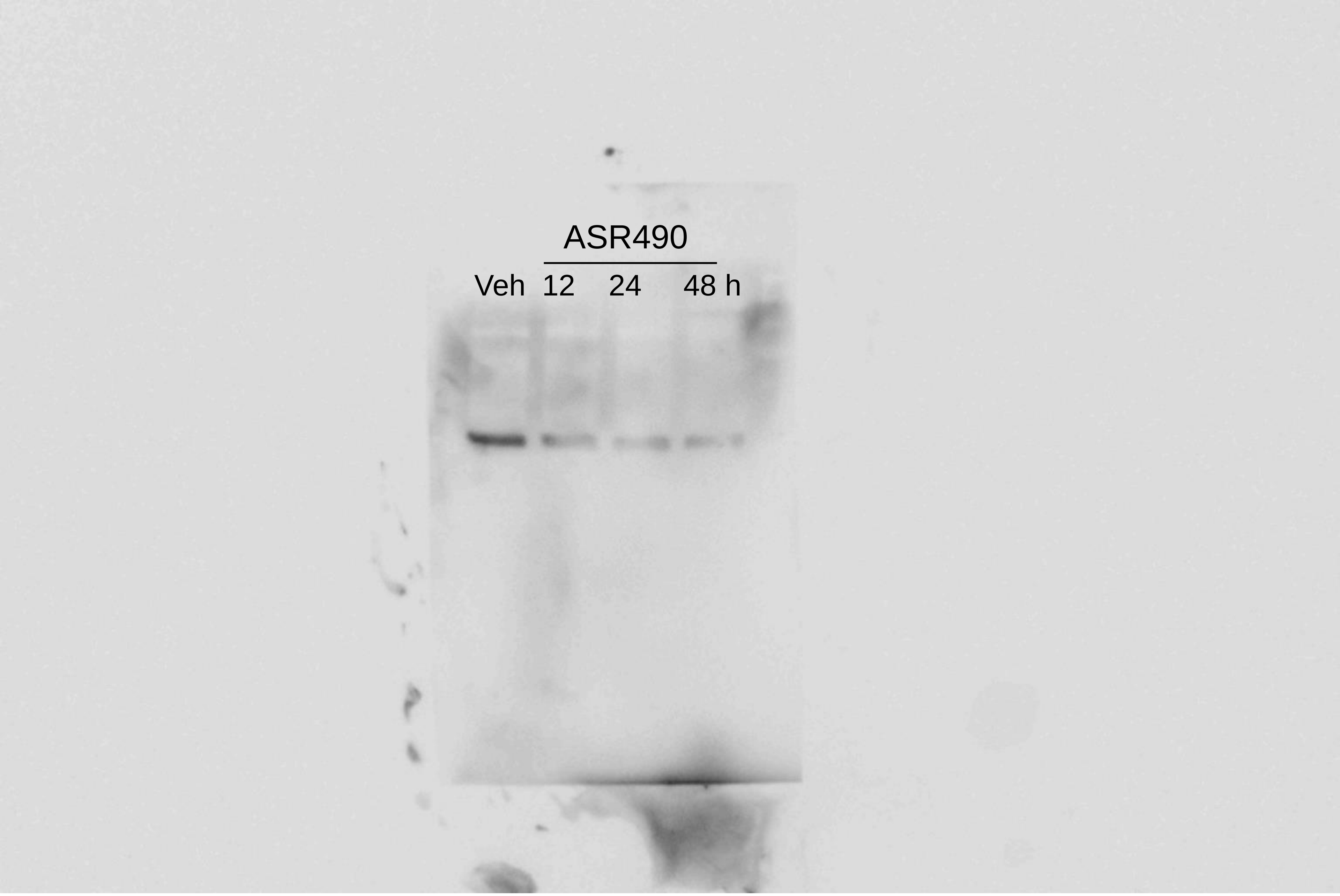

Hey1

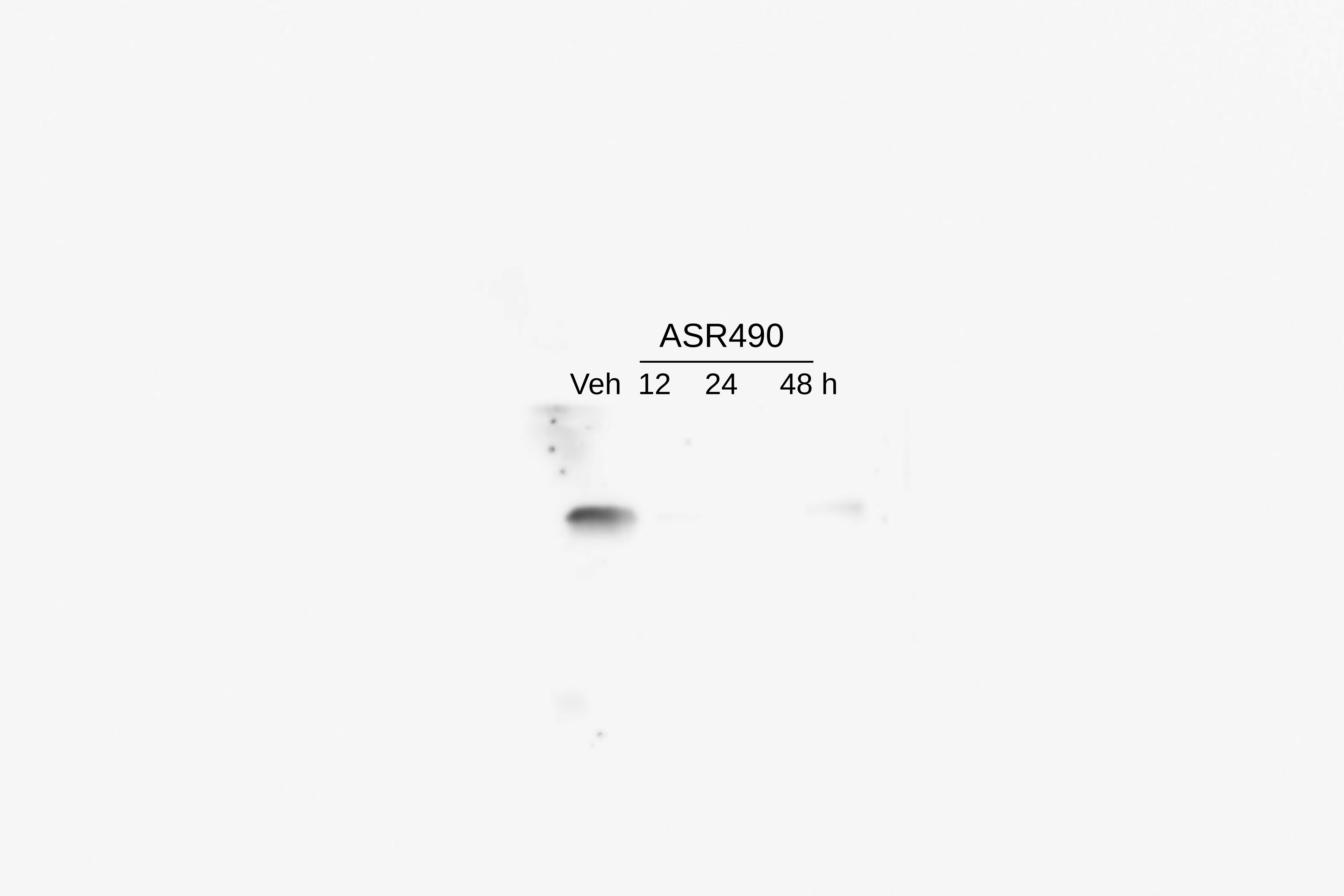

Actin

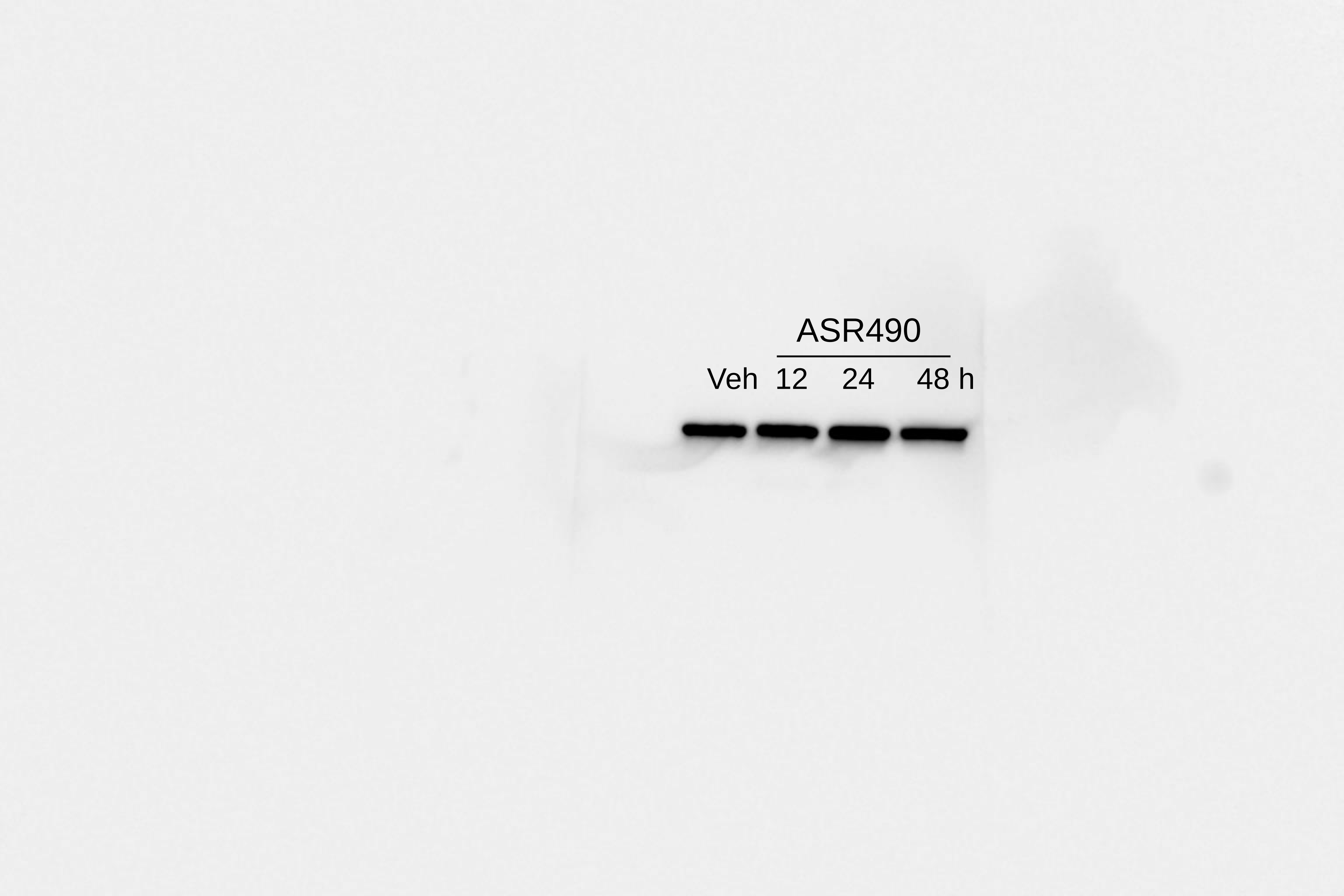

Figure 2E

NICD Notch1

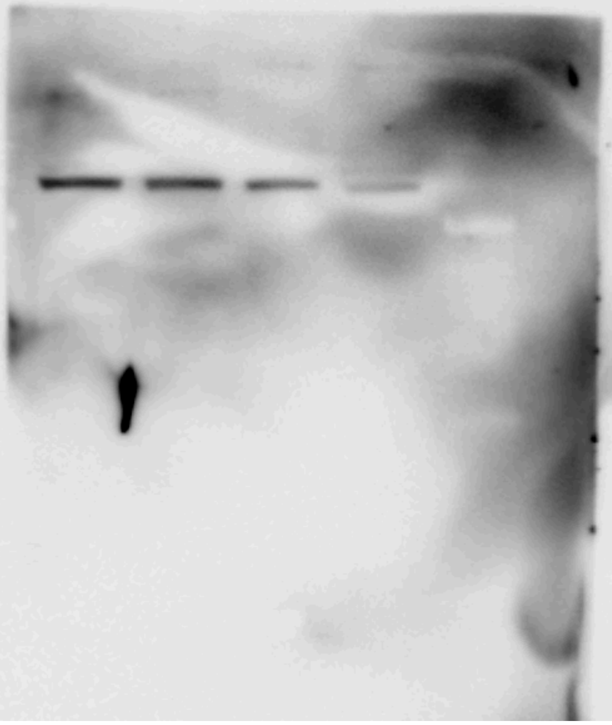

HES1

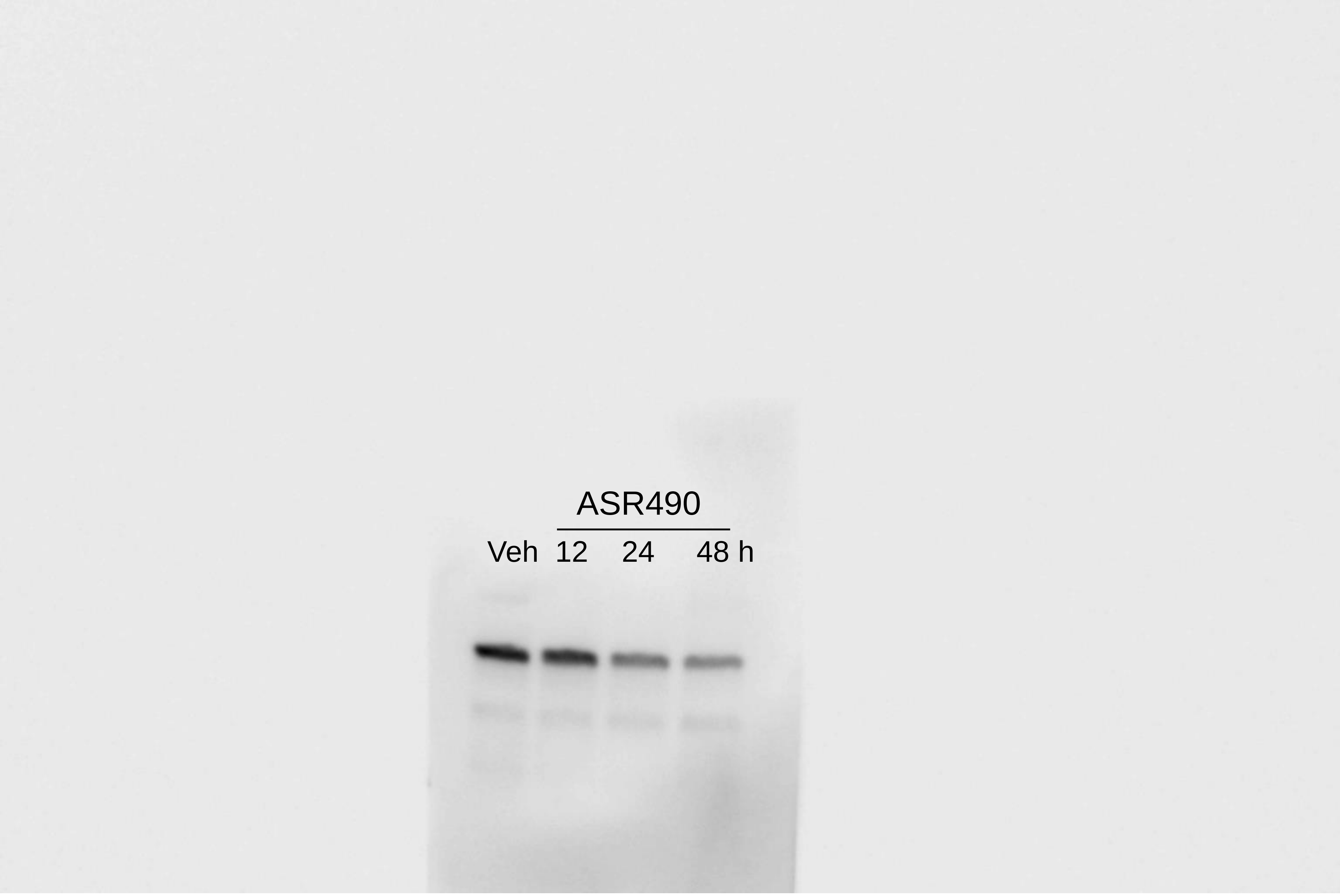

Hey1

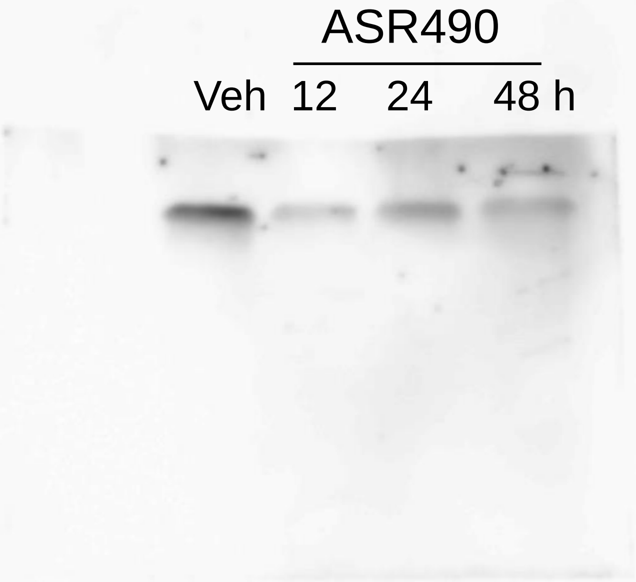

Actin

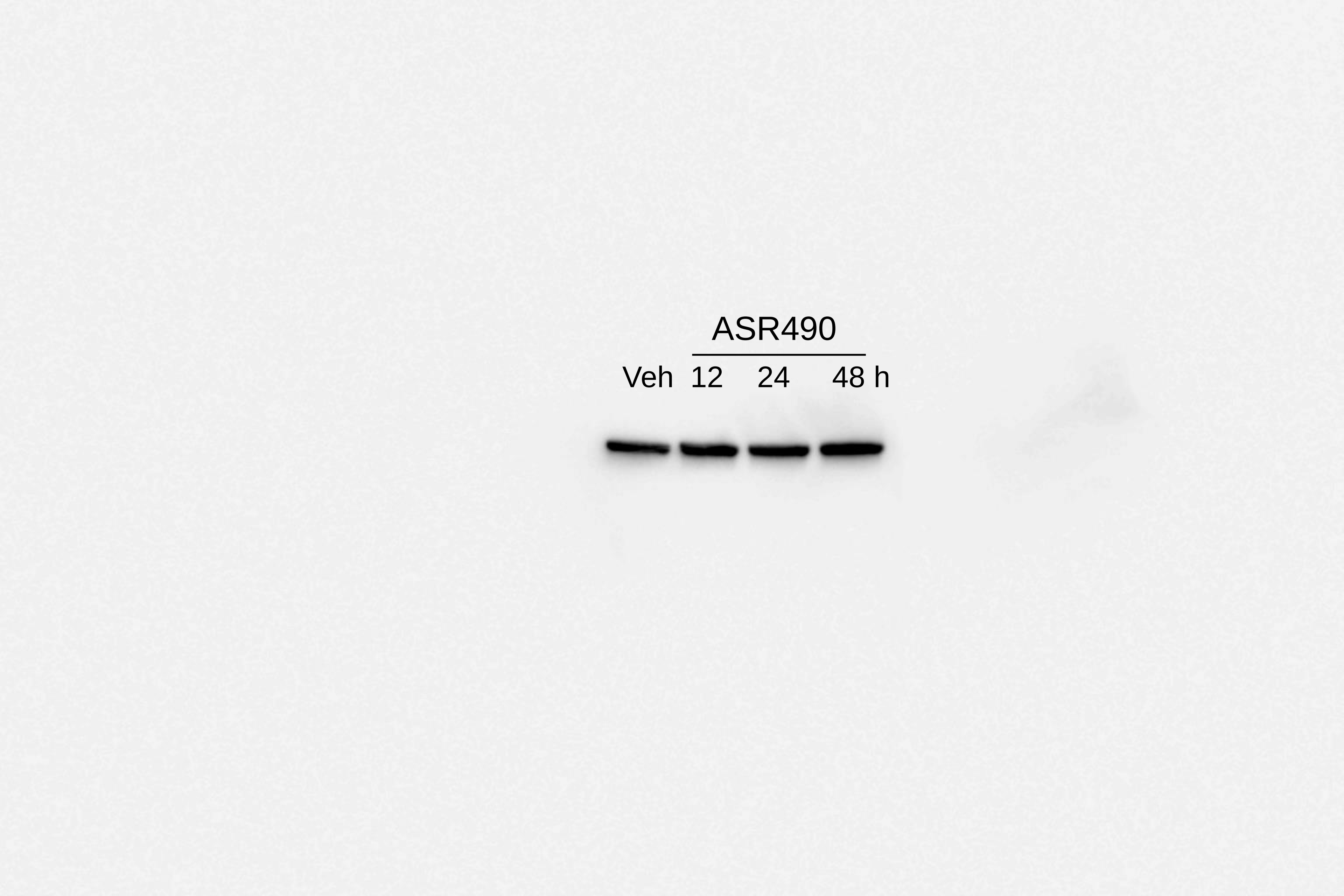

Figure 2F

NICD Notch1

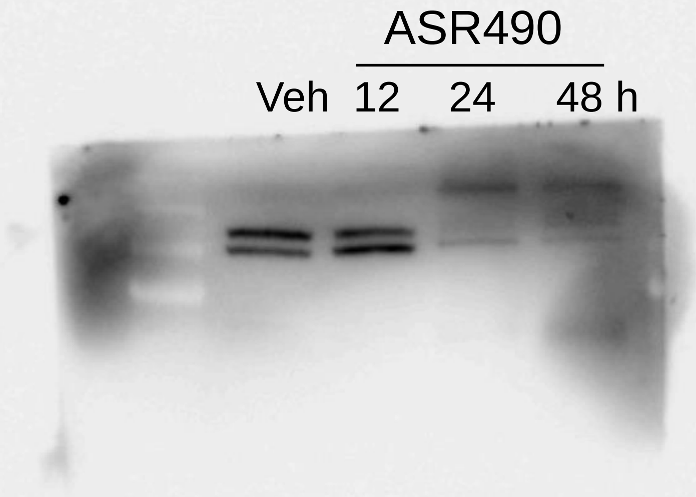

HES1

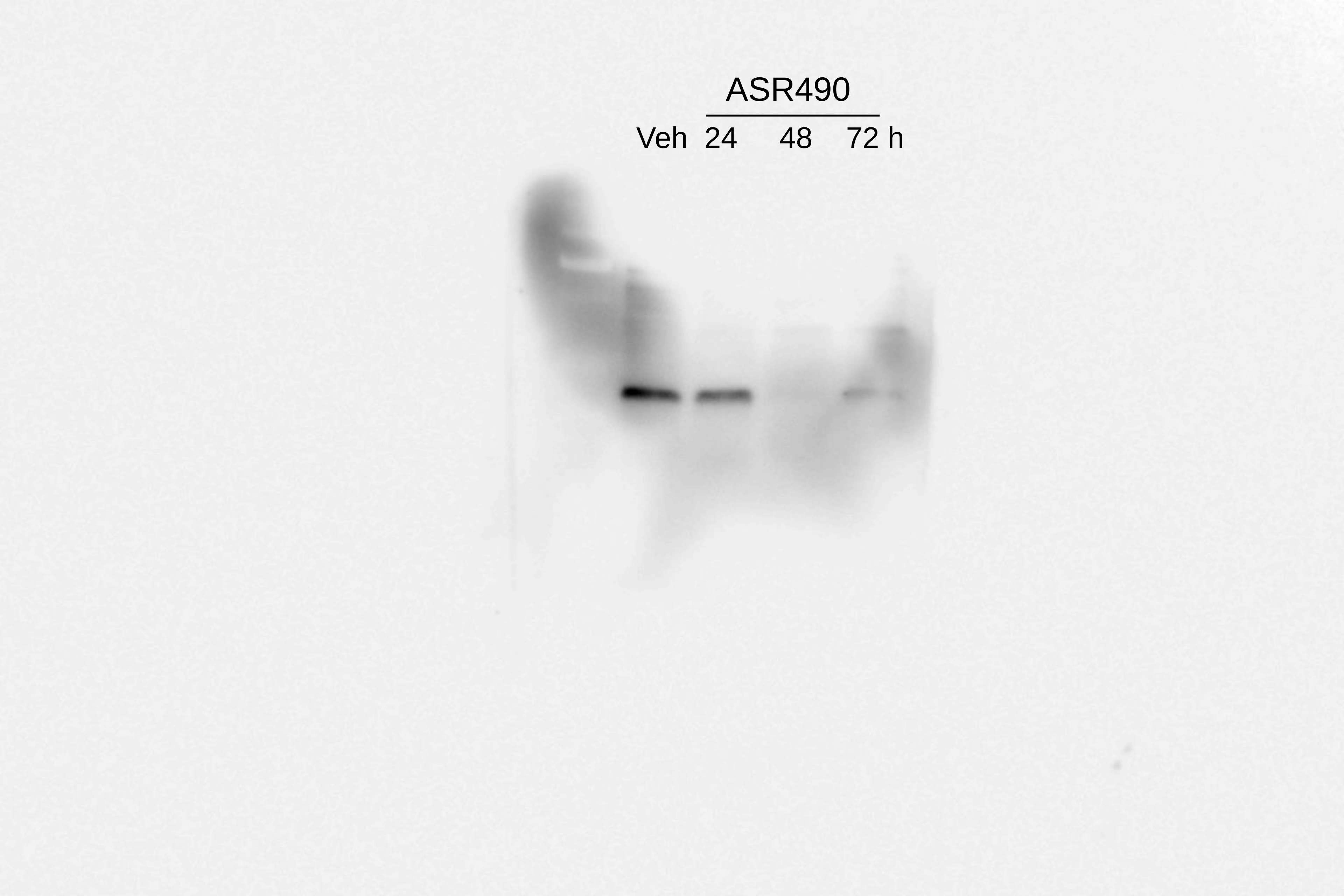

Hey1

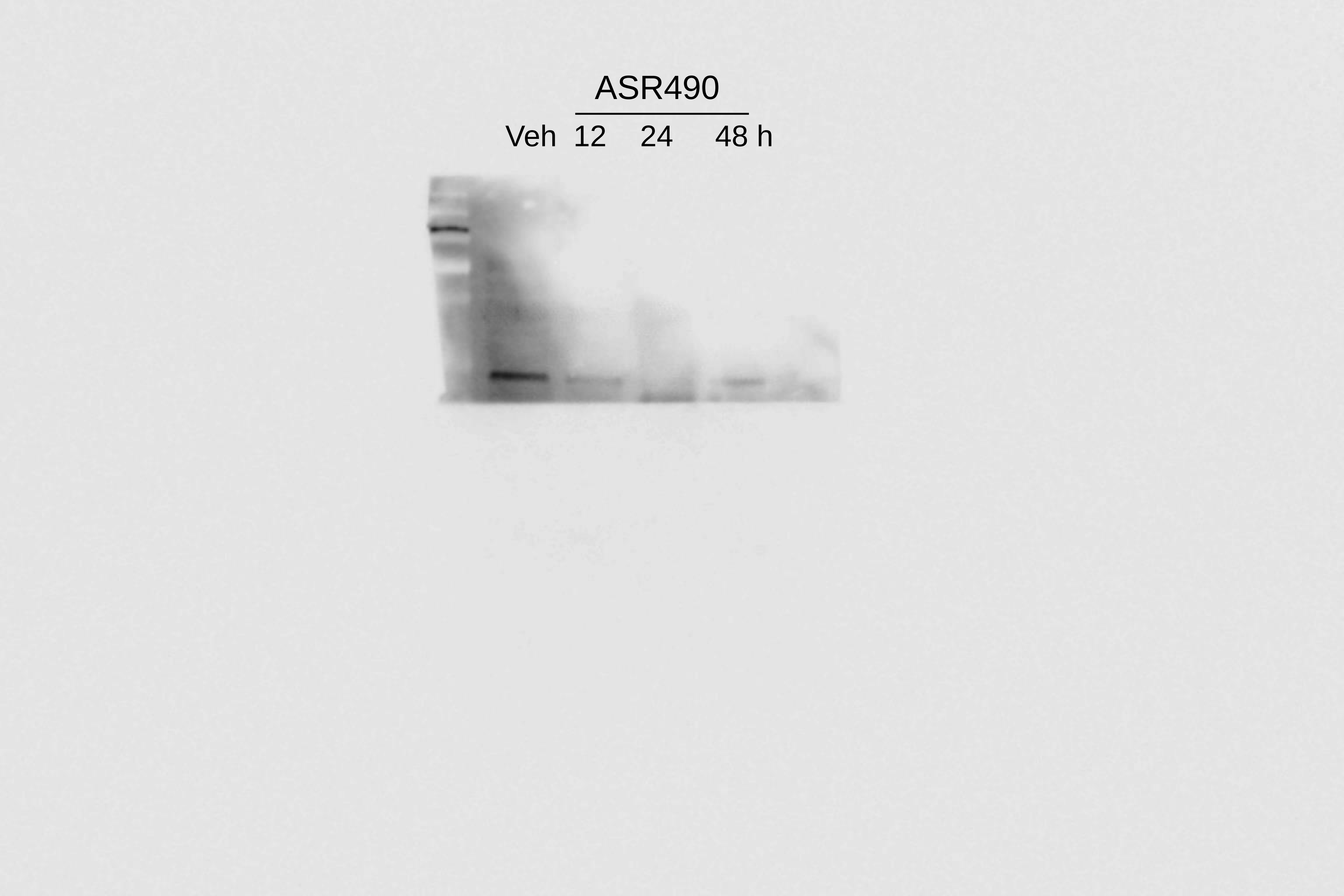

Actin

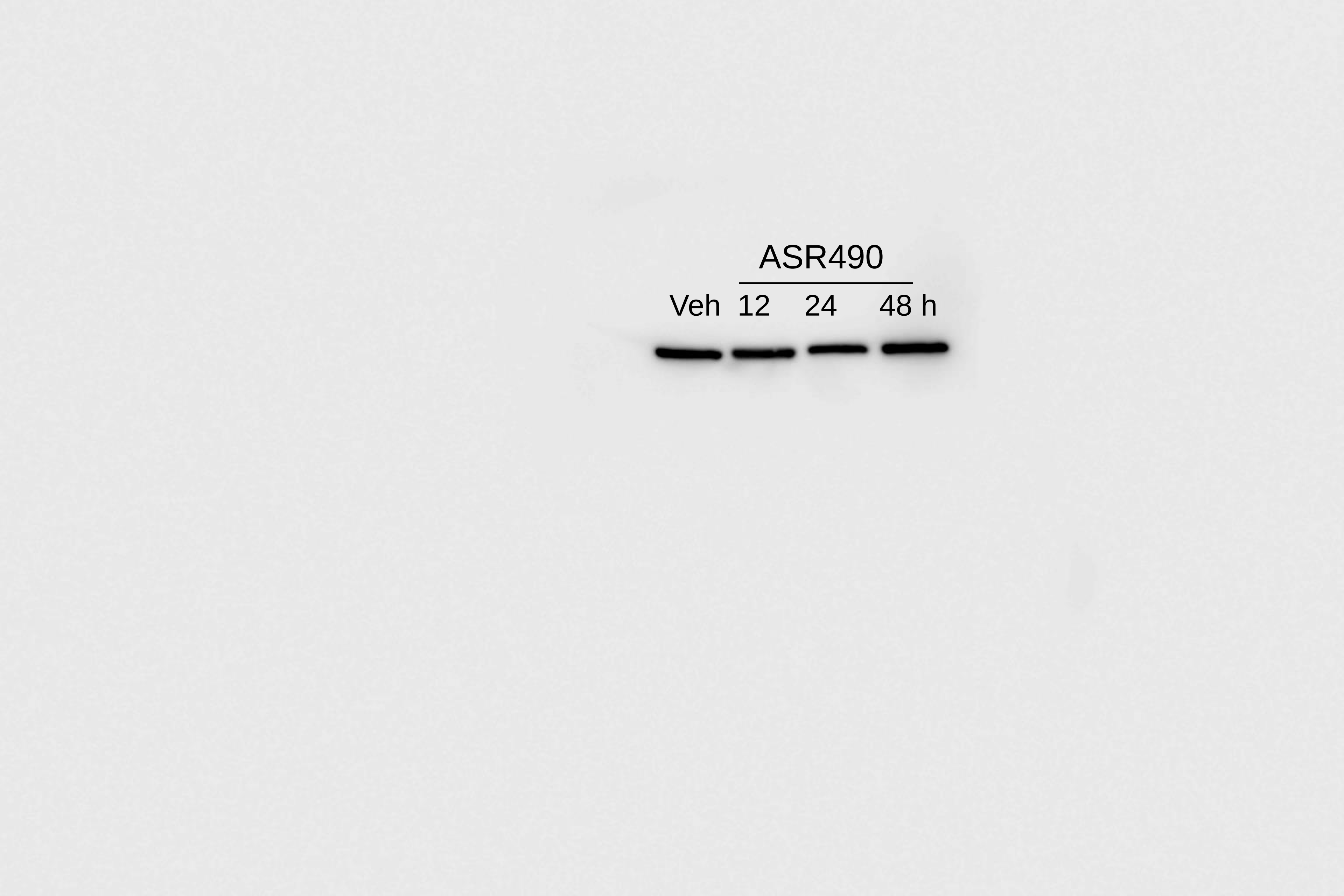

Figure 3A

NICD Notch1

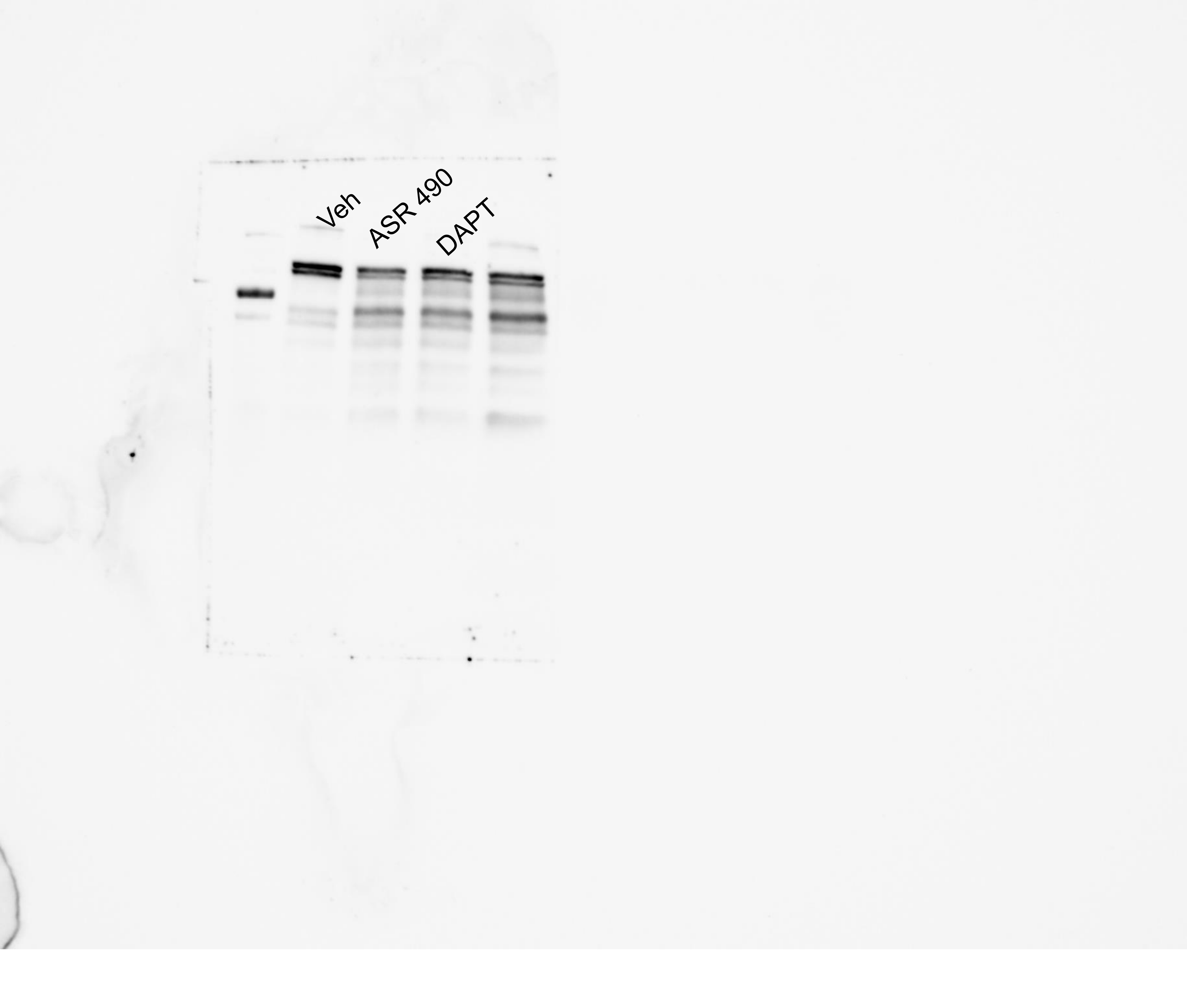

HES1

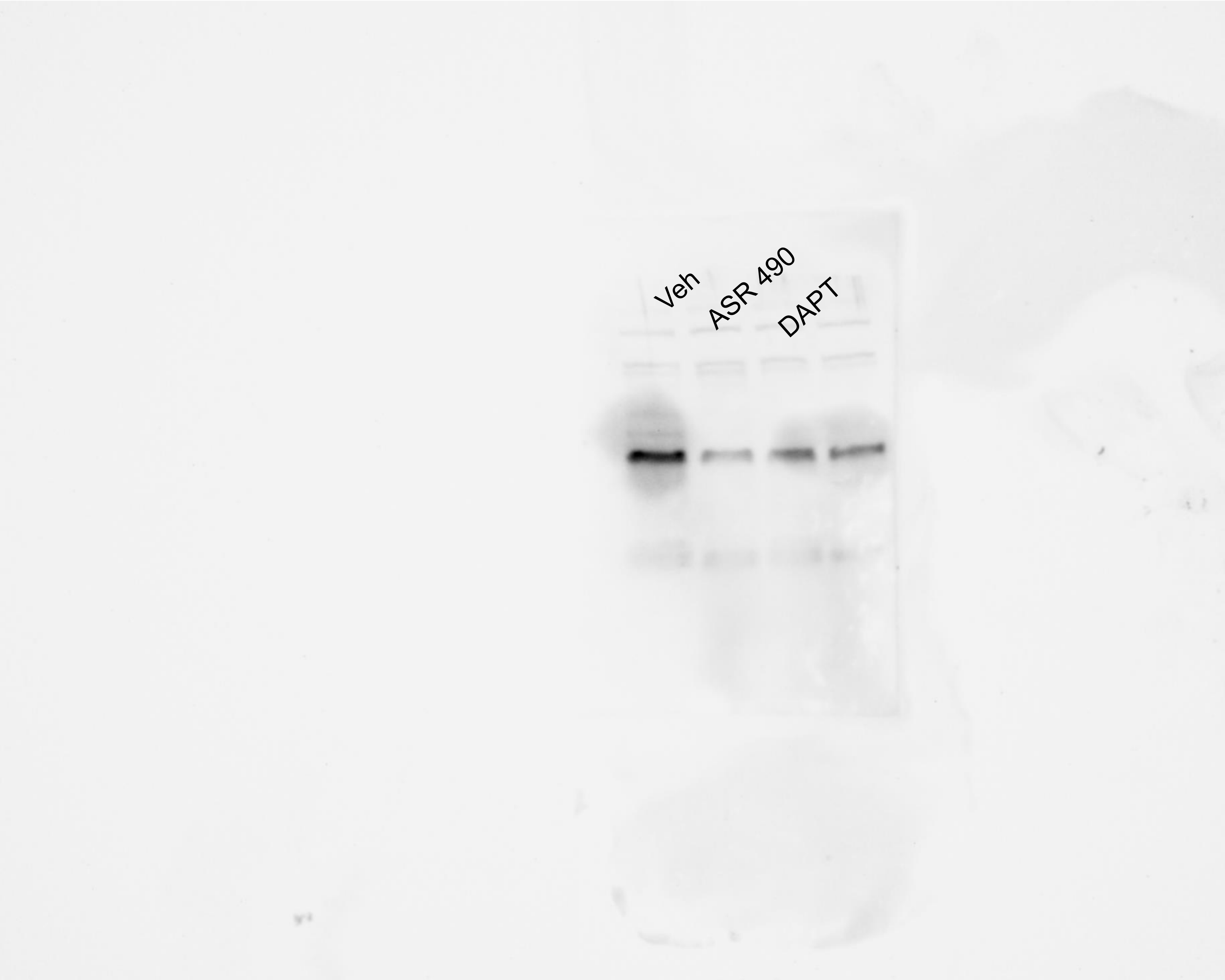

Actin

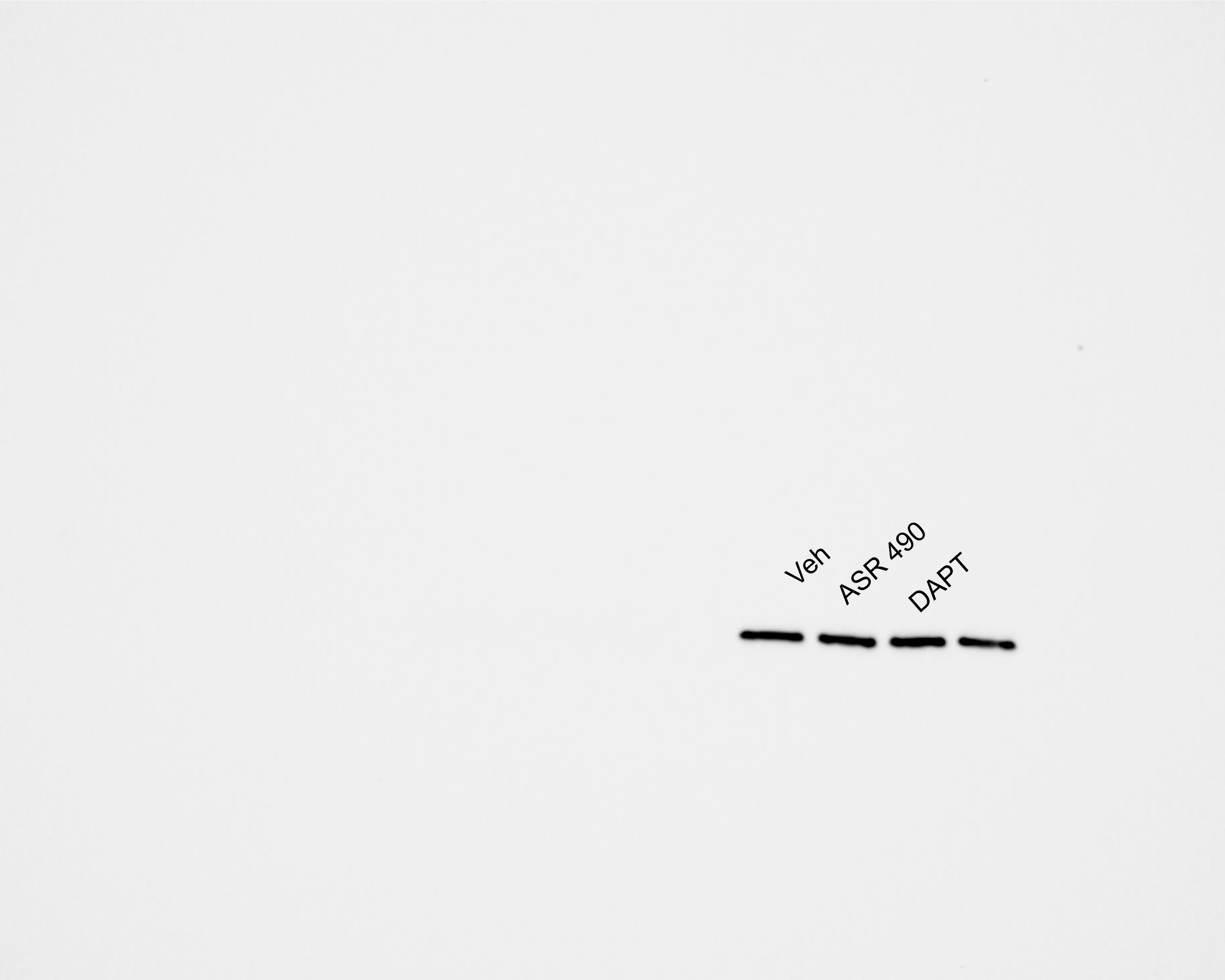

Notch 2

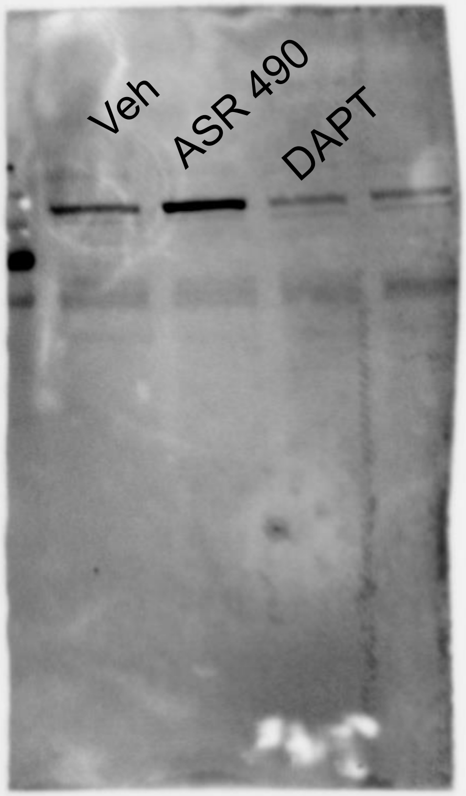

Actin

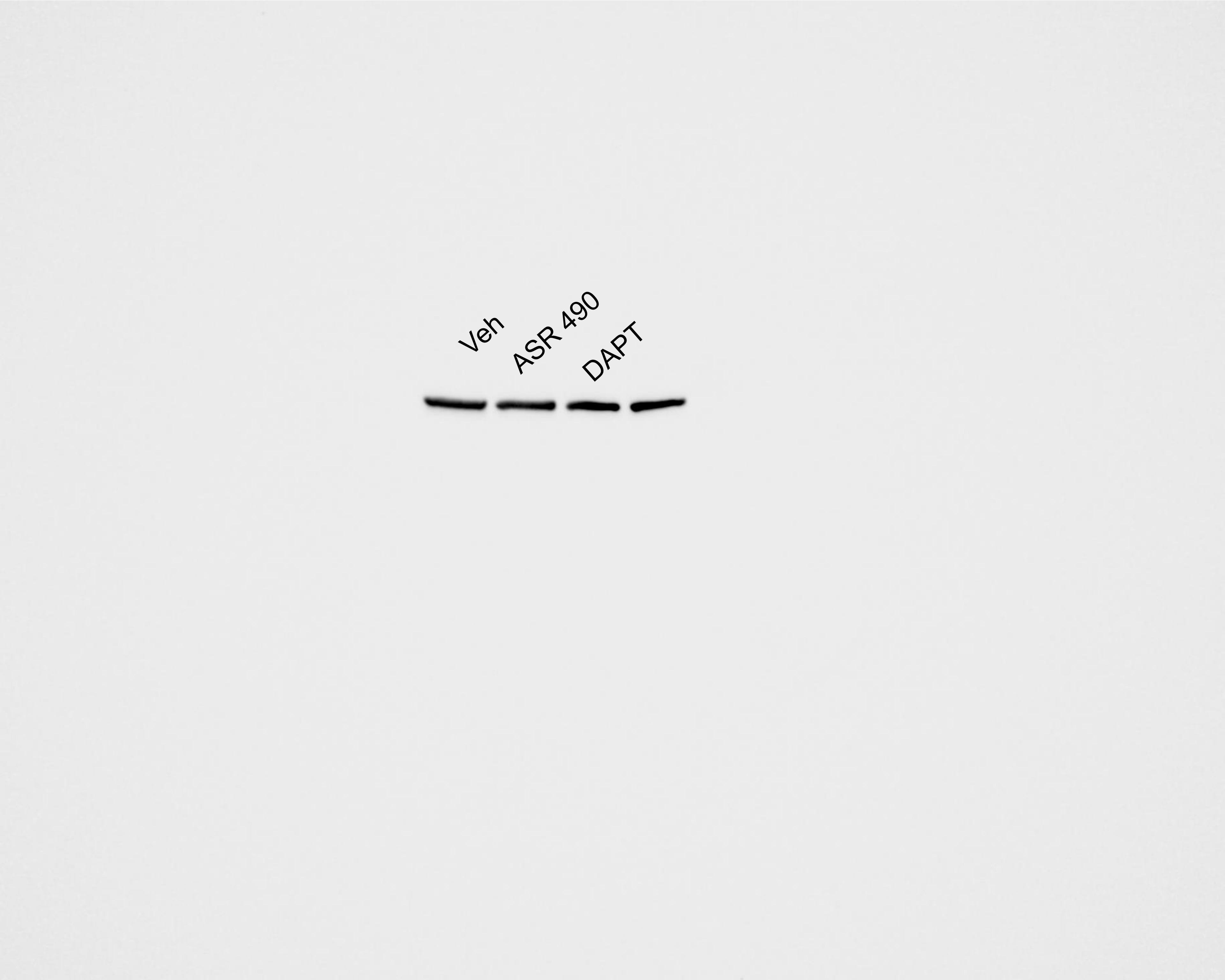

Figure 3D

NICD Notch1

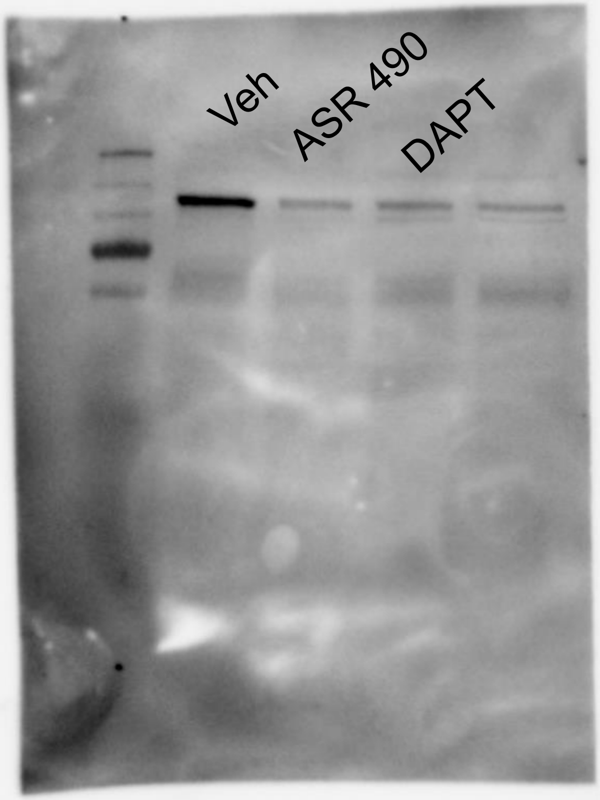

HES1

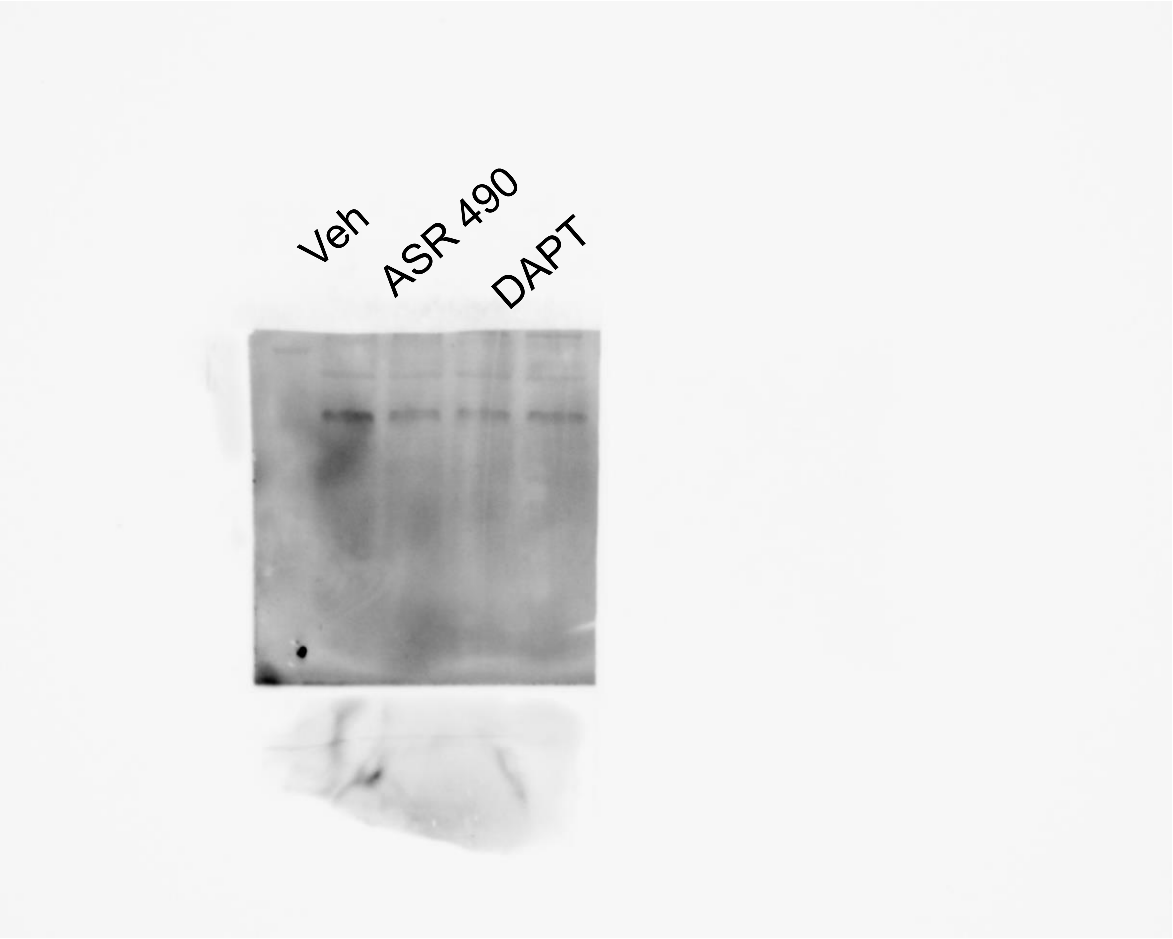

Actin

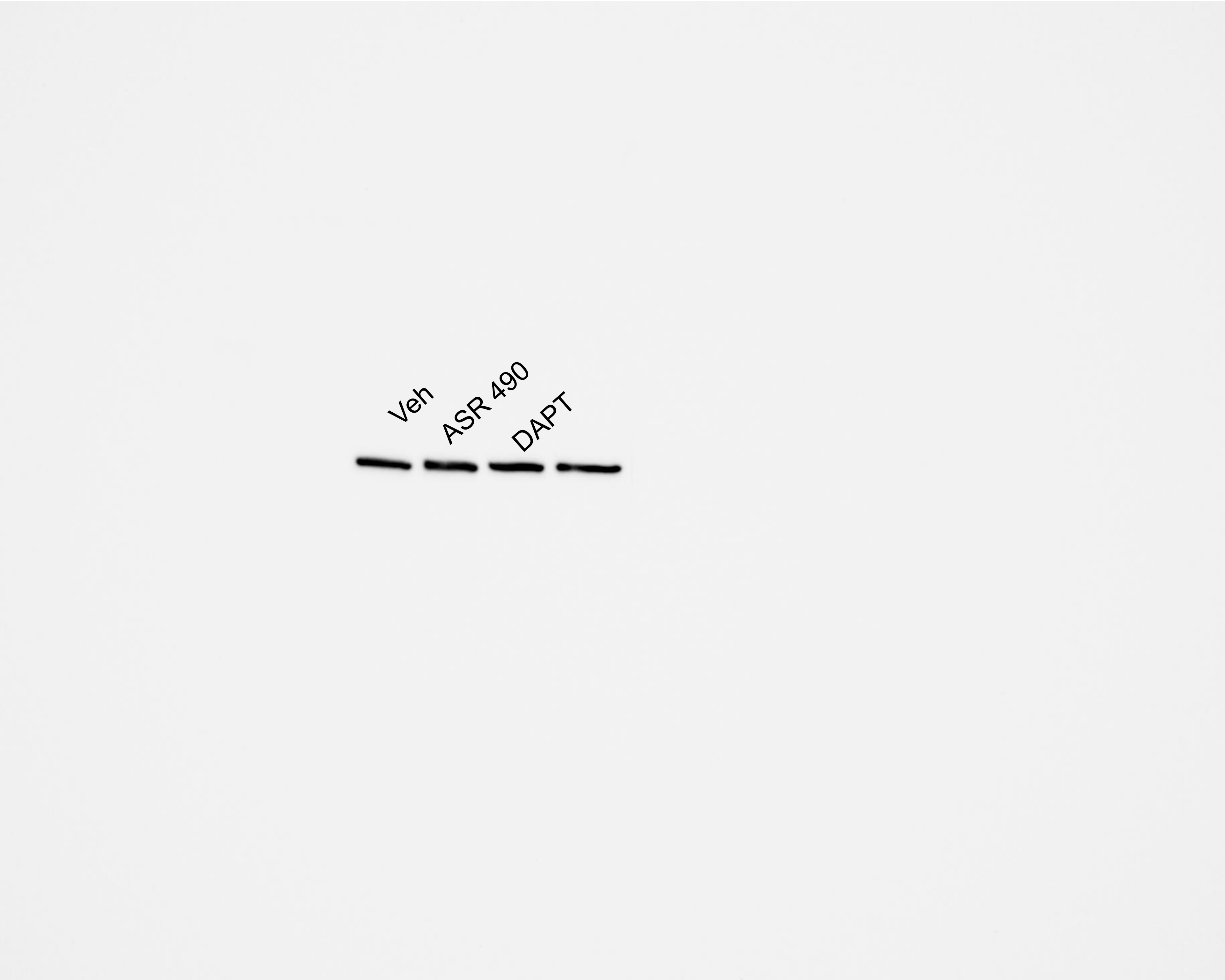

Notch2

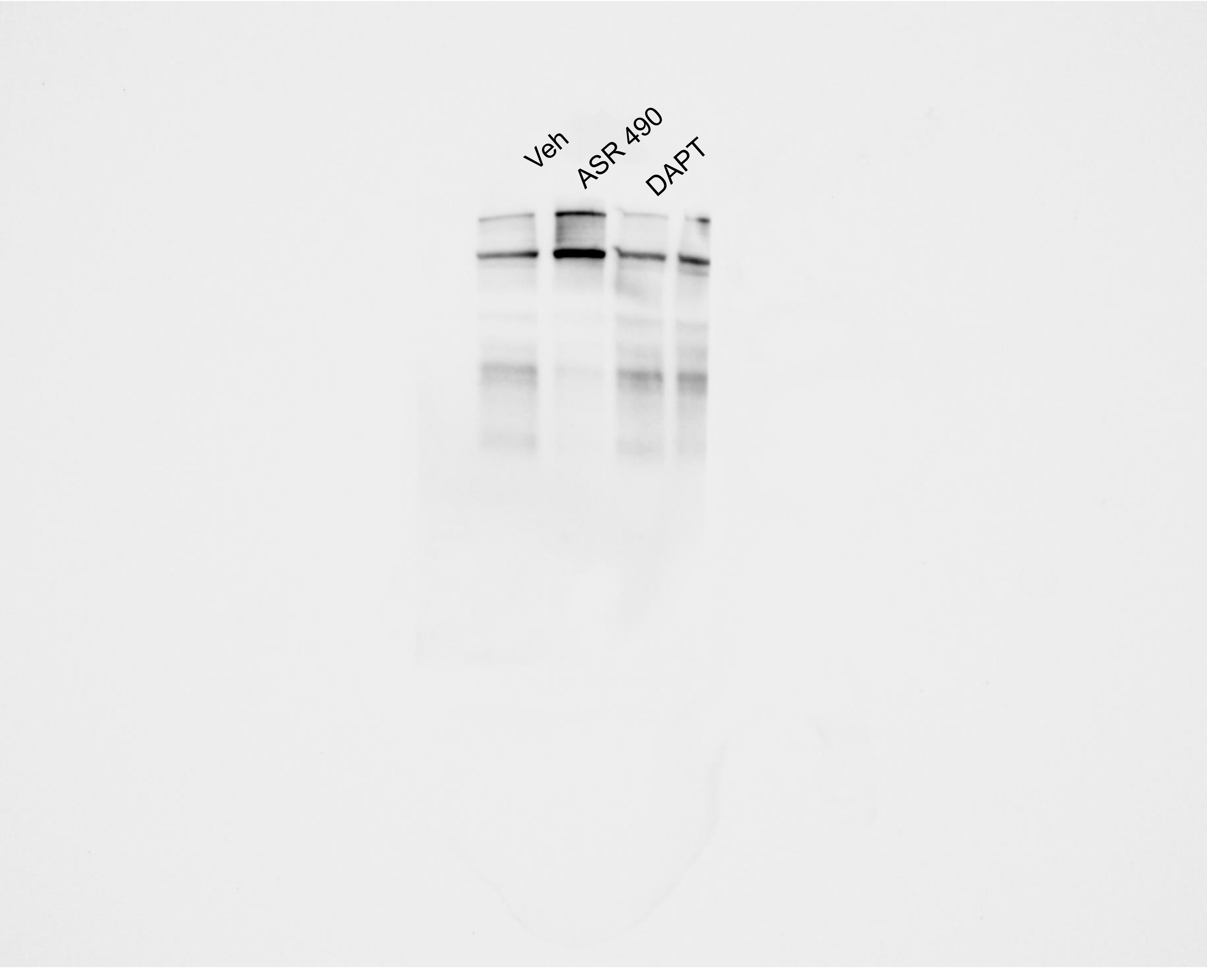

Actin

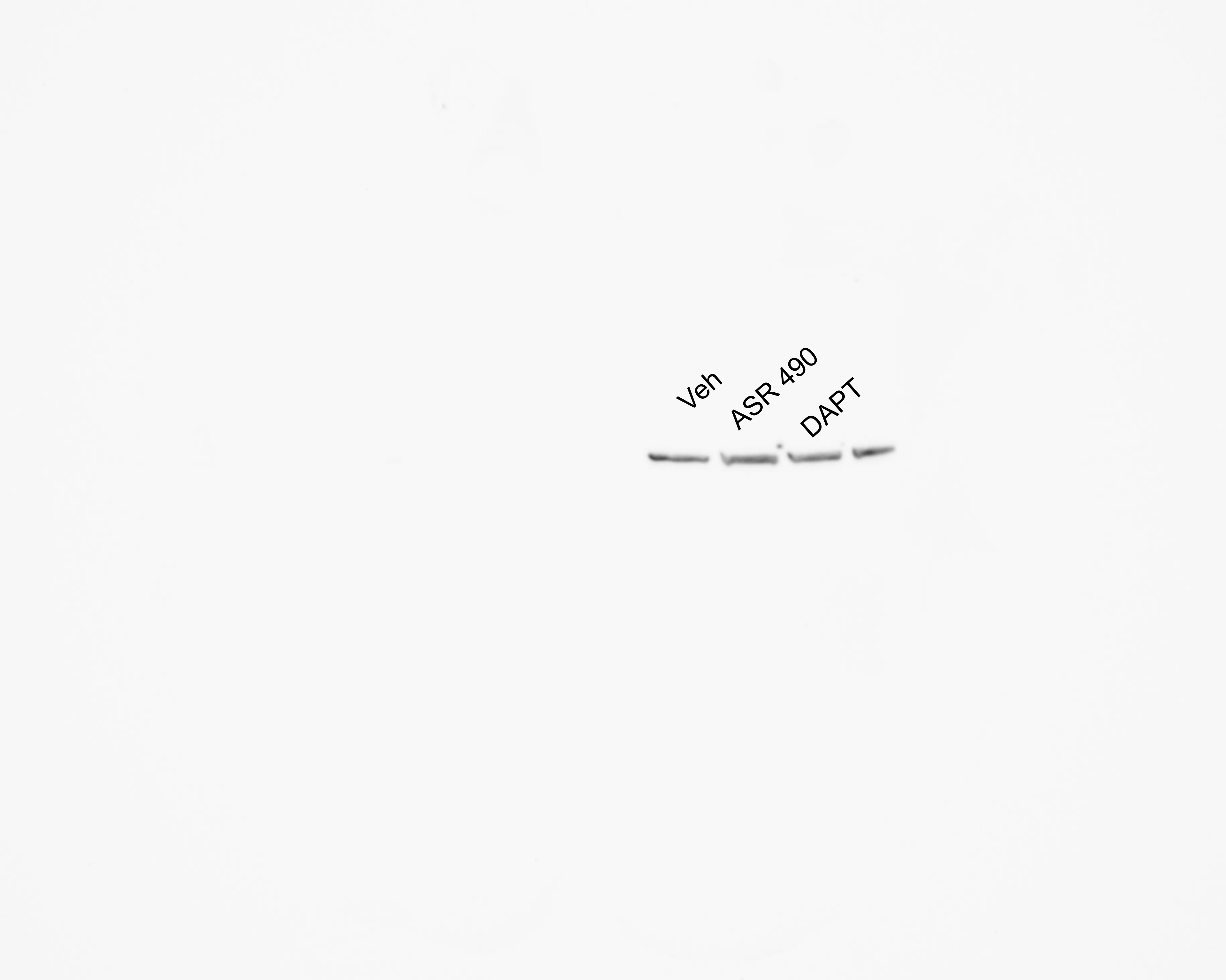

Figure 3J

NICD Notch1

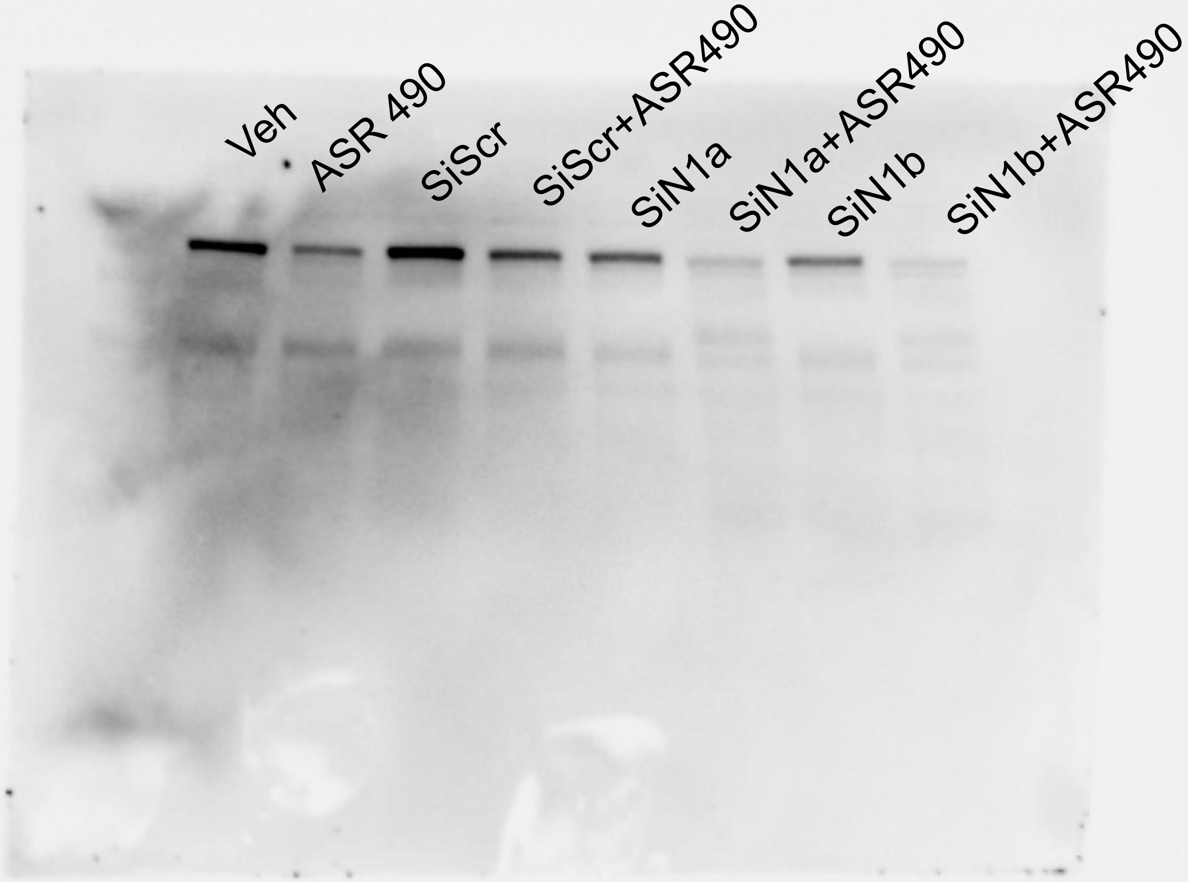

HES1

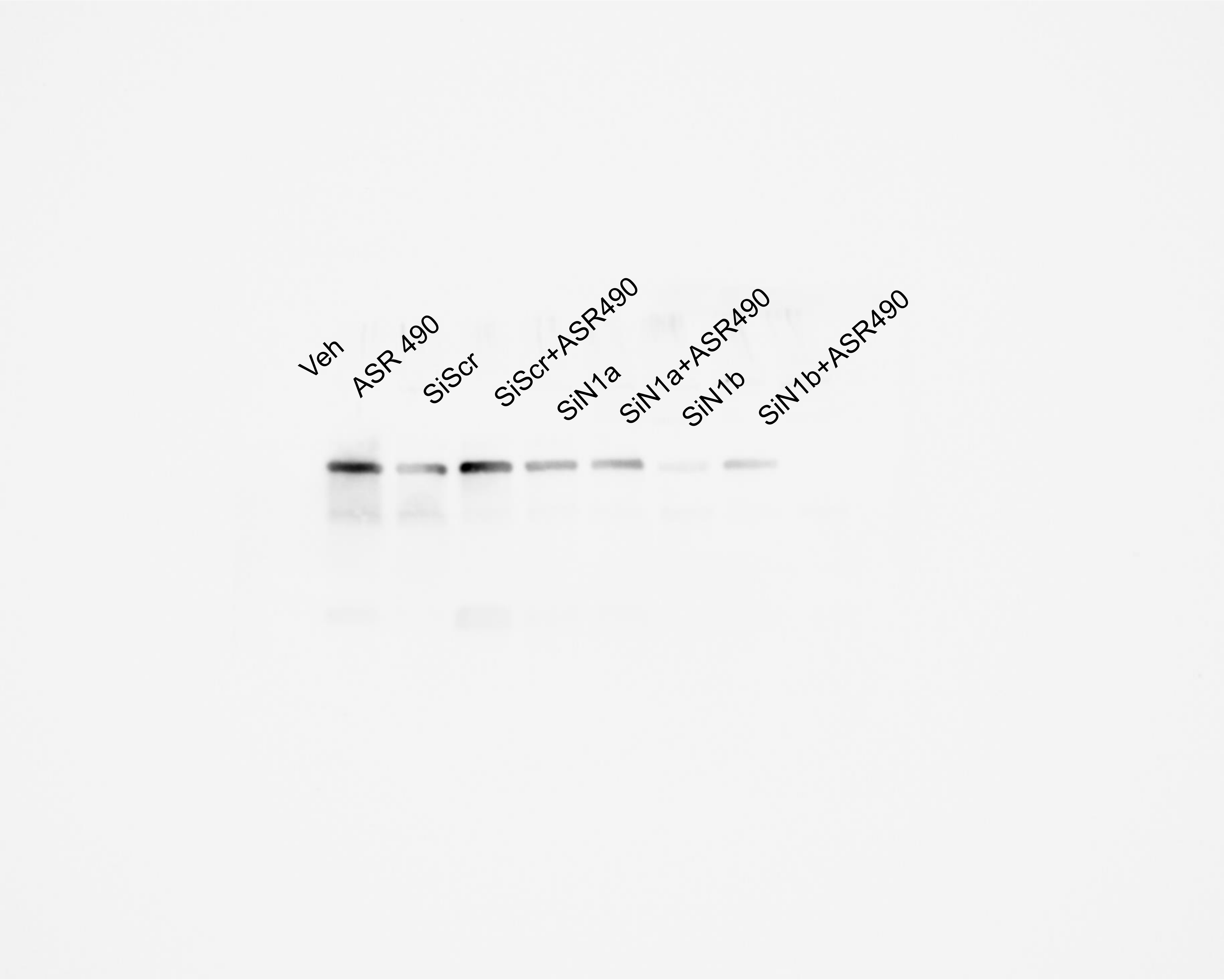

Veh
ASR 490
SiScr
SiScr+ASR490
SiN1a
SiN1a+ASR490
SiN1b
SiN1b+ASR490

Actin

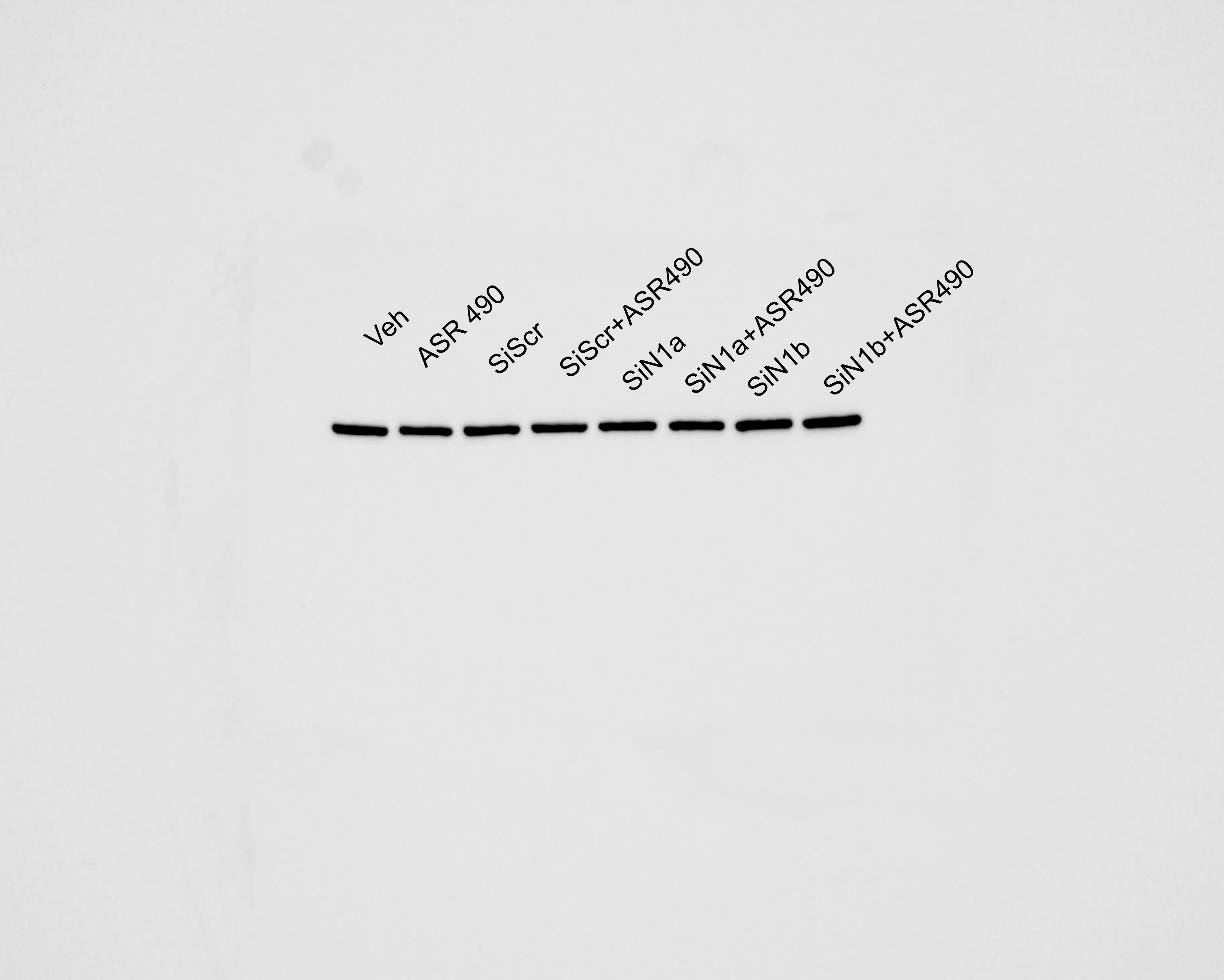
Veh
ASR 490
SiScr
SiScr+ASR490
SiN1a
SiN1a+ASR490
SiN1b
SiN1b+ASR490

Figure 4A

NCID Notch1

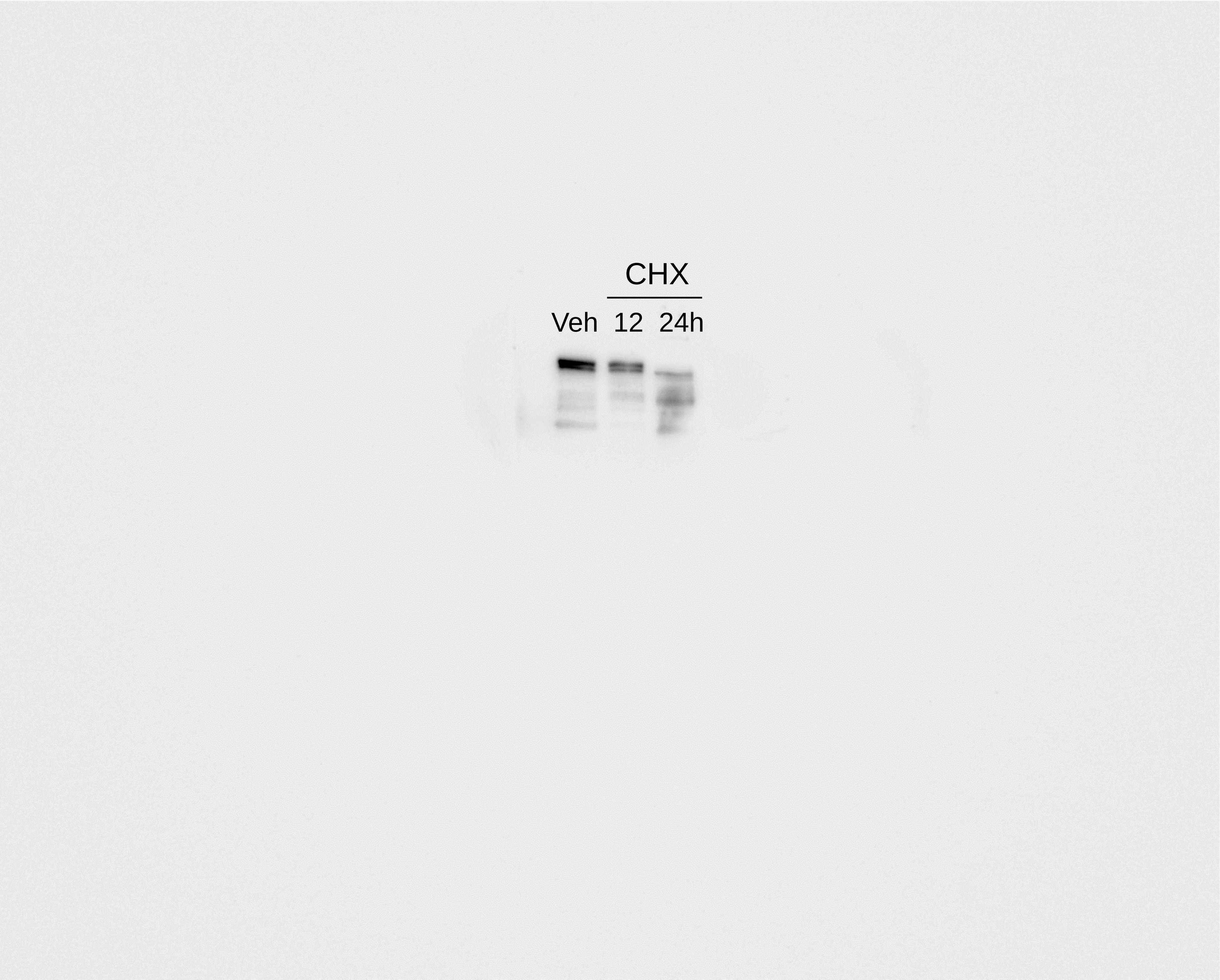

Actin

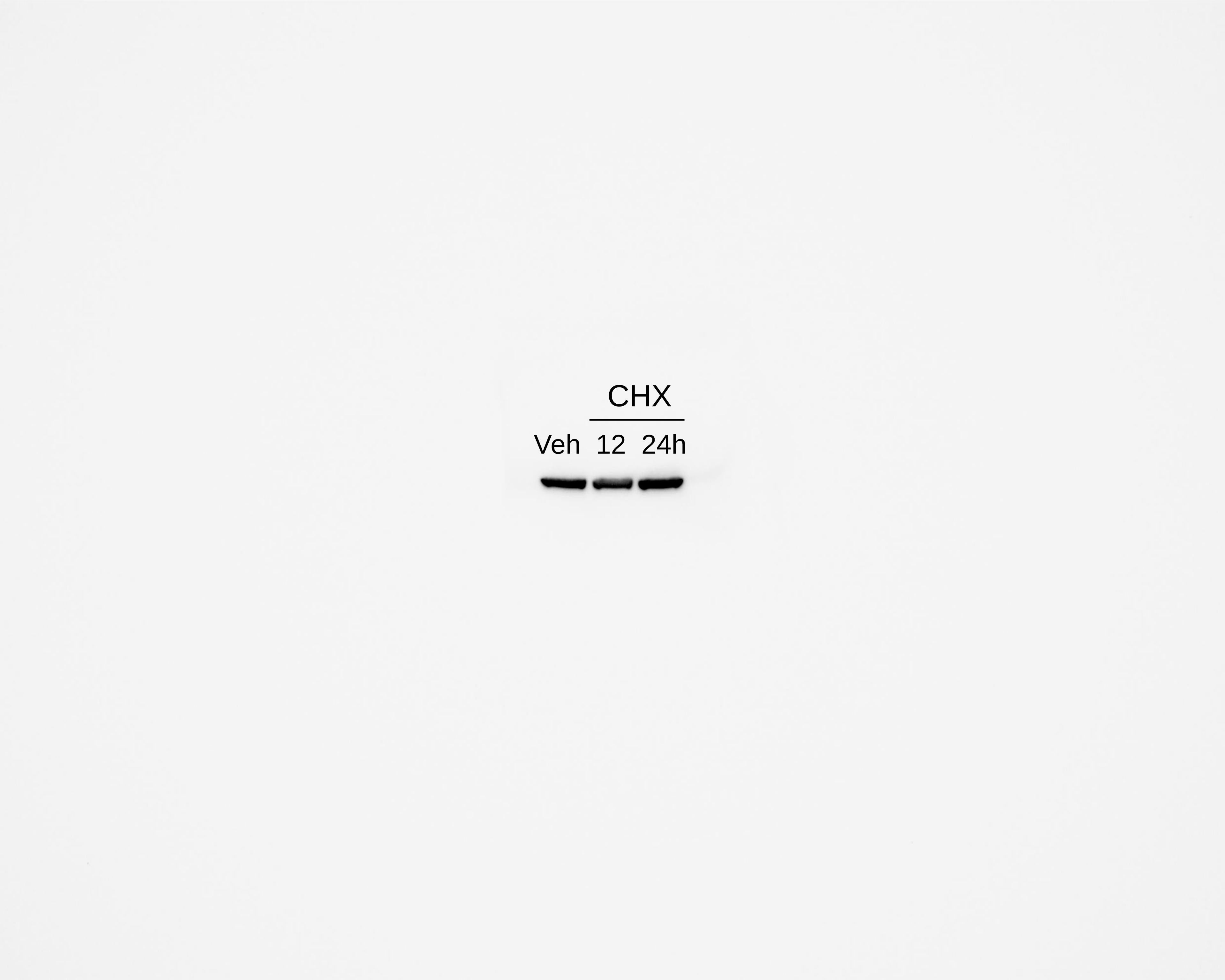

NCID Notch1

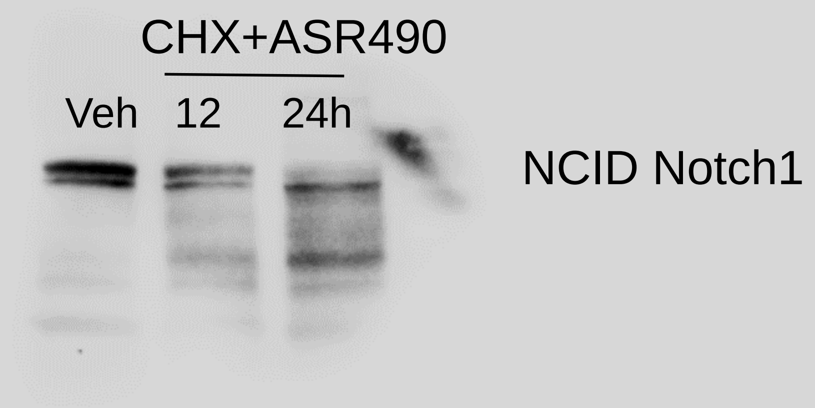

Actin

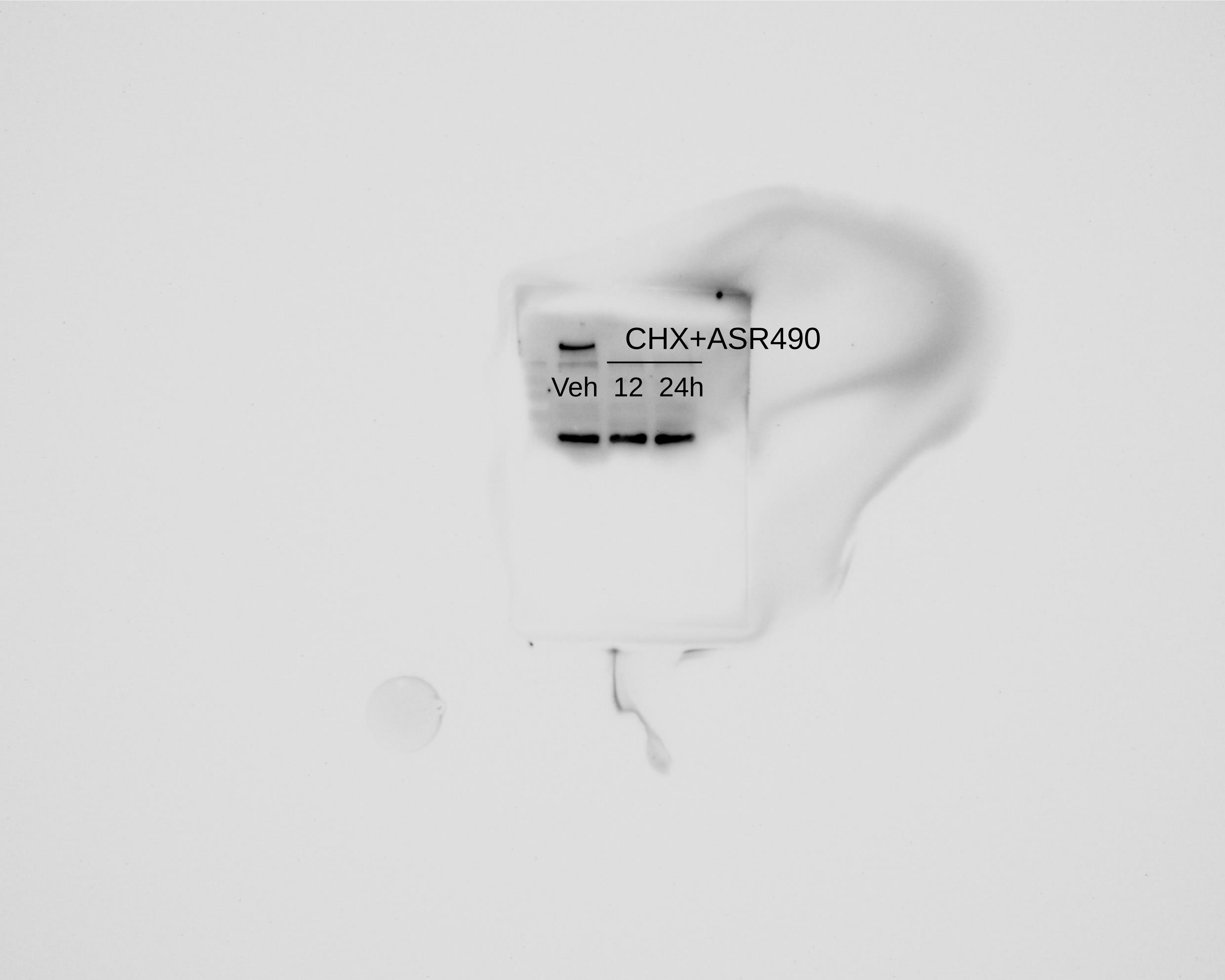

Figure 4B

NCID Notch1

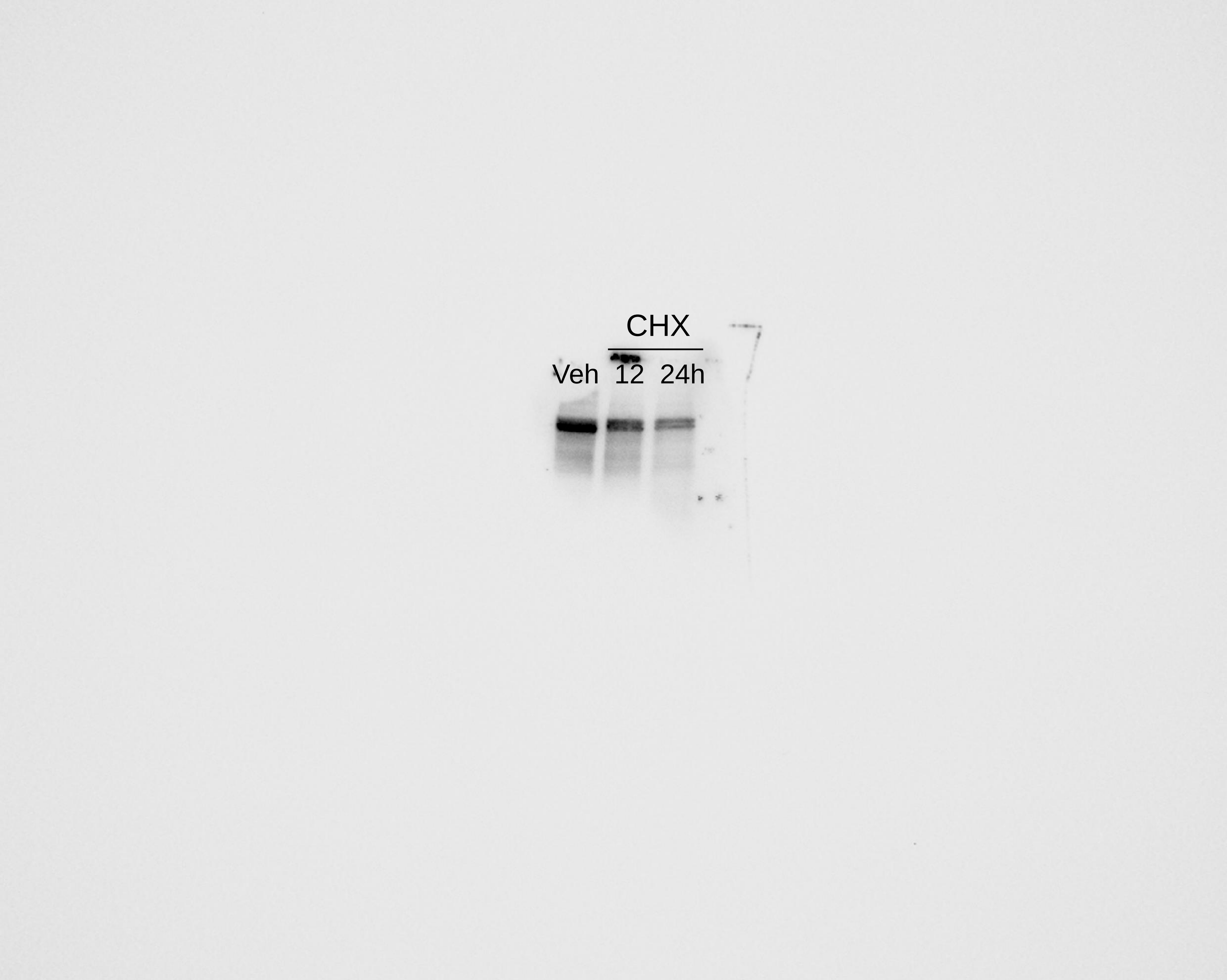

Actin

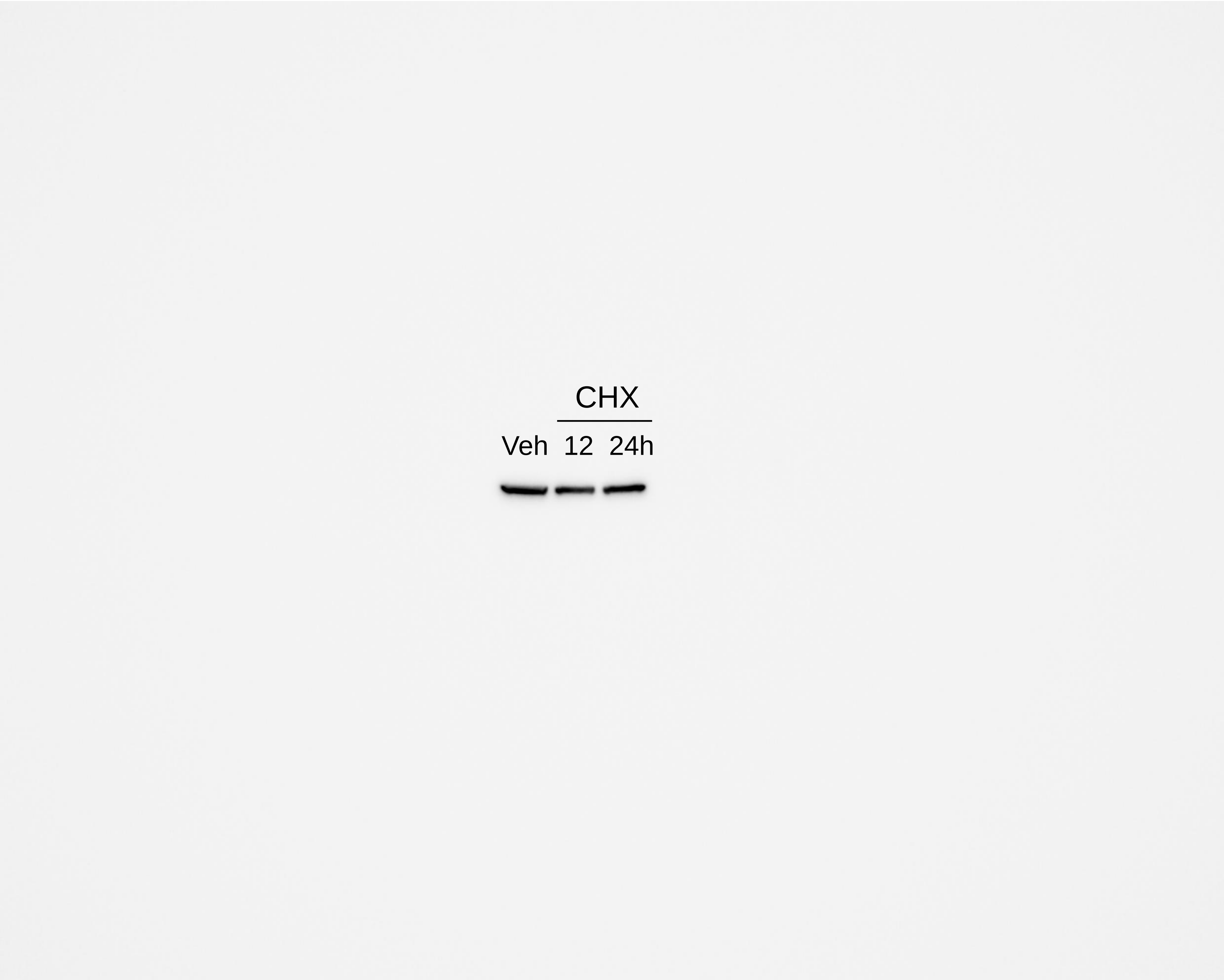

NCID Notch1

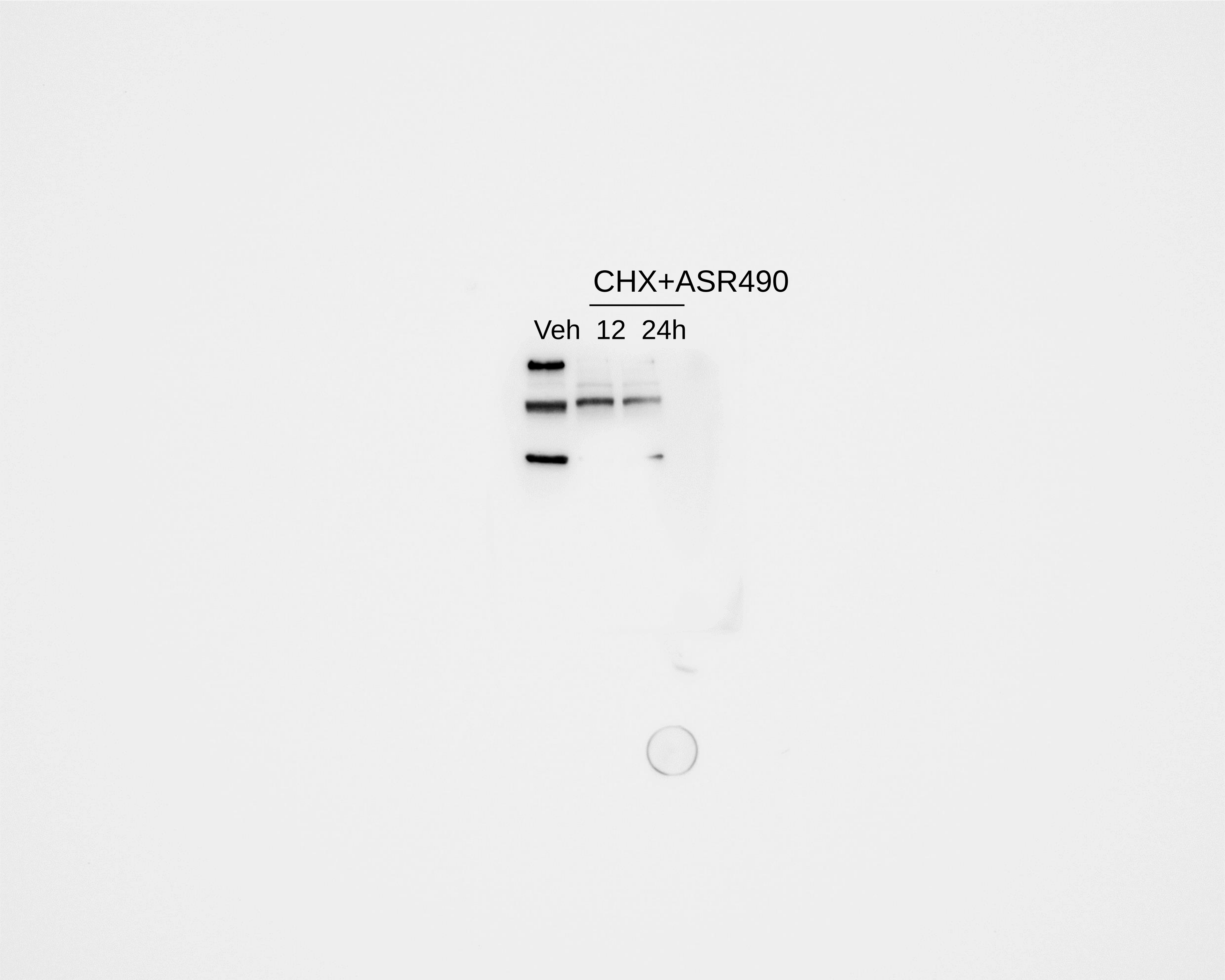

Actin

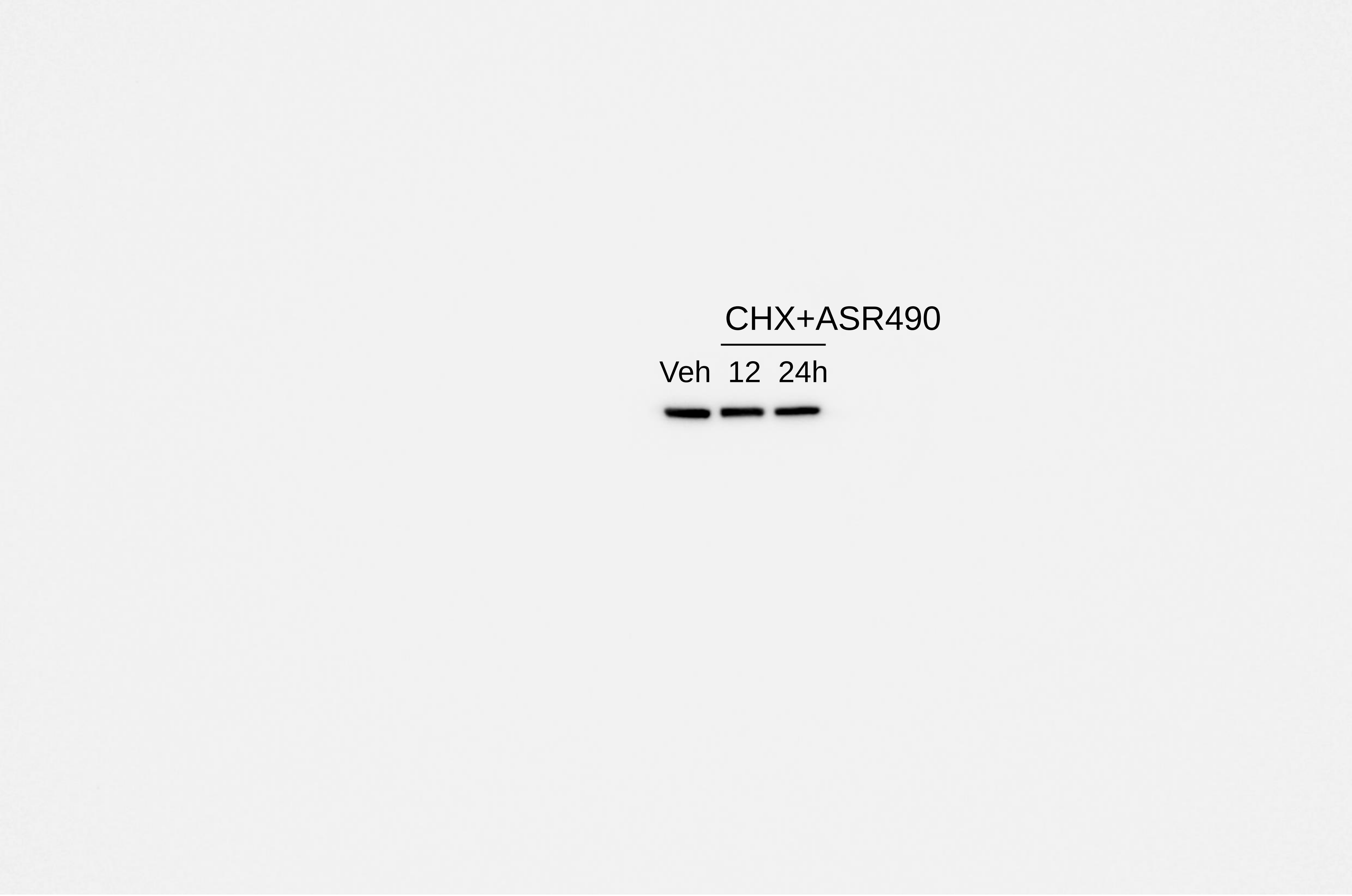

Figure 4D

NCID
Notch1

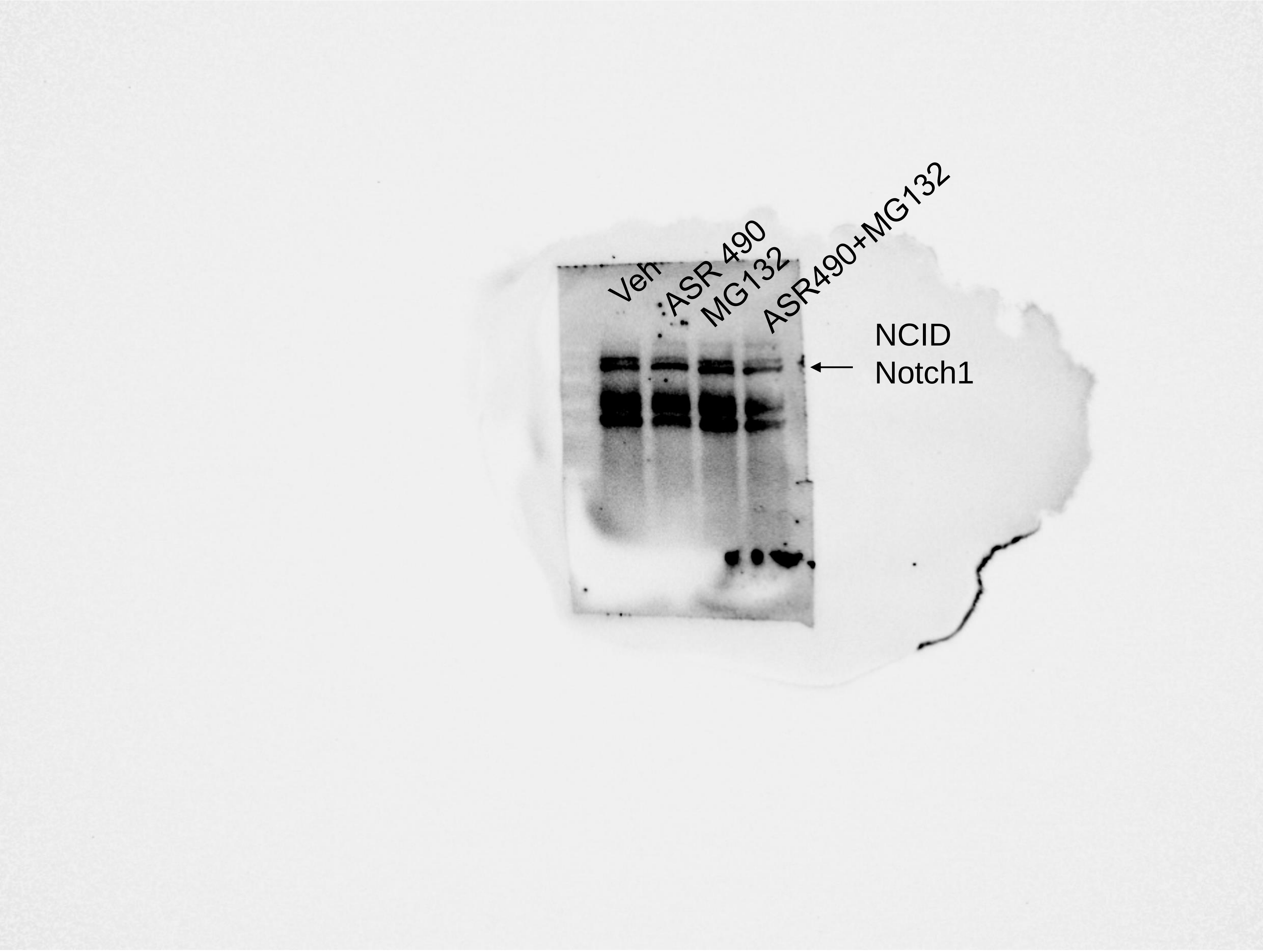

Actin

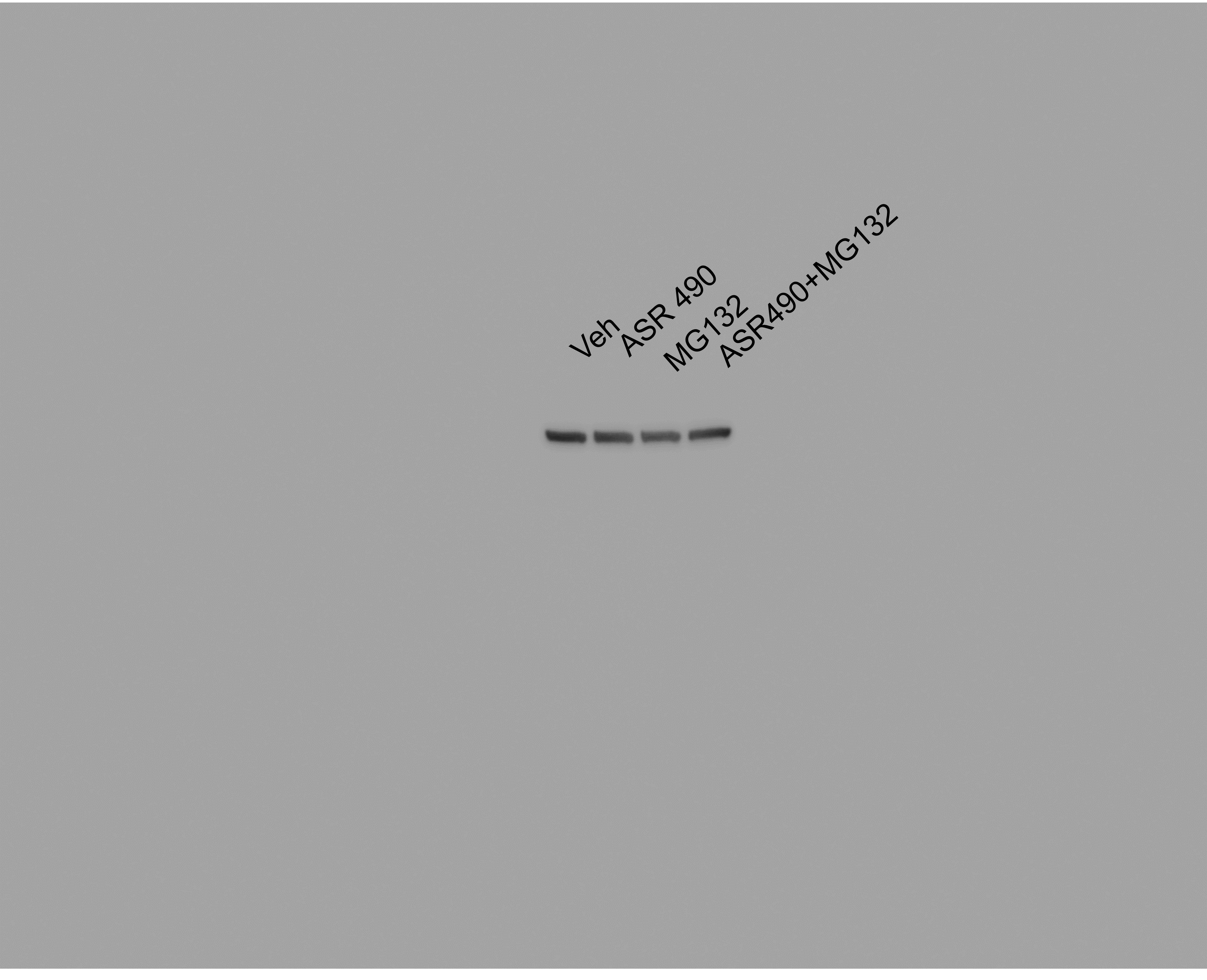

Figure 4E

NCID
Notch1

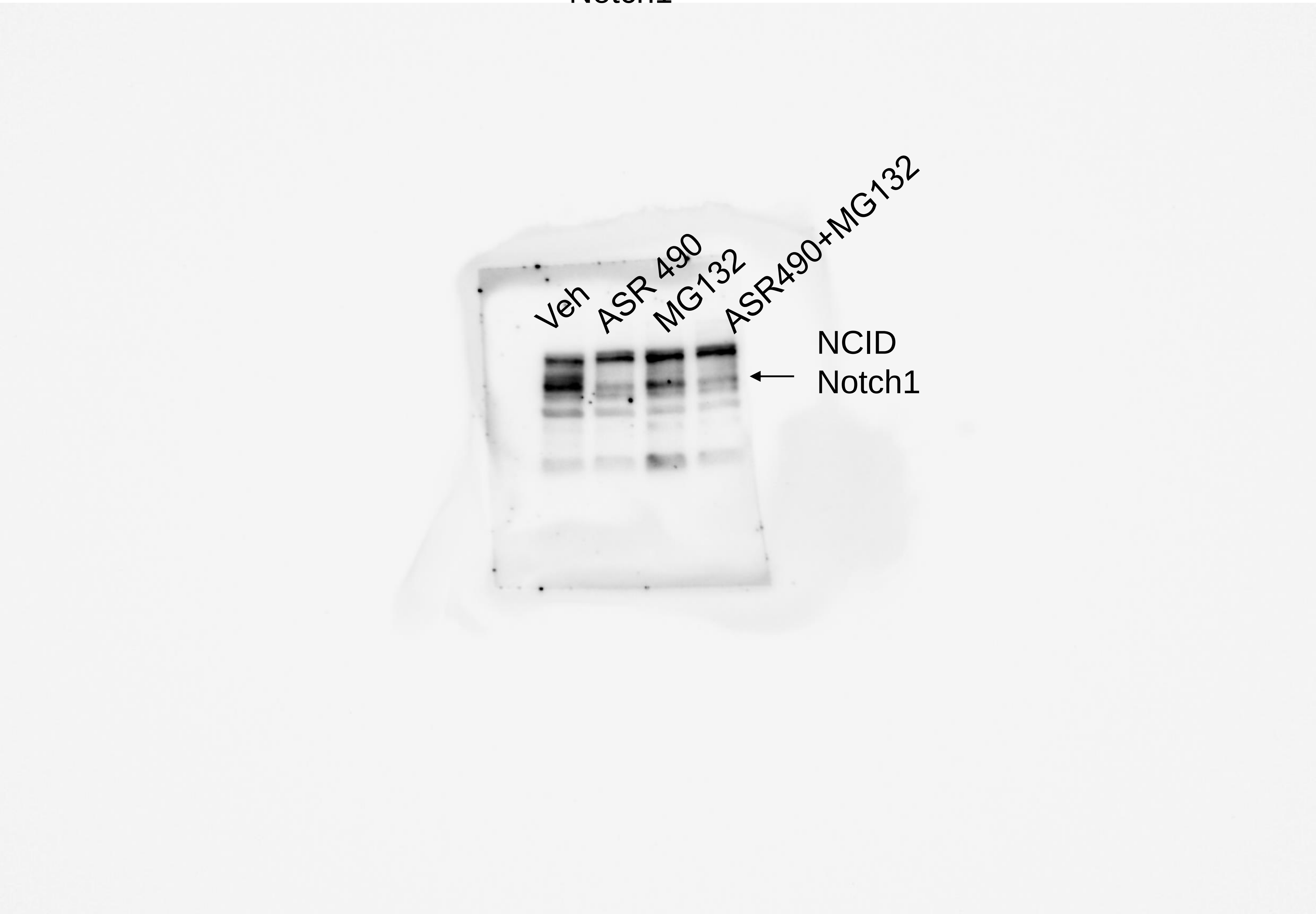

Actin

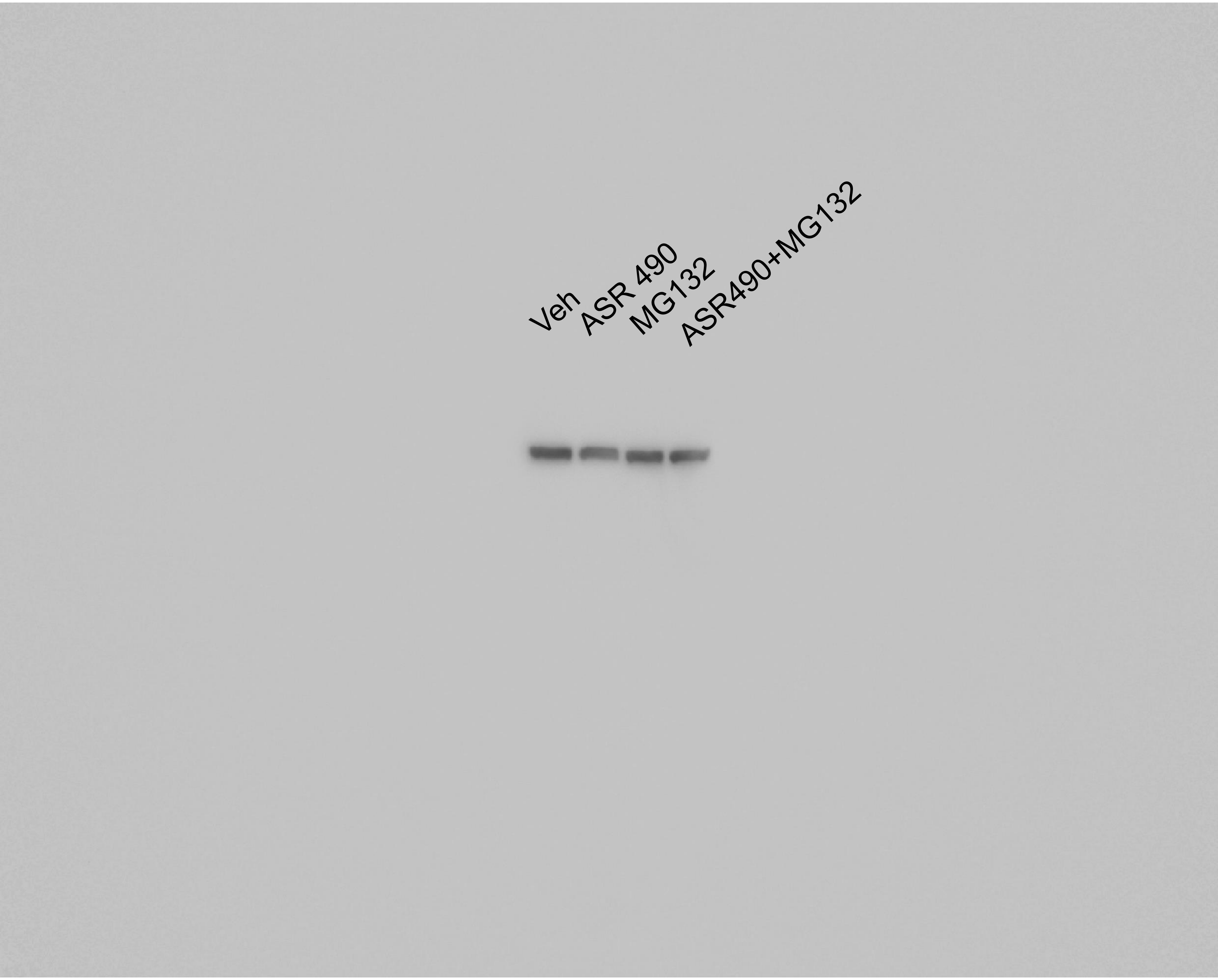

Figure 4F

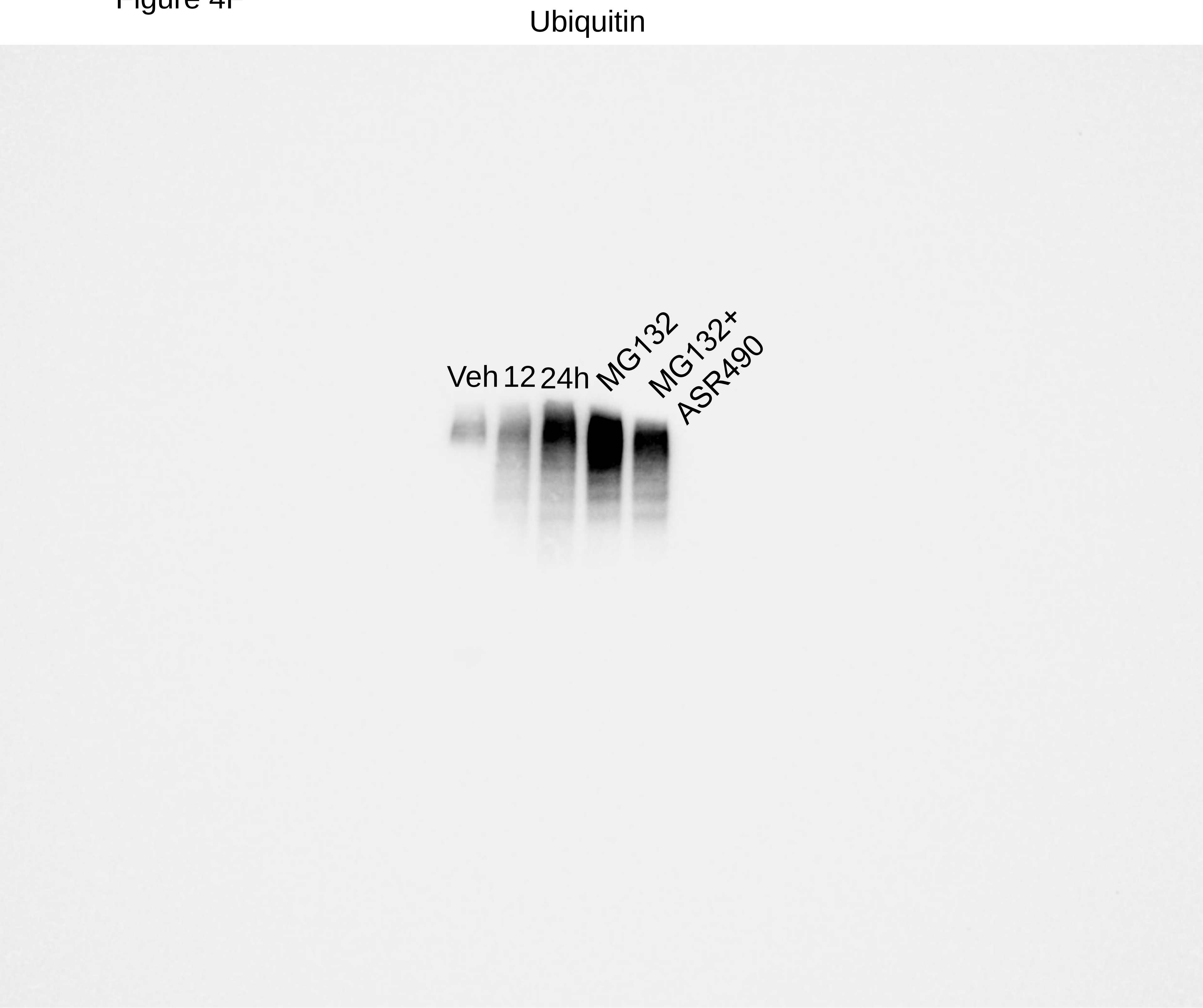

Actin

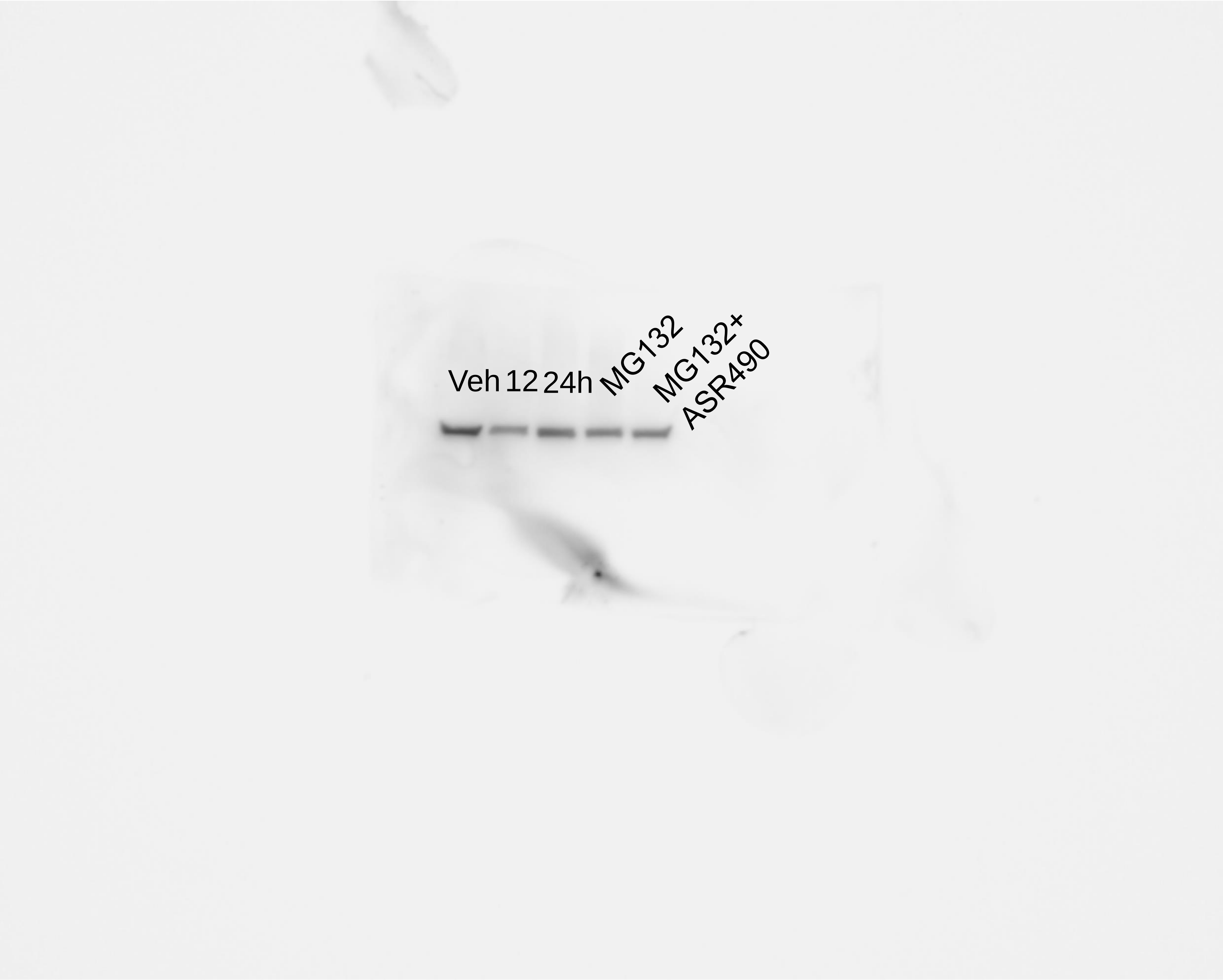

Figure 4G

Ubiquitin

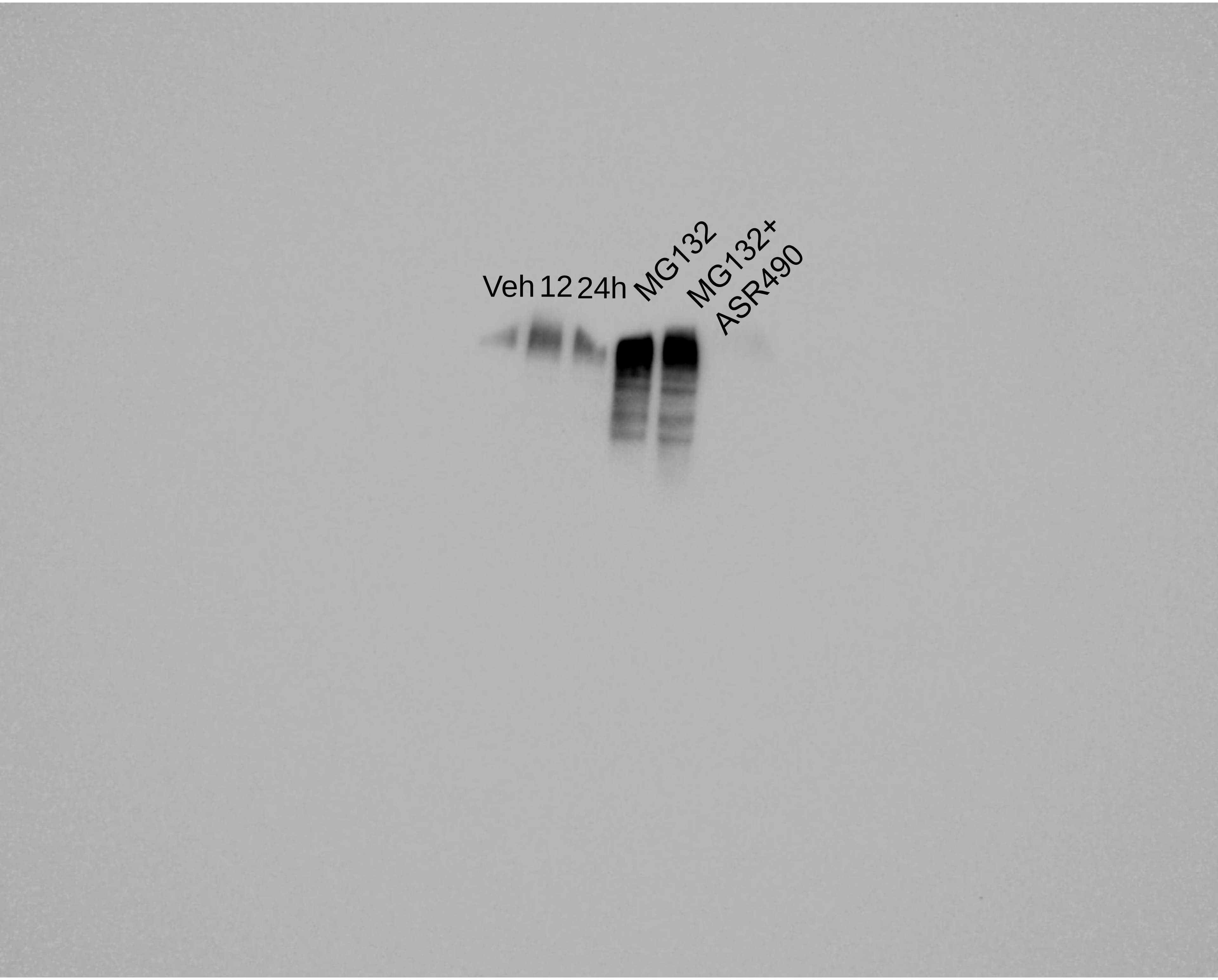

Actin

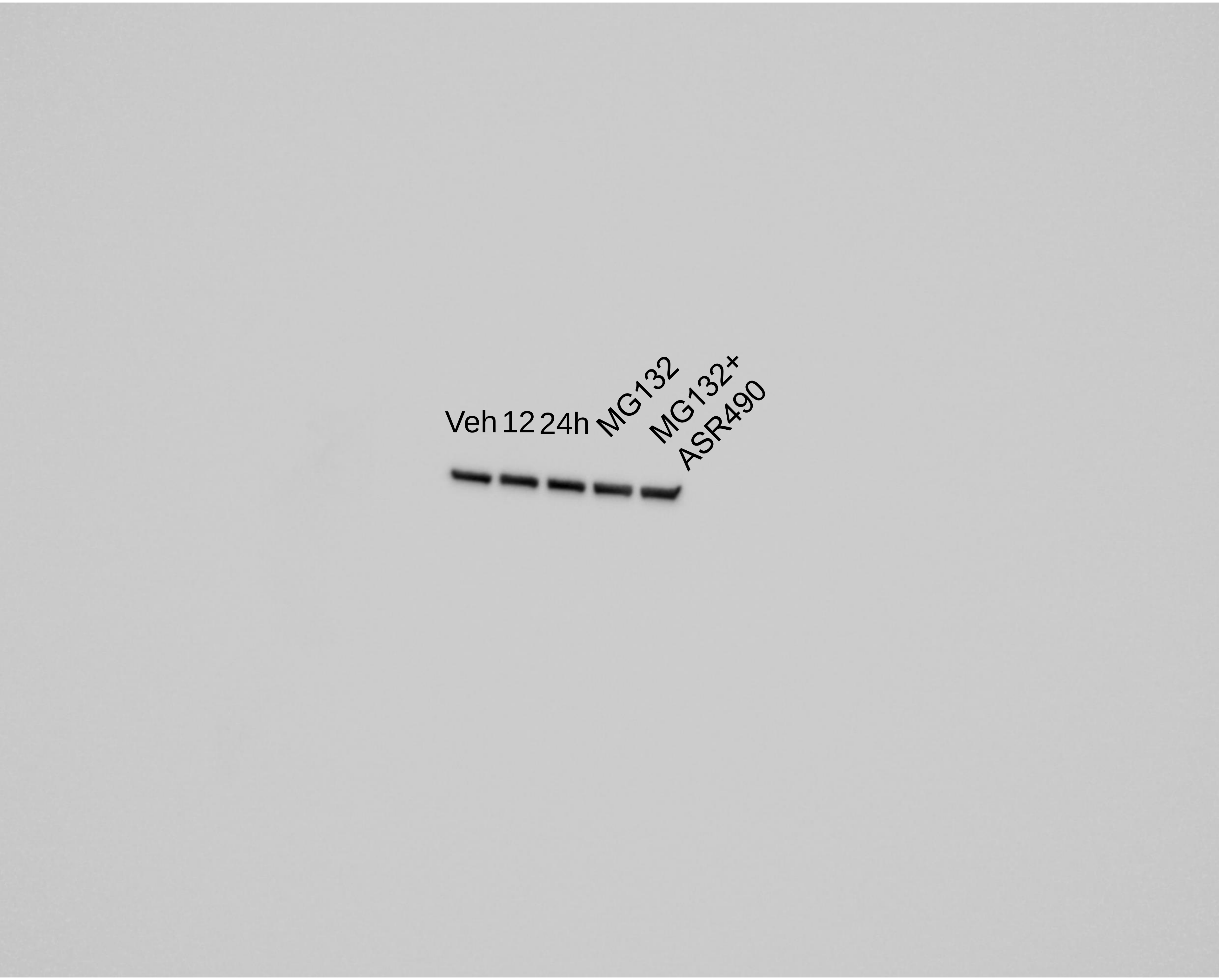

Figure 5A

NICD Notch1

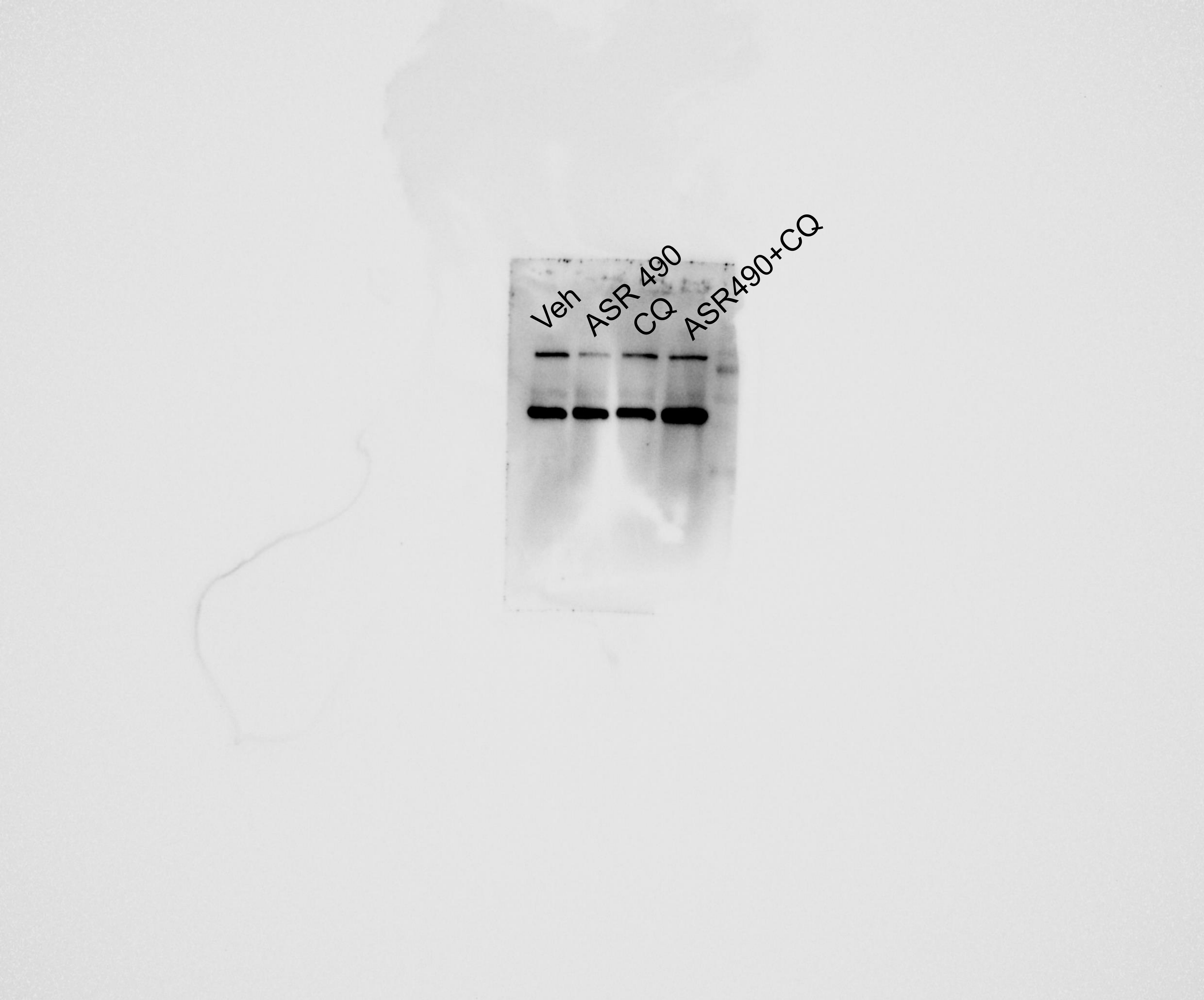

Actin

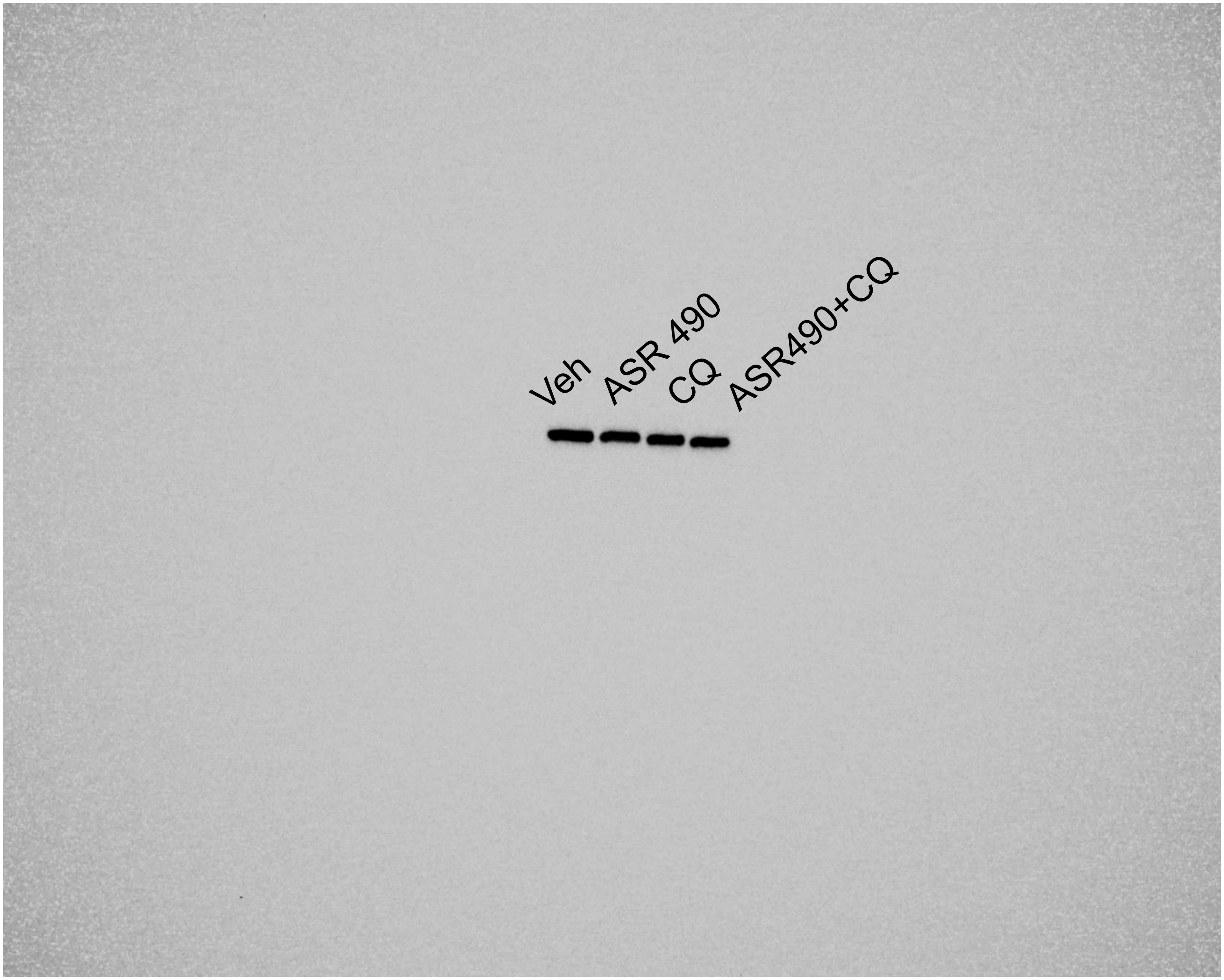

Figure 5B

NICD Notch1

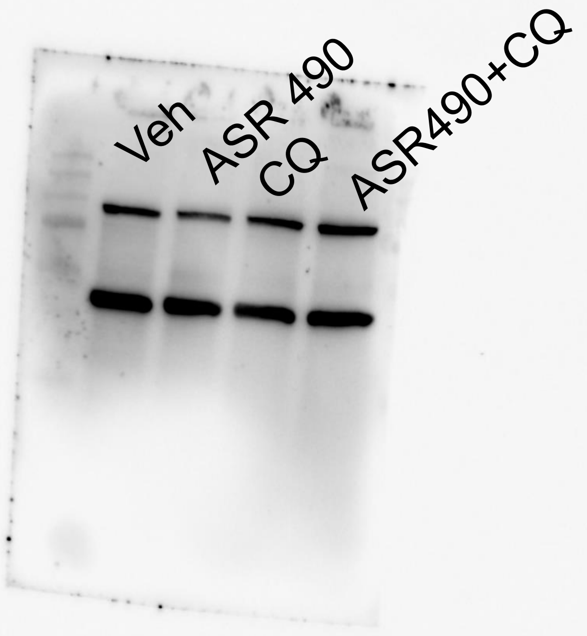

Figure 5B

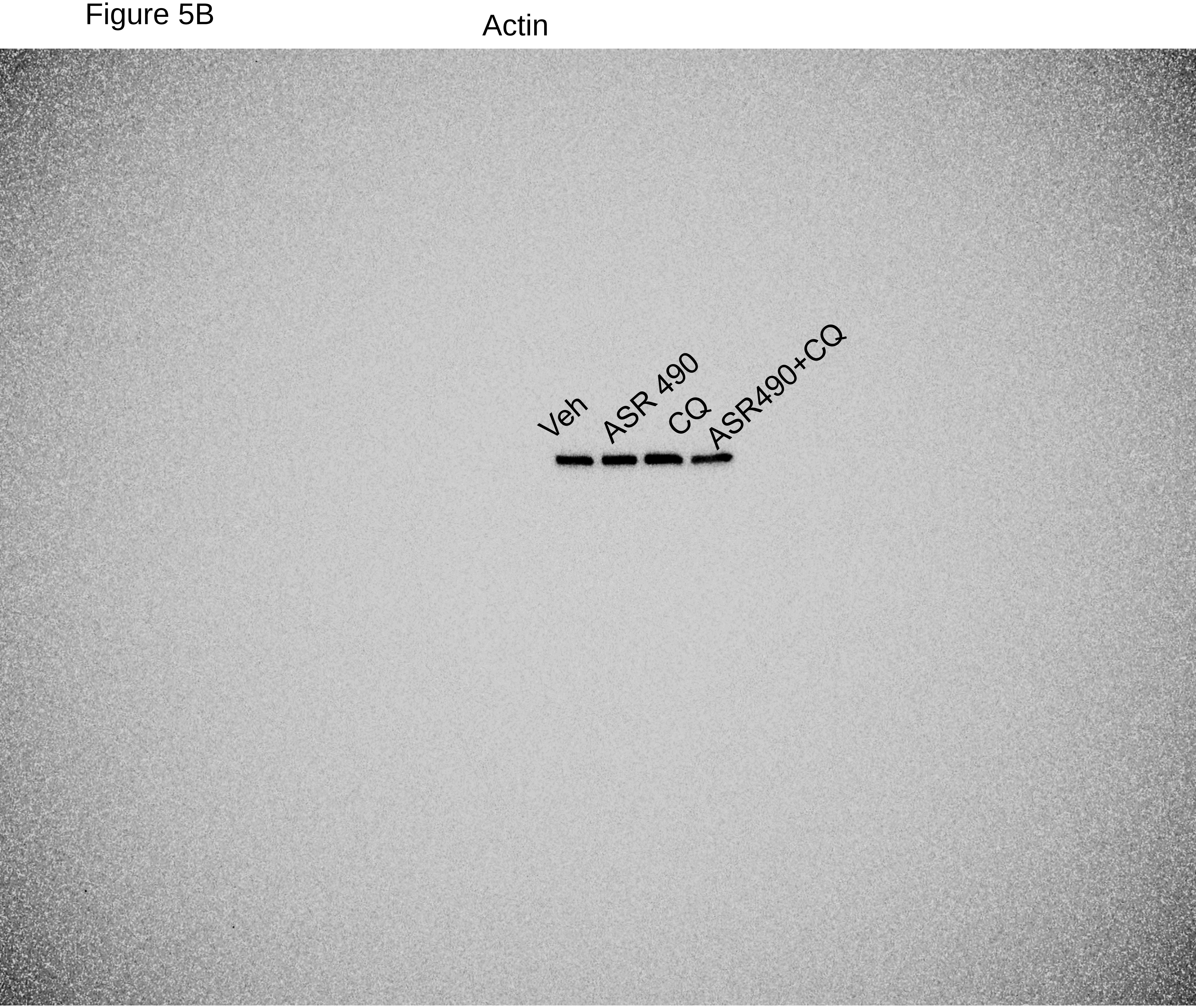

Figure 5E

Lamp1

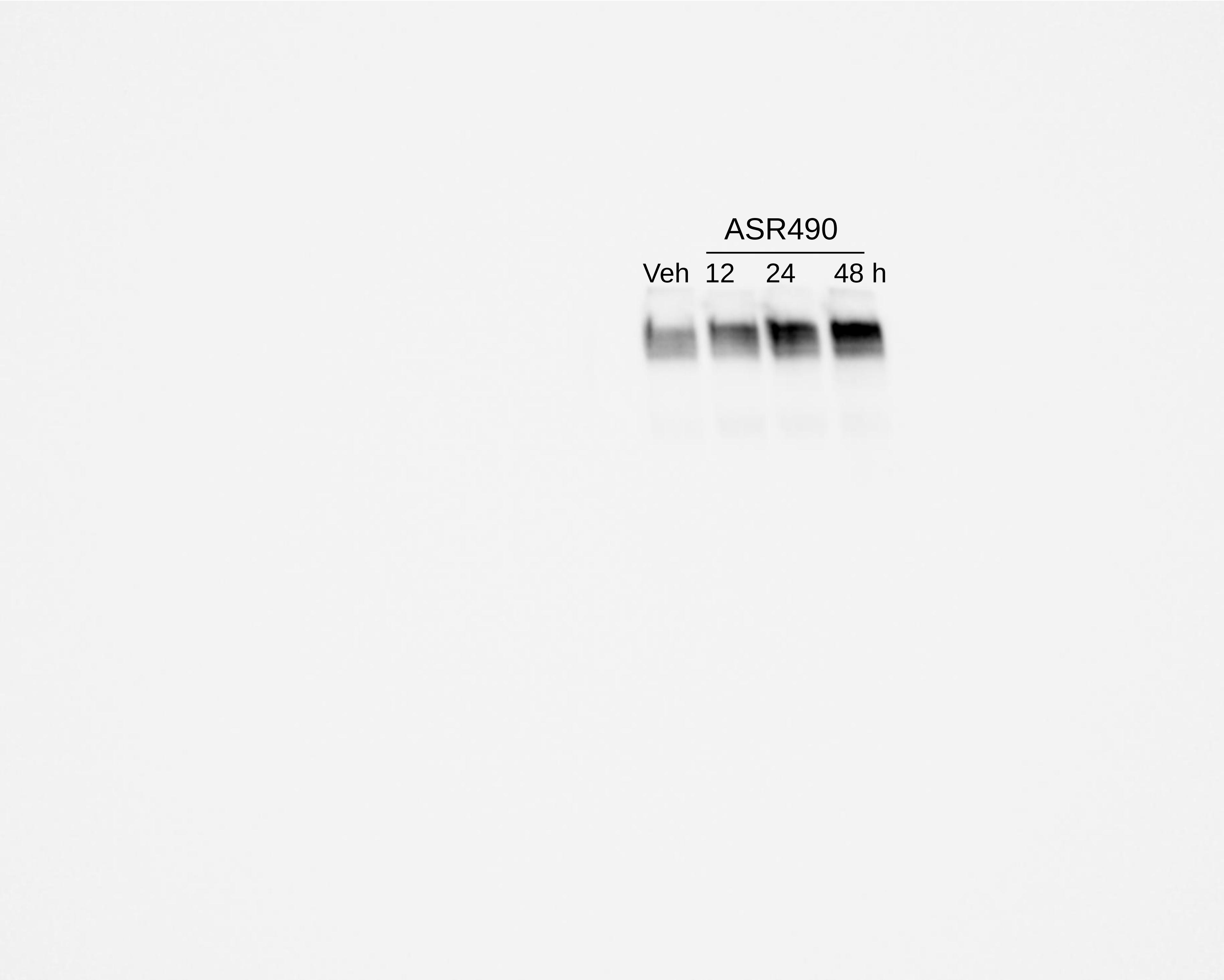

LC3B

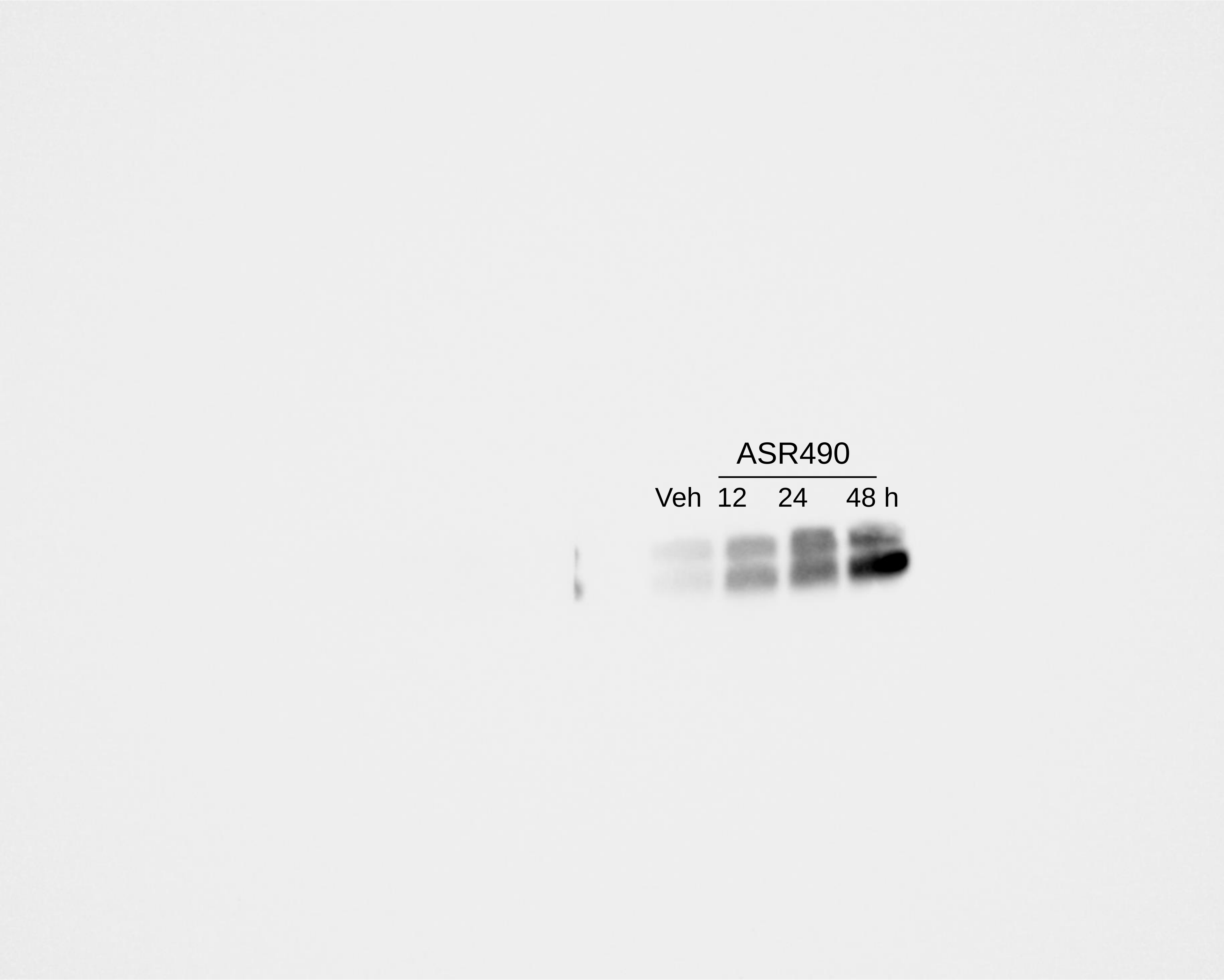

Actin

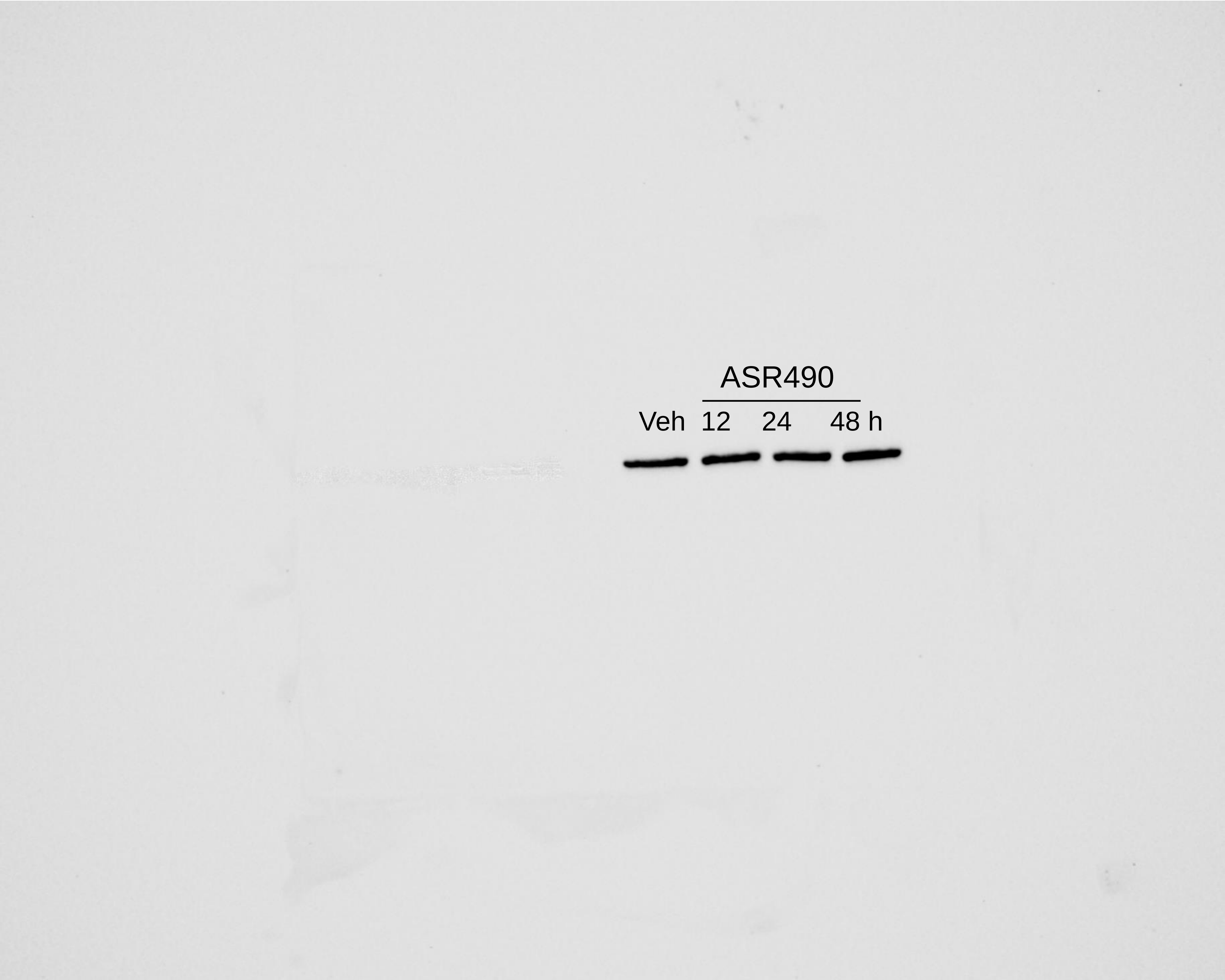

Figure 5F

Lamp1

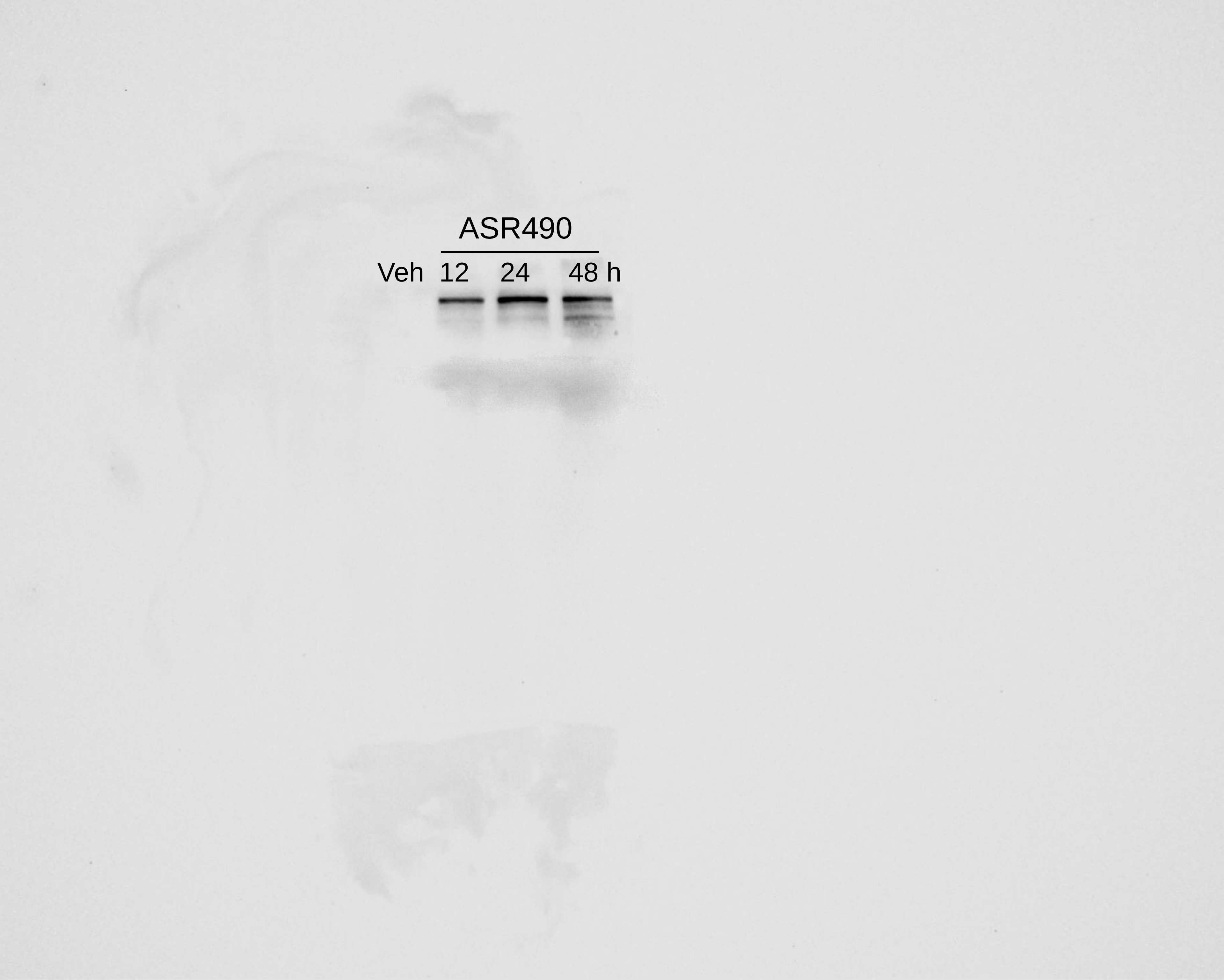

LC3B

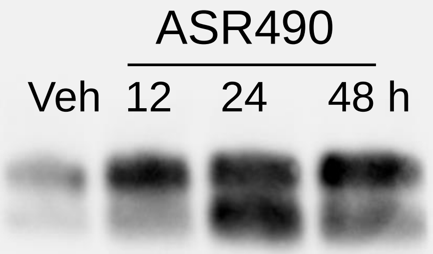

Actin

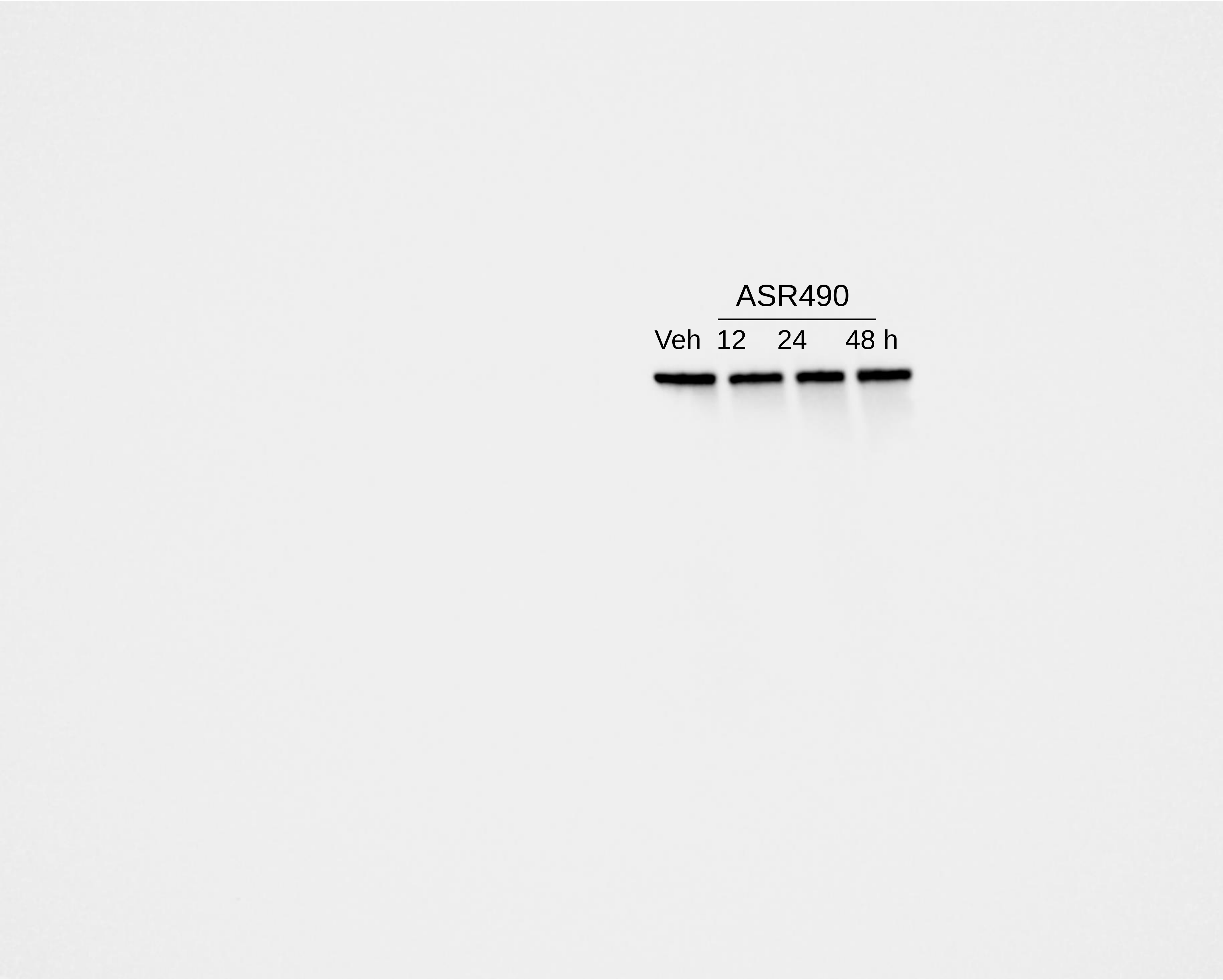

Figure 5G

Lamp1

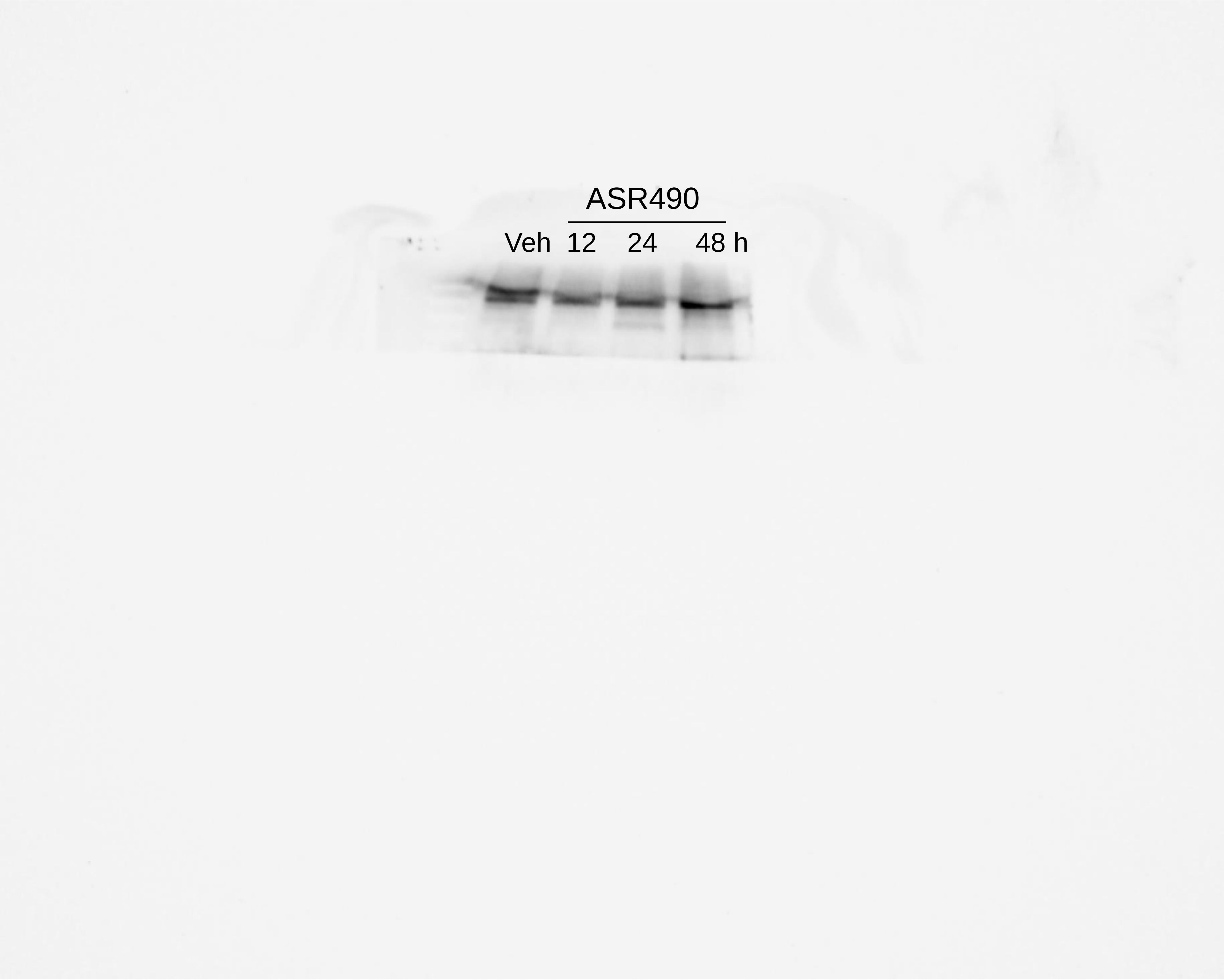

LC3B

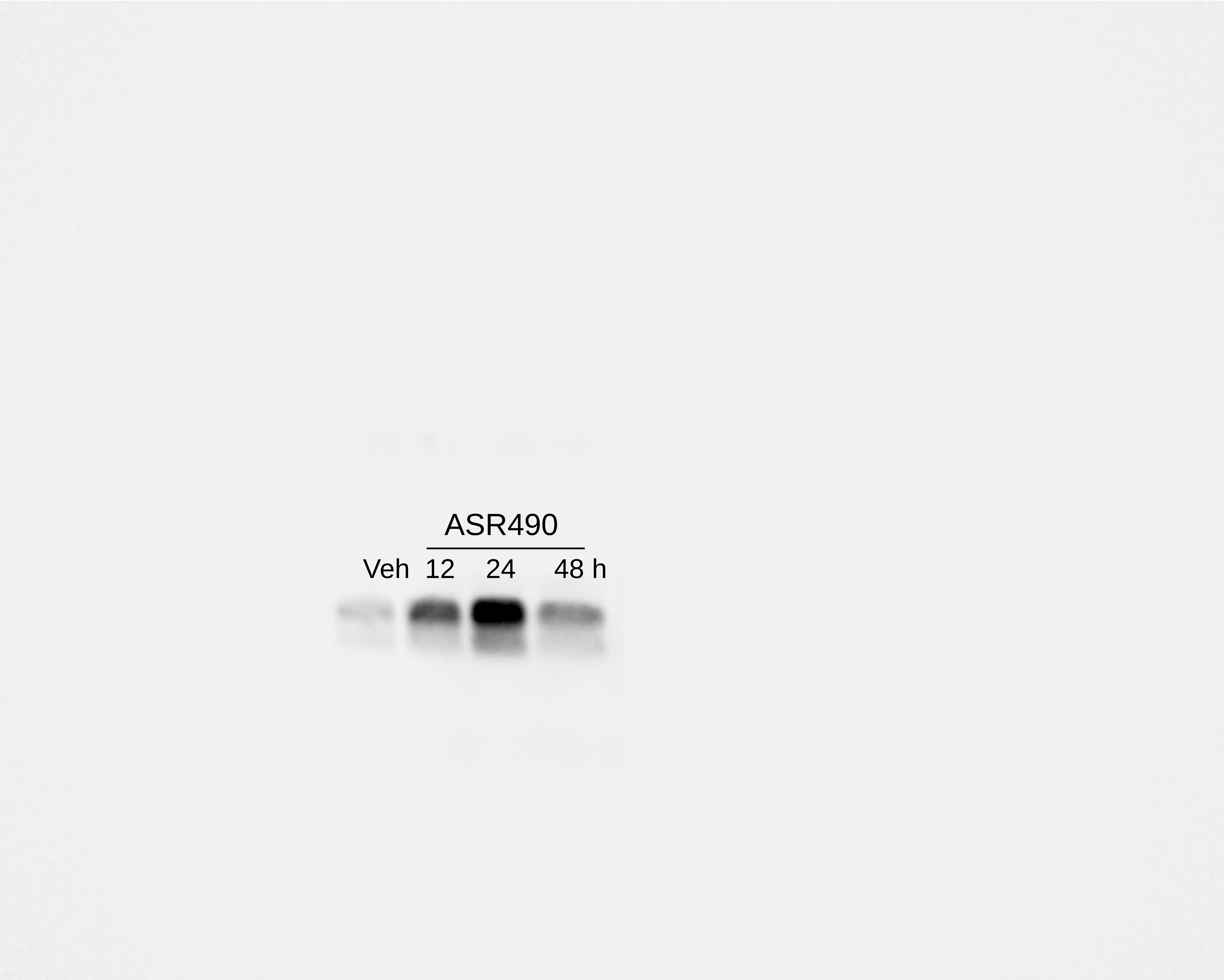

Actin

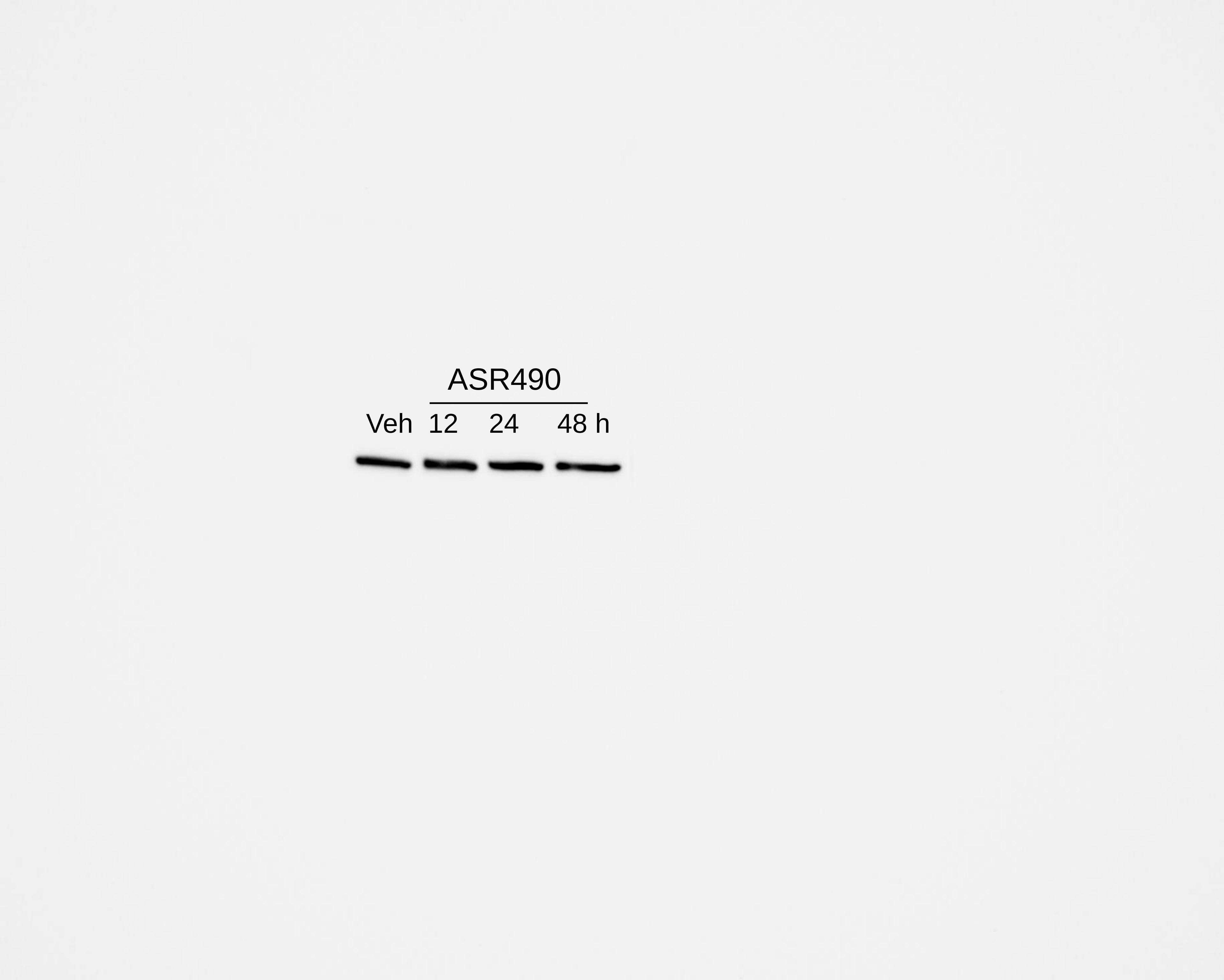

Figure 5H

Lamp1

ASR490

Veh 0.400 0.800 1.6 2.0 µM

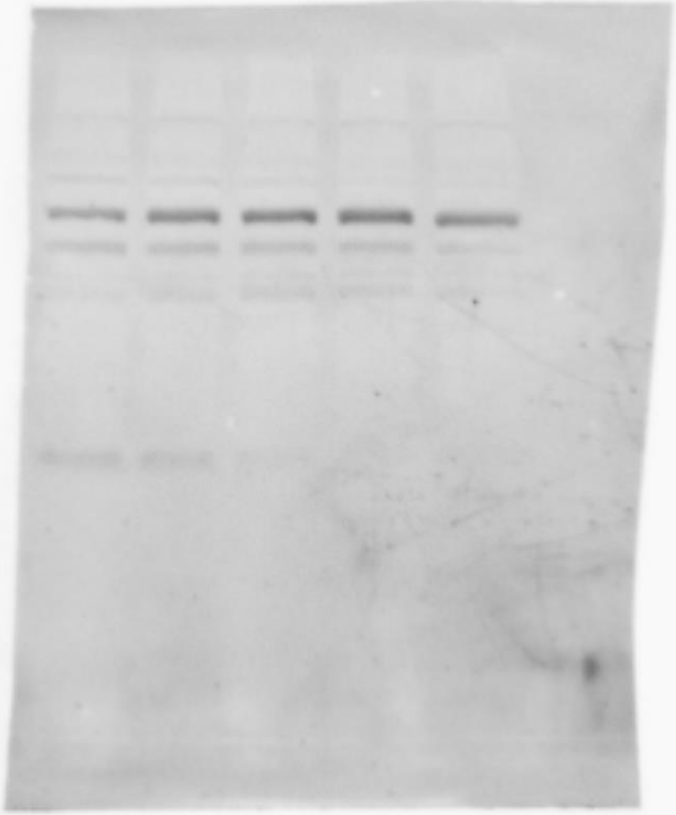

LC3B

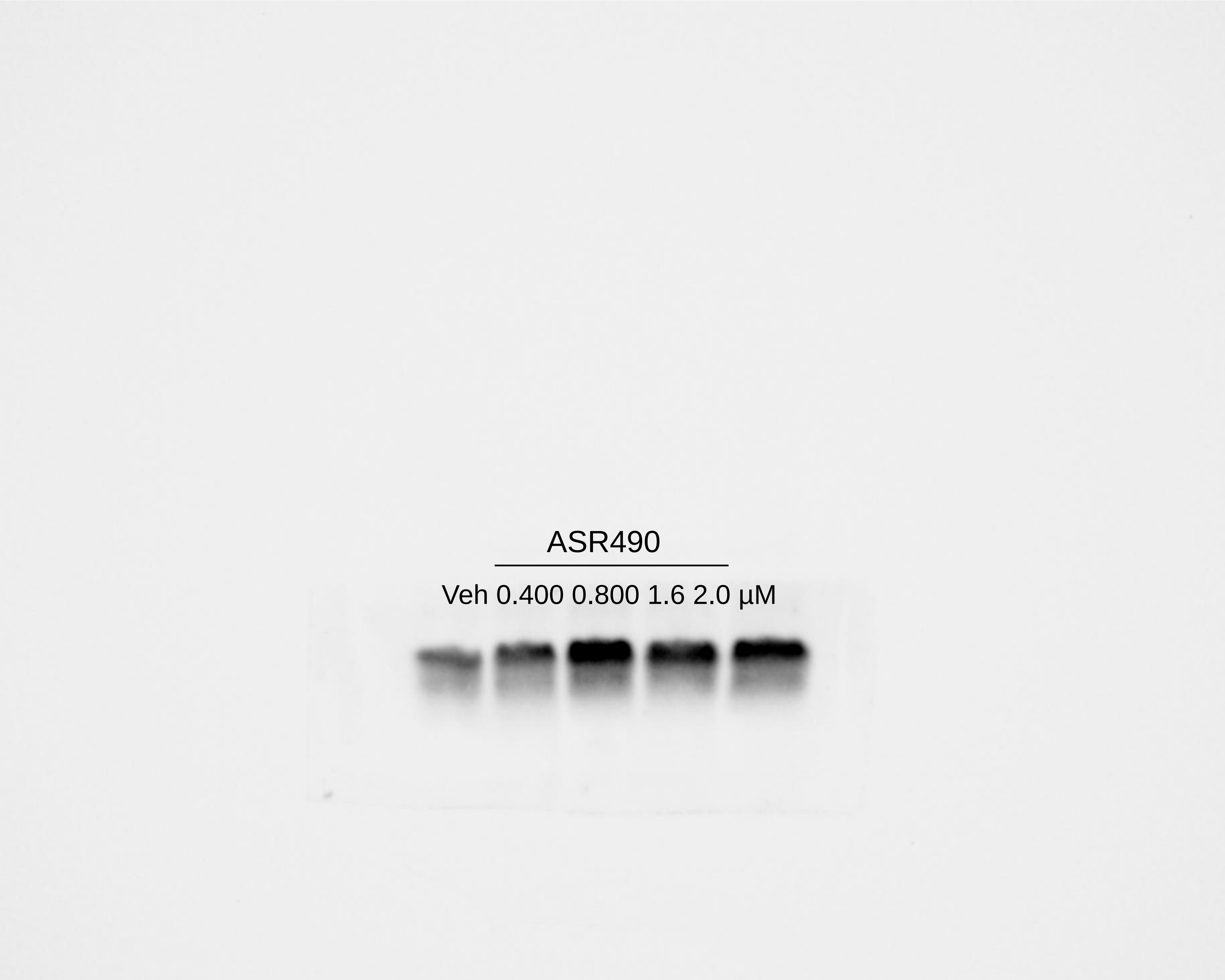

Actin

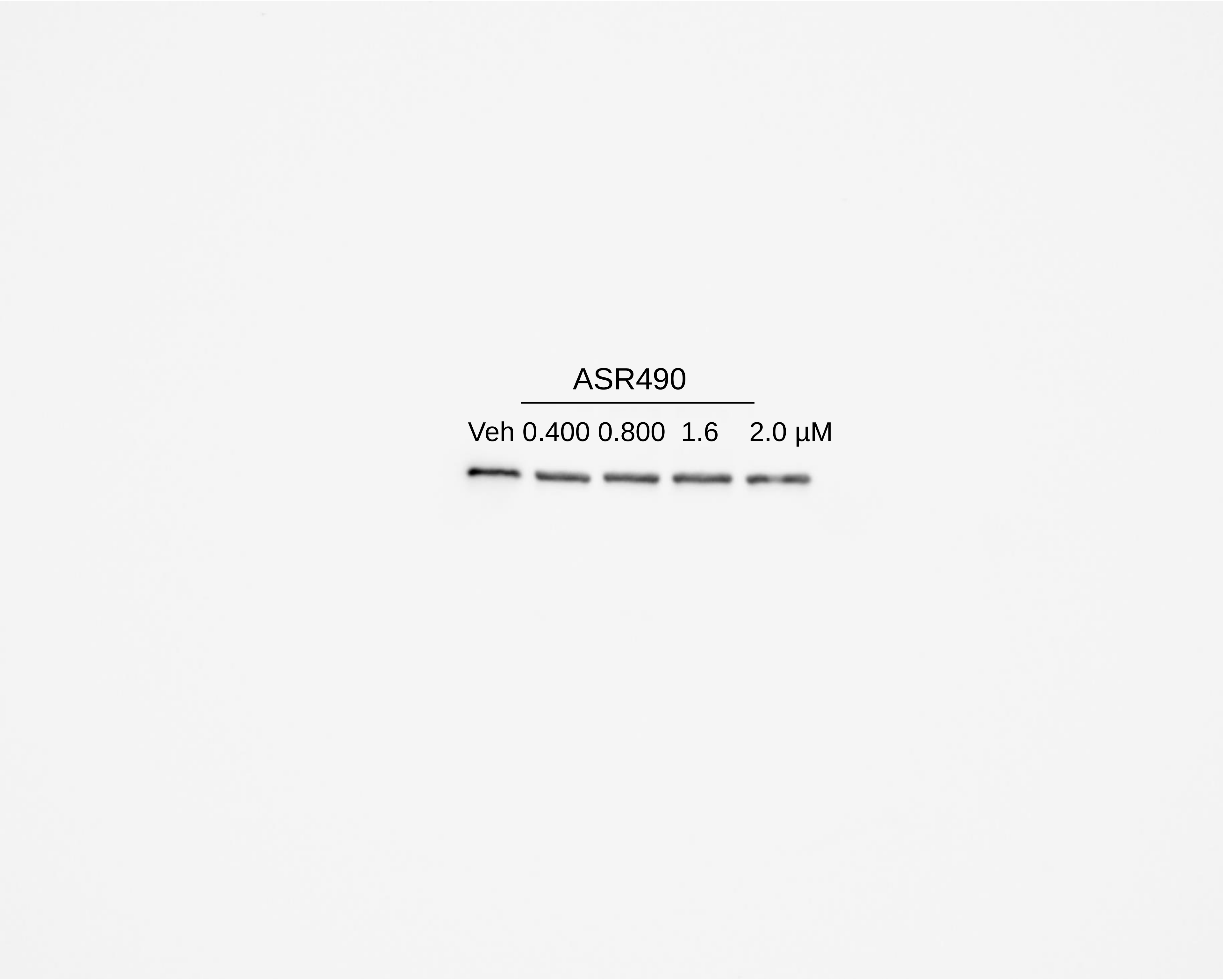

Figure 5I

Lamp1

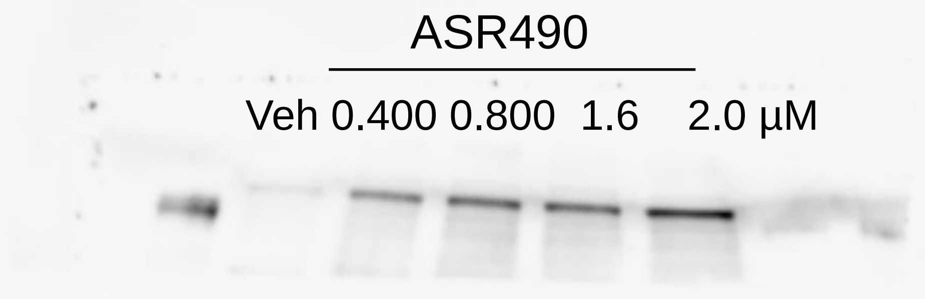

LC3B

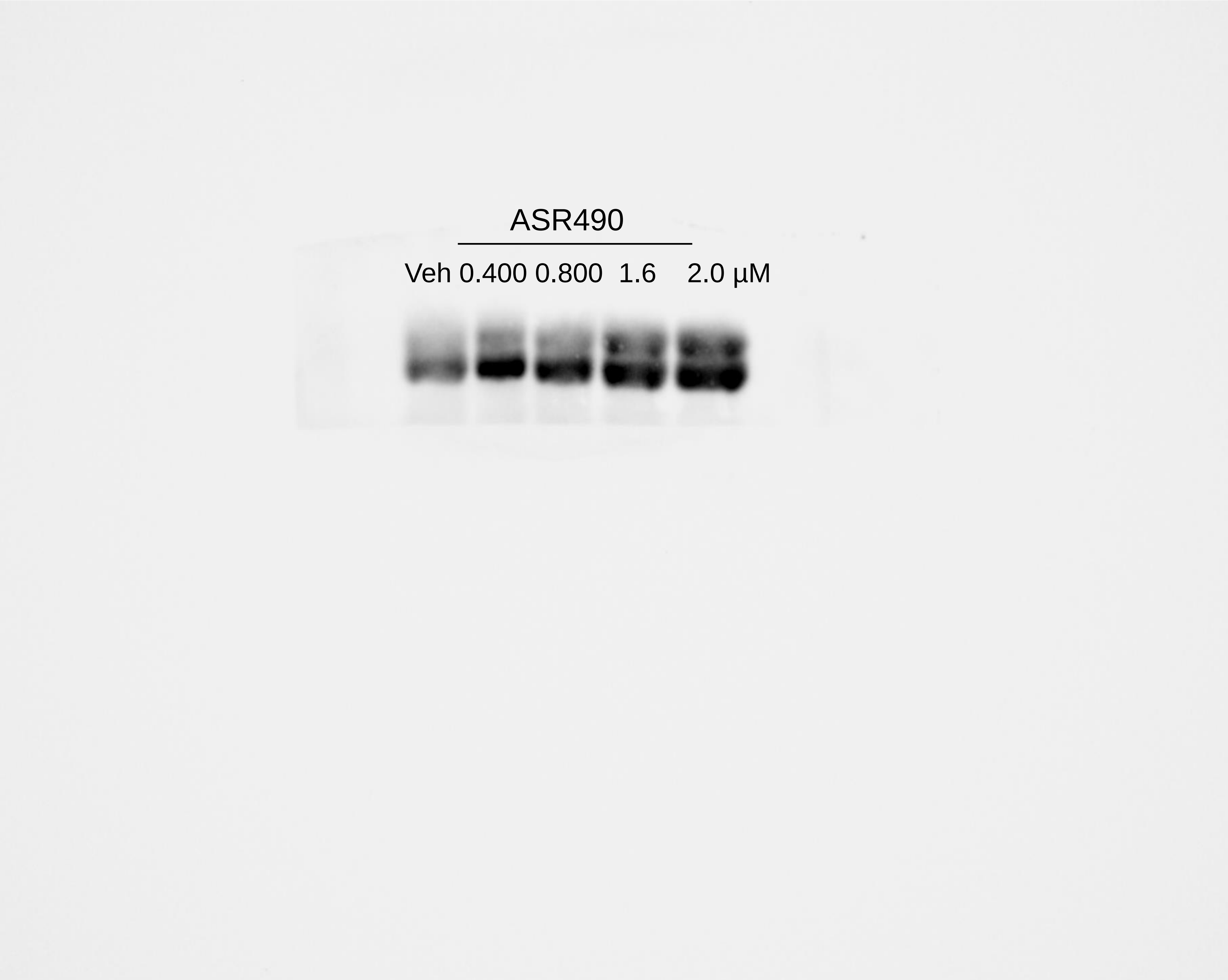

Actin

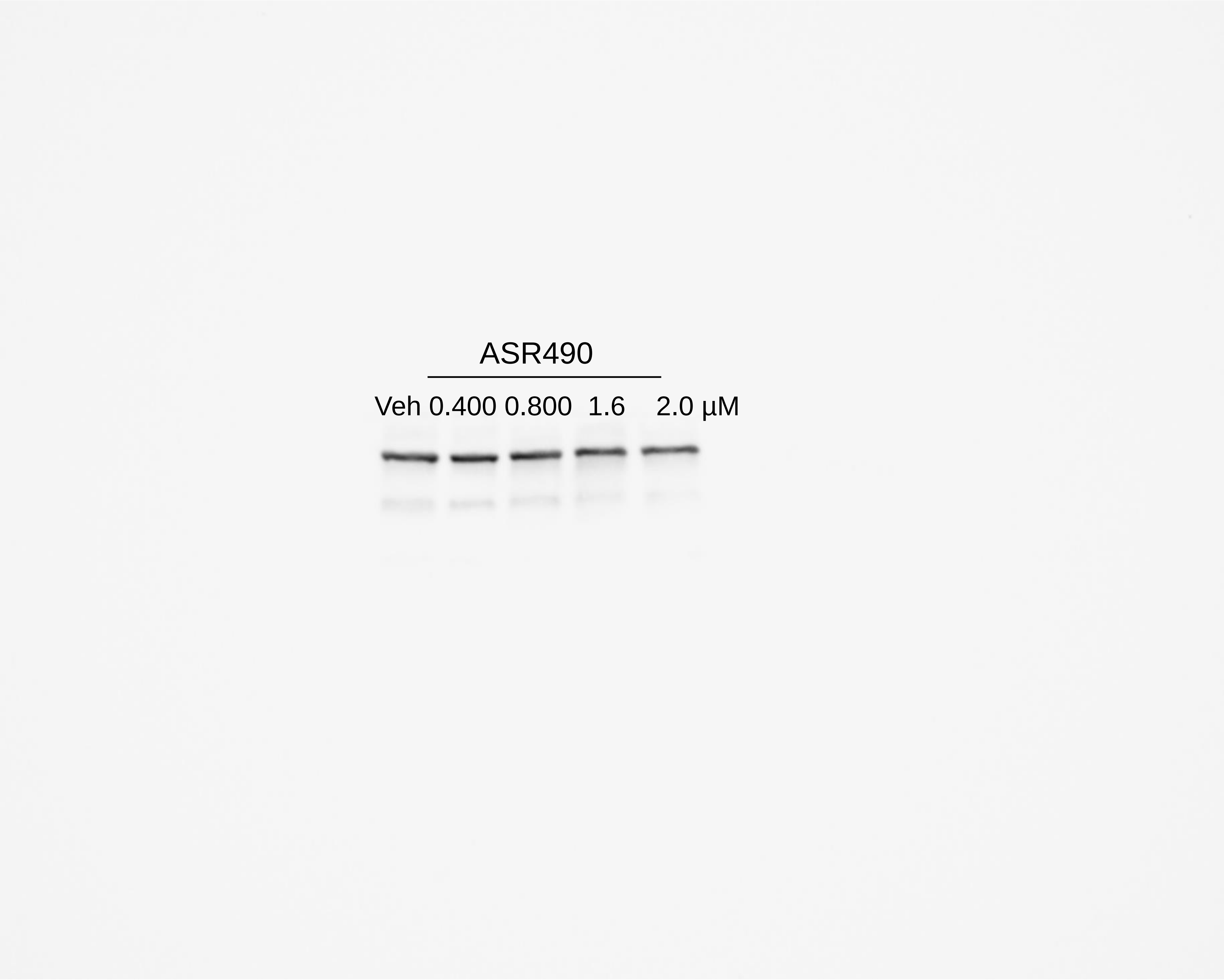

Figure 5J

Lamp1

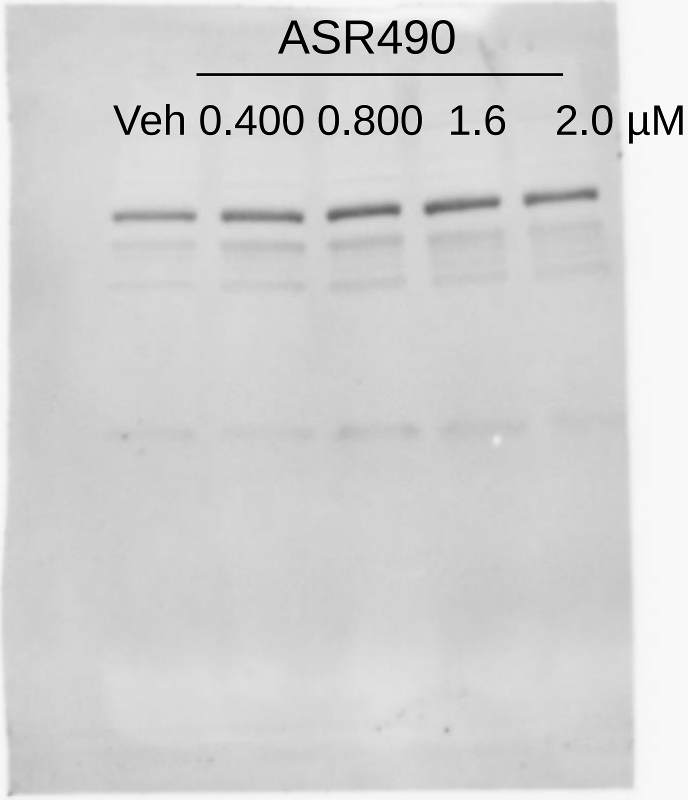

LC3B

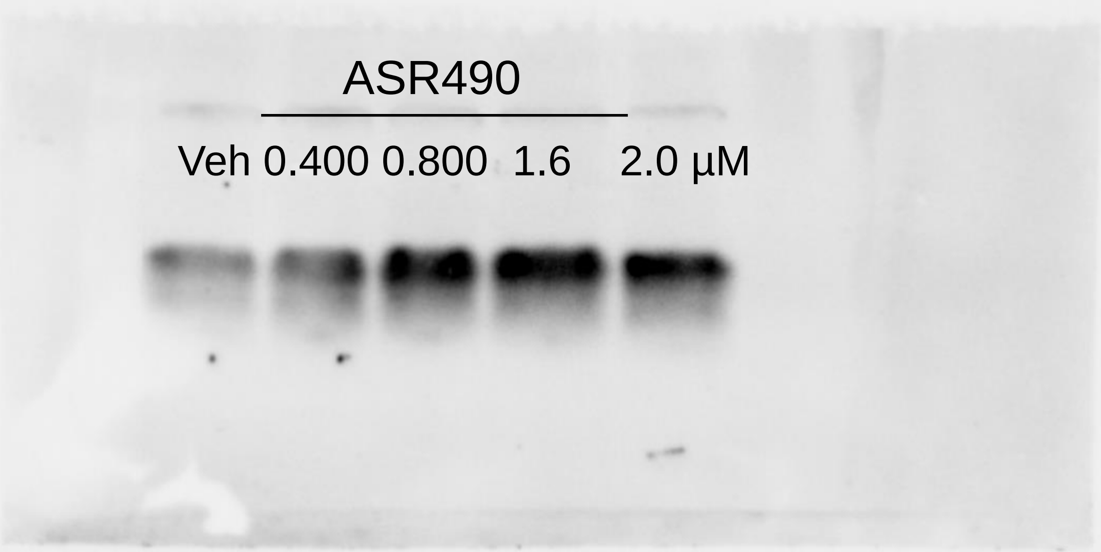

Actin

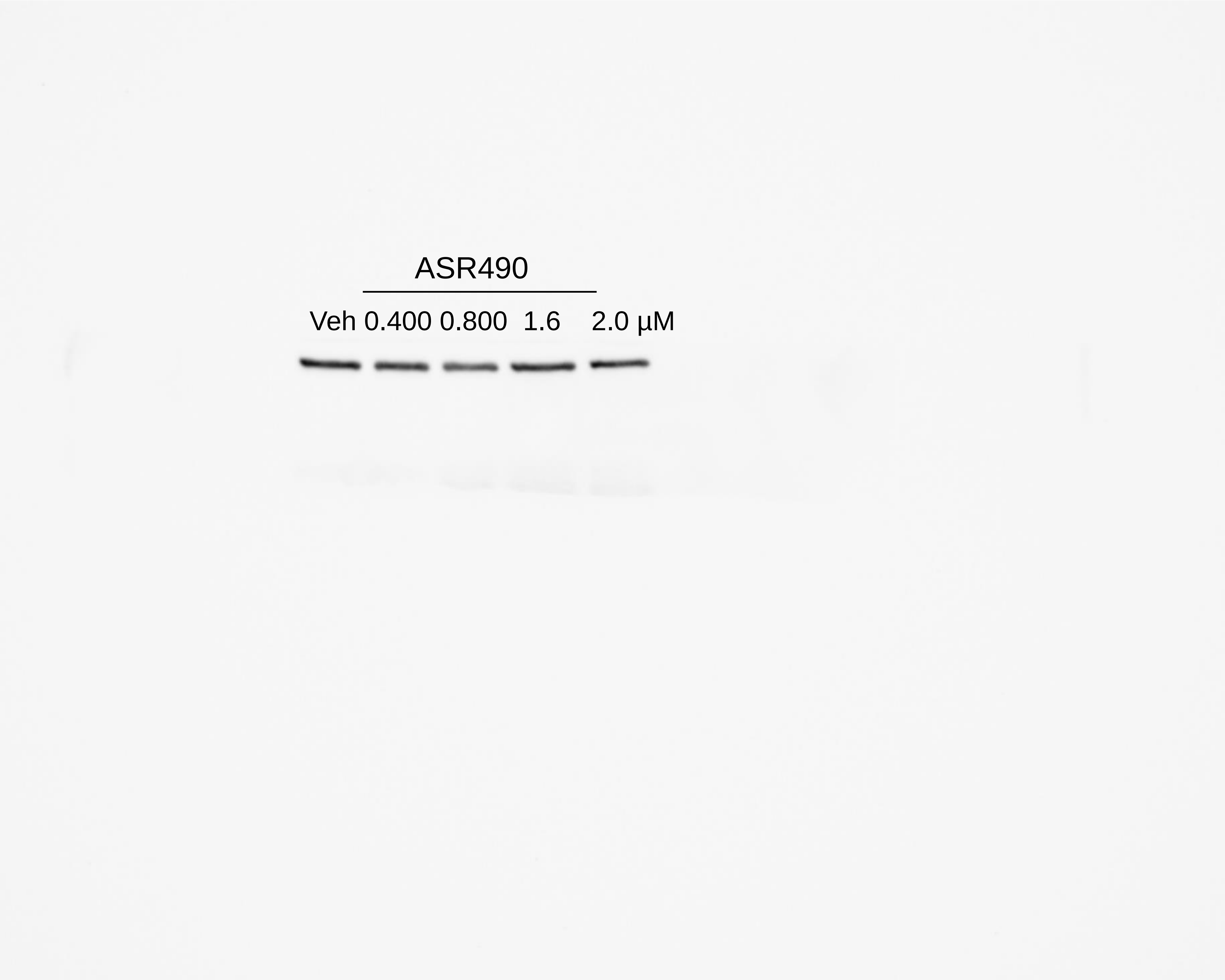

Figure 6B

NICD Notch1

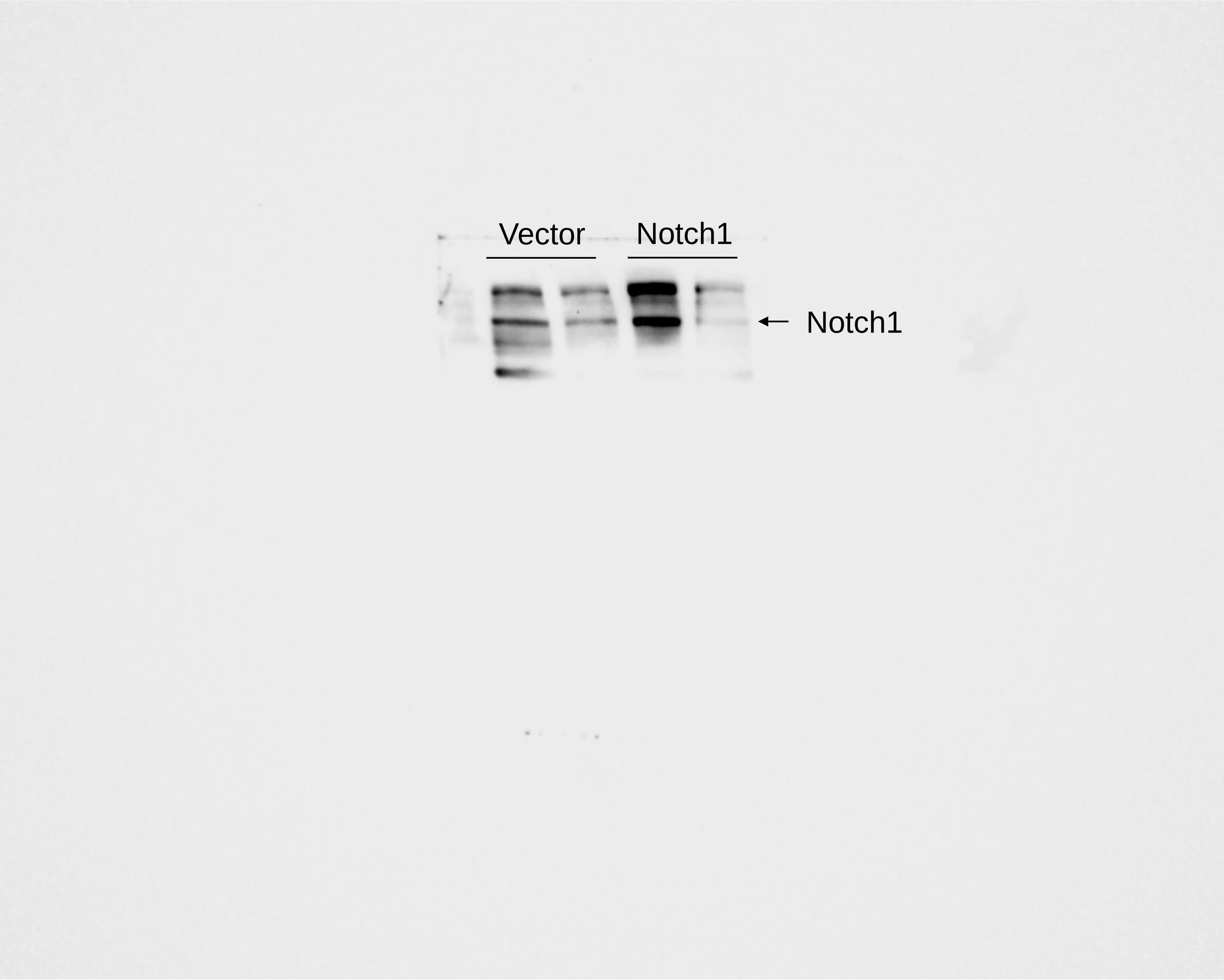

Lamp1

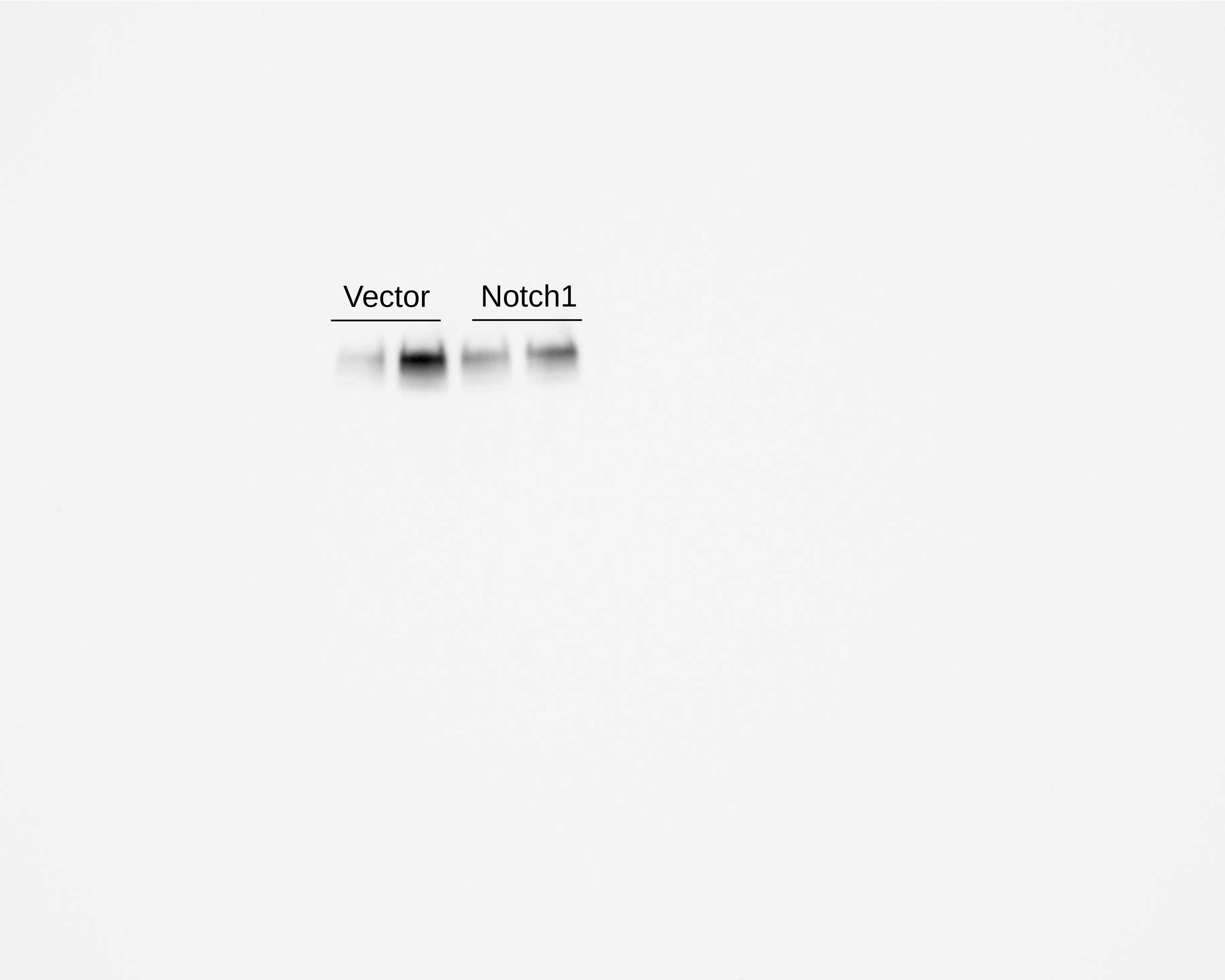

LC3B

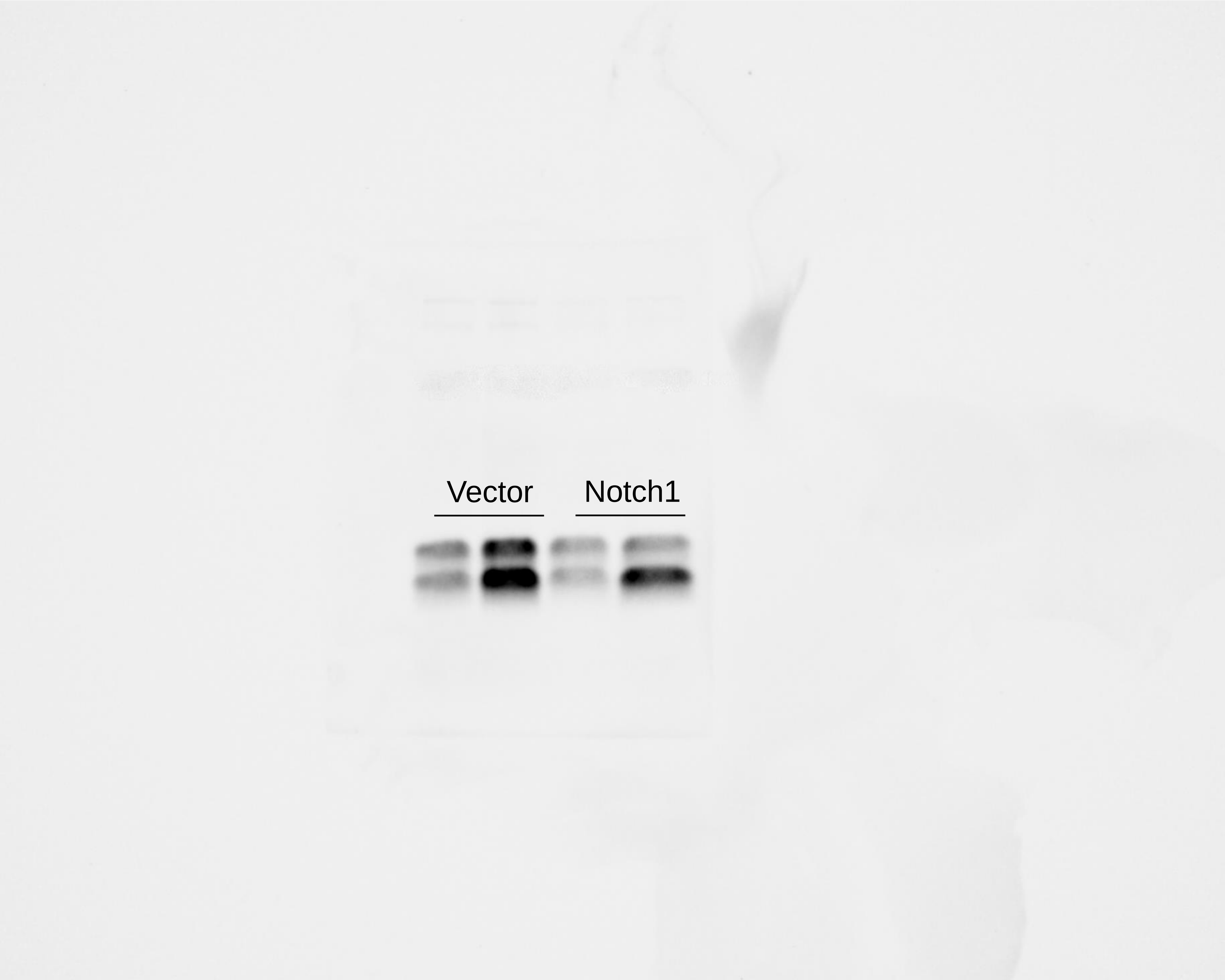

Actin

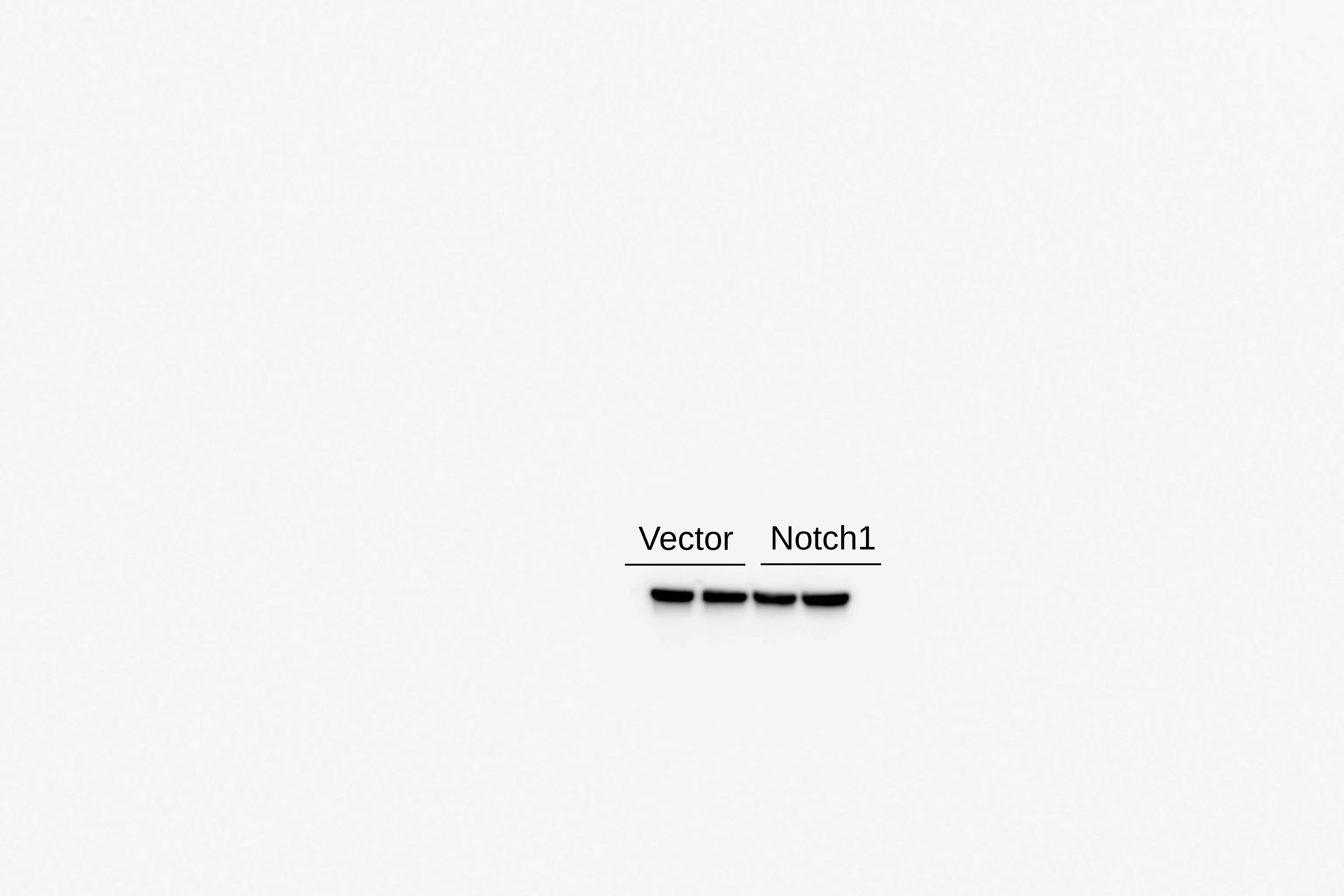

Figure 6C

Lamp1

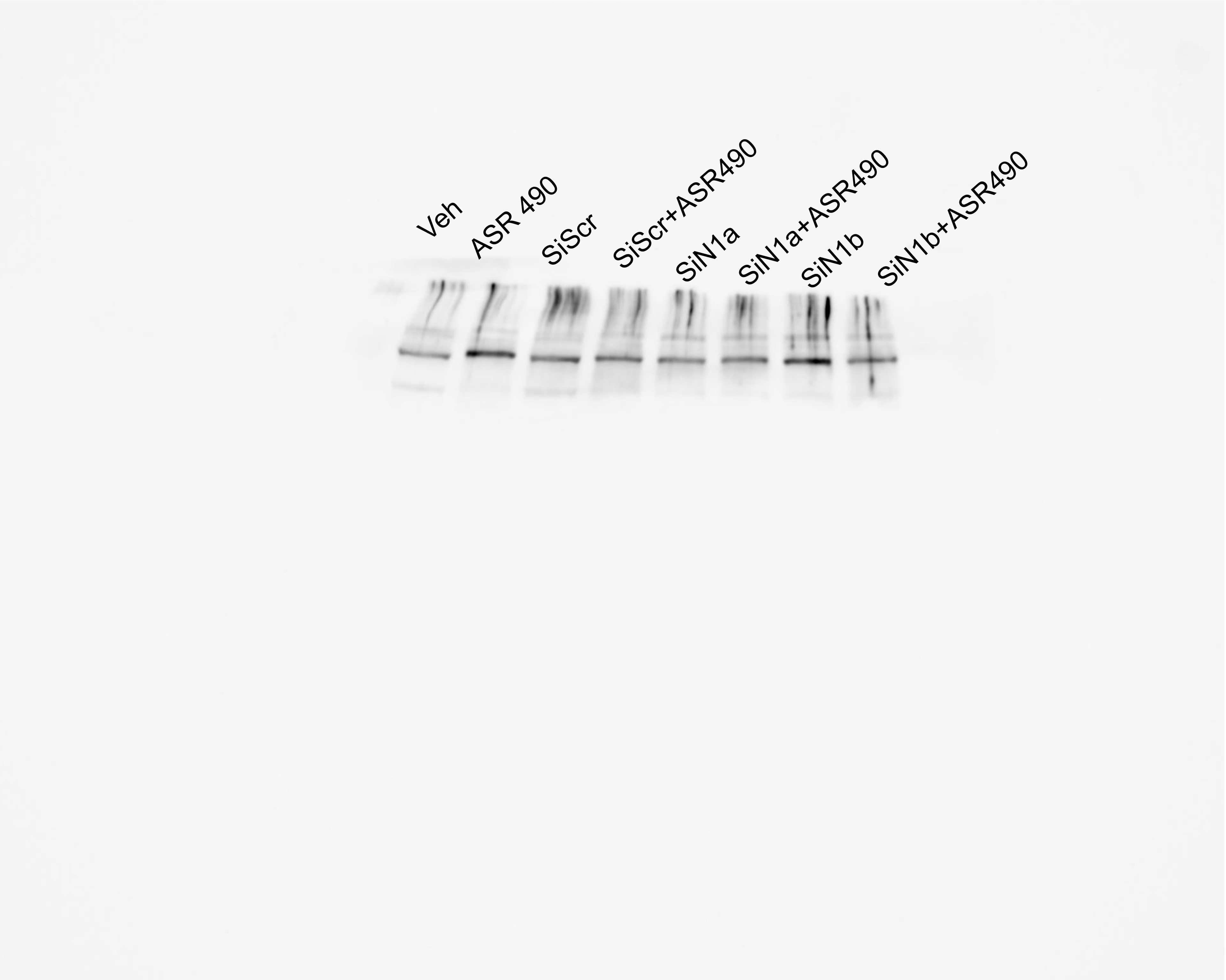

LC3B

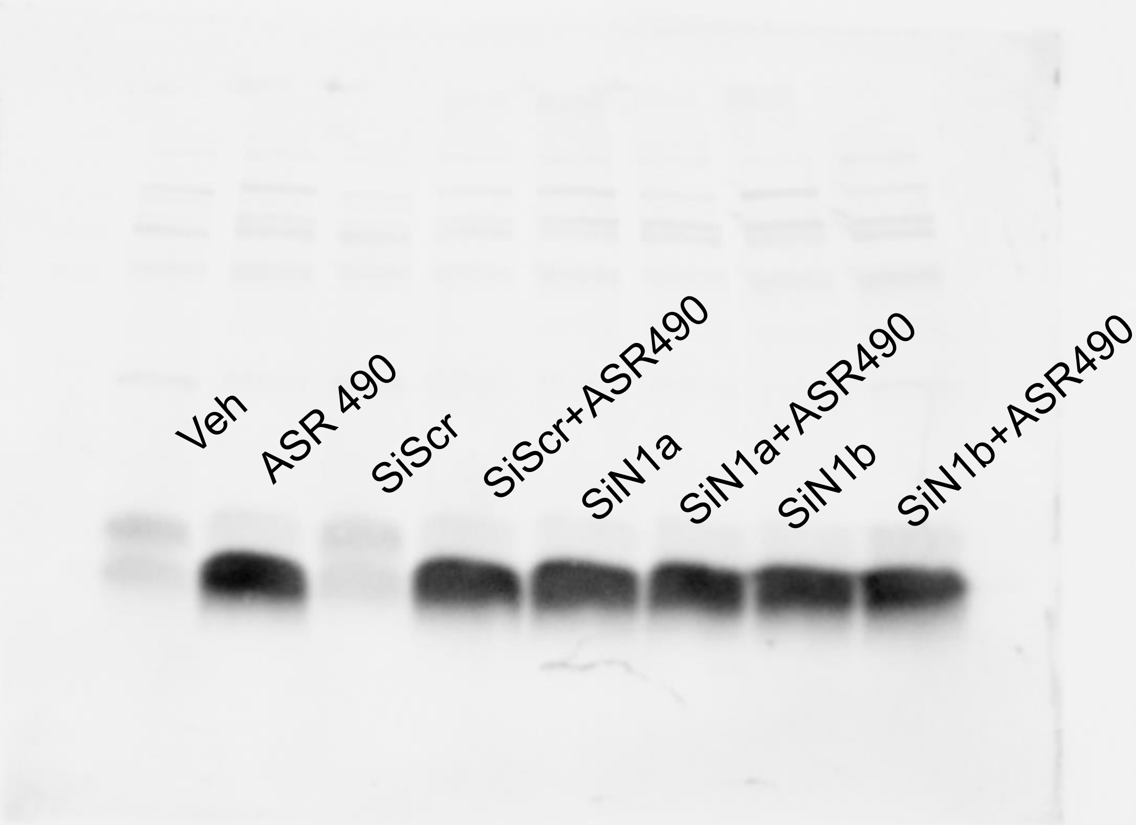
Veh
ASR 490
SiScr
SiScr+ASR490
SiN1a
SiN1a+ASR490
SiN1b
SiN1b+ASR490

Actin

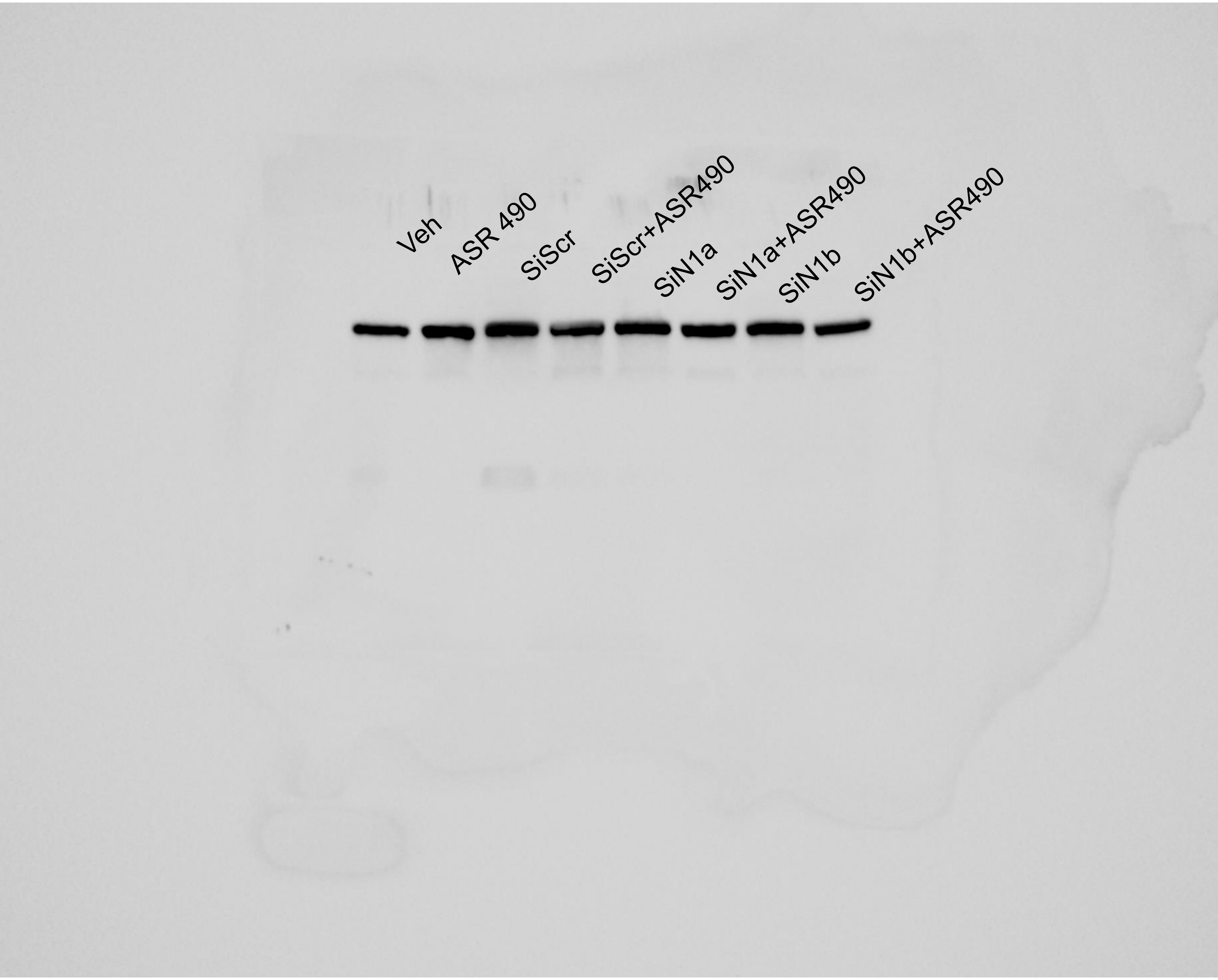
Veh
ASR 490
SiScr
SiScr+ASR490
SiN1a
SiN1a+ASR490
SiN1b
SiN1b+ASR490

Figure 6D

Lamp1

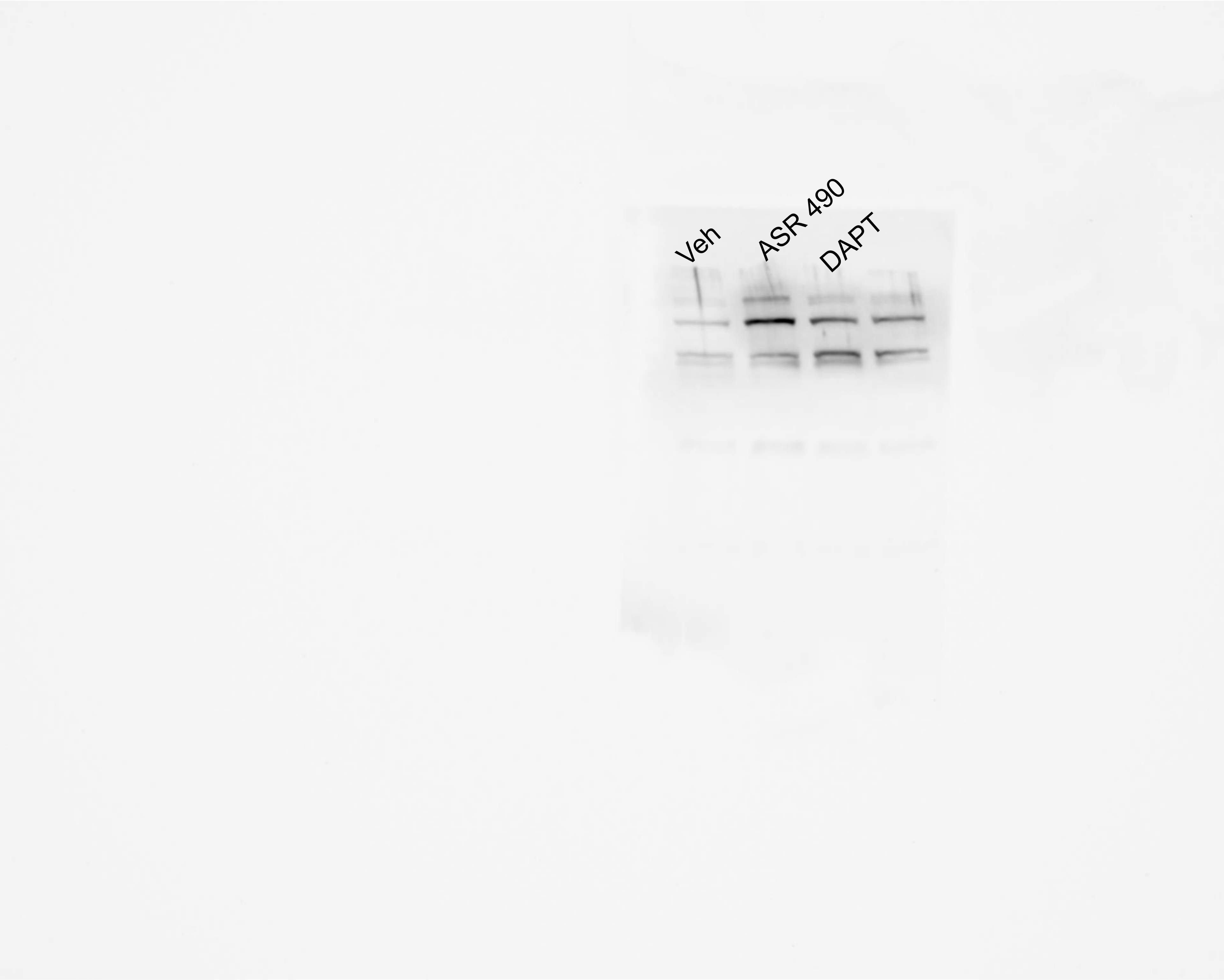

LC3B

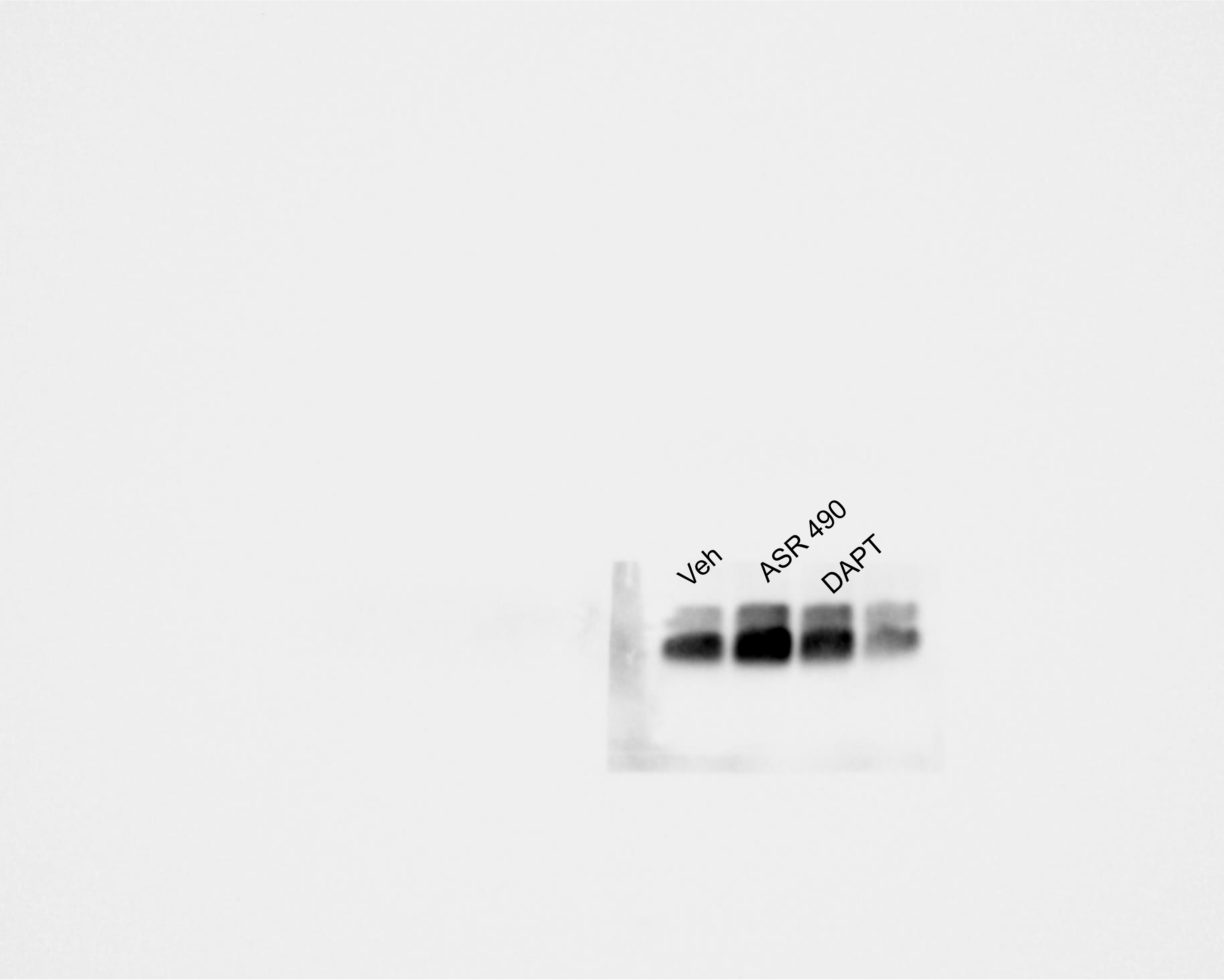

Actin

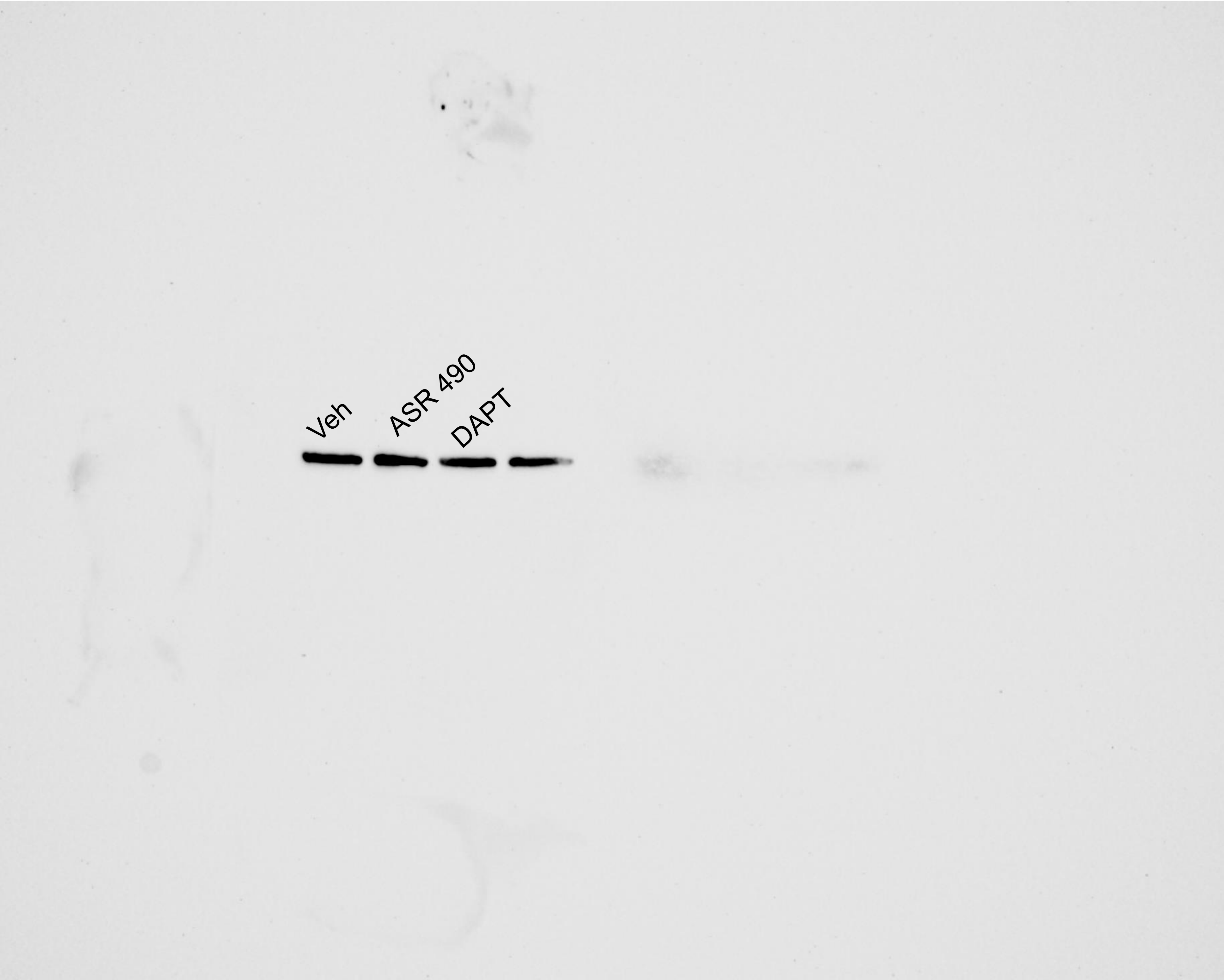

Figure 6E

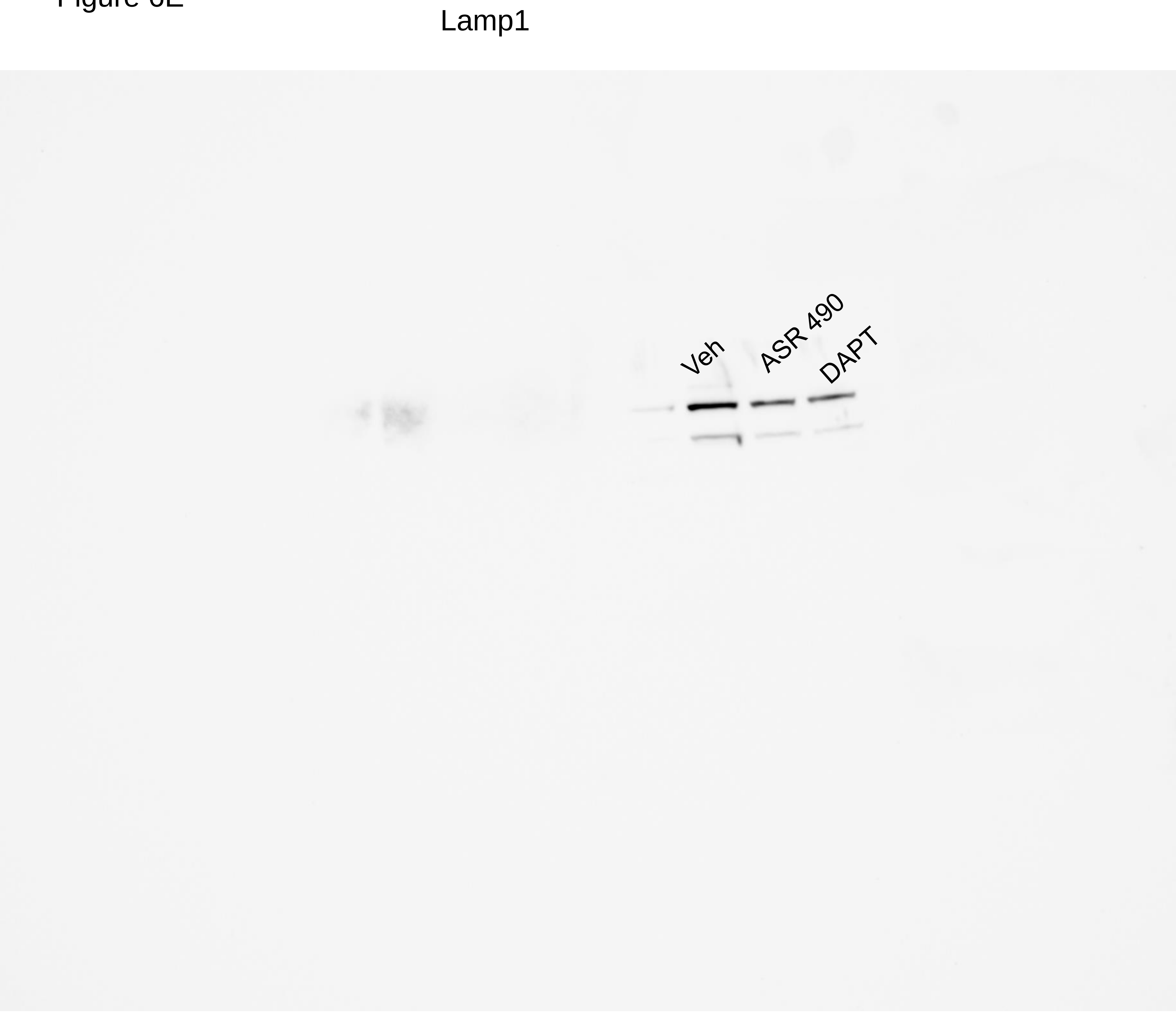

Figure 6E

LC3B

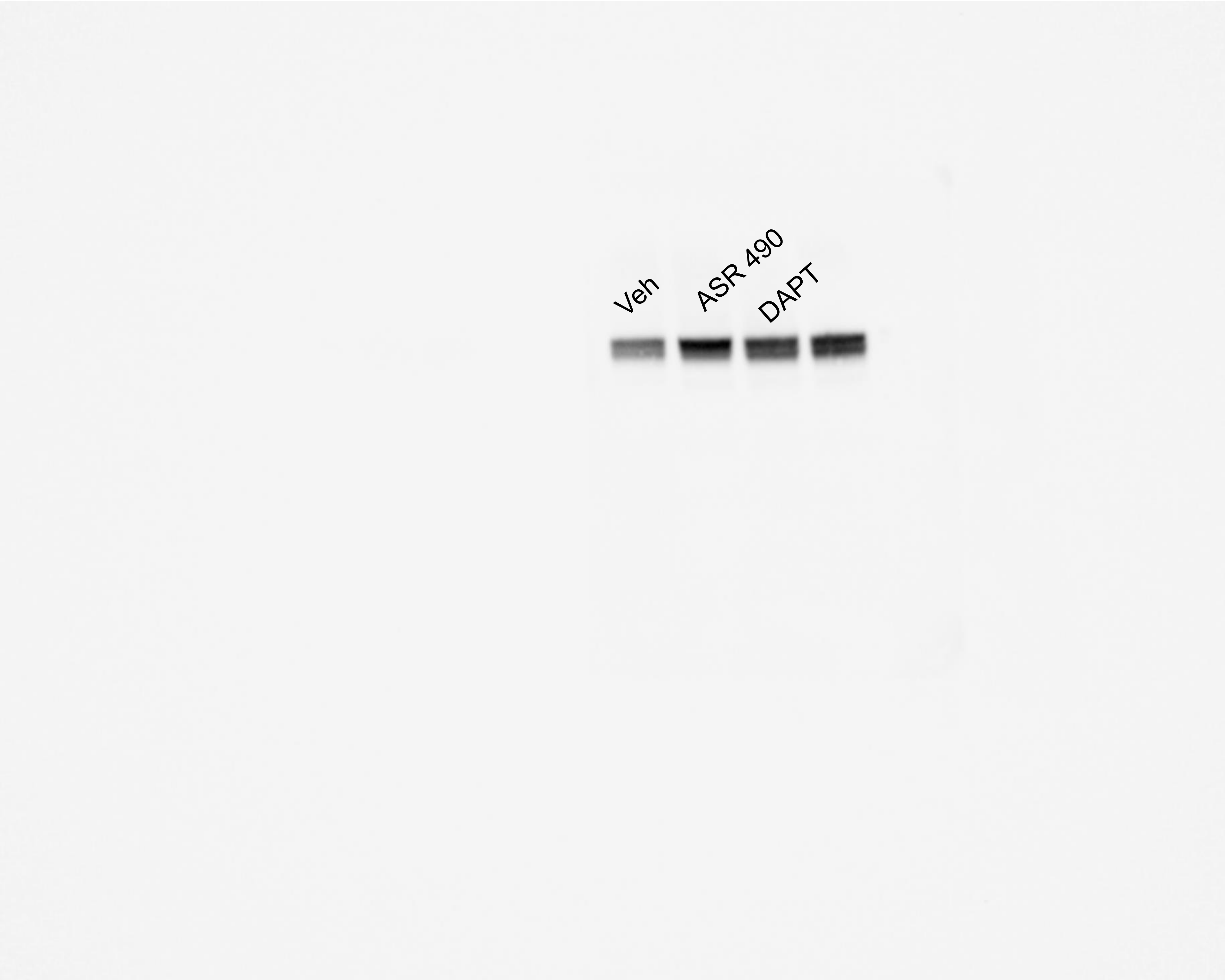

Actin

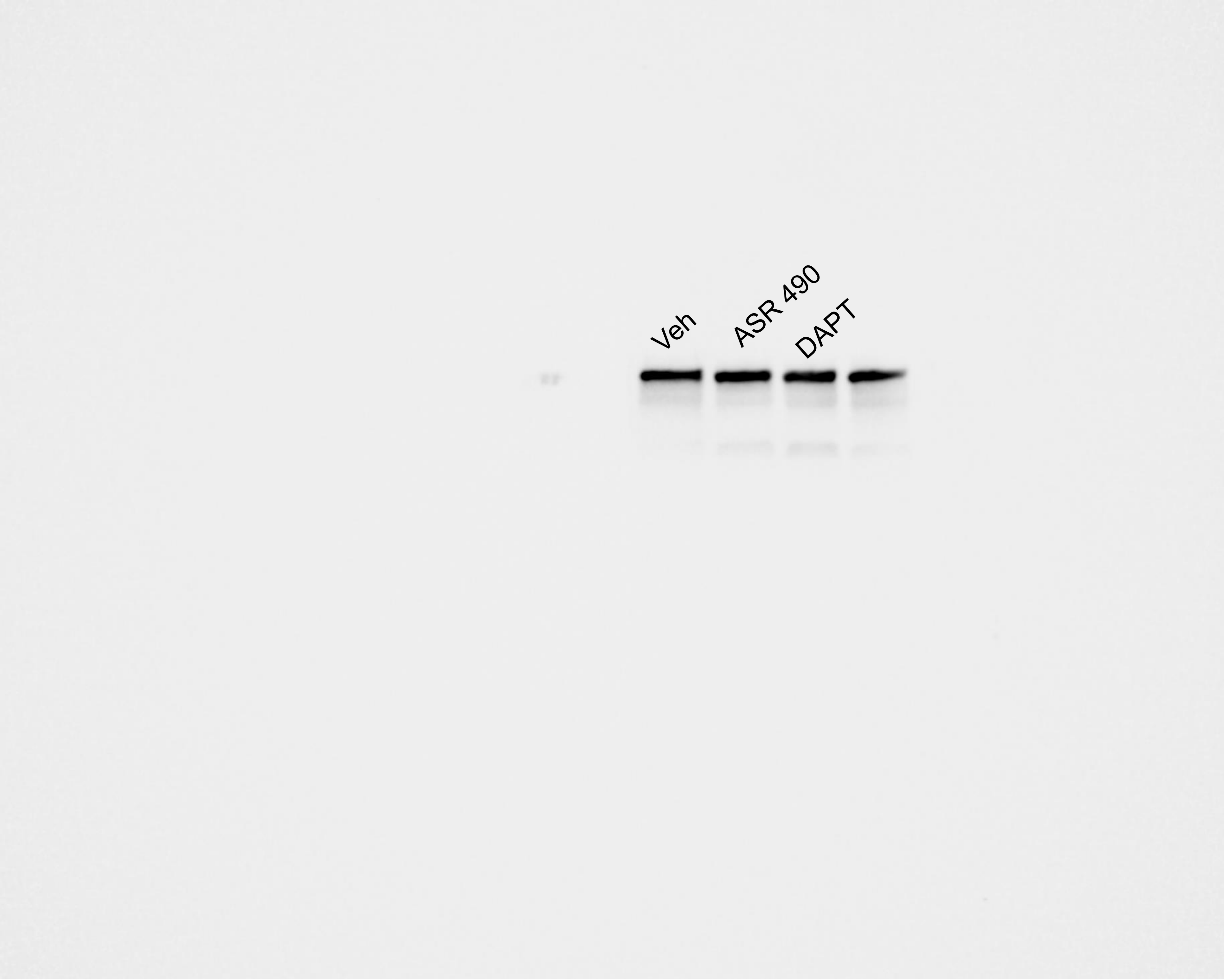

Figure 7C

E-cadherin

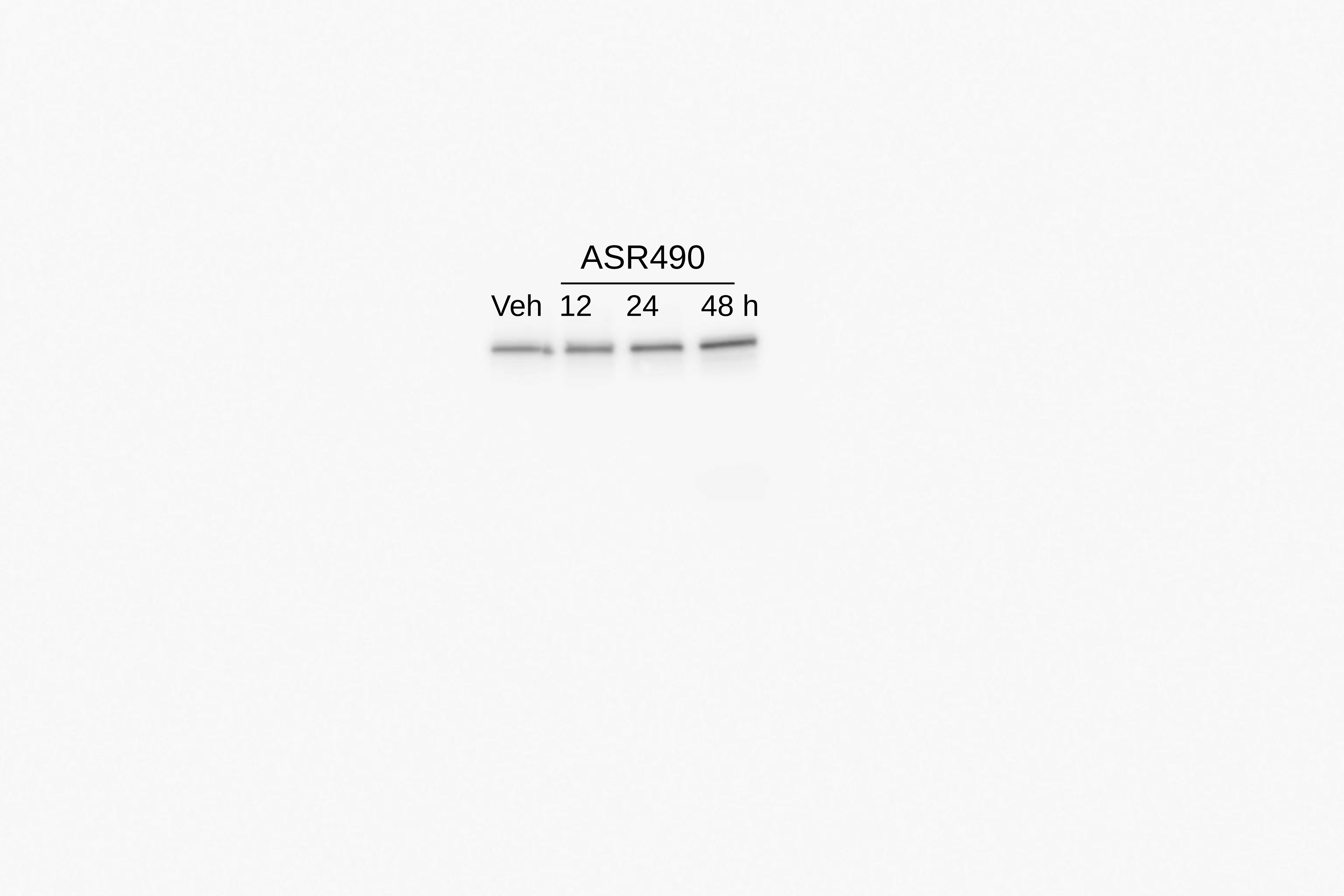

β-Catenin

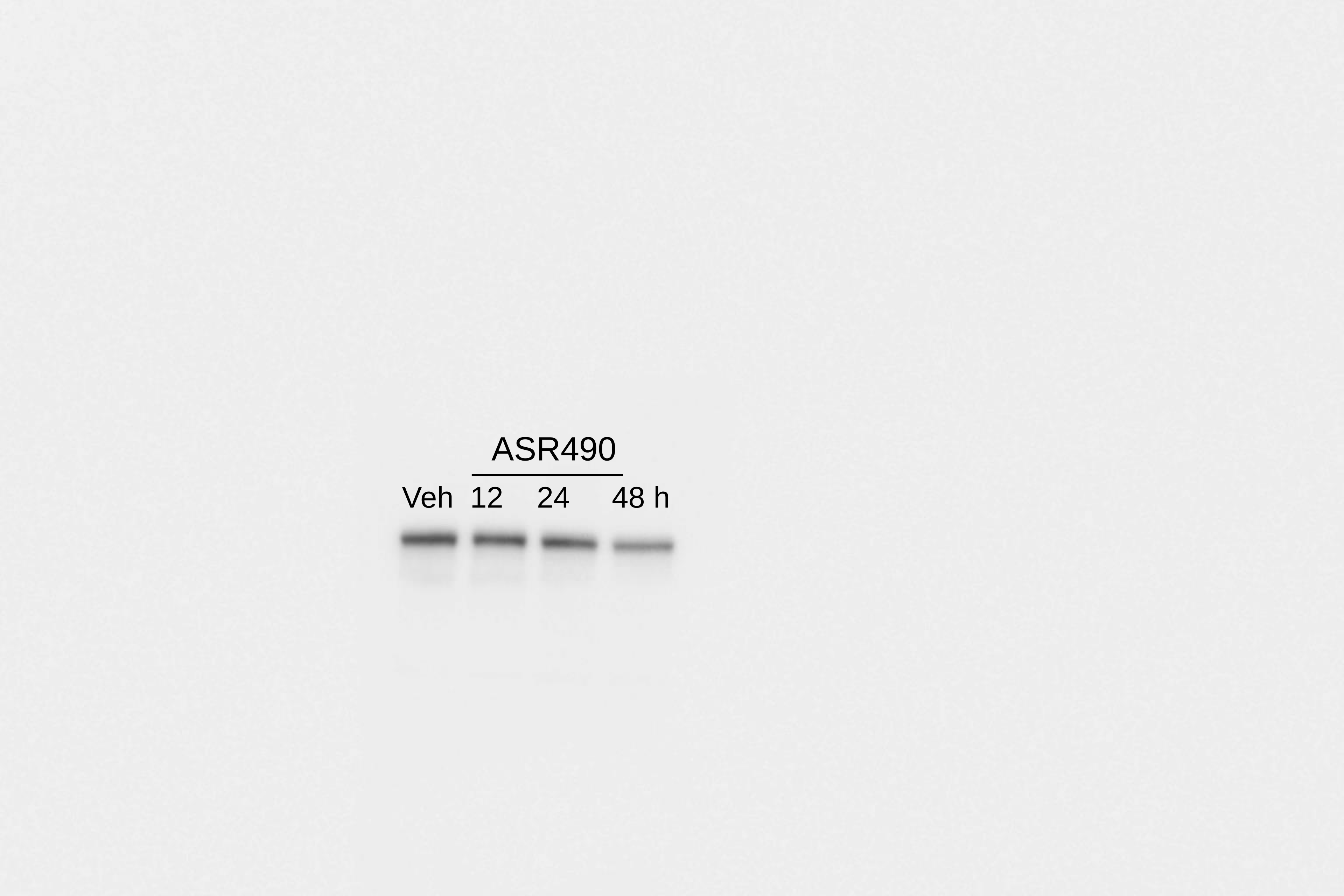

Slug

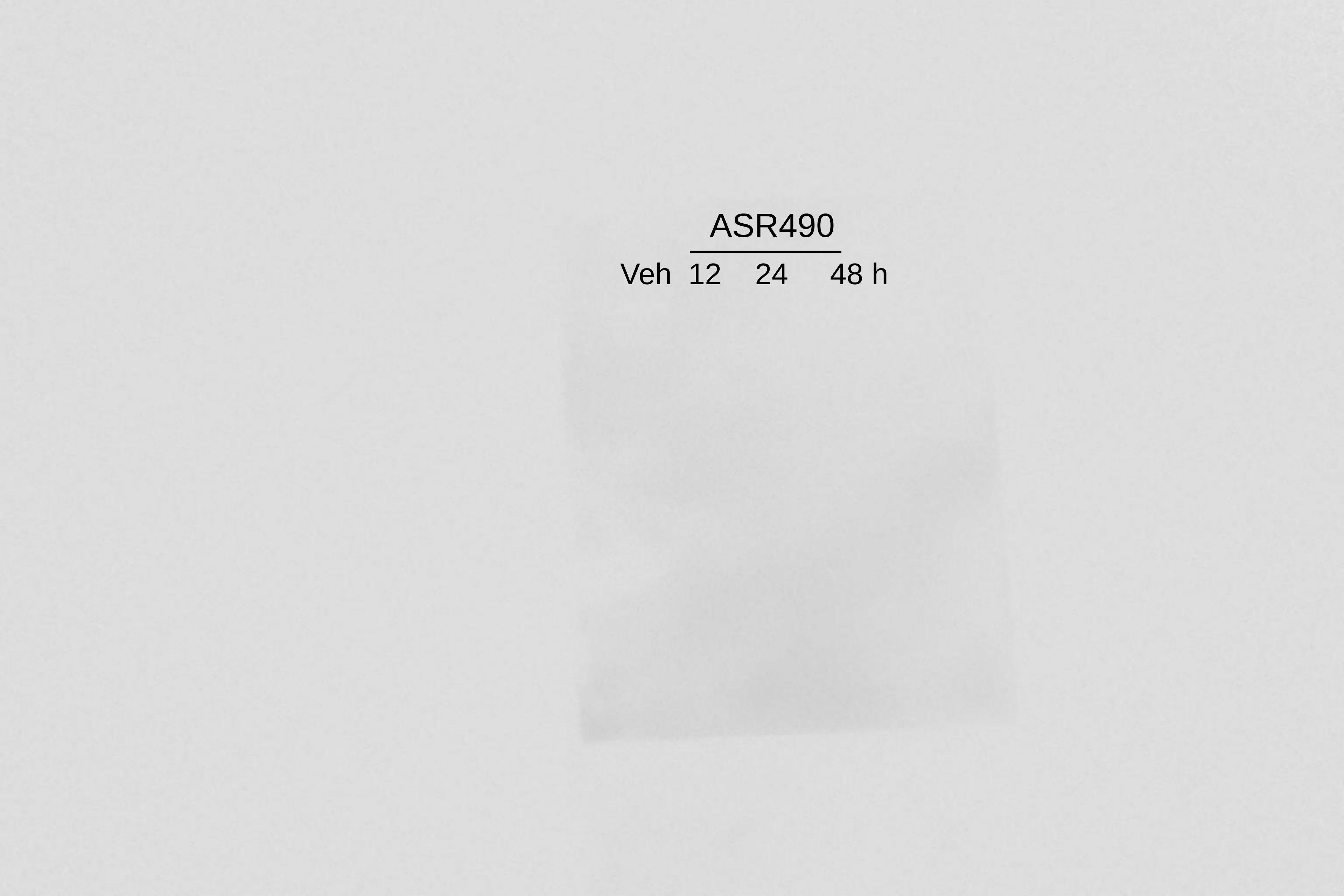

Vimentin

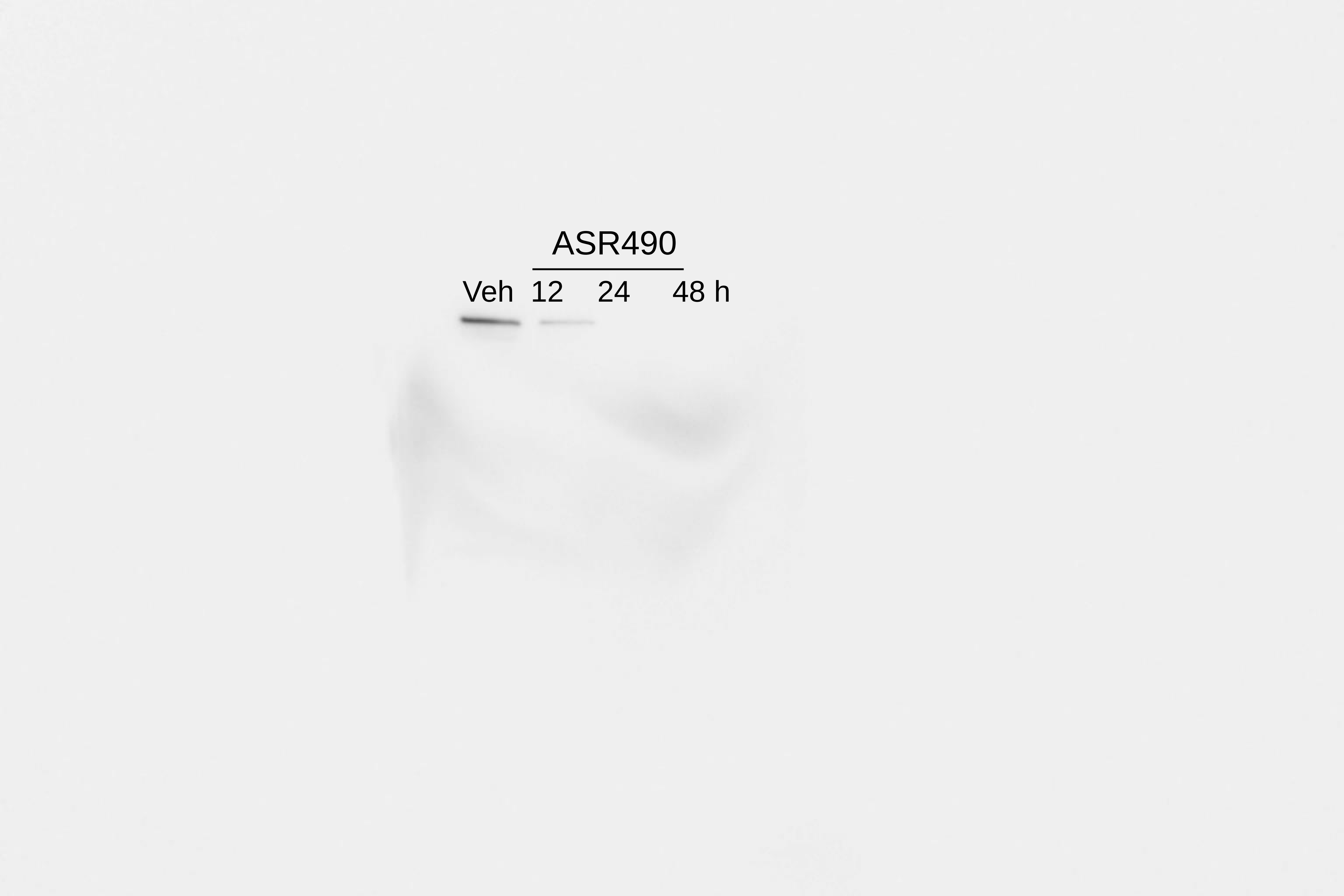

Actin

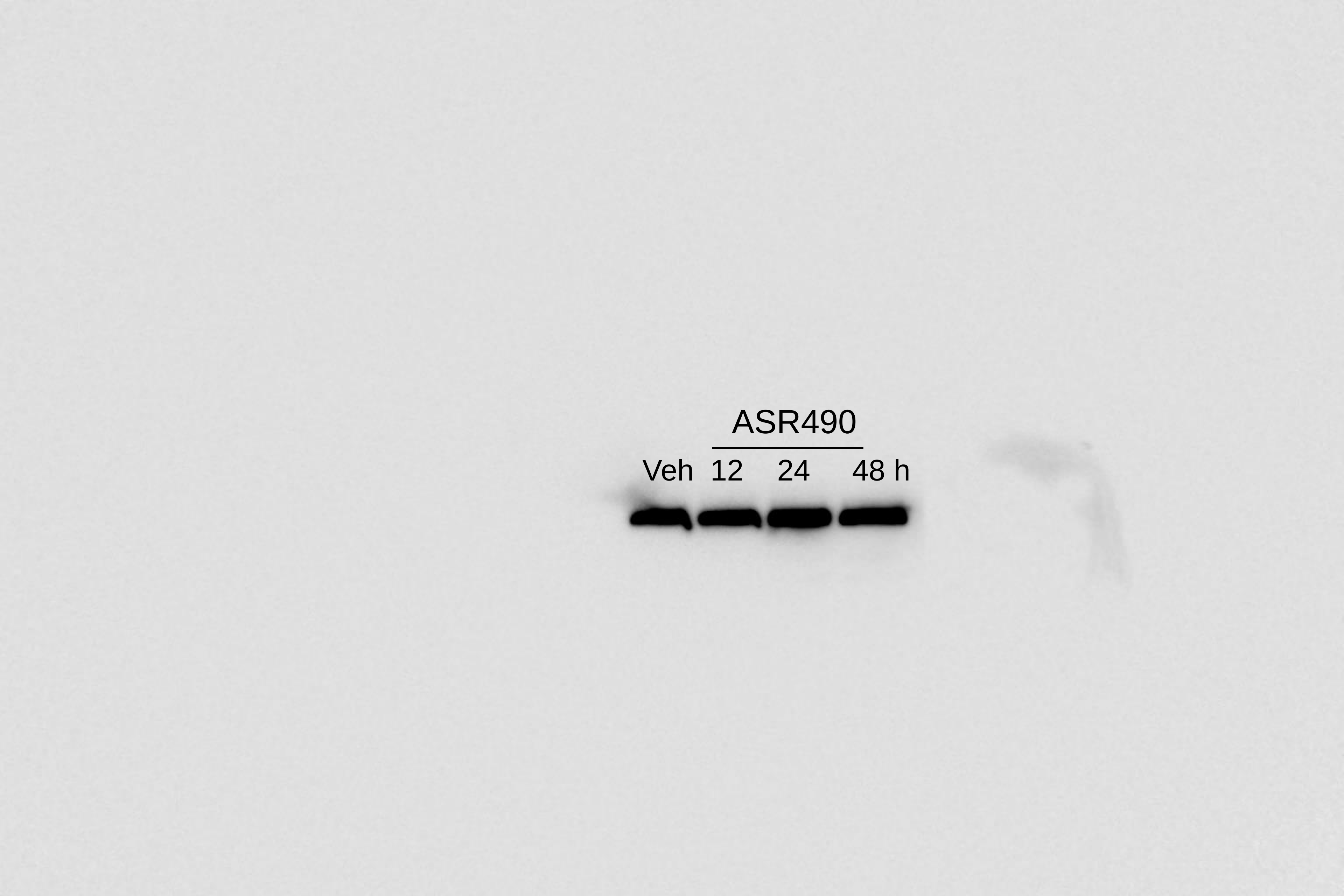

Figure 7D

E-cadherin

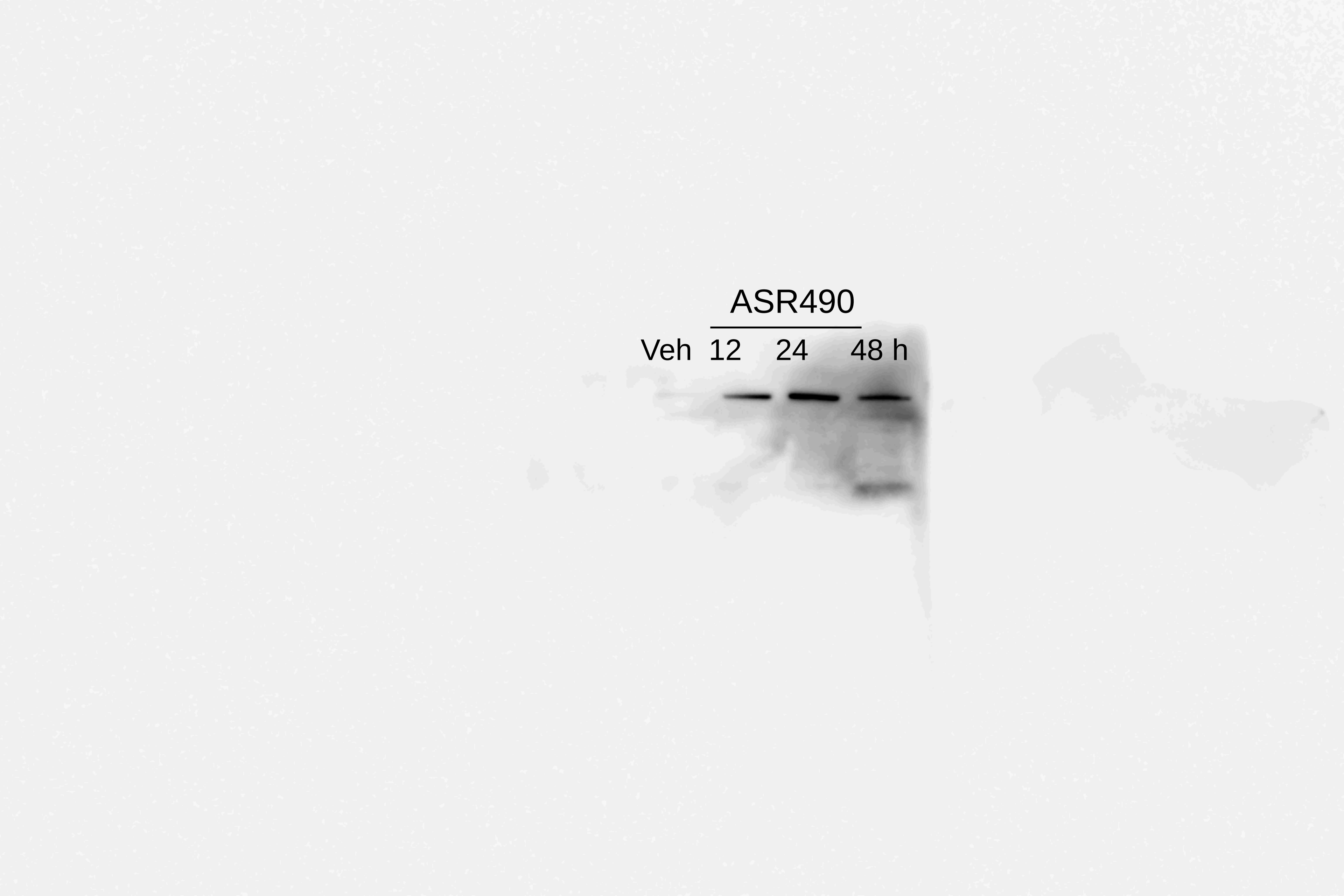

β-Catenin

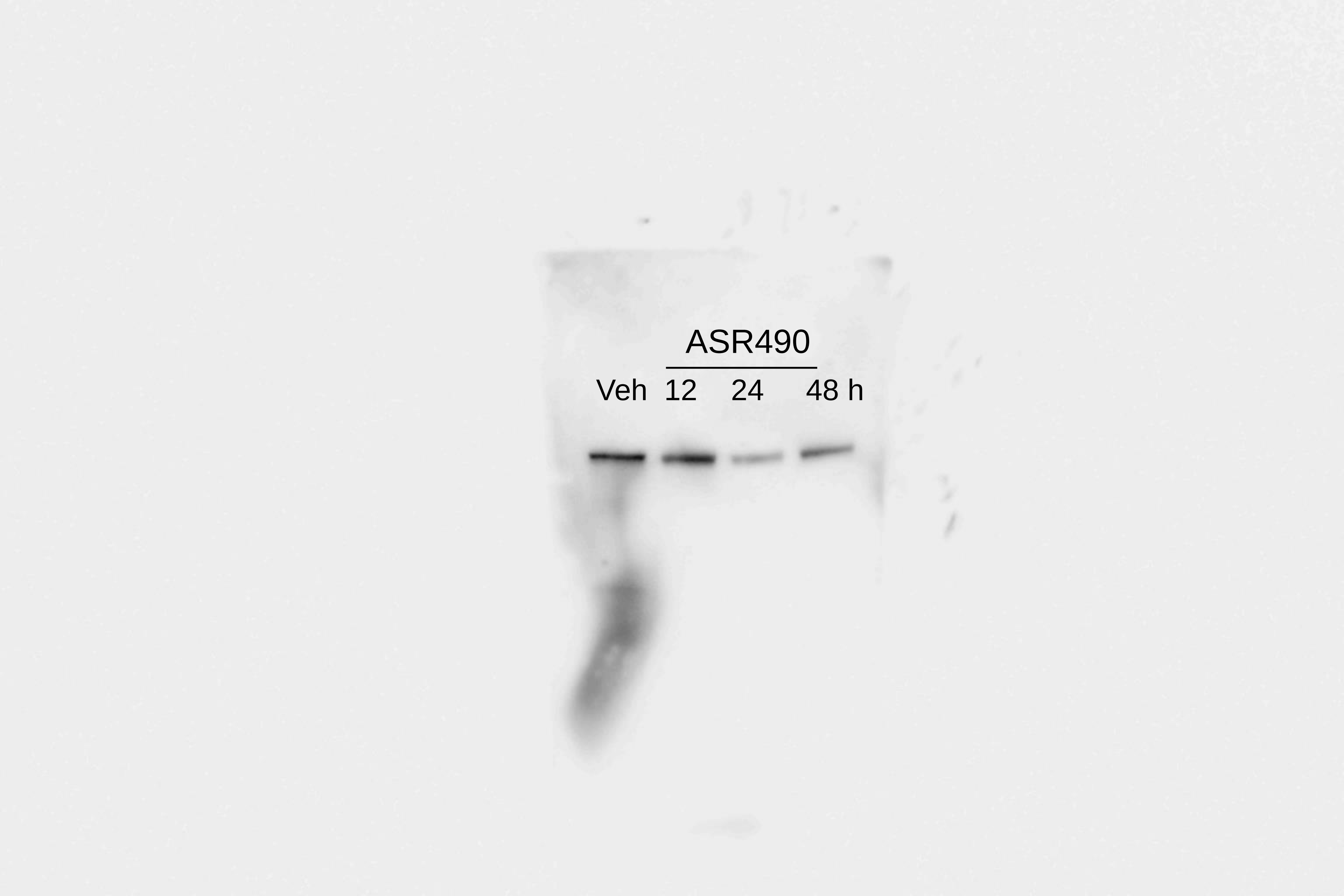

Slug

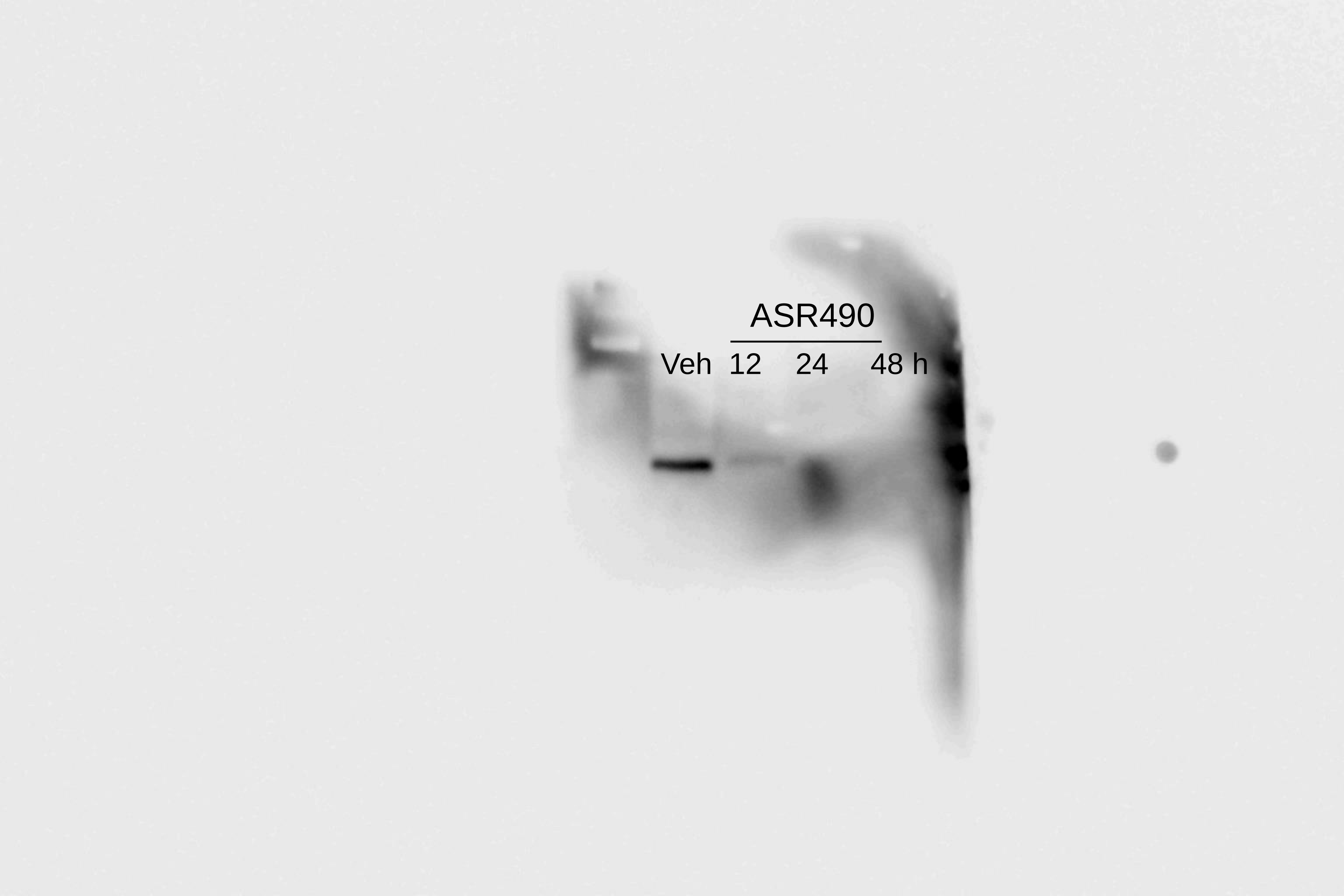

Vimentin

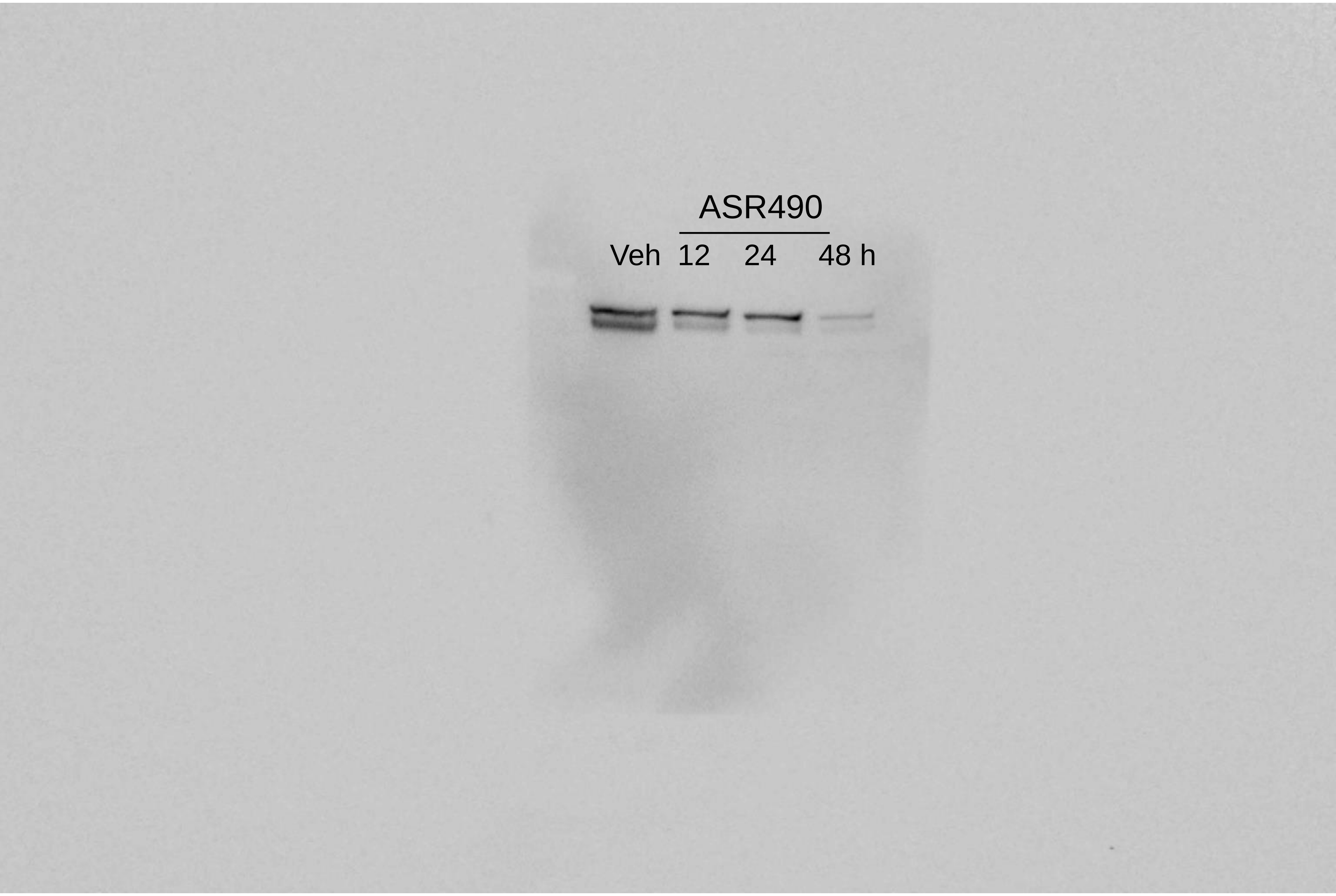

Actin

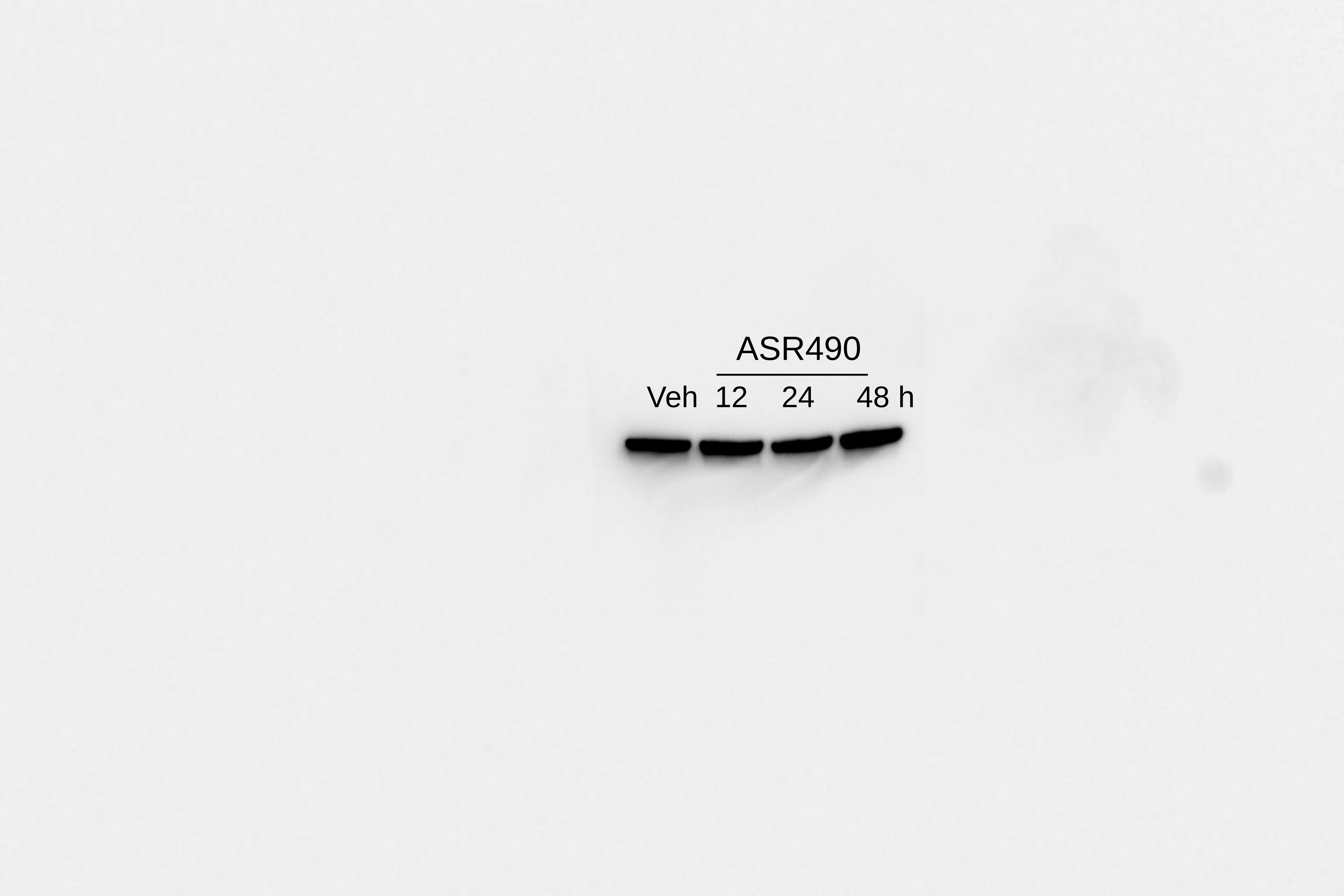

Figure 7E

E-cadherin

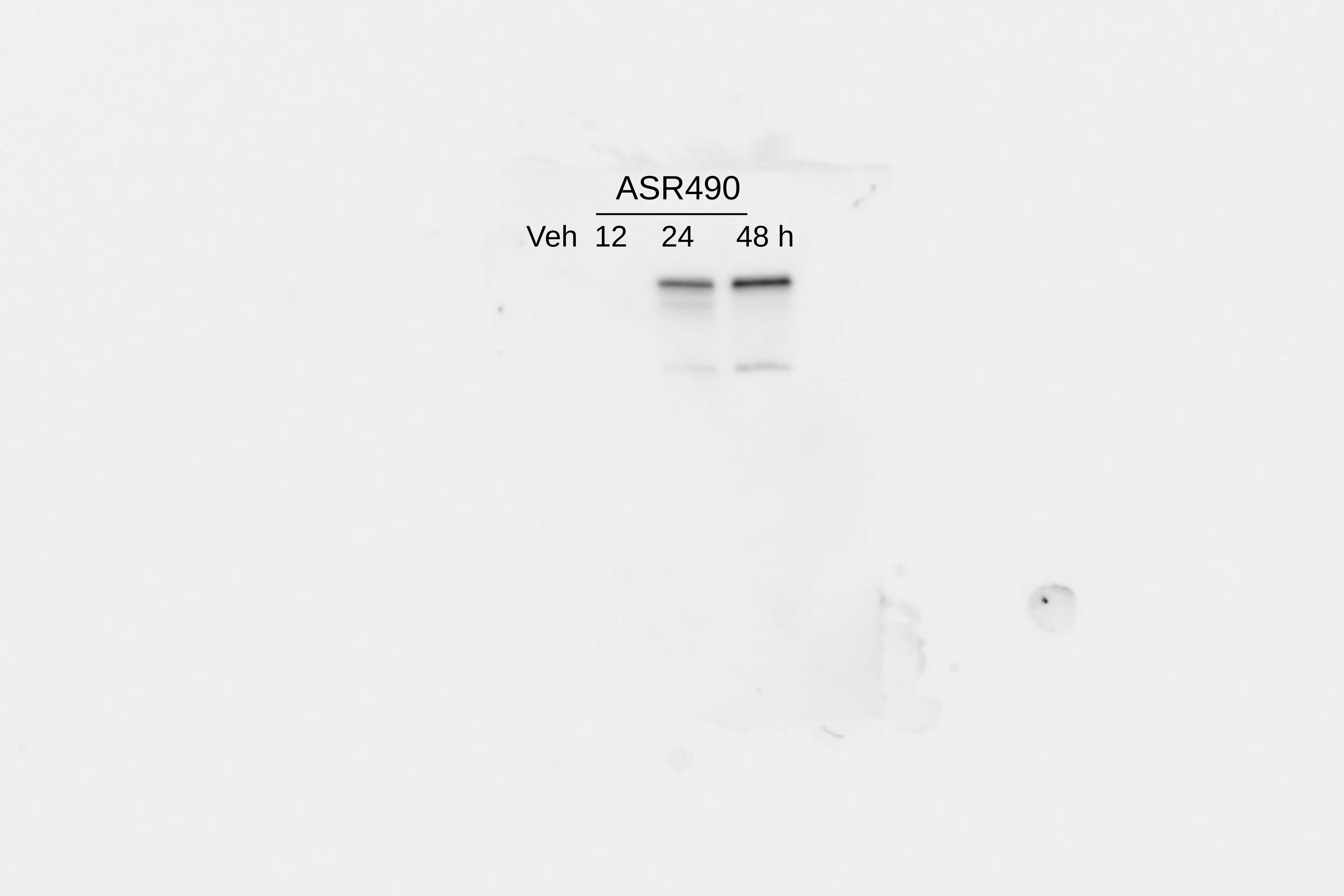

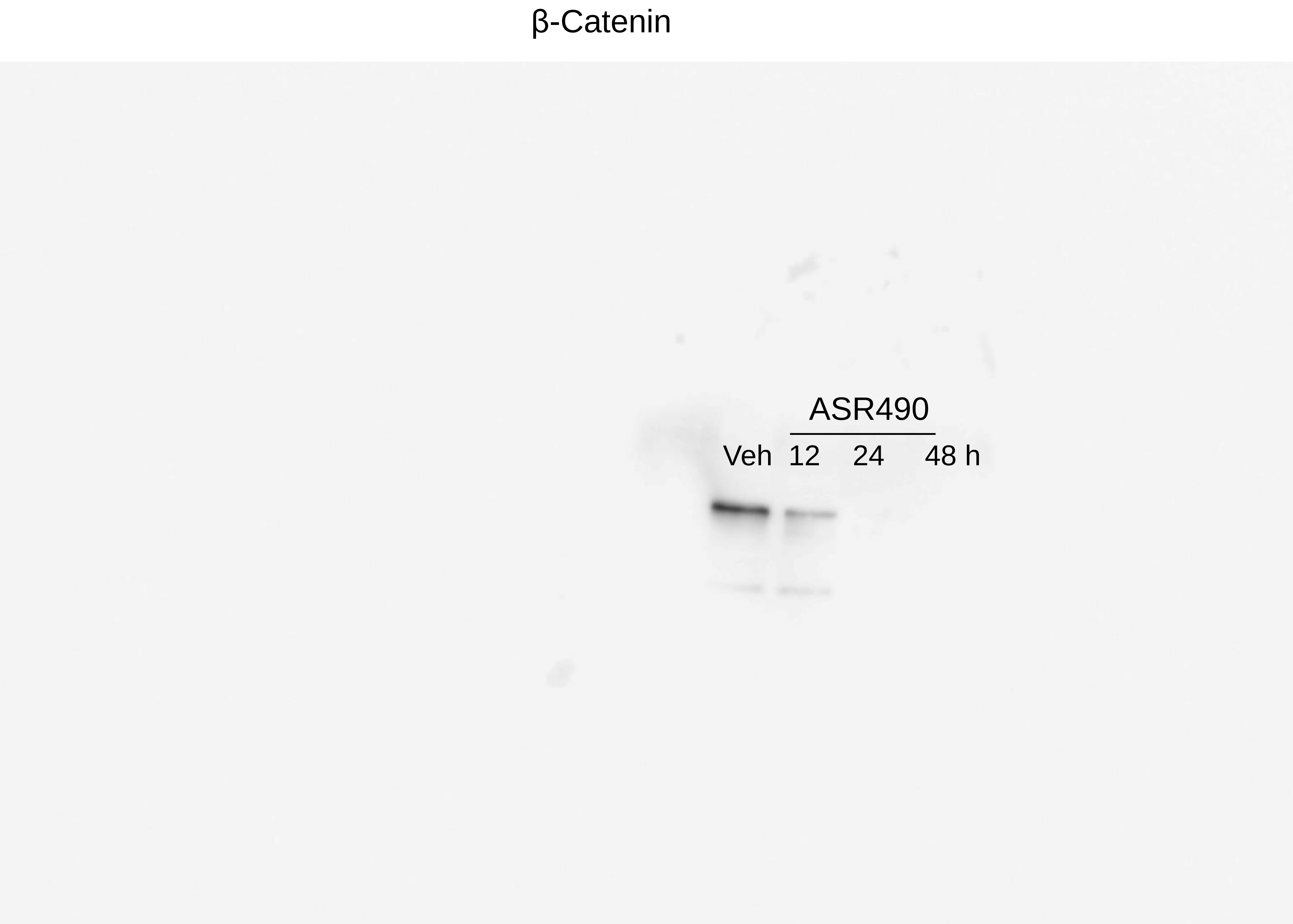
β-Catenin
ASR490
Veh
12
24
48 h

Slug

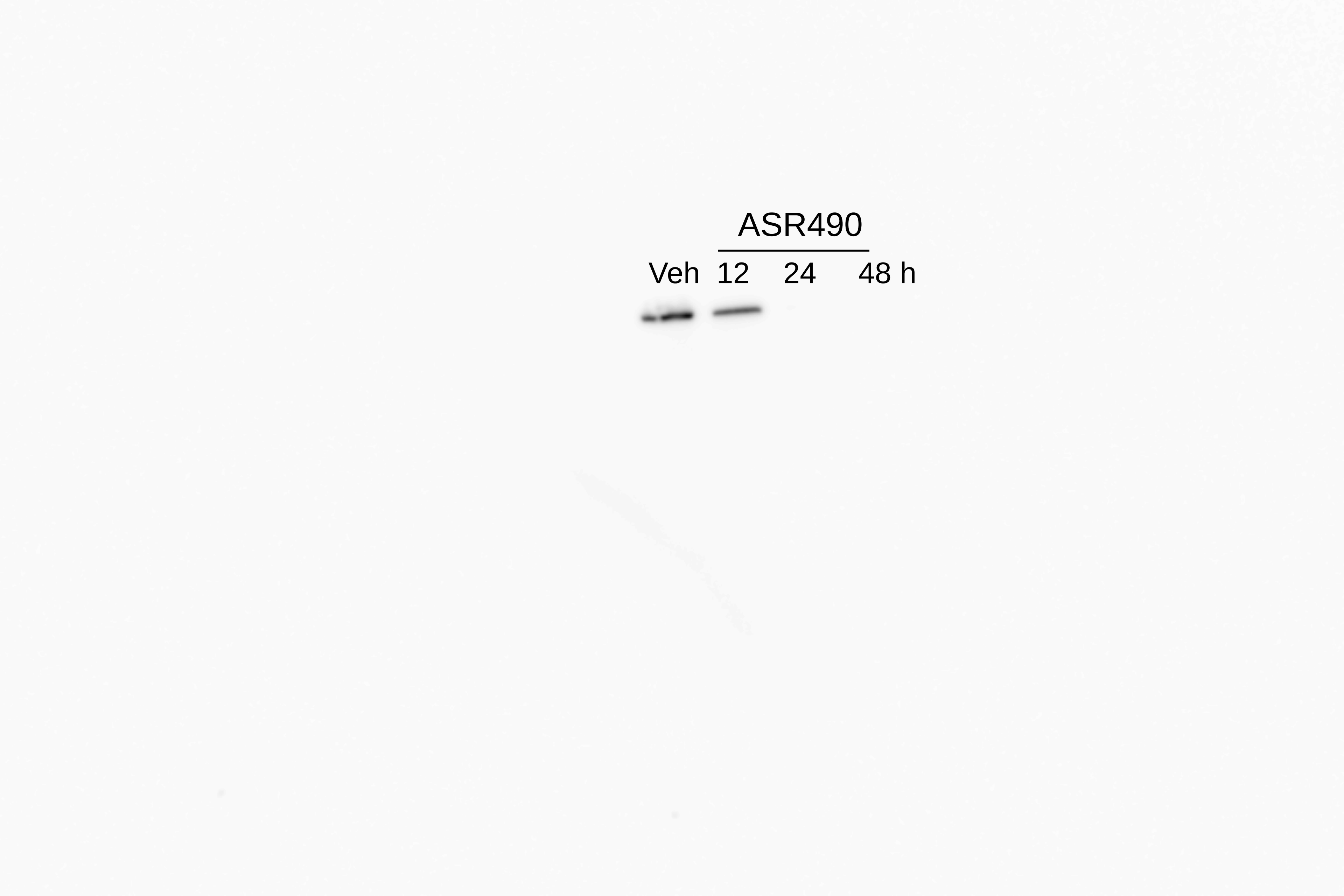

Vimentin

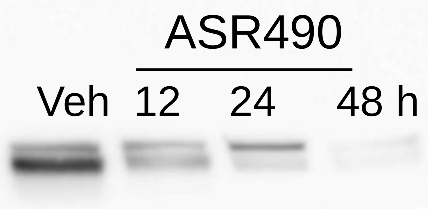

Actin

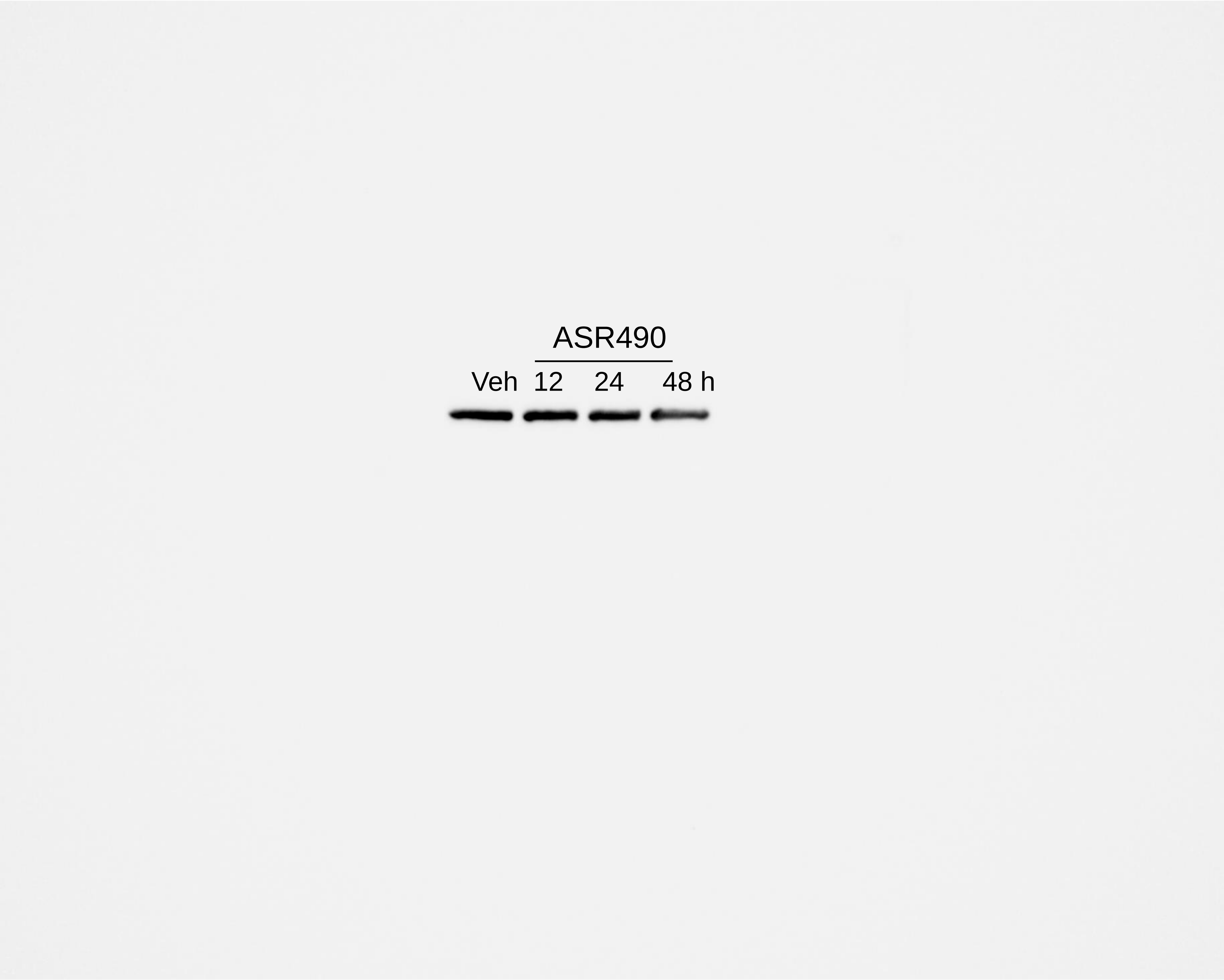

Figure 8B

p65

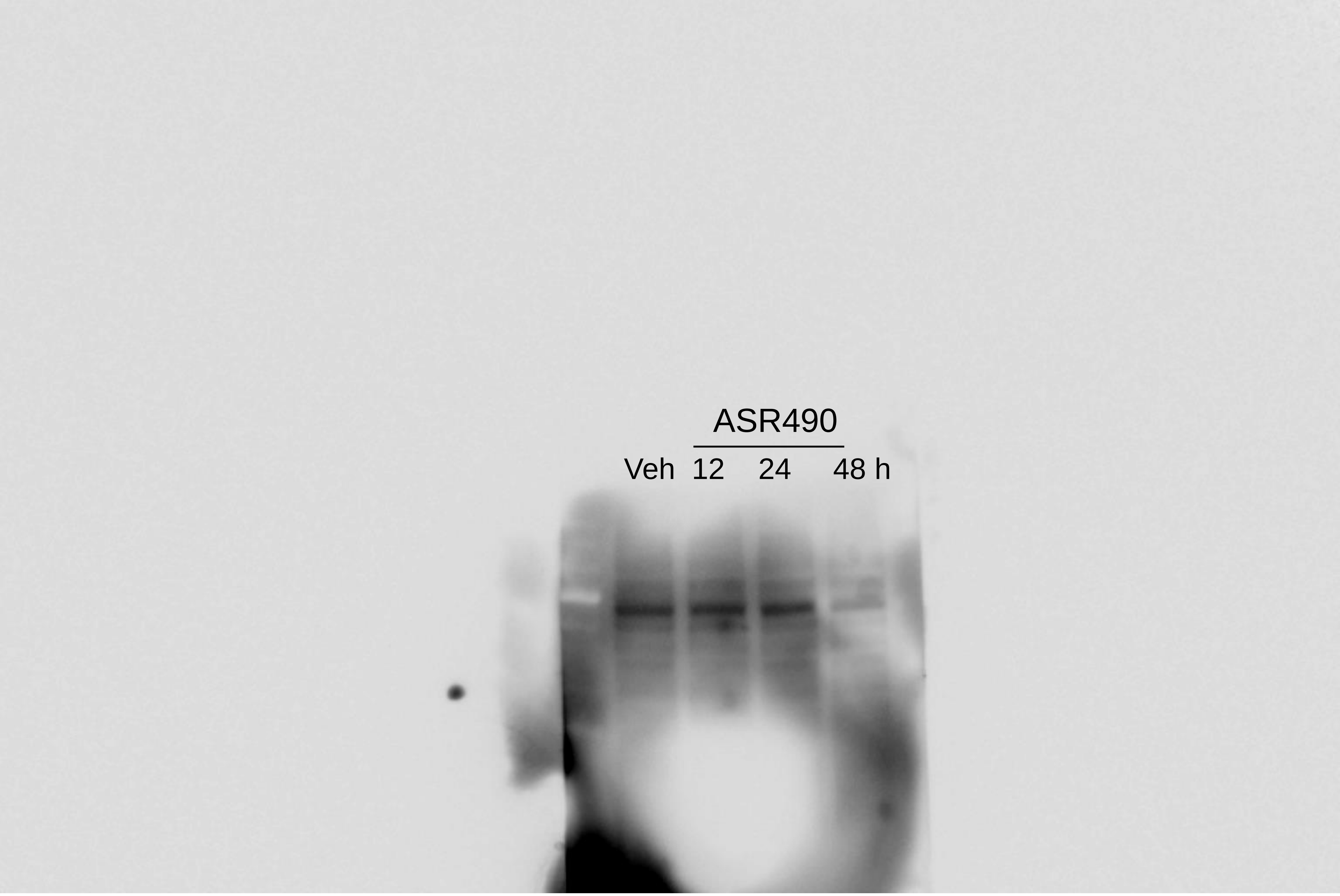

Bcl2

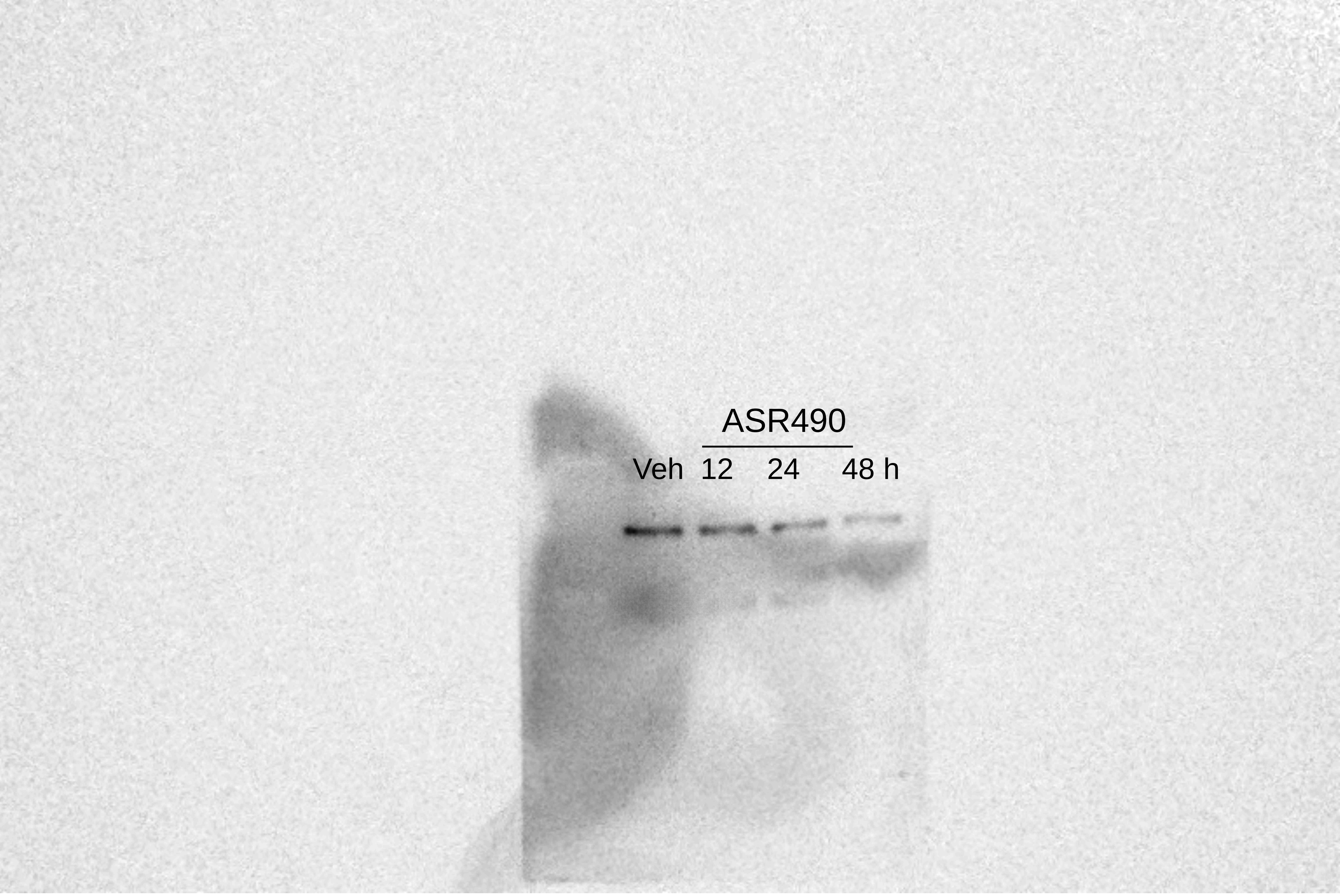

BClxL

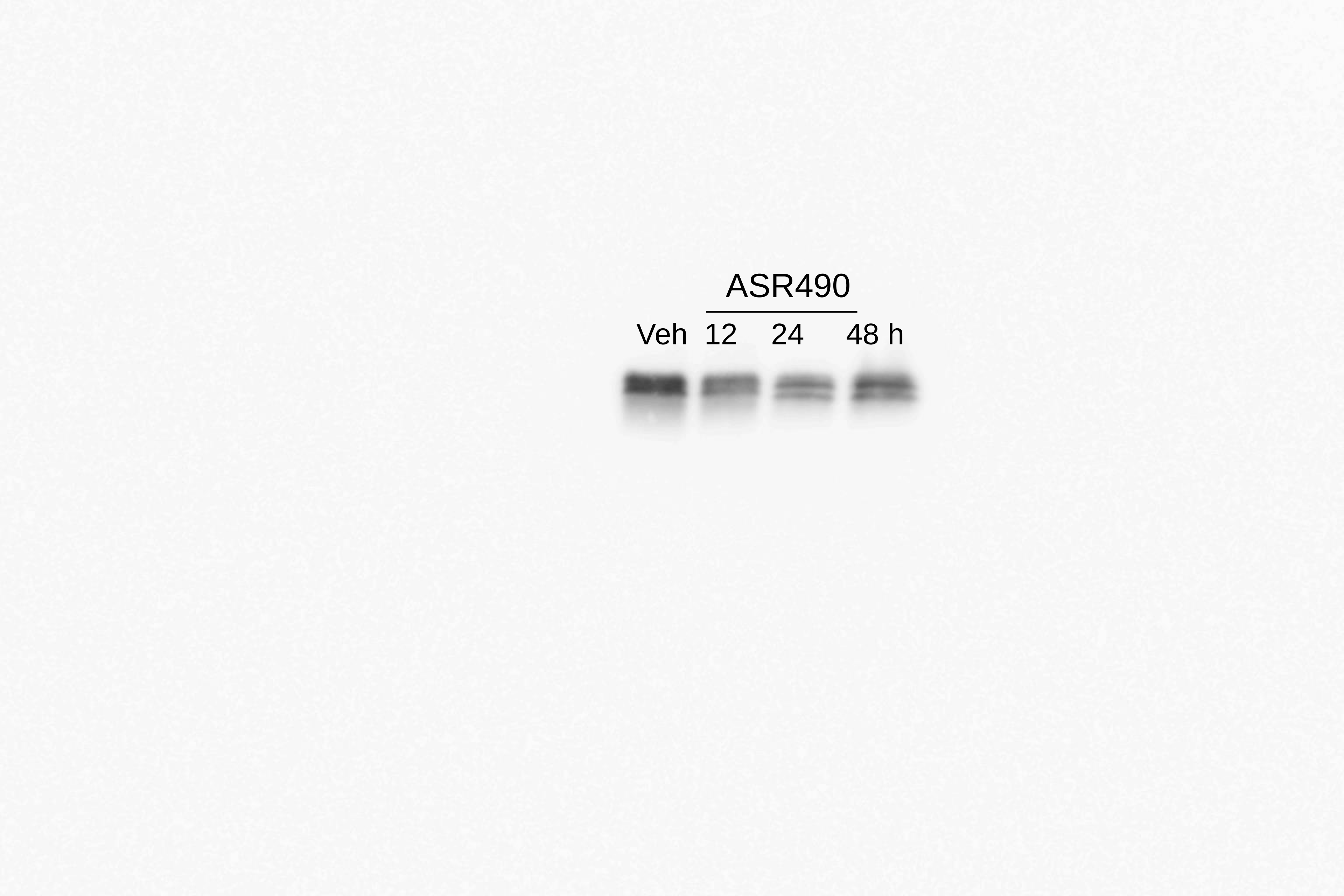

Actin

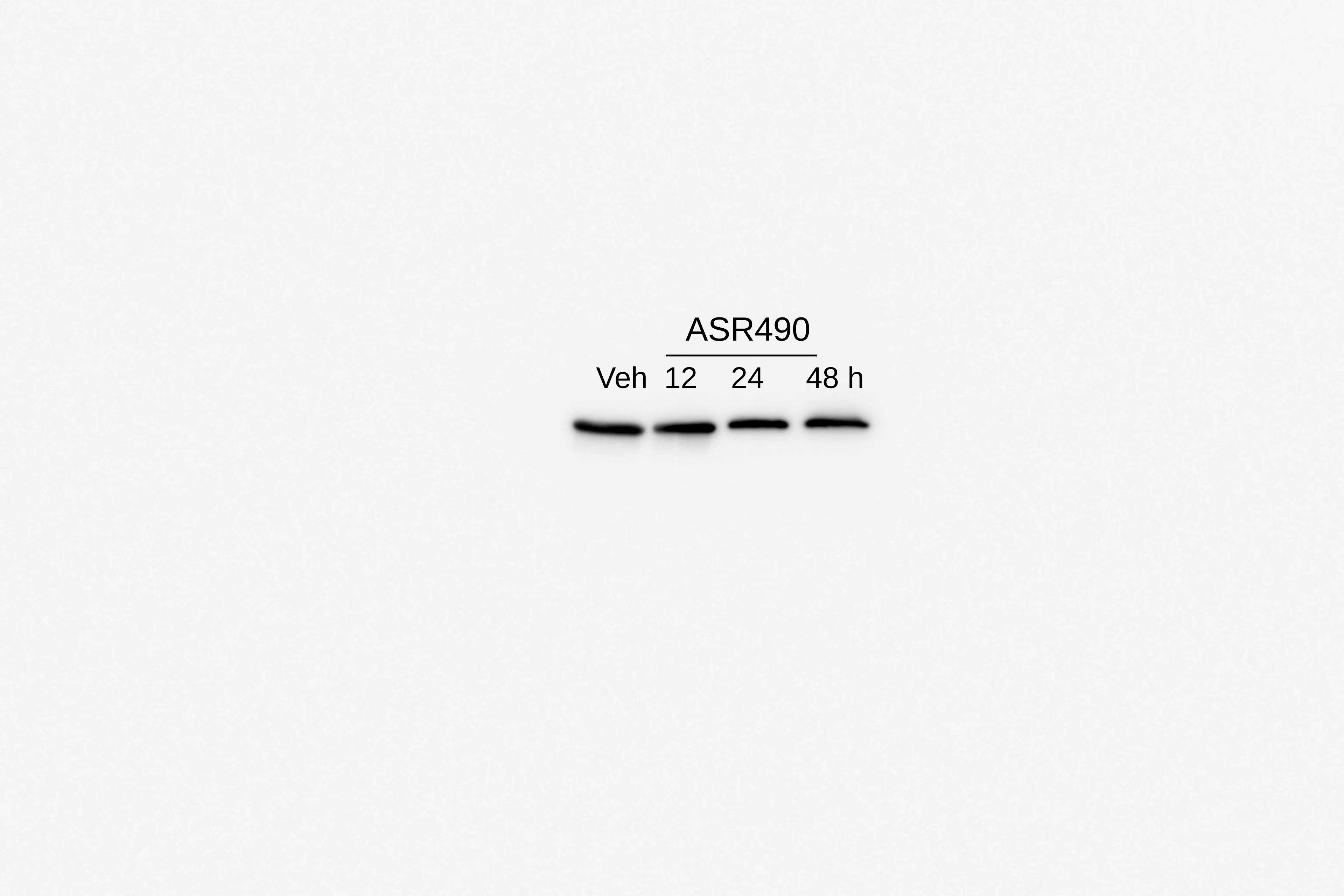

Figure 8C

p65

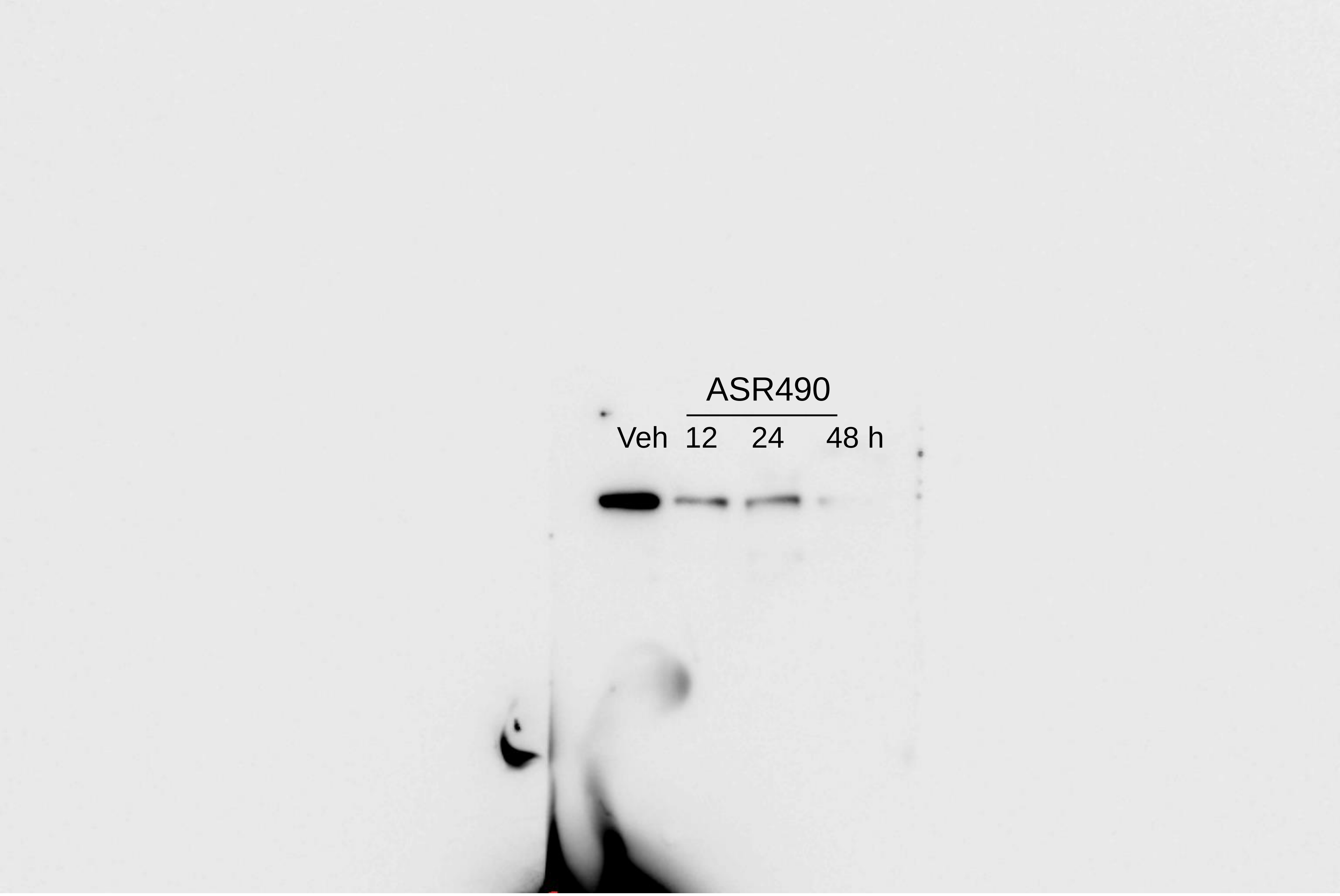

BCl2

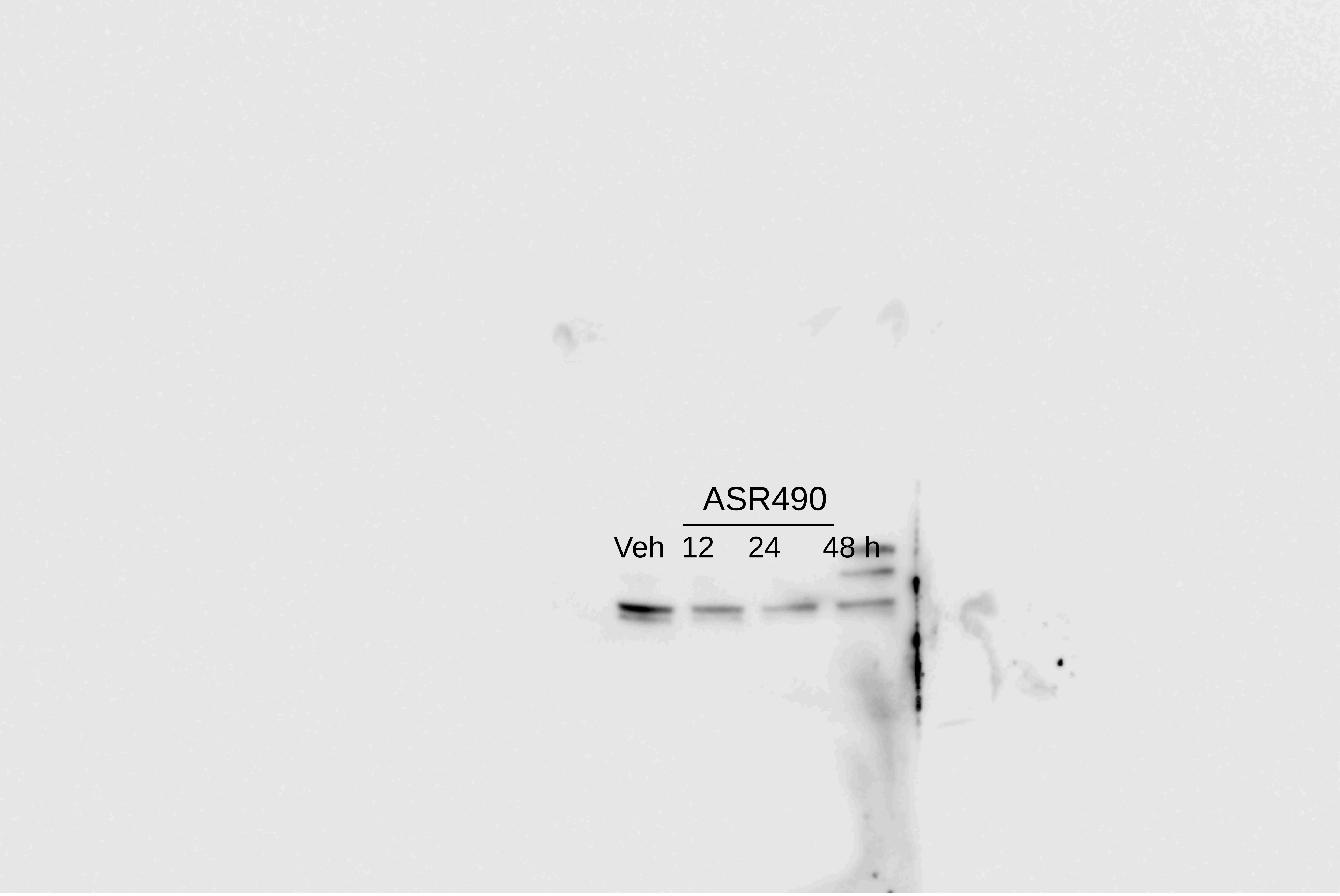

BClxL

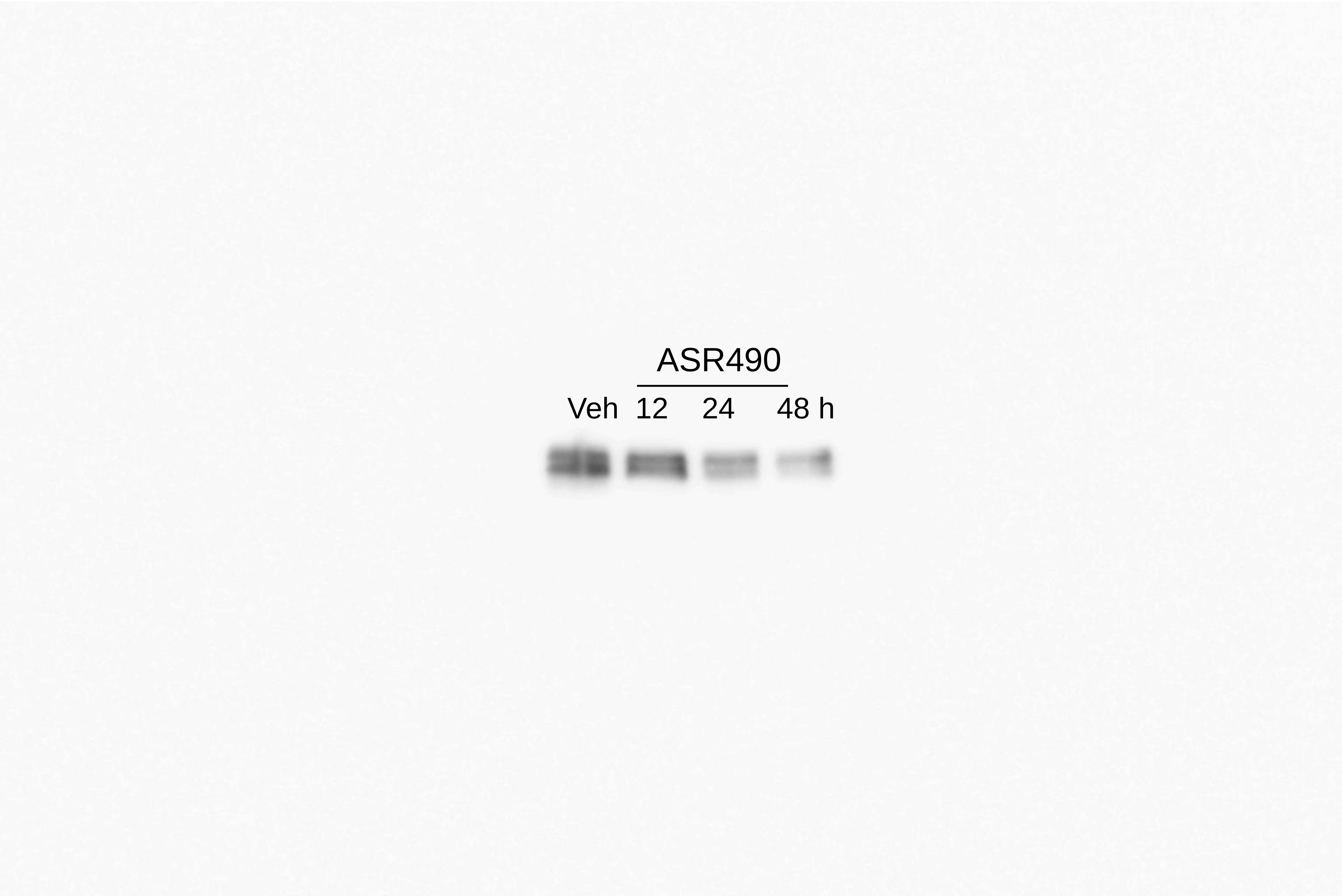

Actin

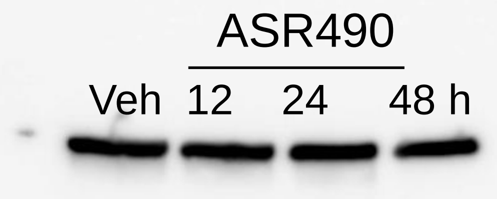

Figure 8D

p65

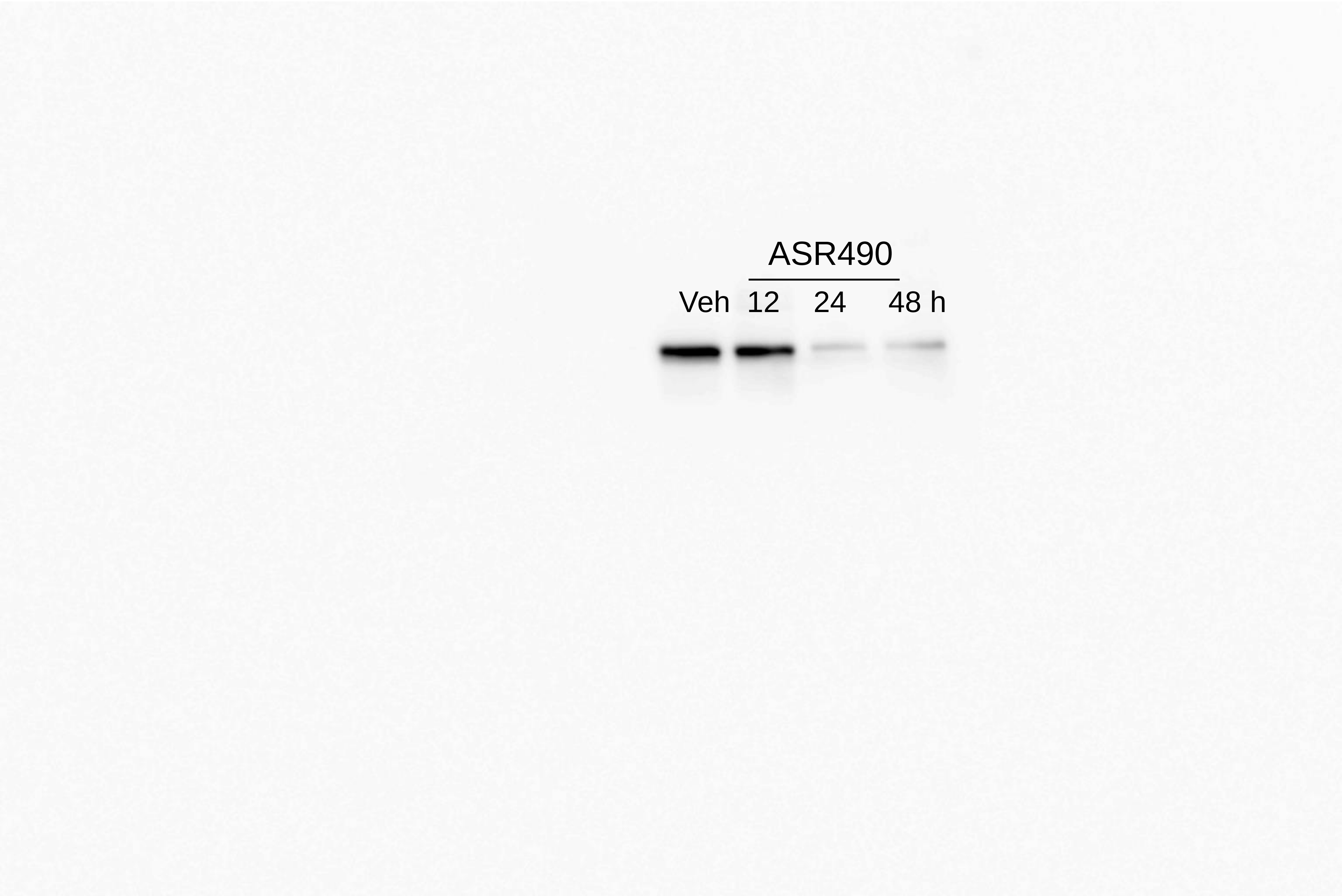

Bcl2

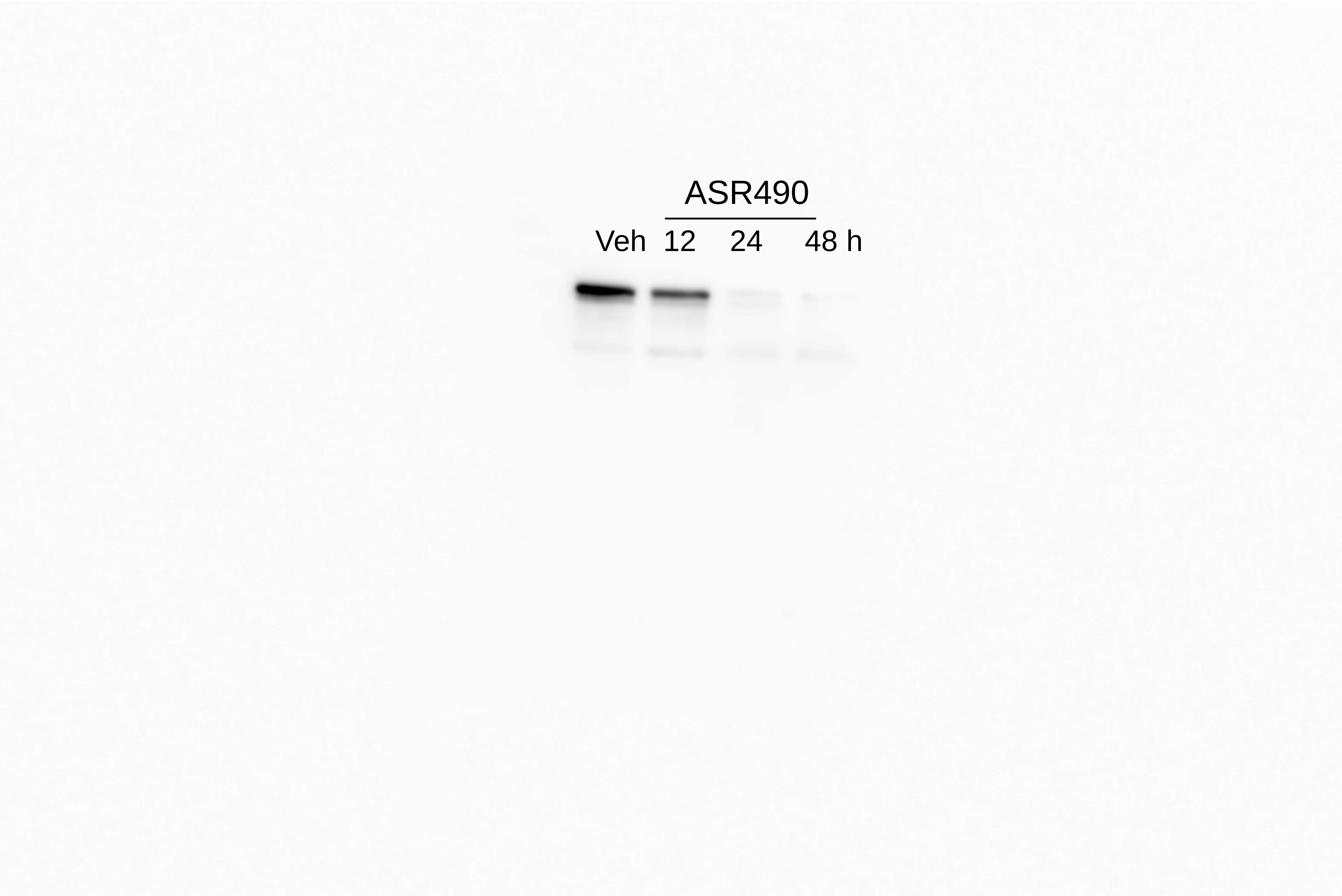

BclxL

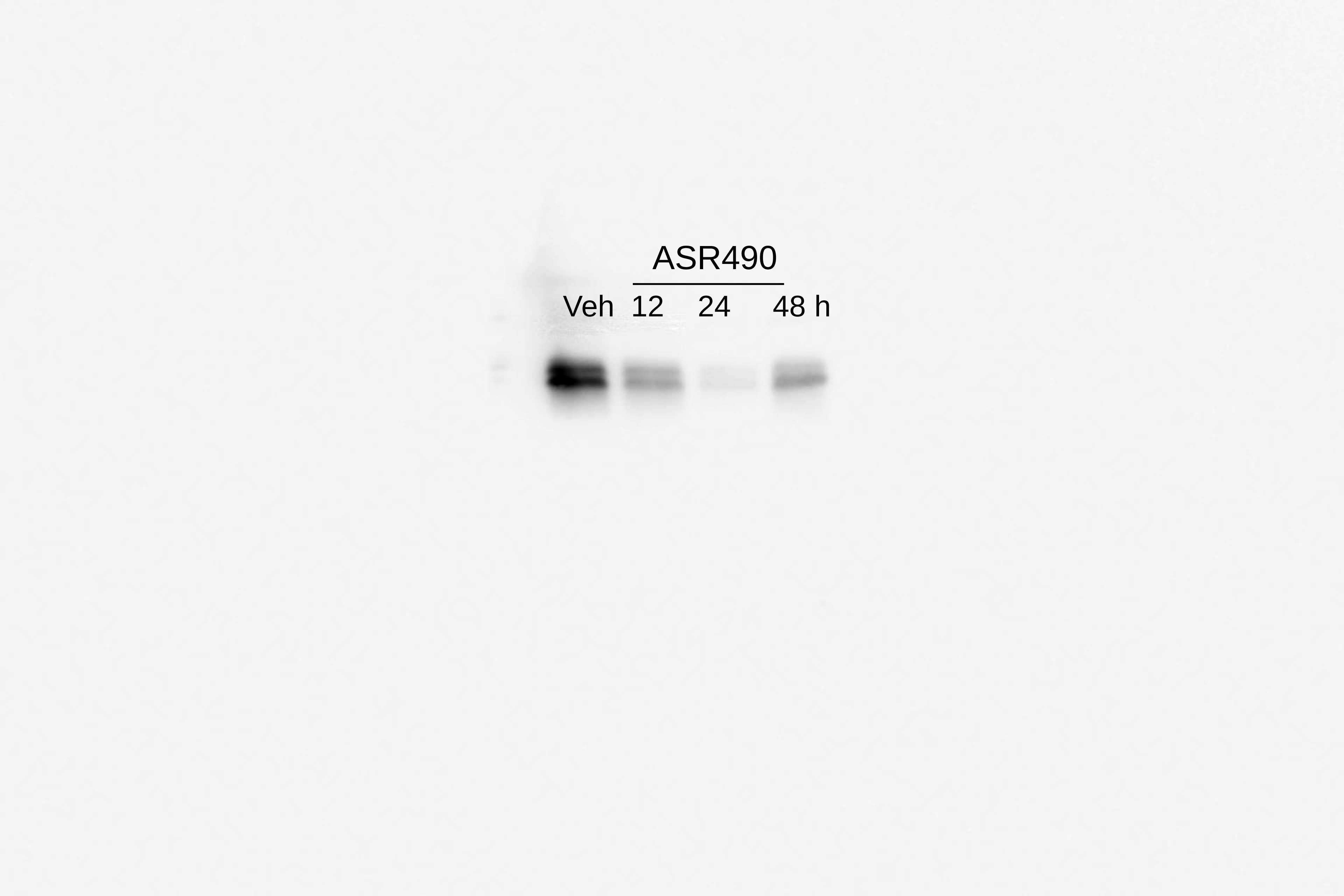

Actin

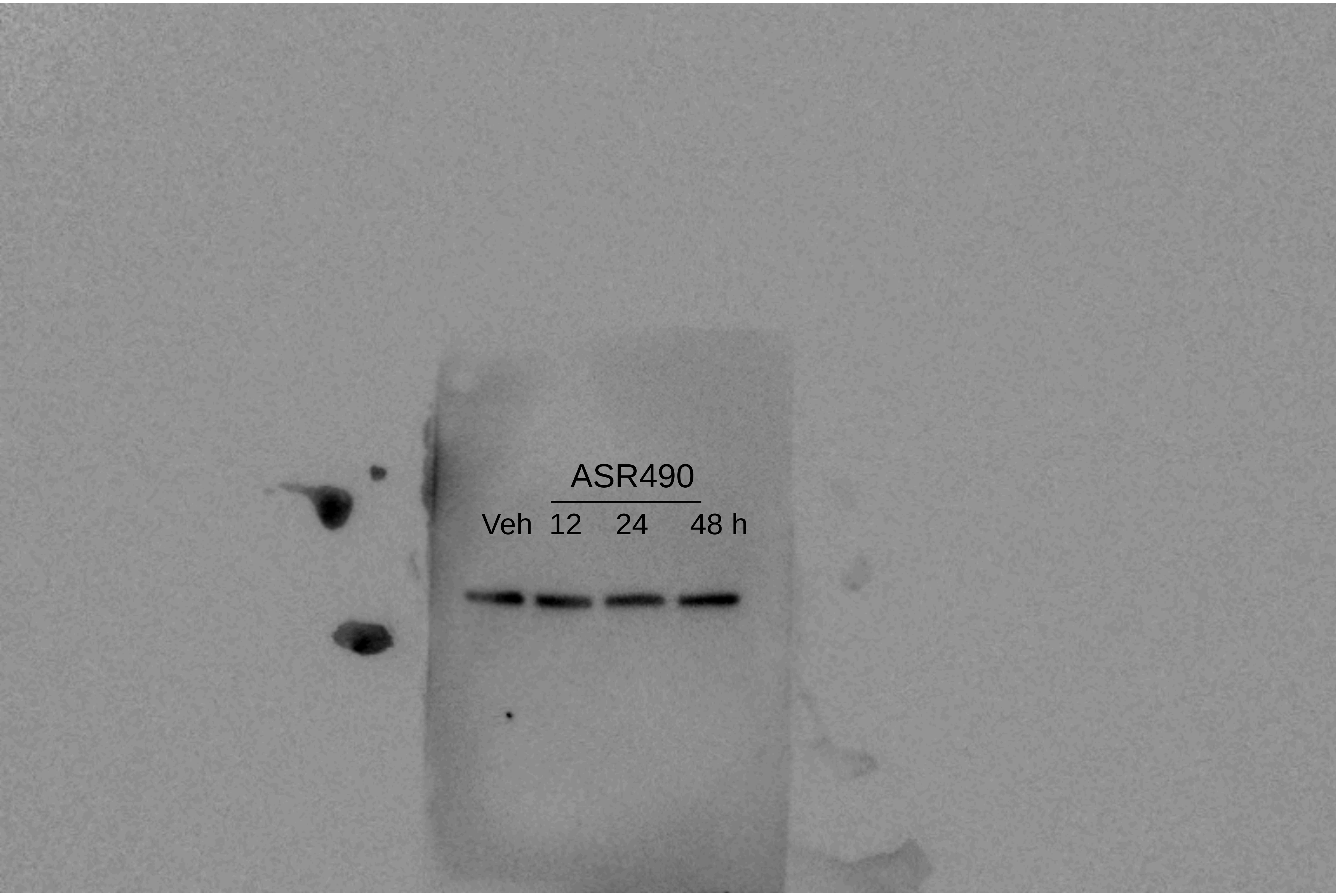

Figure 8F

Cl-PARP

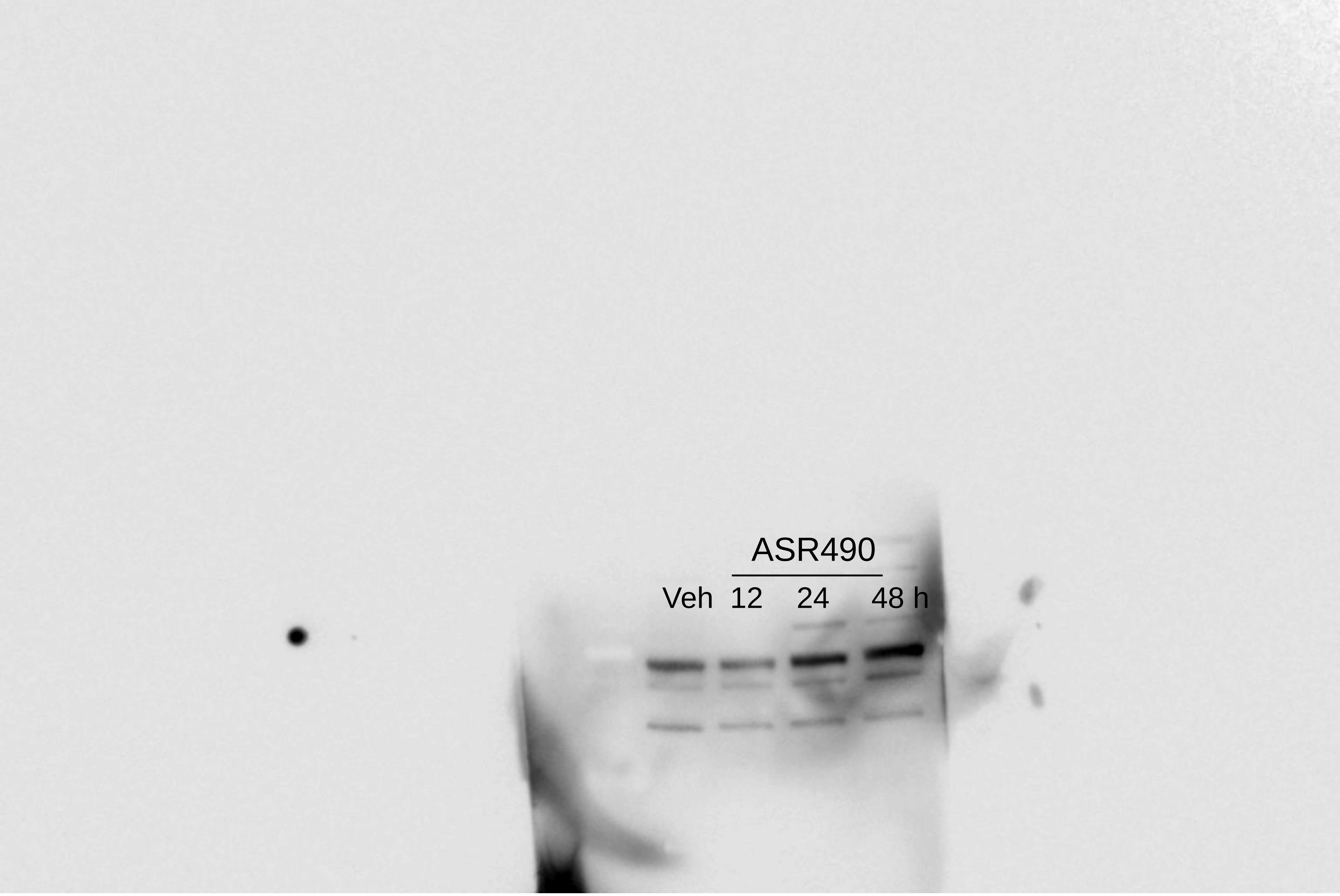

Cl-Cas 9

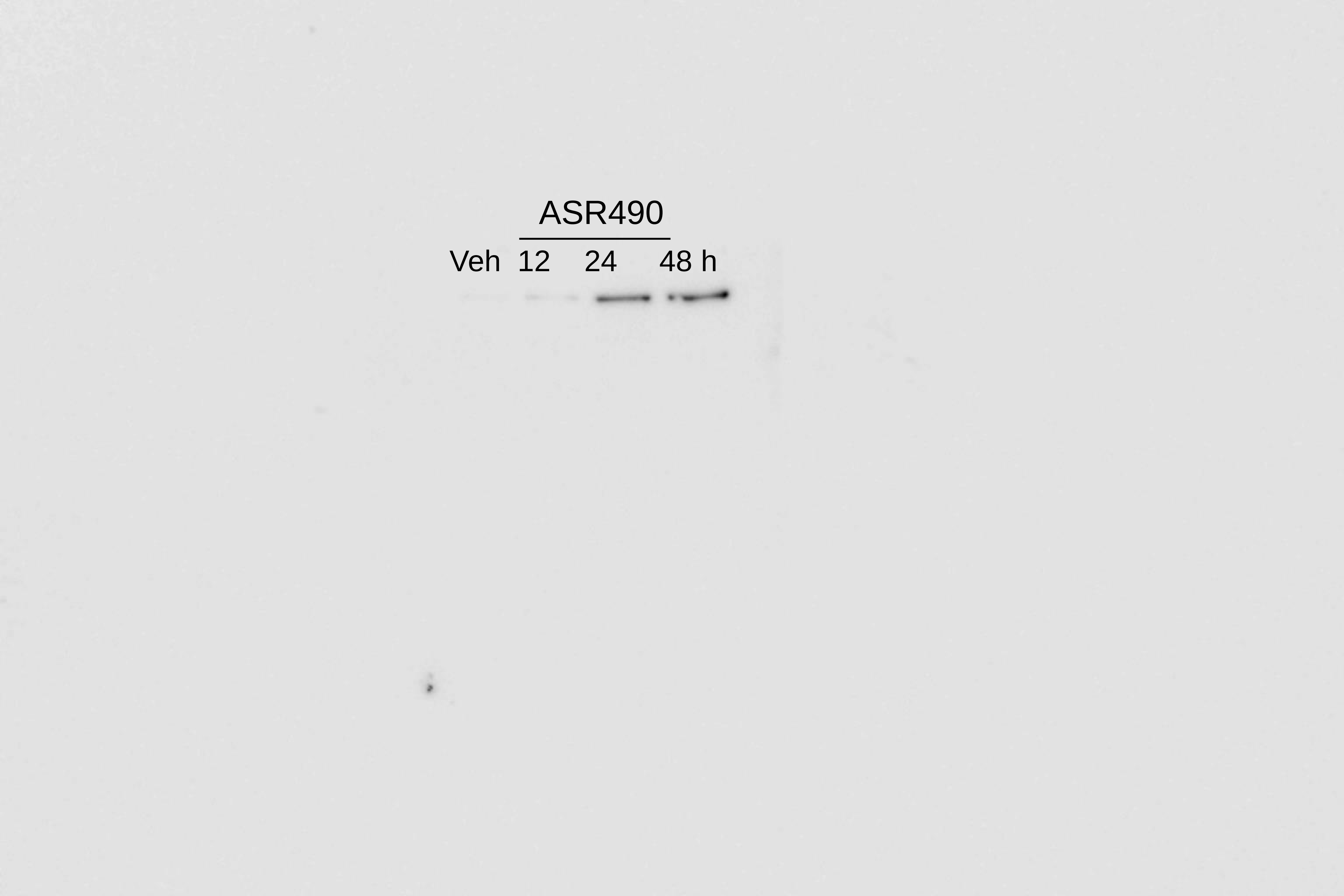

Bax

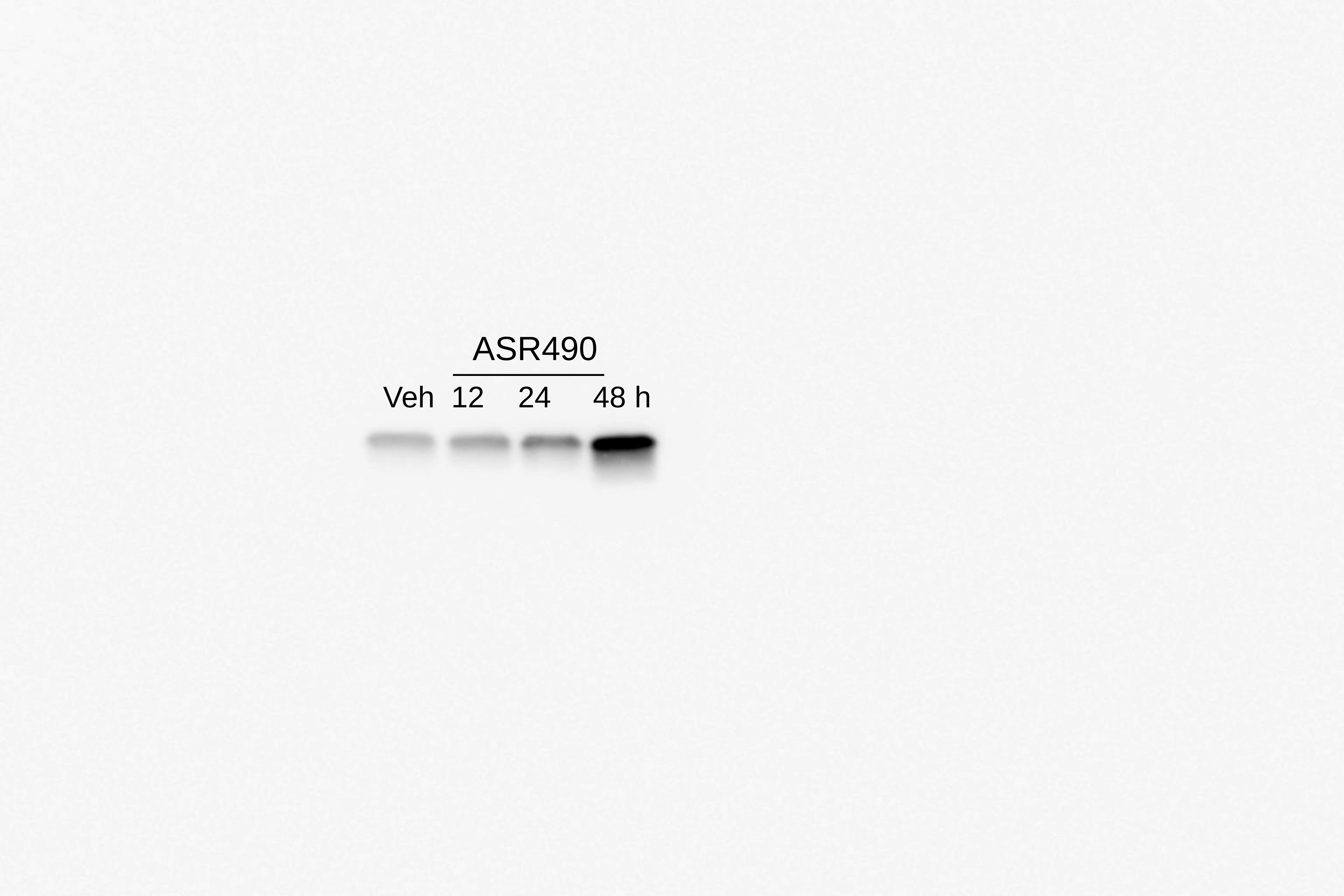

Actin

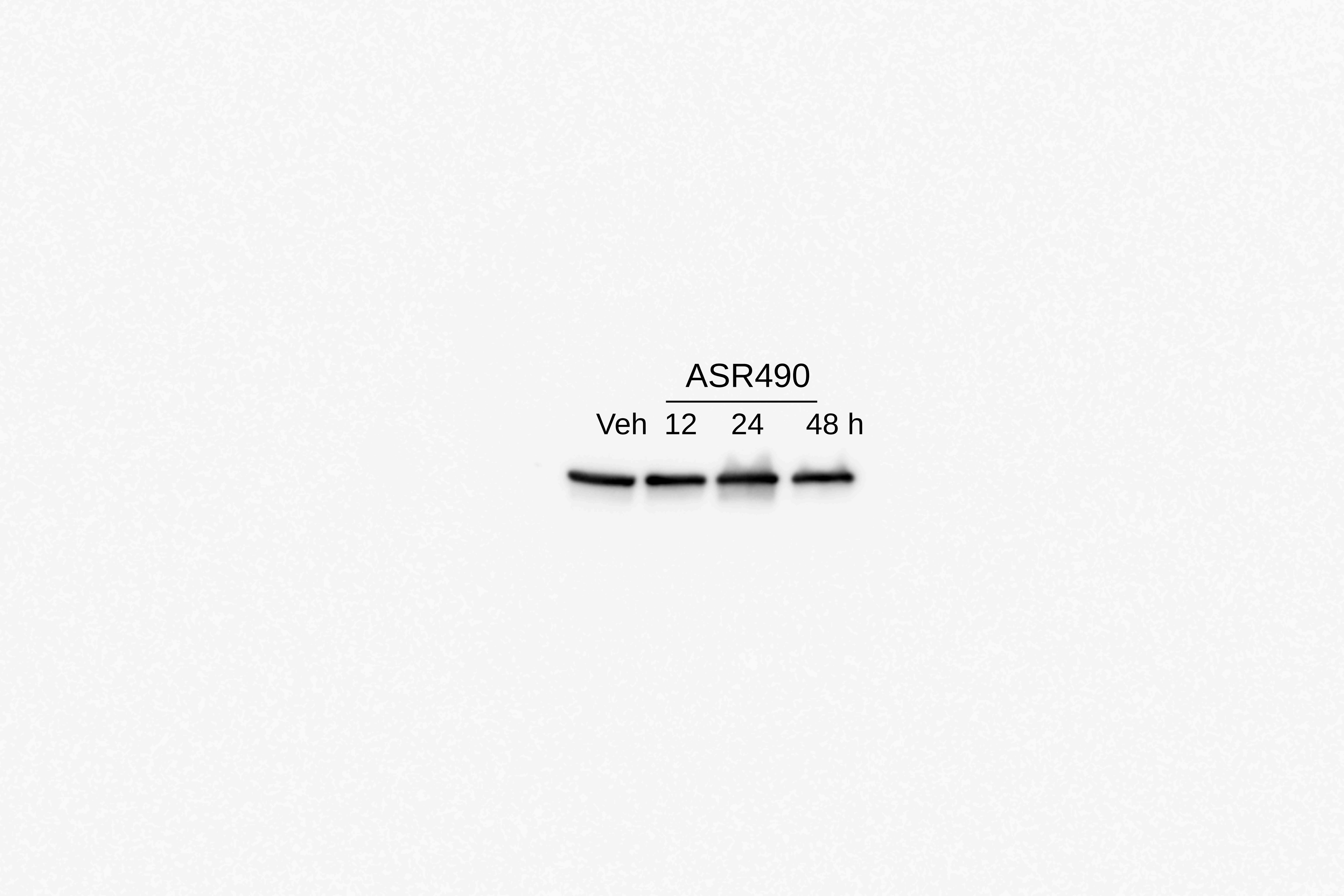

Figure 8G

Cl-PARP

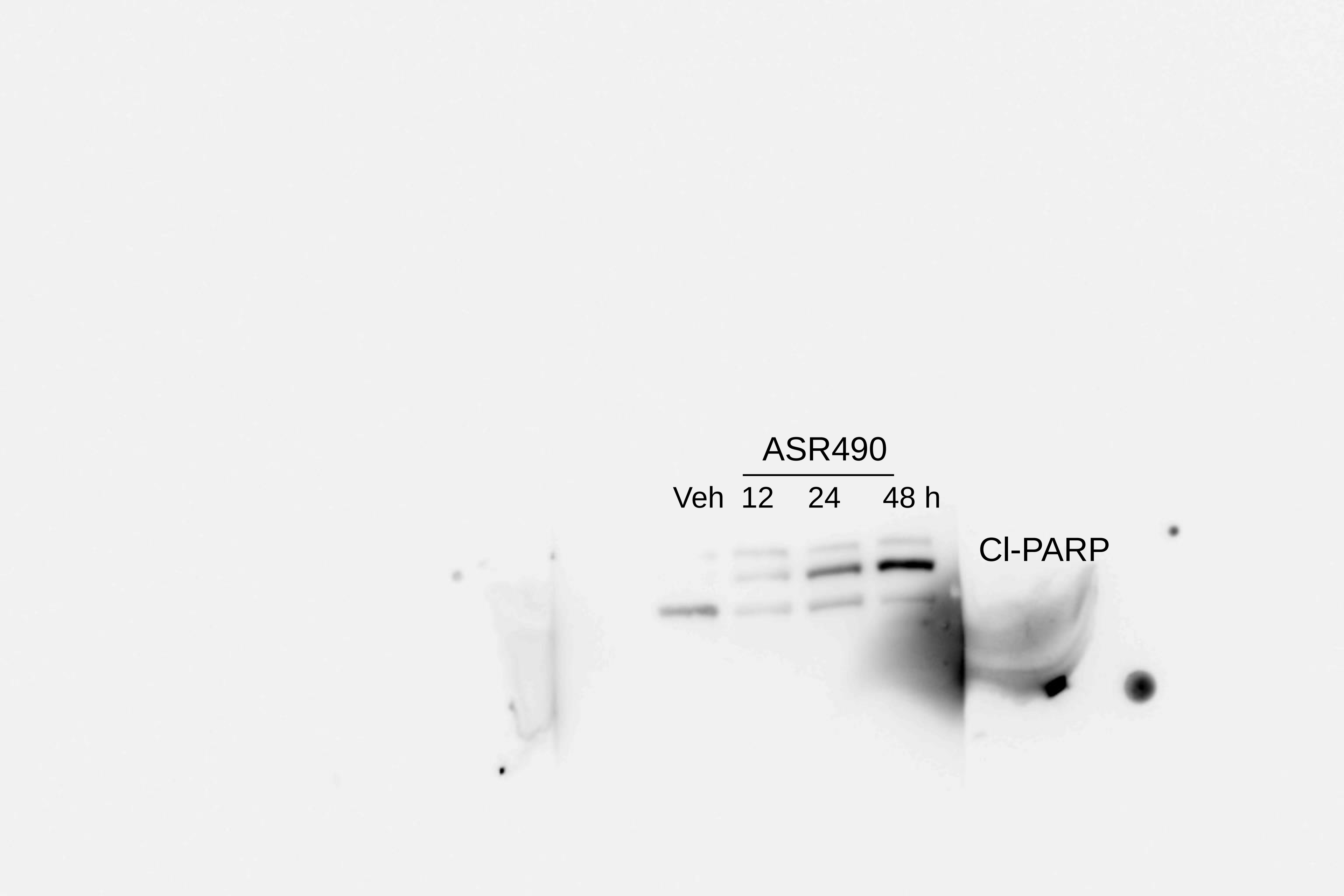

Cl-Cas 9

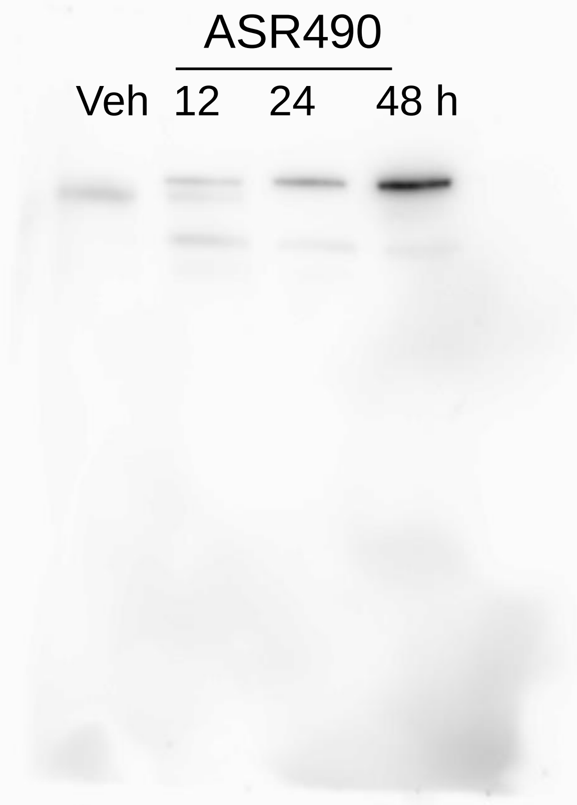

Bax

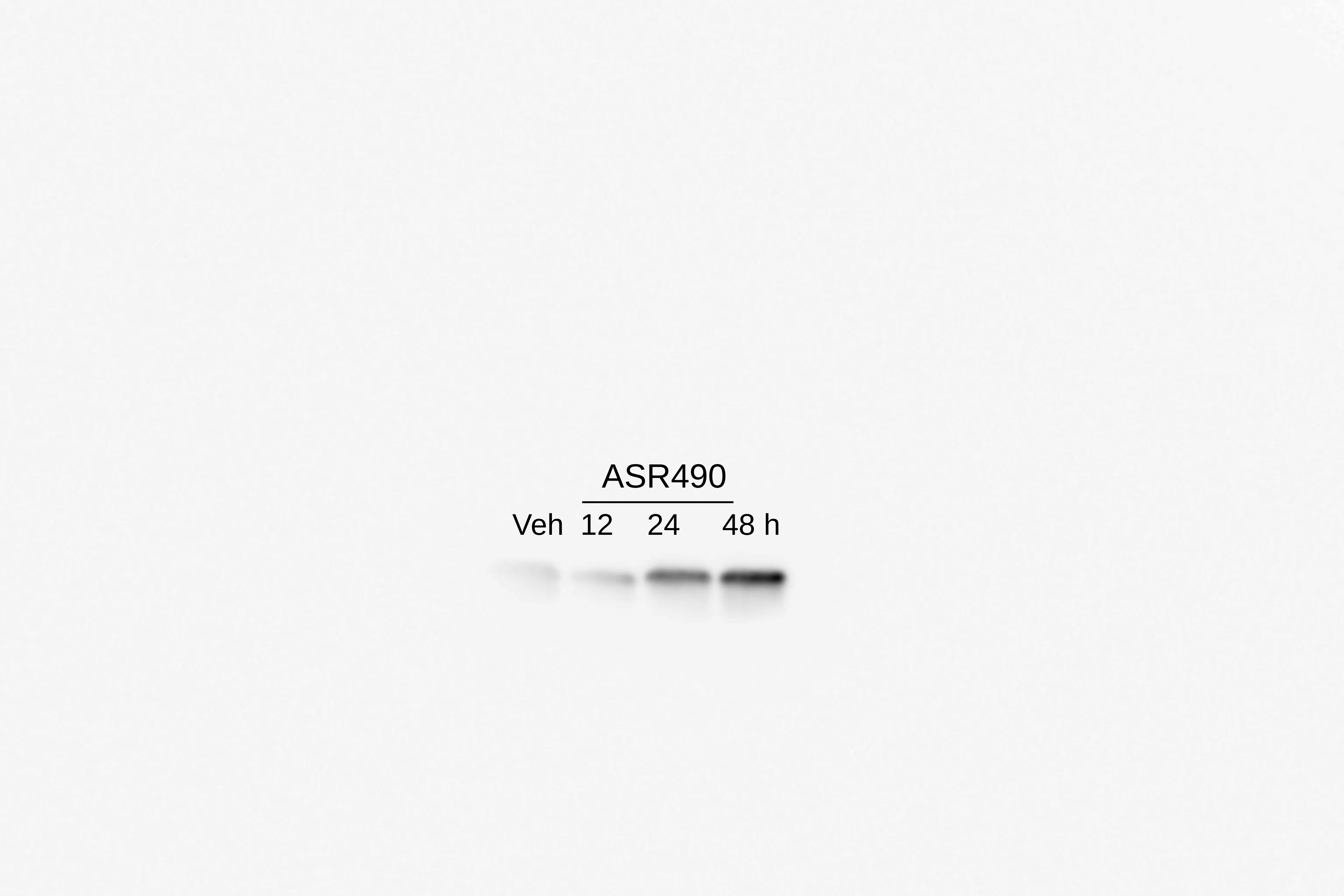

Actin

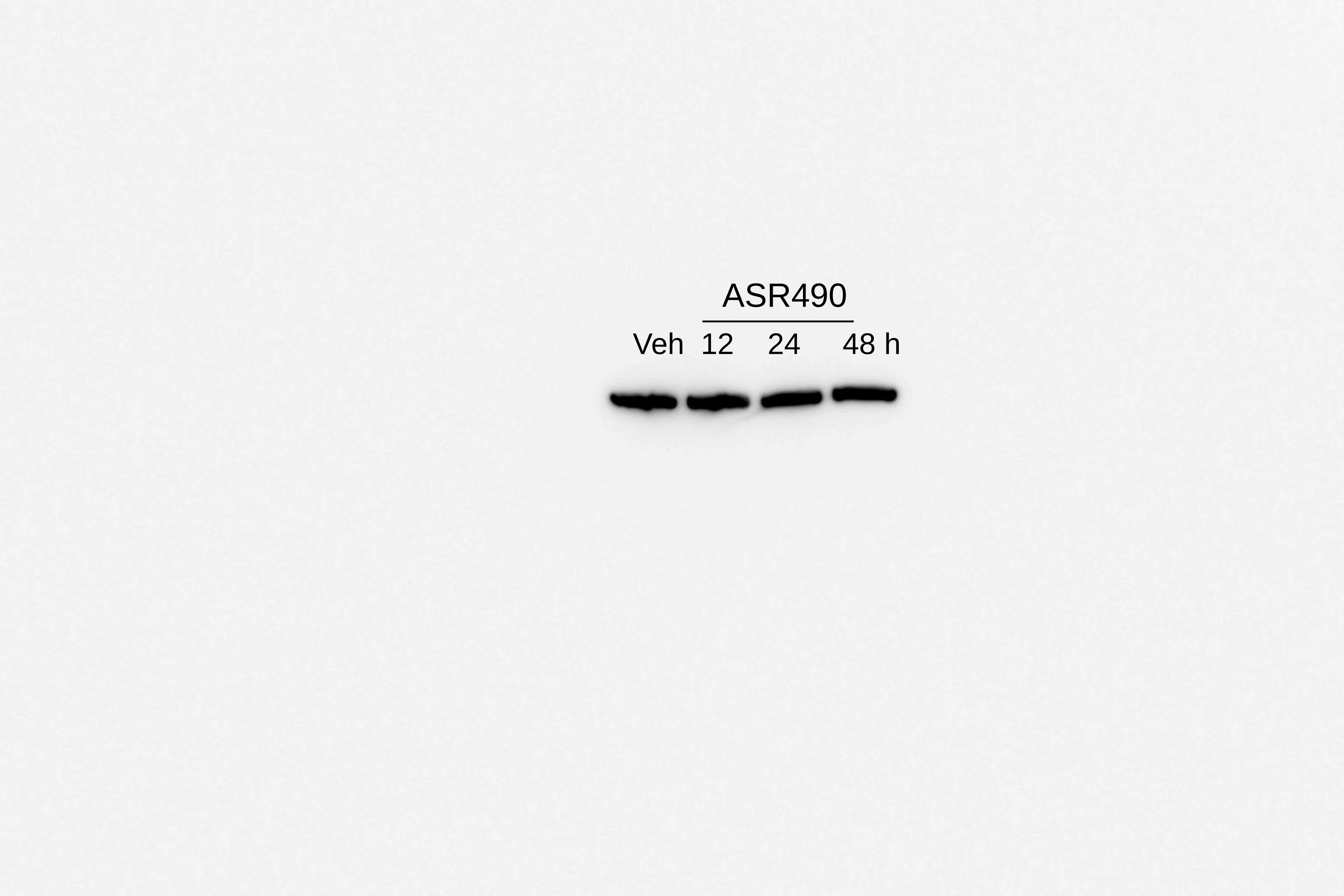

Figure 8H

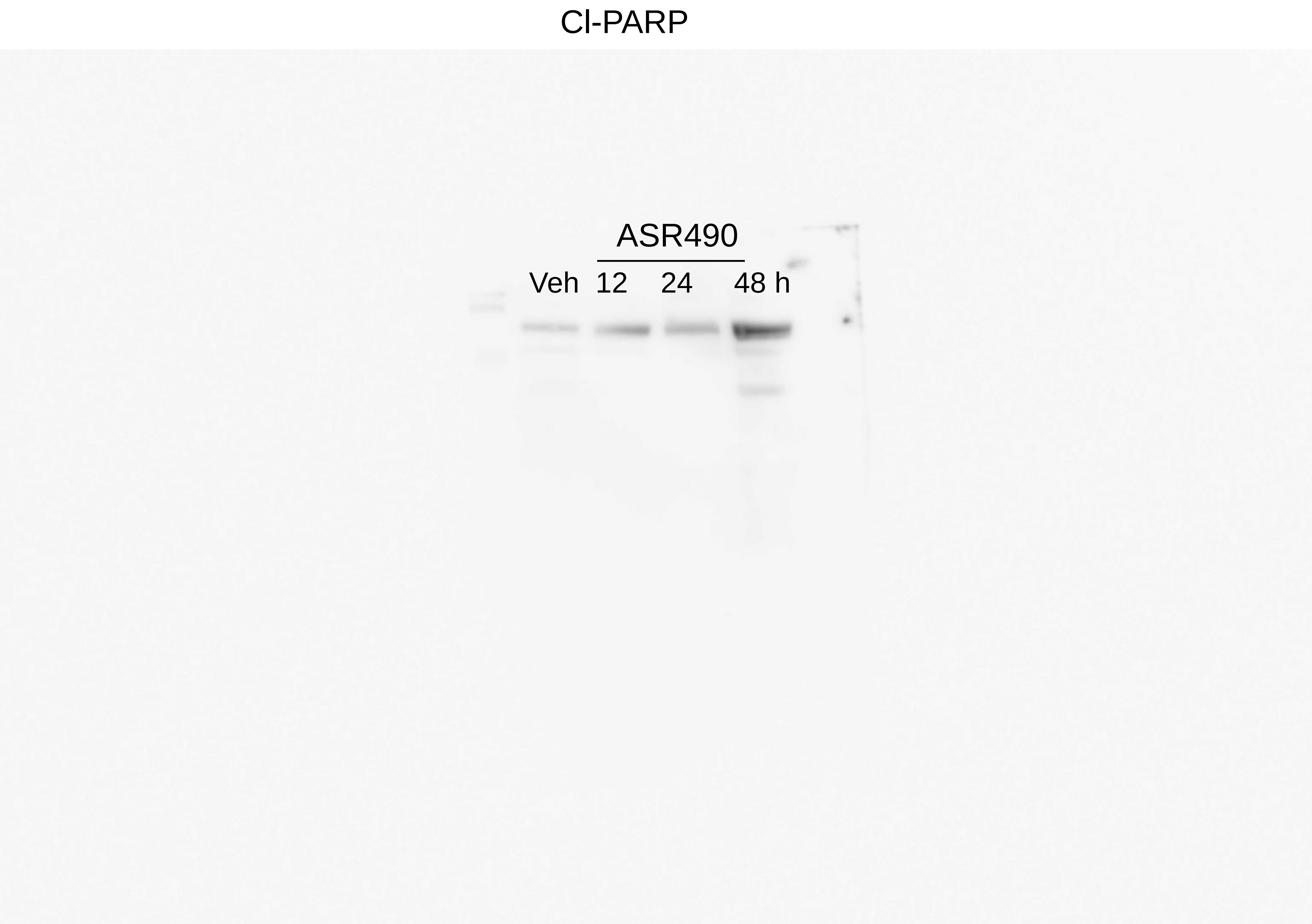

Cl-Cas 9

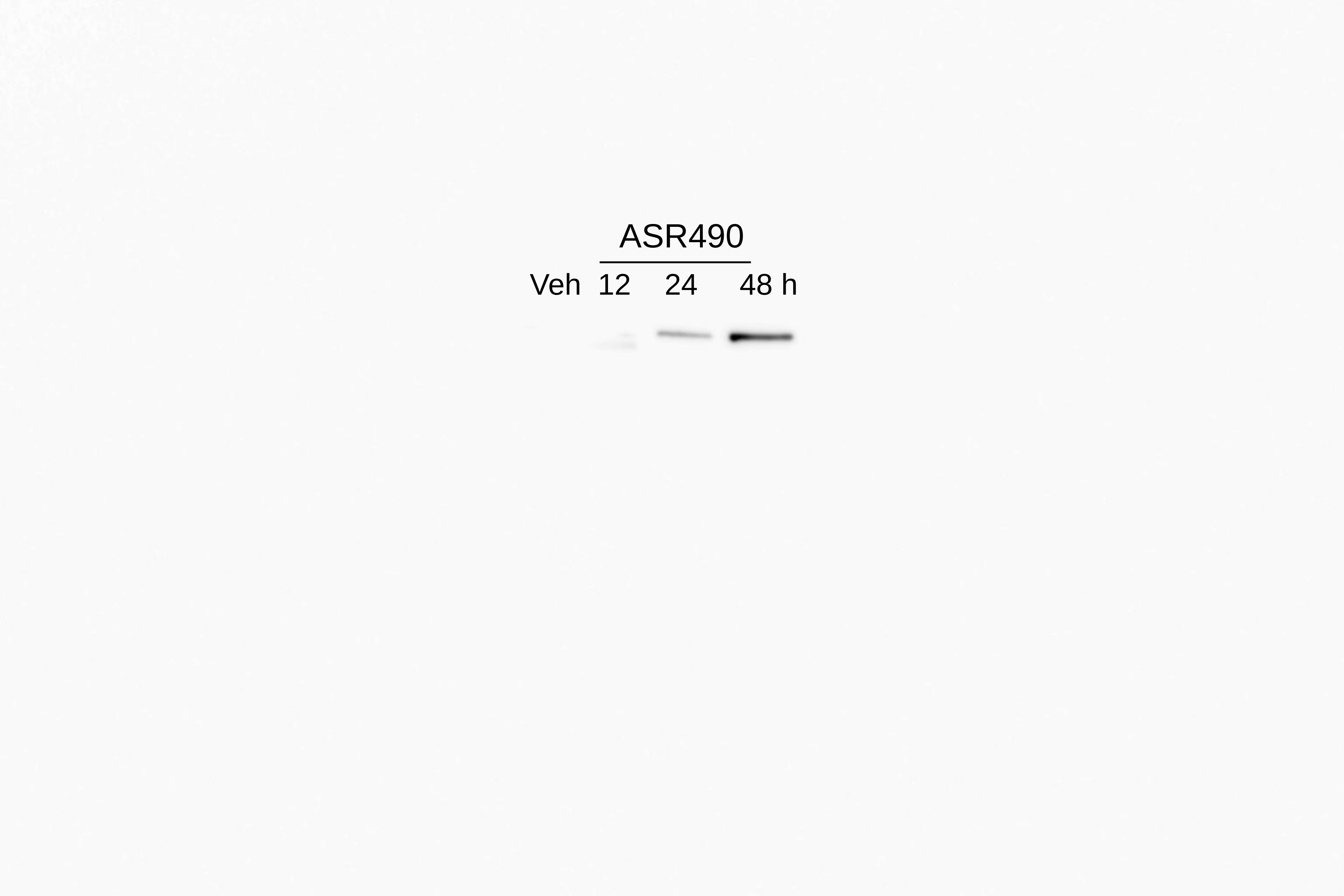

Bax

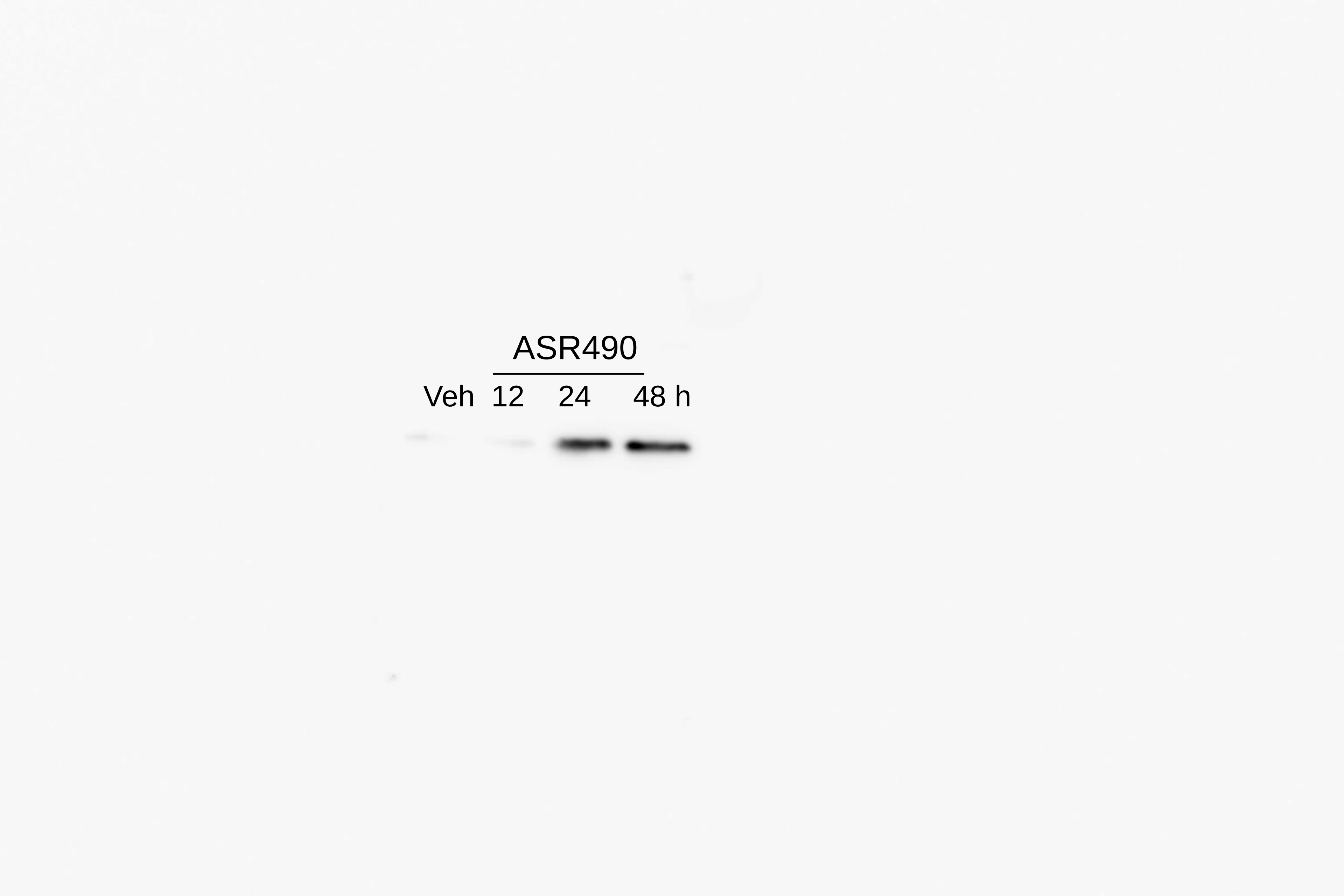

Actin

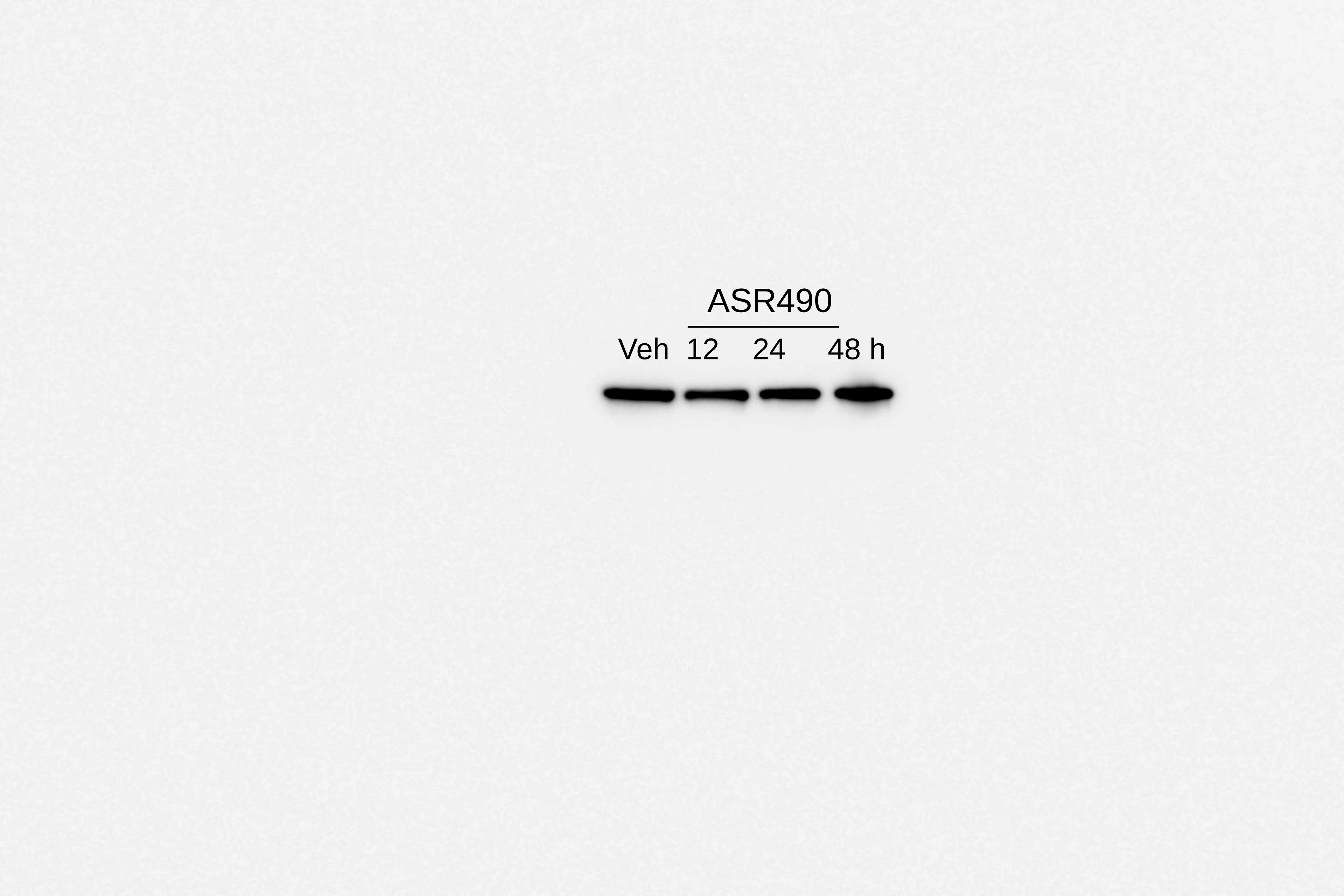

Figure 9E

NICD Notch1

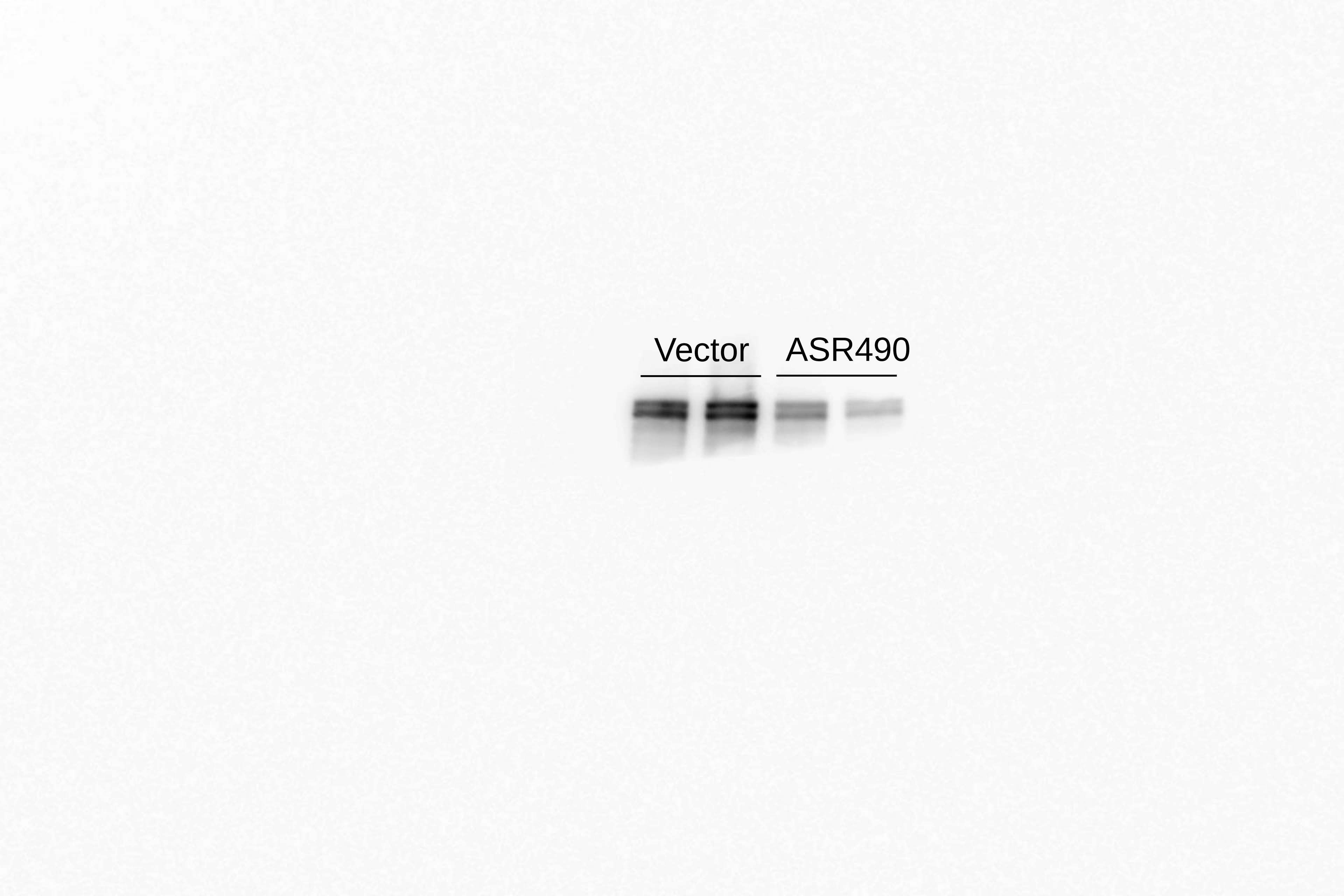

HES1

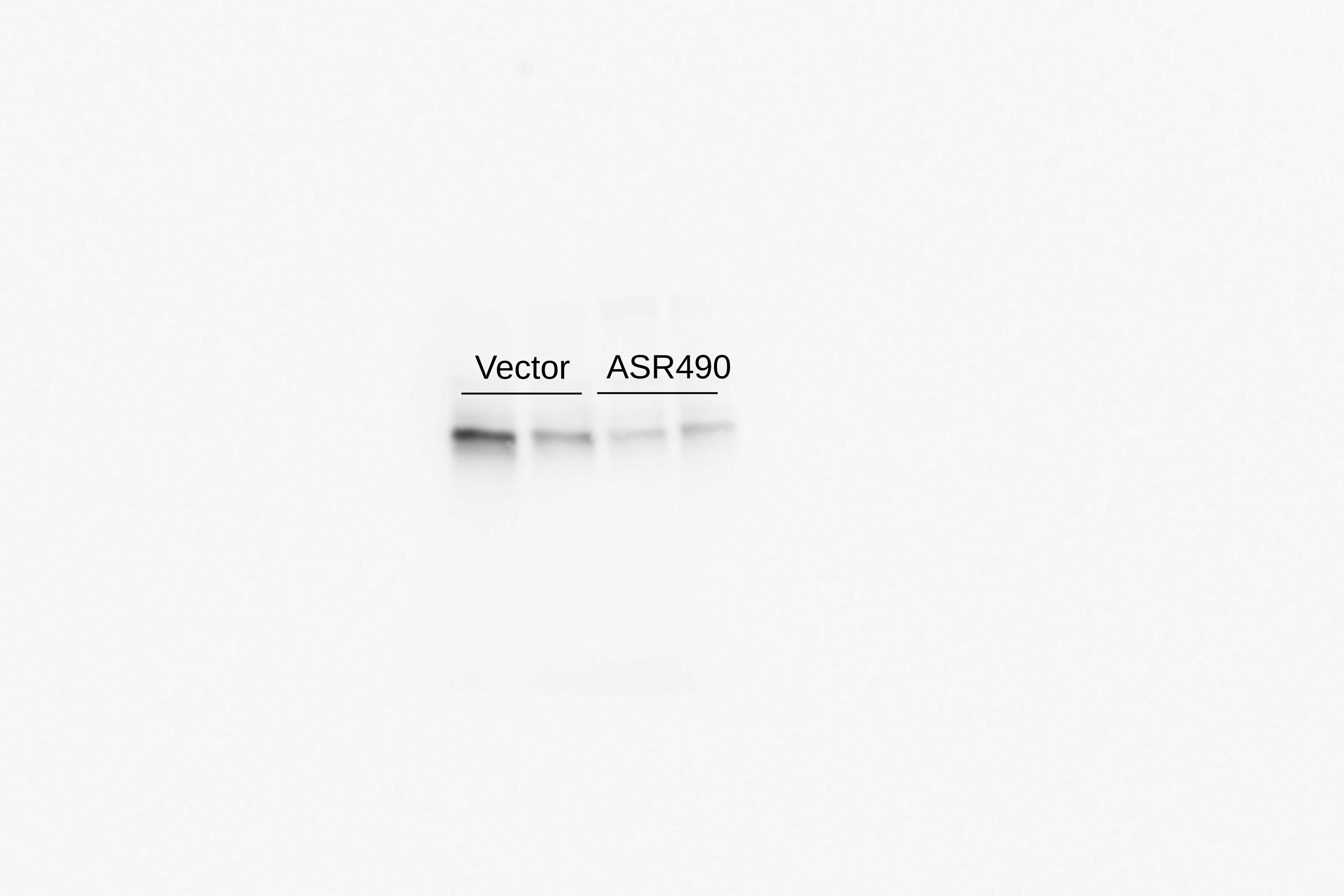

Actin

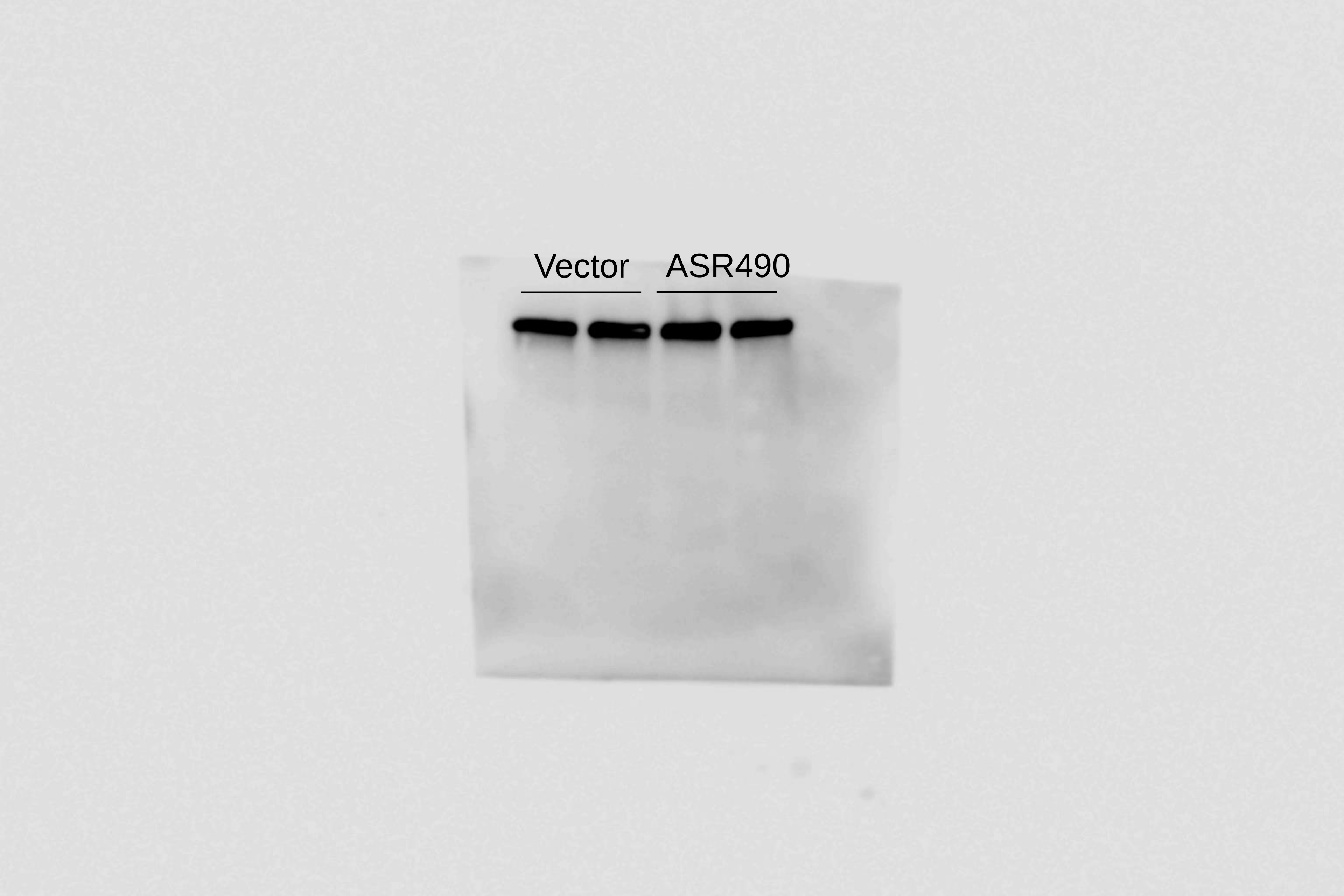

Figure 9F

NICD Notch1

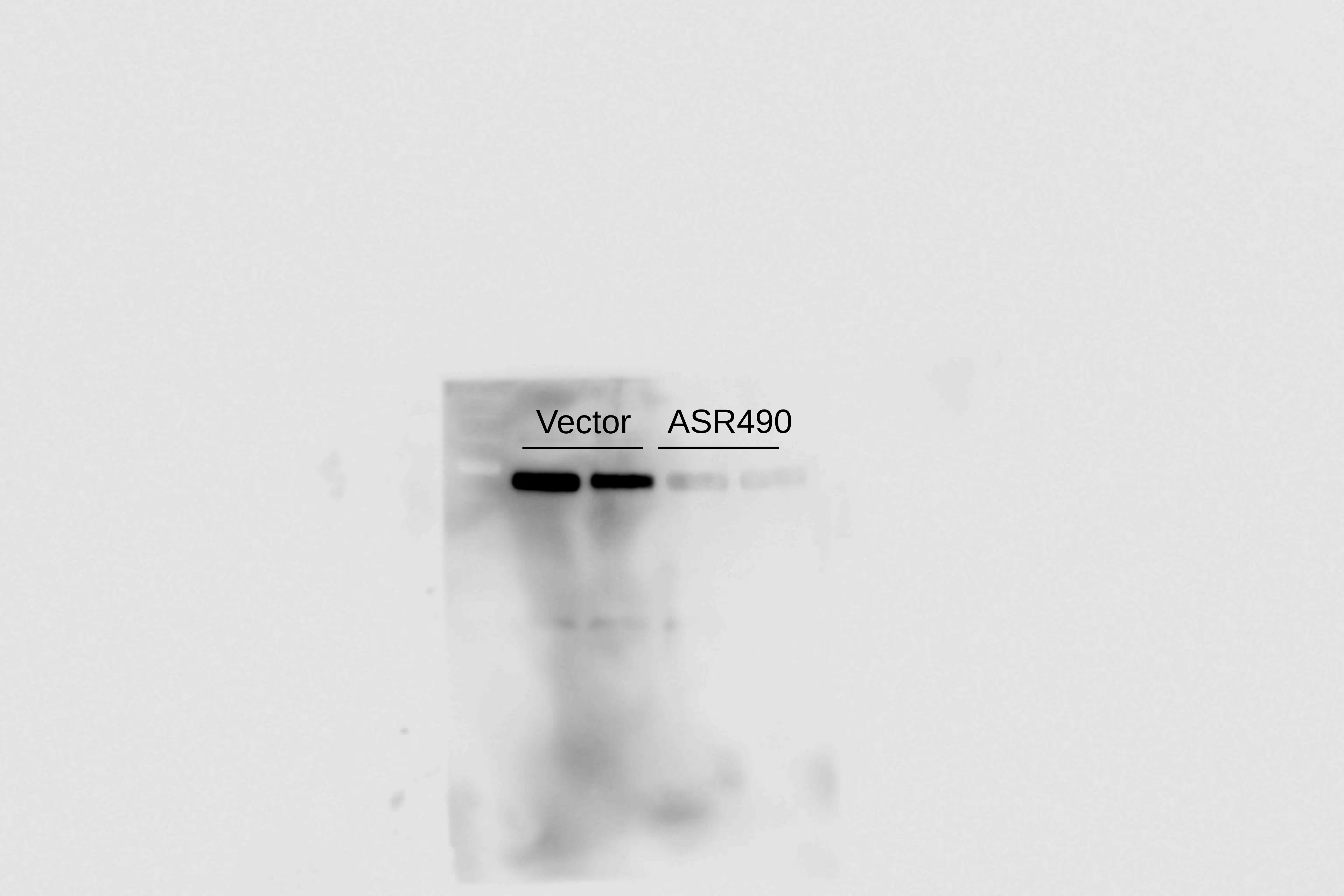

Hes1

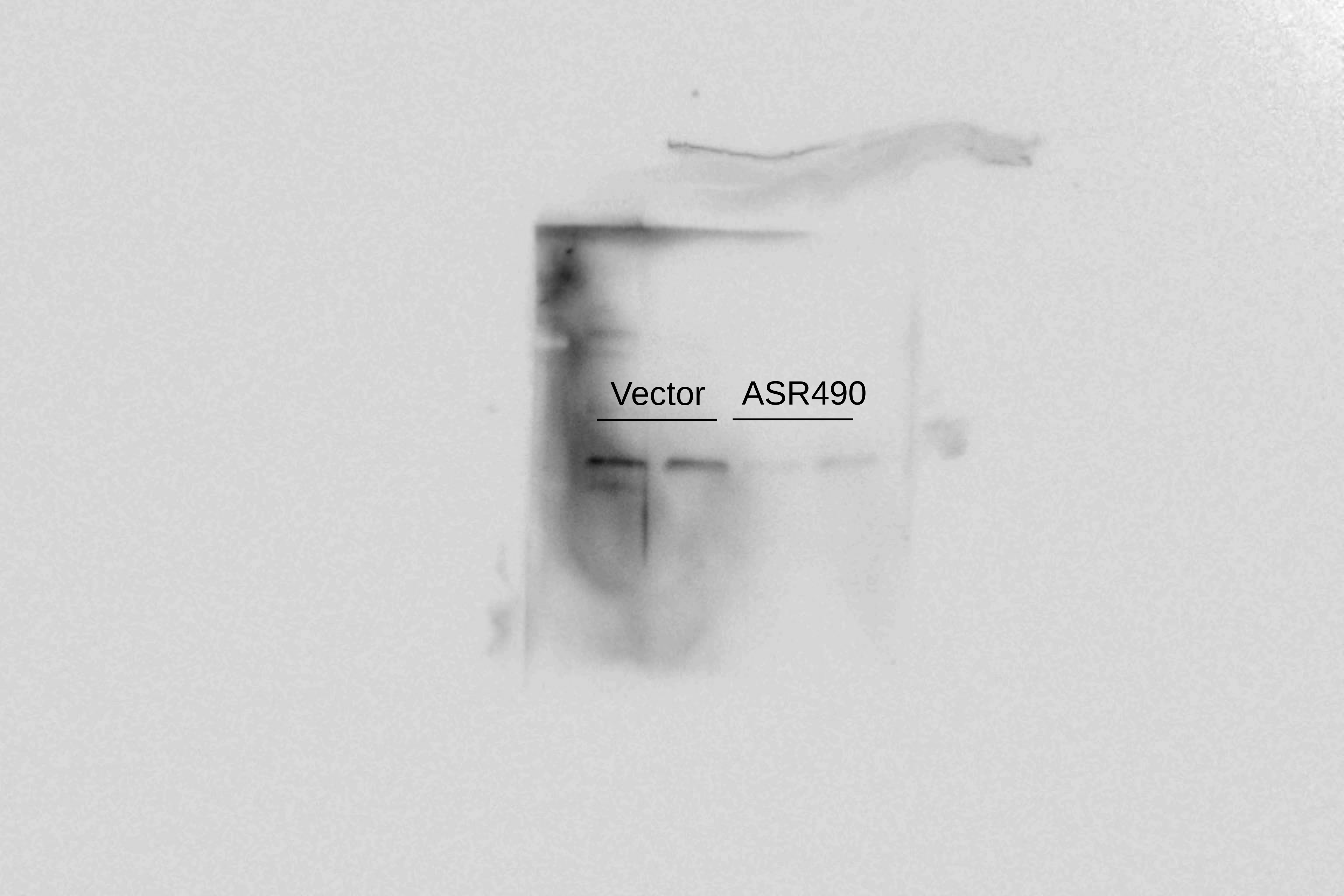

Actin

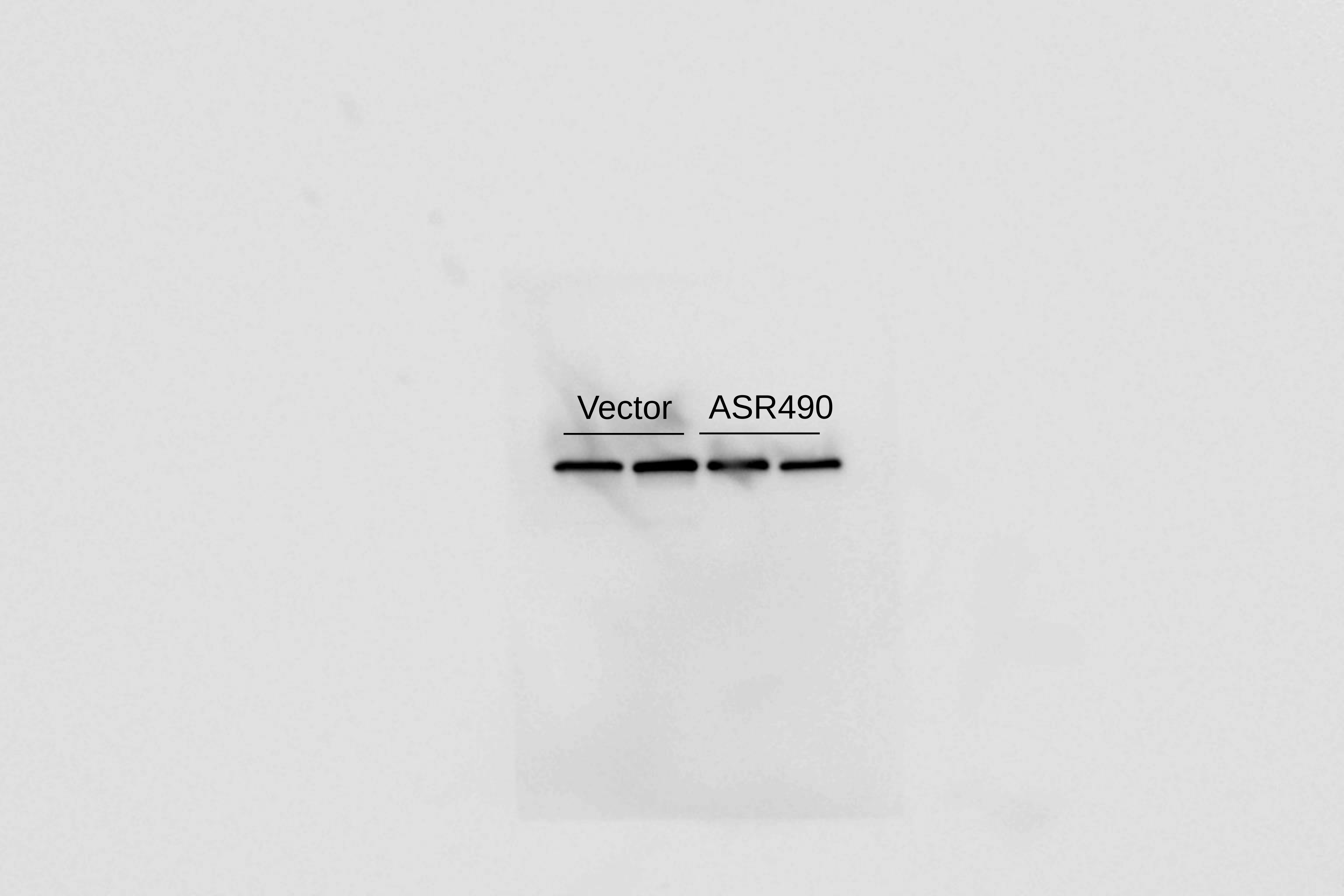

Supplement: Supplementary file 5 [file DataSheet1.pdf]
